# 1,2-Redox Transpositions of Tertiary Amides

**DOI:** 10.1021/jacs.3c08466

**Published:** 2023-09-27

**Authors:** Benjamin D. A. Shennan, Sergio Sánchez-Alonso, Gabriele Rossini, Darren J. Dixon

**Affiliations:** † Department of Chemistry, University of Oxford, Chemical Research Laboratory, 12 Mansfield Road, Oxford, OX1 3TA, U.K.

## Abstract

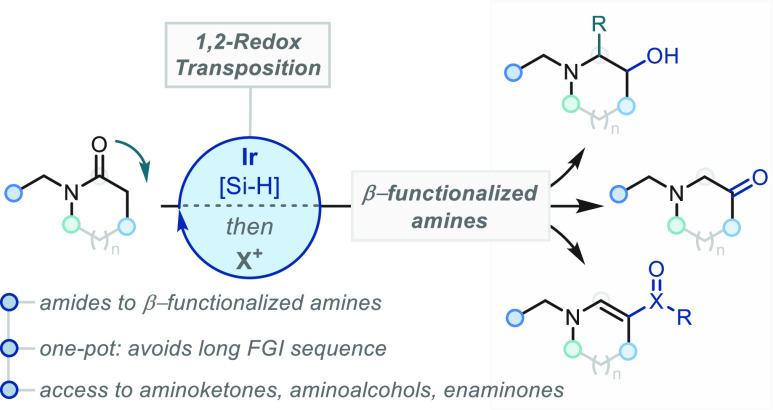

Reactions capable of transposing the oxidation levels of adjacent carbon atoms enable rapid and fundamental alteration of a molecule’s reactivity. Herein, we report the 1,2-transposition of the carbon atom oxidation level in cyclic and acyclic tertiary amides, resulting in the one-pot synthesis of 1,2- and 1,3-oxygenated tertiary amines. This oxidation level transfer was facilitated by the careful orchestration of an iridium-catalyzed reduction with the functionalization of transiently formed enamine intermediates. A novel 1,2-carbonyl transposition is described, and the breadth of this redox transposition strategy has been further explored by the development of aminoalcohol and enaminone syntheses. The diverse β-functionalized amine products were shown to be multifaceted and valuable synthetic intermediates, accessing challenging biologically relevant motifs.

Chemical reactions that dramatically transform the reactivity of molecules and bypass traditional sequences of functional group interconversion expedite synthetic routes and transform retrosynthetic logic. The emergent class of molecular skeletal editing reactions elegantly encapsulates such a concept, enabling highly coveted transformations to become a reality.^1^ A related yet comparatively under-explored avenue is the analogous approach towards redox editing of molecules. This reaction paradigm introduces transformations in which adjacent carbon atoms undergo complementary oxidation level transfers, obviating the need for single carbon atom oxidation level changes thereby curtailing nonstrategic and lengthy sequences of redox manipulations.^2^ Such a conceptual framework has been encapsulated elegantly in a seminal report from Dong and co-workers of a net redox-neutral sequence transposing ketone functionality and, more recently, in an oxidative rearrangement of 1,1-disubstituted alkenes to ketones, reported by Zhu.^3^

Given the ubiquity of amines throughout chemistry, the development of a related strategy to incorporate nitrogen-containing structural motifs would have far-reaching and immediate impact.^4^ In particular, the conversion of amides to β-functionalized amines represents a uniquely appealing strategy given the ubiquity of the amide functional group in known chemical space and the high occurrence of the amine products in natural products, pharmaceutical, and agrochemical molecules (Scheme 1A and 1B).^5^ Despite several prominent contemporary contributions to access β-functionalized amines,^6^ the predominating synthetic logic, from amides, remains amide α-deprotonation, electrophilic functionalization, and subsequent amide reduction. Such an approach is inherently limited by the functional group intolerance of the harsh deprotonation and reduction steps, by a limited scope of viable electrophiles and is unappealing from the point of view of step and redox economy.^7^ We envisioned a complete reversal of this sequence, initiated by a catalytic reduction of the amide to access a species at the enamine oxidation level (Scheme 1A). Guided by well-established principles in enamine reactivity,^8^ appropriately leveraging this enamine intermediate could enable direct access to β-functionalized amines (Scheme 1C).

**Scheme 1:**
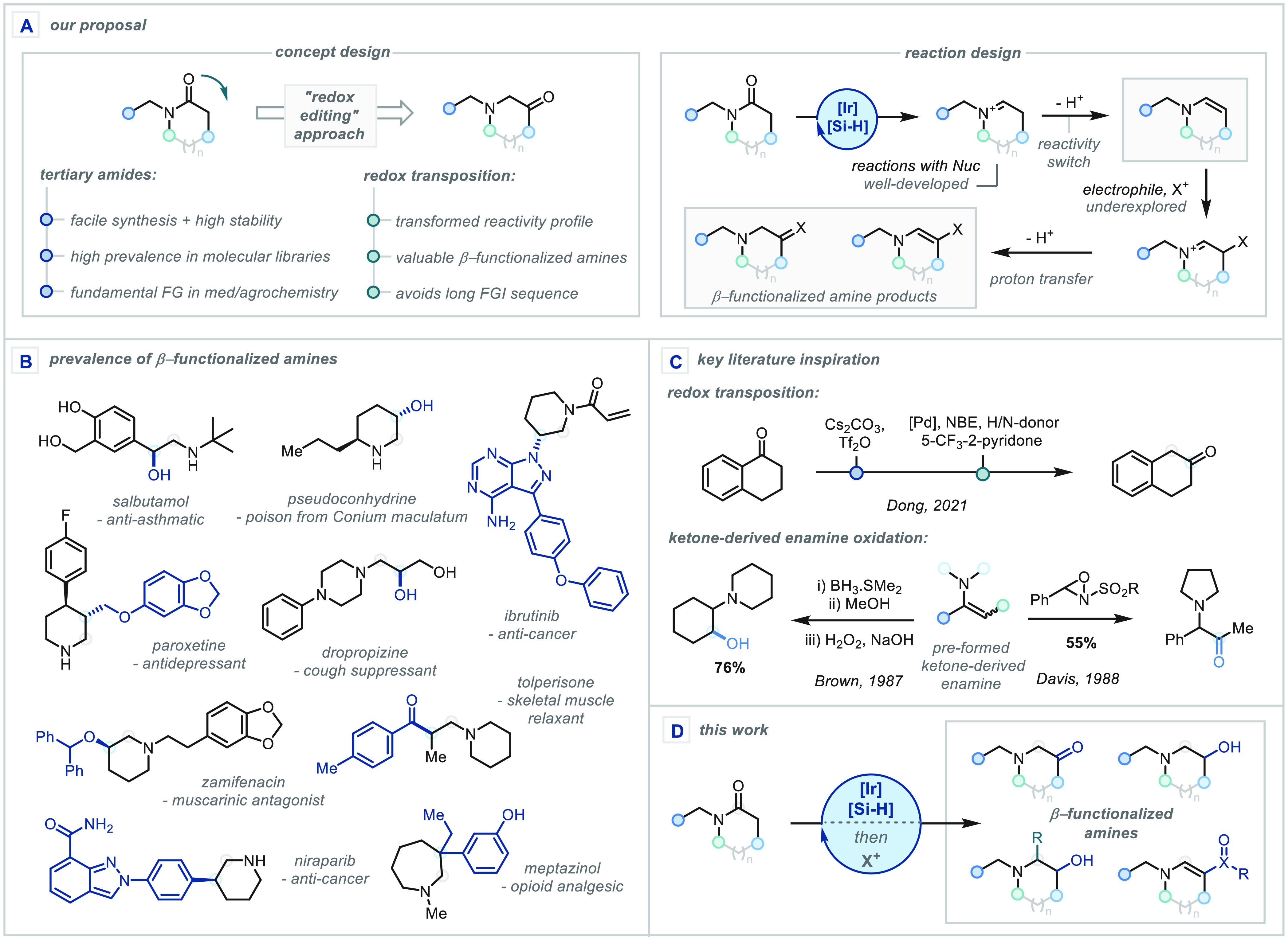
(A) Concept and Reaction Design for the Redox Transposition of Amides; (B) Prevalence of β-Functionalized Amines in Pharmaceuticals and Natural Products; (C) Key Literature Inspiration; (D) This Work (Tf, triflyl; NBE, a norbornene)

We were confident that the use of Vaska’s complex (IrCl(CO)(PPh_3_)_2_) in conjunction with a silane reductant would be ideally suited to such a strategy and were drawn to its under-explored potential as a means of accessing enamines. While the reductive functionalization of tertiary amides to α-substituted amines has been well-explored,^9^ the analogous approach to β-functionalized amines has remained limited to a few isolated reports on the generation of enamines.^9i,10^ In the seminal report from Nagashima, a series of stable enamines were generated from select substrates; however, limited product derivatization was demonstrated. Extending this concept, Adolfsson reported the synthesis of a number of functionalized heterocycles and highlighted the potential for installing β-oxygenation in such sequences. Both works, however, notably omitted extension to lactams and thereby fall short of accessing highly valuable N-heterocyclic products.

Taking inspiration from the Amadori and α-iminol rearrangements,^11^ we reasoned that appropriate β-oxidation of the enamine to the hydroxy-iminium ion should then follow a related mechanistic course and tautomerize to the thermodynamically more stable α-aminoketone. Indeed, enamine-type oxidation has been demonstrated on “N-deactivated” substrate classes, e.g. N-aryl indoles and N-Boc-protected heterocycles, and ketone-derived enamines; however, such oxidations remain limited to a narrow set of stable isolable enamines and are frequently accompanied by overoxidation (Scheme 1C).^12^ In our proposed one-pot strategy from the tertiary amide, this reaction would constitute an unprecedented 1,2-carbonyl shift, enabling the direct conversion of widely available amides to synthetically valuable α-aminoketones (Scheme 1D). Such a transformation could be further extended to β-acylation or other C–C and C–X bond forming reactions to broaden this redox transposition concept, facilitating diverse access to β-functionalized amines of fundamental importance in biomedical sciences.

Critical to achieving this reactivity was establishing conditions for the clean and reliable formation of stable enamine intermediates, in a manner amenable to both acyclic and lactam substrates. In order to investigate this goal, *N*-benzyl caprolactam **1a** was chosen as a model substrate and studied under standard Vaska’s reductive conditions [IrCl(CO)(PPh_3_)_2_ (1.0 mol %), tetramethyldisiloxane (TMDS, 1.5 equiv), C_6_D_6_ at room temperature and monitored via ^1^H NMR]. Following the reaction mixture in a time course experiment, after 10 min, no silylated hemiaminal was observed in the reaction mixture, but pleasingly, enamine **2a** was observed in near-quantitative conversion.^13^ This hemilabile species was observed to convert rapidly to a ring-opened dimeric dienamine **2b** (Scheme 2A) and to converge completely to this species as the sole reaction product, upon standing. This process likely occurs following acid-catalyzed generation of the iminium ion from the enamine and subsequent enamine-iminium addition (Scheme S2). To avoid this deleterious acid-mediated pathway, the reaction was repeated in the presence of diisopropylethylamine (DIPEA) and a marked improvement in enamine stability was observed.

**Scheme 2:**
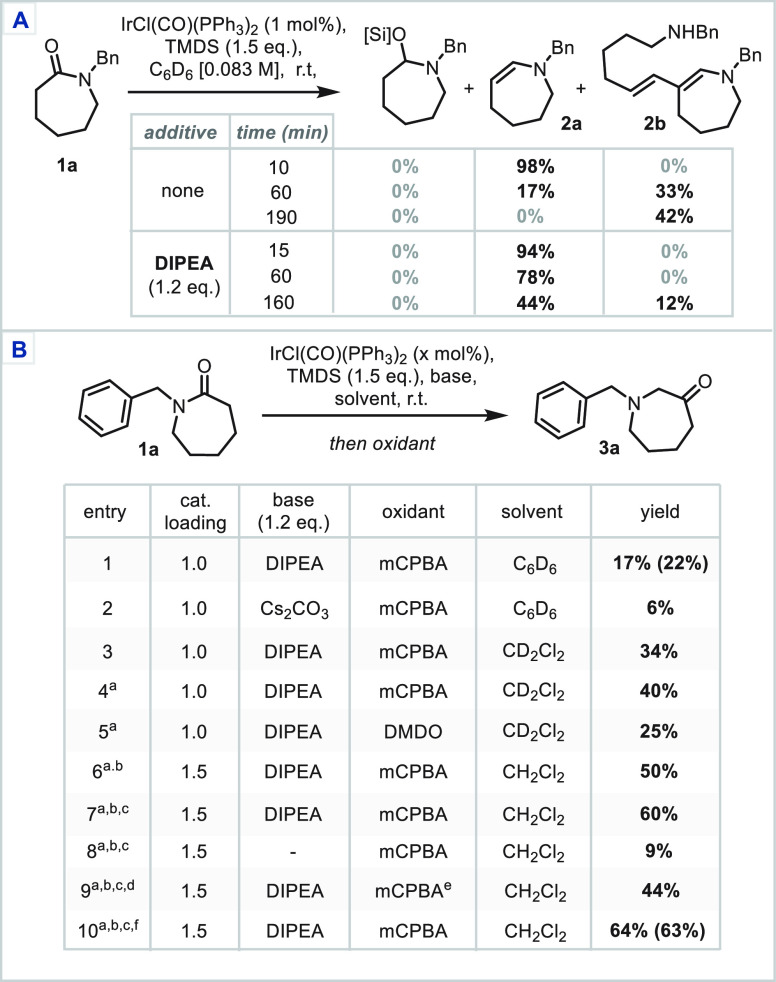
Optimization Studies: (A) Investigation into Enamine Stability; (B) Optimization of the Carbonyl Transposition Oxidant added at −78 °C. Reaction left for 16 h. *m*CPBA added as a CH_2_Cl_2_ solution. pH 5 aq. acetate buffer used in 2nd stage. Commercial *m*CPBA used. Reaction conducted with rigorous exclusion of air (see SI). TMDS, 1,1,3,3-tetramethyldisiloxane; DMDO, dimethyldioxirane; yields refer to ^1^H NMR yields employing 1,2,4,5-tetramethylbenzene as internal standard; isolated yields are given in parentheses.

Treatment of the in situ-generated enamine with *meta*-chloroperoxybenzoic acid (mCPBA) resulted in a very rapid consumption of the enamine to afford the desired aminoketone **3a**, albeit in low 17% yield (entry 1, Scheme 2B). The solvent, base, temperature, and stage timings of the reaction were optimized, enabling the ^1^H NMR yield to be increased to 50% (entries 2–6). Running the reaction with the rigorous exclusion of air and addition of the mCPBA as a CH_2_Cl_2_ solution to dilute the reaction mixture enabled the product to be isolated in 63% yield. Additional studies revealed that Brønsted acidic, Lewis acidic, or Lewis basic additives could not further improve the yield (see Supporting Information (SI) for details).

The scope of this 1,2-carbonyl transposition, with respect to the amide, was then investigated (Scheme 3A). *N*-Benzyl lactams (6-, 7-, and 8-membered rings) afforded the corresponding α-aminoketones in moderate to good yields (**3a**–**e**, **3m**–**n**). Interestingly, a small subset of substrates, possessing variously positioned electron-withdrawing groups, required longer times to bring about enamine formation and, following oxidation, rearrangement to the aminoketone products. By lengthening the reaction times, carbonyl transposition products bearing varied nitrogen and ring substitution (**3f**–**g** and **3i**–**l**) could be afforded in synthetically useful yields. Gratifyingly, despite potential instability in the corresponding products,^14^ acyclic amides could also be successfully employed to afford the desired α-aminoketones (**3p**–**r**). Notably, the reaction could be extended to complex natural product, natural product-like, and drug analogue scaffolds (**3r**–**3t**). Under the current reduction conditions, primary and secondary amides were not applicable; additionally, N-aryl substrates showed low conversion to the corresponding enamines and poor reactivity in the oxidation step.

**Scheme 3:**
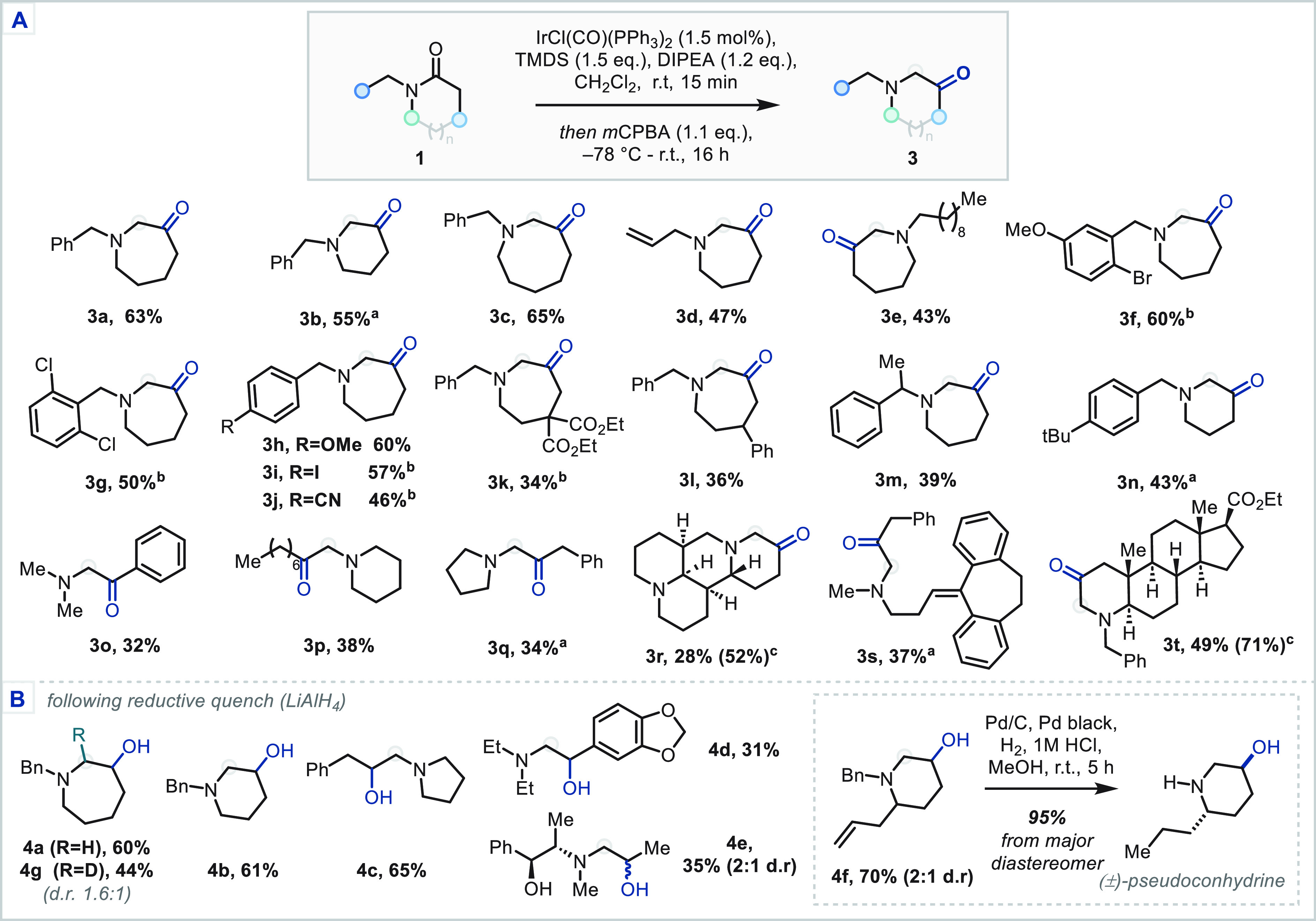
Scope of the 1,2-Carbonyl Transposition (A) and Reductive Modification (B) Reaction quenched at −78 °C with pH 5 aq. acetate buffer and stirred at rt for 2 h. Lengthened reaction times, see SI for details. NMR yield, in parentheses, as determined by ^1^H NMR using 1,2,4,5-tetramethylbenzene as internal standard—all other yields refer to isolated yields.

Suitable modification of the reaction conditions and introduction of an intermediate quench with a potent reductant successfully enabled the synthesis of β-amino alcohols via reduction of the putative β-hydroxy-iminium ion. For example, following the standard Ir-reduction/*m*CPBA-oxidation sequence on substrate **1a**, the reaction mixture was treated with LiAlH_4_ (4 equiv) as a 1 M solution in THF. Pleasingly, the resulting β-aminoalcohol **4a** was afforded in good yield (60%). NaBH_4_ in methanol could be employed in place of LiAlH_4_ with no change in yield. Using LiAlH_4_, this reactivity translated well across a series of lactams and amides (**4a**–**f**, Scheme 3B). Importantly, the 3-hydroxypiperidine motif, a fundamental building block reported over 5000 times in the patent literature, could be obtained in good yield (**4b**). The reductive transposition procedure was also shown to be effective in the synthesis of the bioactive β-hydroxyphenethylamine motif (**4d**). Pleasingly, substrates possessing free O–H groups were shown to be applicable by the successful reaction of (+)-pseudoephedrinepropionamide (**4e**). Targeting the synthesis of the hemlock-derived alkaloid pseudoconhydrine,^15^ an appropriate 6-allyl-2-piperidone could be afforded in two steps from the parent imide and the reductive transposition of this lactam proceeded in high yield and moderate diastereoselectivity to yield a 3-hydroxypiperidine **4f**. This was converted to the natural product (±)-pseudoconhydrine in high yield following hydrogenation. Finally, the reduction of the transiently formed β-hydroxy-iminium ion was supported by treatment instead with NaBD_4_ which afforded exclusively the α-deuterated azepane **4g**.

To further develop the utility of this 1,2-transposition strategy and to investigate the potential for C–C bond formation, we envisaged that the use of acid chlorides as electrophilic coupling partners could lead to α,β-unsaturated β-aminoketones (i.e., enaminones), thereby accessing high-value 1,3-oxygenated amine motifs. Such products have been highlighted as valuable intermediates in drug discovery and feature in a number of bioactive compounds.^16^ Acylation of transiently generated enamines to afford enaminones has been demonstrated in oxidative desaturation of amines;^17^ however, typically such reports have focused on the study of piperidines and rely on N-arylated substrates. Emboldened by this precedent, the reaction of enamine **2a** with benzoyl chloride was monitored by ^1^H NMR. A rapid reaction was observed, resulting in near-quantitative conversion to enaminone **5a**; an ethanolamine quench, and a cold aq. NH_4_Cl workup allowed the desired enaminone **5a** to be isolated in 71% yield.

Variation in both the ring size of the lactam and the N substituent afforded the corresponding cyclic enaminones in good yields (**5a**, **5g**–**j**, Scheme 4). Additionally, acyclic amides could be successfully applied (**5k**–**m**). A range of acyl chlorides were explored in this chemistry, demonstrating that the coupling proceeded efficiently with *para*-substituted benzoyl chlorides while lower yields were observed in the coupling of *ortho*-substituted aromatic and aliphatic acyl chlorides (**5b**–**f**). The reactivity extended well to aryl isocyanates to afford the corresponding enaminamides (**5q**–**u**), as well as to phenyl sulfonyl chloride and the Michael acceptor dimethyl acetylenedicarboxylate (**5o** and **5p**). Enaminone **5n** could be converted in good yield to muscle relaxant tolperisone following reduction with LiAlH_4_.^18^

**Scheme 4:**
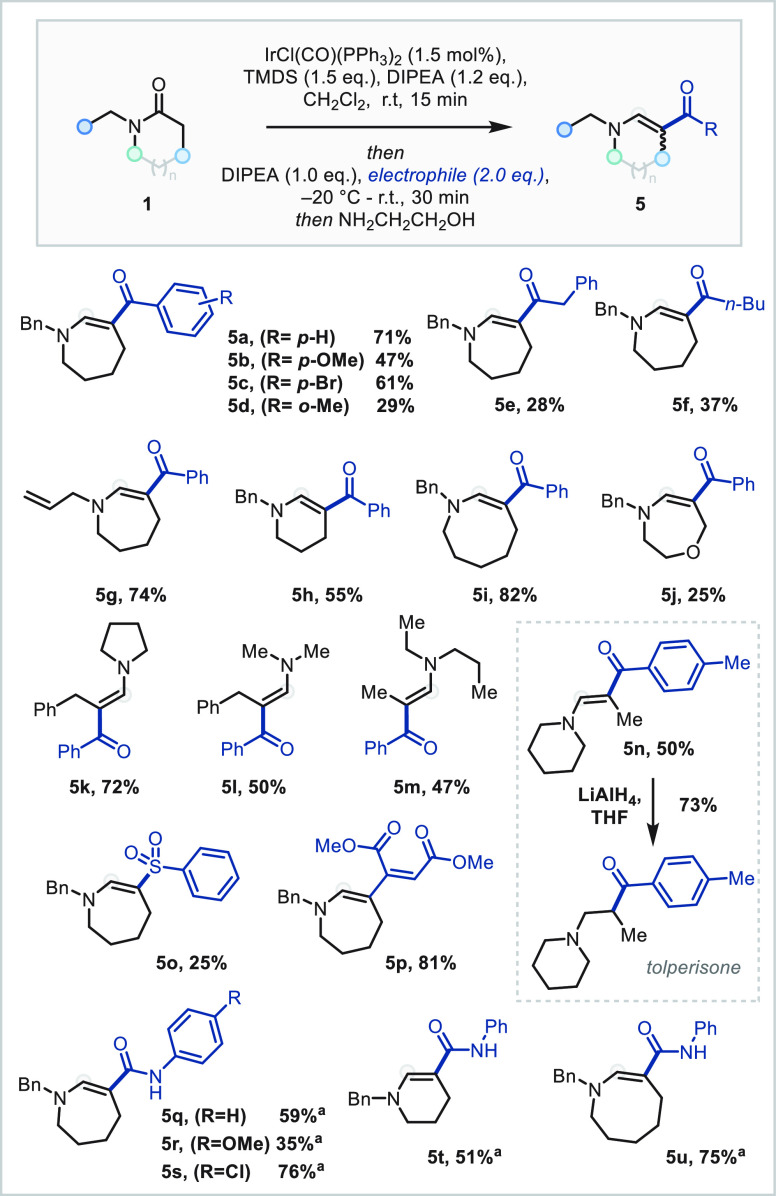
Scope of the β-Functionalization with Carbonyl Electrophiles Towards Enaminones and Related Products No additional DIPEA was added with electrophile.

To highlight the utility of the N-heterocyclic products, the carbonyl transposition reaction was up-scaled to 5 mmol with reduced catalyst loading (0.38 mol %, see SI for details), to afford aminoketone **3a** in unchanged 60% yield (Scheme 5A). Aminoketone **3a** reacted predictably under standard Grignard and Horner–Wadsworth–Emmons reaction conditions, enabling straightforward access to the corresponding β-amino tertiary alcohol (**6a**) and a γ-amino ester (**6b**), following hydrogenation of the corresponding α,β-unsaturated ester. Furthermore, the aminoketone functionality could be employed to access a series of medicinally relevant motifs, including: β-difluoroamines (**6c**) via deoxyfluorination, a β-aminonitrile moiety (**6d**) via a Van Leusen reaction, spirocyclic hydantoins (**6f**) via a high-yielding Bucherer–Bergs cyclization, and a high-value 2-aminoazepane building block (**6g**) via reductive amination/hydrogenation. Additionally, under Friedländer quinoline synthesis conditions, a 3,4-fused azepane-quinoline tricycle (**6e**) was afforded with excellent regioselectivity. Finally, a single step ring contraction of the aminoketone was demonstrated to afford the δ-lactam (**1b**).^19^ A one-pot lactam ring contraction from **1a** was additionally developed by a combination of the carbonyl transposition and the oxidative ring contraction using aq. H_2_O_2_ in conjunction with *m*CPBA.

**Scheme 5:**
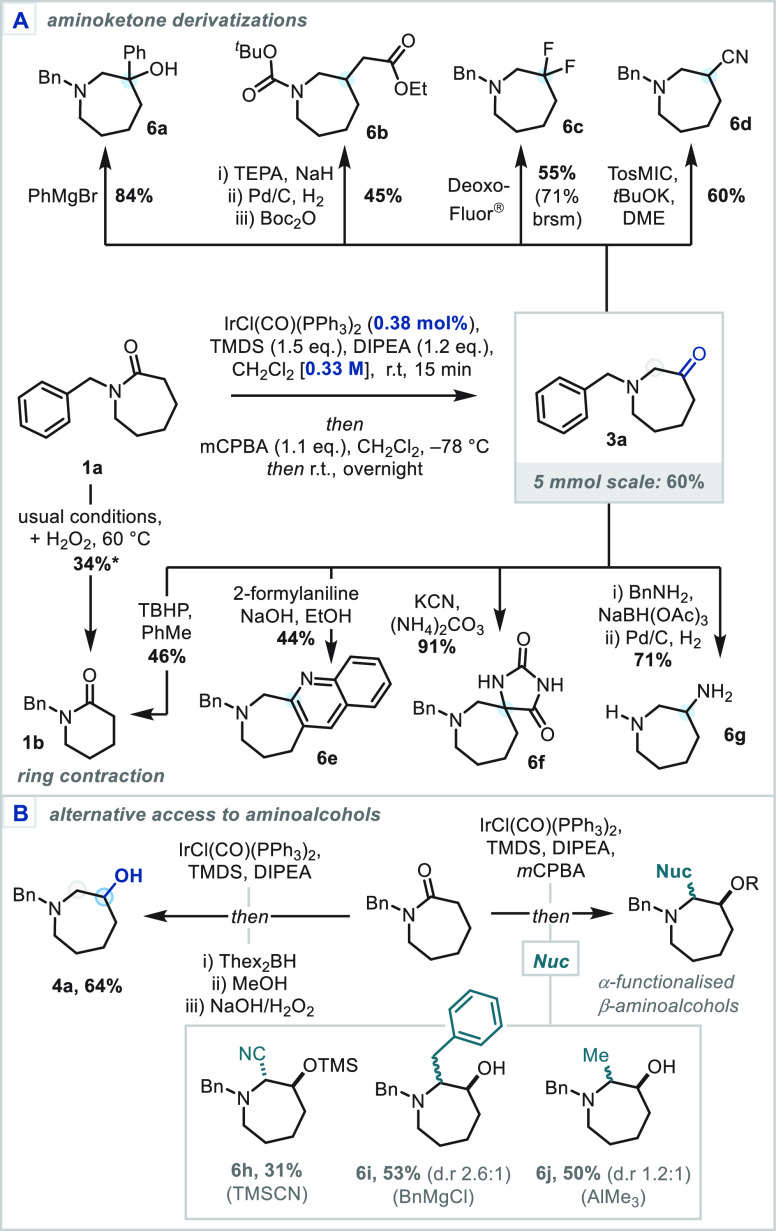
(A) Derivatization of Aminoketone Products; (B) Alternative Oxidative Capture of the Enamine Intermediates TEPA, triethyl phosphonoacetate; TBHP, *tert*-butyl hydroperoxide; Thex, *tert*-hexyl. *Determined by ^1^H NMR spectroscopy.

To investigate further the utility of this 1,2-redox transposition approach, the putative intermediate β-hydroxy-iminium ions were intercepted with a range of nucleophiles (Scheme 5B). Following the standard Ir-reduction/*m*CPBA-oxidation sequence, treatment with TMSCN afforded the desired β-siloxy-α-aminonitrile as a single diastereomer (**6h**);^20^ Grignard and organoaluminum reagents afforded the corresponding branched β-aminoalcohols as diastereomeric mixtures (**6i**–**j**). Finally, a complementary reductive transposition was demonstrated, following inspiration from Brown and Singaram,^10d,12n,12p^ utilizing an Ir-catalyzed reduction/hydroboration/oxidation sequence to access aminoalcohol **4a** in good yield.

In conclusion, a new strategy for the single-step redox editing of tertiary amides is described. Through judicious choice of an electrophilic coupling partner, a diverse set of 1,2- and 1,3-oxygenated amines could be afforded directly from tertiary amides following an Ir-catalyzed hydrosilylation. Most notably, an unprecedented 1,2-carbonyl transposition has been demonstrated, leading to α-aminoketone products that were shown to be robust intermediates for diverse downstream transformations. Furthermore, β-aminoalcohols and enaminones were also accessed by modification of the electrophilic coupling partner, enabling the synthesis of bioactive and pharmaceutically relevant molecules. Our hope is that this single step “redox-shuffling” strategy will provide new retrosynthetic logic toward polyfunctionalized saturated and semisaturated N-heterocycles thereby expediting the synthesis of bioactive molecules of fundamental importance.

## Supporting Information Available

Additional optimization data, full synthetic methods, and characterization data are available in the Supporting Information. (PDF)
