# Supplementary material for: 1,2-Redox Transpositions of Tertiary Amides
Source: J Am Chem Soc. 2023 Sep 27;145(40):21745–51. doi: 10.1021/jacs.3c08466 (PMC10571086; doi:10.1021/jacs.3c08466)

Supporting Information

for

1,2-Redox Transpositions of Tertiary Amides

Benjamin D. A. Shennan,[1] Sergio Sánchez-Alonso,[1] Gabriele Rossini,[1] Darren J. Dixon[*,1]

[1] Department of Chemistry, Chemistry Research Laboratory, University of Oxford,

12 Mansfield Road, Oxford OX1 3TA, UK

E-mail: darren.dixon@chem.ox.ac.uk

1. General information 2

2. Optimisation data 4

3. Proposed Mechanism 6

4. Unsuccessful examples 7

5. Synthetic procedures 8

5.1 General procedures 8

5.2 Starting material synthesis 10

5.3 Aminoketone synthesis 21

5.4 Aminoalcohol synthesis 30

5.5 Synthesis of enaminones and derivatives 34

5.6 Derivatizations and additional enamine reactivity 45

6. Reaction Troubleshooting and further comments 53

7. ESI References 55

8. NMR Spectra 56

1. General information

Proton, carbon and fluorine NMR spectra were recorded on Bruker 400 MHz (^{1}H NMR at 400 MHz and ^{13}C NMR at 101 MHz unless otherwise specified). Chemical shifts for protons are reported in parts per million downfield from $Si(CH_3)_4$ and are referenced to residual protium in the deuterated solvent ($CHCl_3$ at 7.26 ppm, unless otherwise specified). NMR data are presented in the following format: chemical shift (multiplicity [app = apparent, br = broad, d = doublet, t = triplet, q = quartet, dd = doublet of doublets, dt = doublet of triplets, dq = doublet of quartets, ddd = doublet of doublet of doublets, m = multiplet], number of equivalent nuclei by integration, coupling constant [in Hz]). Diastereomeric ratios were determined by analysis of ^{1}H NMR spectrum of the unpurified mixture following work-up unless otherwise specified. High-resolution mass spectra (ESI) were recorded on an ACQUITY I-Class PLUS UPLC System (Waters, Milford, MA, USA) coupled to an ACQUITY RDa mass spectrometer (Waters, Milford, MA, USA) equipped with an ESI probe, in positive ion mode. The flow rate was set to 0.300 mL/min using a 50% methanol (aq) + 0.1% formic acid eluent. Scan parameters were set as follows: analyzer mode, full scan; scan range, 50-2000 m/z; scan rate, 2 Hz; cone voltage, 30 V; capillary voltage, 1.5 kV; desolvation temperature, 550 ºC; and intelligent data capture, on. Infrared spectra were recorded on a Bruker Tensor 27 FT-IR spectrometer as a thin film. Only selected maximum absorbances are reported (in ν_{max} (cm^{-1})). Melting points were obtained on a Leica Galen III Hot-stage melting point apparatus and microscope and on a Kofler hot block and are reported uncorrected. Analytical thin-layer chromatography (TLC) was performed on Merck silica gel 60 F254 plates and visualized with UV light (254 or 365 nm), and/or a vanillin stain or basified aq. $KMnO_4$. Silica gel column chromatography was performed using 60 Å silica gel 40-63 µm purchased from VWR. Isolated yields are given for samples obtained at >90% purity by NMR – in select cases analytical data is presented following further purification for clarity. All reactions were performed using reagents obtained from Sigma-Aldrich, Acros Organics, Alfa Aesar, STREM or Fluorochem without further purification unless stated. All water used was purified through a Merck Millipore reverse osmosis purification system prior to use. Anhydrous dichloromethane and diethyl ether were dried by filtration through activated alumina (powder ~150 mesh, pore size 58 Å, basic, Sigma-Aldrich) columns and stored over 3 Å molecular sieves under an atmosphere of N_2 prior to use. Anhydrous THF and toluene was obtained from Acros Organics and stored under AcroSeal® over molecular sieves. Deuterated solvents were used as supplied. Reactions were performed under a balloon of N_2 if not stated. Temperatures quoted are external. Solvents were removed under reduced pressure using Büchi Rotavapor apparatus.

2. Optimisation data

Table S1A, NMR study investigating the carbonyl transposition of N-benzyl caprolactam, reactions conducted on 0.05 mmol; [a] oxidant added at –78 °C; [b] reaction run at 0.042 M; [c] oxidation procedure following ref [1]; Table S1B, reaction optimization for the carbonyl transposition of 1a; yields refer to NMR yields using 1,2,4,5-tetramethylbenzene as internal standard unless specified; [d] reaction left for 16 hours following oxidation; [e] 60 µL MeOH added before oxidant; [f] isolated yield; [g] *m*CPBA added as a CH_2Cl_2 solution; [h] reaction conducted with rigorous exclusion of air.

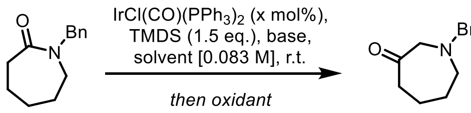

entry	cat. loading	base	oxidant	solvent	yield
1	1.0	DIPEA	*m*CPBA	C_6D_6	17% (22%)[f]
2	1.0	DIPEA	**Ox-1**	C_6D_6	*trace*
3	1.0	**TMP**	*m*CPBA	C_6D_6	*n.d.*
4	1.0	**Cs_2CO_3**	*m*CPBA	C_6D_6	6%
5	1.0	DIPEA	**Oxone**	C_6D_6	*n.d.*
6	1.0	DIPEA	**O_2**	C_6D_6	*n.d.*
7	1.0	DIPEA	*m*CPBA	**CD_2Cl_2**	34%
8[a]	1.0	DIPEA	*m*CPBA	CD_2Cl_2	40%
9[a]	1.0	DIPEA	*m*CPBA	**d_4-THF**	22%
10[a]	1.0	DIPEA	***m*CPBA (2 eq.)**	CD_2Cl_2	12%
11[a,b]	1.0	DIPEA	*m*CPBA	**CD_2Cl_2**	*n.d.*
12[a,c]	1.0	DIPEA	**DMDO**	CD_2Cl_2	25%
13[a,d]	**1.5**	DIPEA	*m*CPBA	CD_2Cl_2	50%
14[a,d]	1.5	DIPEA	**Ox-2**	CD_2Cl_2	0%
15[a,d,e]	1.5	DIPEA	**Ox-2**	CD_2Cl_2	35%
16[a,d]	1.5	DIPEA	**Ox-3**	CD_2Cl_2	40%
17[a,d]	1.5	**DBU**	*m*CPBA	CD_2Cl_2	25%

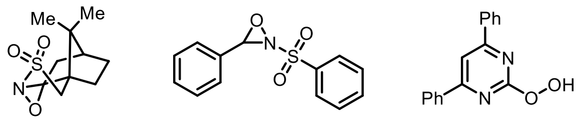

Ox-1 **Ox-2** **Ox-3**

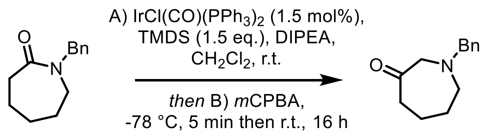

entry	variation from standard conditions	yield
1	-	50%
2	-20 °C for B	13%
3	40 °C for B	25%
4	*m*CPBA added as EtOH sol.	36%
5	*m*CPBA added with hexane cosolvent	15%
6[g]	-	60%
7	menthol (1 eq.) in B	40%
8	$MeCO_2Na$ (3 eq.) in B	40%
9	PPh_3 (3 eq.) in B	43%
10	no DIPEA	9%
11	commercial *m*CPBA	35%
12	commercial *m*CPBA, pH 5 buffer	44%
13[g,h]	-	64% (63%)[f]
14[g,h]	pH 5 buffer after B	51%
15[g,h]	pH 6.5 buffer after B	31%
16[g,h]	pH 8 buffer after B	33%
17[g,h]	pH 9.5 buffer after B	44%
18[g,h]	B) 40 °C, 3 h	56%
19[g,h]	$Sc(OTf)_3$ (0.1 eq) after B	53%
20[g,h]	TMSCl *then* HCl after B	43%
21[g,h]	TMSOTf *then* HCl after B	50%
22[g,h]	$BF_3.OEt_2$ after B	48%

Figure S1. NMR time-course experiments showing the reduction of lactam 1a; A) in the absence of DIPEA; B) in the presence of DIPEA

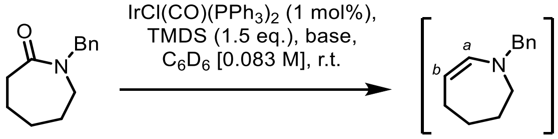

A (no base)

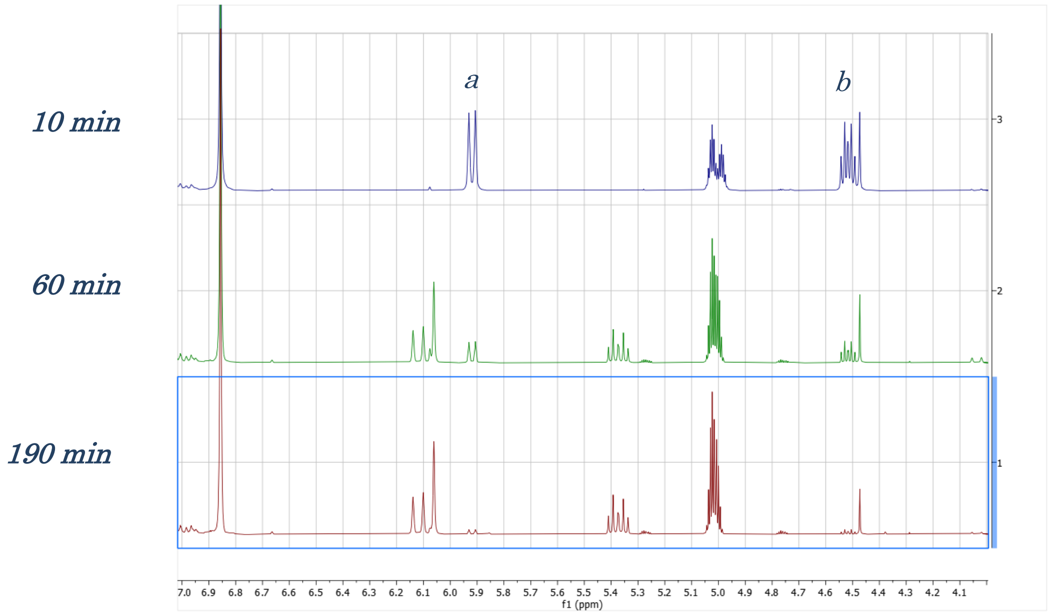

B (+DIPEA)

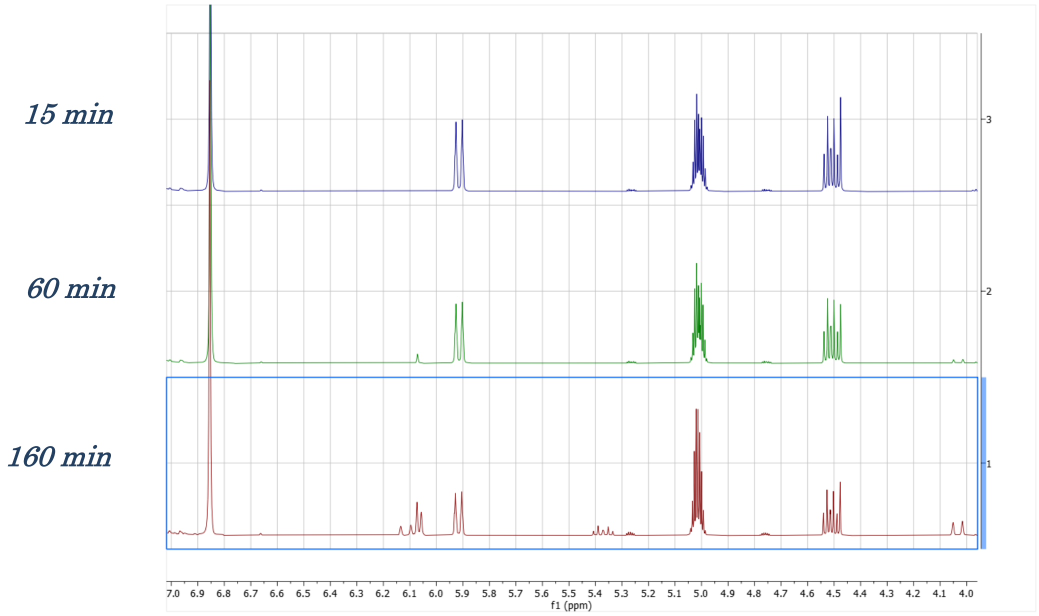

3. Proposed Mechanism

Scheme S1. Proposed mechanism for carbonyl transposition procedure

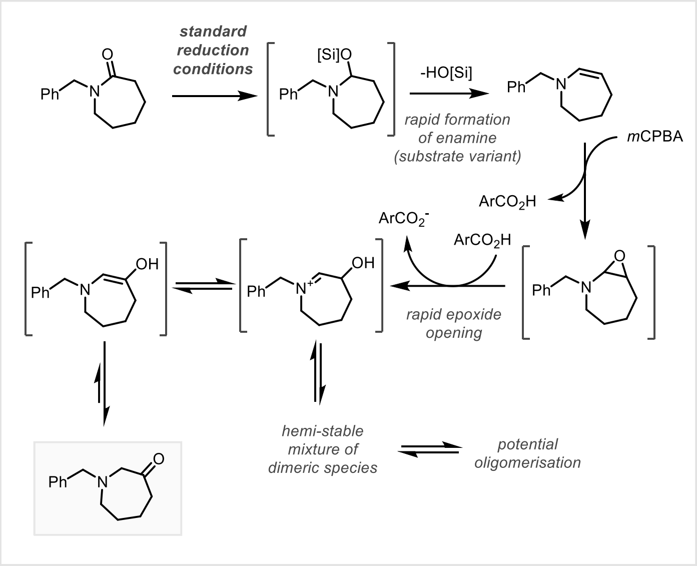

Scheme S2. Proposed mechanism for formation of the dienamine side-product

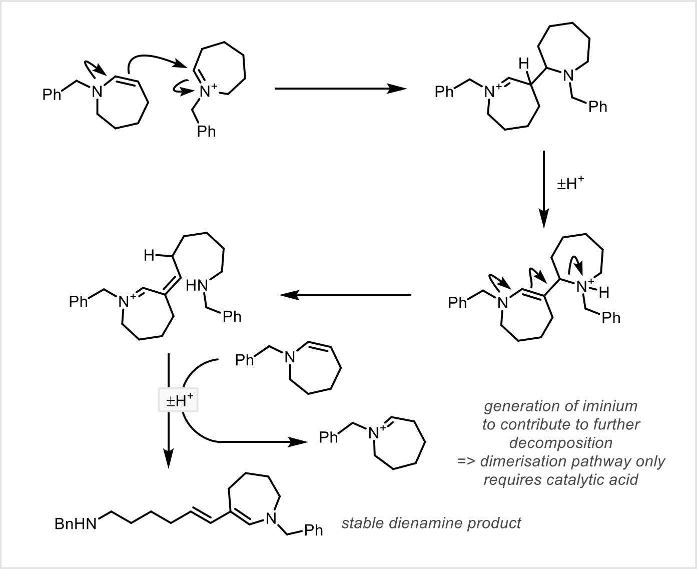

Characterization data for **2b**

IR (film) ν_{max}/cm^{-1} 2931, 1673, 1495

^{1}H NMR* (400 MHz, C_6D_6) δ 7.31 (m, 2H), 7.24 – 6.97 (m, 8H), 6.12 (d, *J* = 15.3 Hz, 1H), 6.06 (s, 1H),

5.38 (dt, J = 15.3, 7.0 Hz, 1H), 3.78 (s, 2H), 3.61 (s, 2H), 2.87 – 2.77 (m, 2H), 2.49 (t, J = 6.8 Hz, 2H), 2.46 – 2.38 (m, 2H), 2.21 (qd, J = 6.5, 5.2 Hz, 2H), 1.71 – 1.59 (m, 2H), 1.53 – 1.40 (m, 6H).

^{13}C NMR* (101 MHz, C_6D_6) δ 142.2, 141.3, 140.0, 135.3, 128.7, 128.6, 128.5, 127.9, 127.4, 127.1, 119.9, 114.6, 62.0, 54.3, 53.3, 49.7, 33.7, 30.1, 30.0, 28.6, 27.3, 26.4

HRMS (ES+) exact mass calculated for $[M+H]^+$ ($C_{26}H_{35}N_2$) requires m/z 375.2795, found m/z 375.2786

**NMR data represented are crude, taken from reaction mixture (depicted in Figure S1A), after reaction was left for 24 hours.*

4. Unsuccessful examples

Figure S2. Unsuccessful scope examples in redox transposition with comments

substrate	*comment*
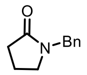	- enamine was monitored before reaction and considerable decomposition was observed at 15 minutes<br>- a rapid oxidation procedure was trialled however no product was observed suggesting greater instability of the transient intermediates
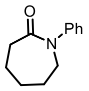	- highly stable hemi-aminal (100% after 2 hours)<br>- high degree of decomposition when hemiaminal treated with Lewis acids (16% enamine)
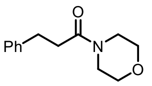	- very slow collapse to enamine (>16 hours) therefore substrate not trialled in oxidation step
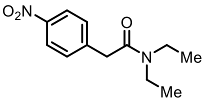	- rapid formation of enamine but no observed reaction of enamine with mCPBA even following isolation and re-treatment
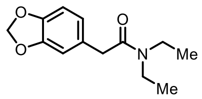	- clear product formation (30-35% NMR) however decomposition of product during isolation (likely via oxidation with O_2)
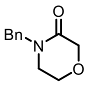	- slow formation of enamine (78% after 4 hours) but following oxidation only a complex mixture was observed
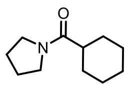	- crude NMR shows only aldehyde (38%) - likely due to iminium ion intermediate unable to proceed via usual mechanism and hydrolysing upon work-up

5. Synthetic procedures

5.1 General procedures

General Procedure 1: Lactam alkylation

A three-necked round bottomed flask was evacuated and back-filled with N_2 three times before being charged with NaH (60% dispersion in paraffin oil, 1.13 eq.). The NaH was washed once with hexane (10 mL), before THF (0.56M or 0.20 M) was added. The suspension was stirred and cooled to 0 °C, at which point the secondary lactam (1 eq.) was added in one portion. The mixture was allowed to come naturally to room temperature over ~1 h and stirred at room temperature for a further hour. The alkyl bromide (1.07 eq.) was added and the mixture was stirred at room temperature and starting material consumption was monitored by TLC. When complete consumption was observed (typically 4 – 16 hours), H_2O (20 mL) was added and the biphasic mixture was extracted with EtOAc (3 x 50 mL). The combined organic phases were dried with Na_2SO_4, filtered and concentrated under reduced pressure. The crude residue was purified by FCC (pentane:EtOAc or pentane:Et_2O).

General Procedure 2: Lactam alkylation B

Following a modified literature procedure,[2] to a solution of secondary lactam (1 eq.) in DMF (0.64 M), cooled to 0 °C, was added NaH (60% dispersion in paraffin oil, 1.2 eq.) and alkyl halide (1.2 eq.). The mixture was stirred at room temperature overnight before the addition of H_2O (0.64 M). The aqueous solution was extracted with Et_2O (3 x 50 mL) and the combined organics were washed with brine (50 mL). The combined organics were dried with Na_2SO_4, filtered and concentrated under reduced pressure. The crude residue was purified by FCC (pentane:EtOAc or pentane:Et_2O).

General Procedure 3: Amide coupling

To a solution of the carboxylic acid (1 eq.), secondary amine (1.2 eq.), DIPEA (1.2 eq.), in CH_2Cl_2 (0.11 M), was added HOBt.H_2O (1.2 eq.) and EDC (1.2 eq.). The resulting mixture was stirred overnight before addition of H_2O. The resulting biphasic mixture was extracted with CH_2Cl_2 (3 x 50 mL). The combined organics were dried with Na_2SO_4, filtered and concentrated under reduced pressure. The crude residue was purified by FCC (pentane:Et_2O).

General Procedure 4: 1,2-Carbonyl transposition

To a 10 mL vial equipped with a magnetic stirring bar was added the amide (0.2 mmol). The vial was capped with a septum and evacuated and backfilled with N_2 three times. The N_2 line was then exchanged for a N_2 balloon. A freshly prepared solution of Vaska's catalyst (1.5 mol%, 2.4 mg) and DIPEA (1.2 eq.,

41.6 µL, 0.24 mmol) in dry CH_2Cl_2 (83 mM, 2.4 mL) was added shortly followed by TMDS (1.5 eq., 52.4 µL, 0.30 mmol). The reaction mixture was stirred at room temperature for 15 minutes and then cooled to –78 °C. After 5 minutes at this temperature, a solution of purified mCPBA[3] (1.1 equiv., 38 mg, 0.22 mmol, CAUTION – see comment in section 5) in CH_2Cl_2 (2.4 mL) was added and the vial was removed from the cooling bath and stirred overnight. The reaction mixture was then diluted with EtOAc and sat. aq. $NaHCO_3$ was added and the aqueous layer is extracted with EtOAc (3 x 20 mL). The combined organics were dried with Na_2SO_4, filtered and concentrated under reduced pressure. The crude residue was purified by FCC (pentane:Et_2O or pentane:EtOAc, Et_3N used as an additive where noted).

General Procedure 5: 1,2-Transposition with reductive quench

To a 10 mL Schlenk tube equipped with a magnetic stirring bar was added the amide (0.2 mmol). The vessel evacuated and backfilled with N_2 three times. The N_2 line was then exchanged for a N_2 balloon. A solution of Vaska's catalyst (1.5 mol%, 2.4 mg) and DIPEA (1.2 eq., 41.6 µL, 0.24 mmol) in CH_2Cl_2 (83 mM, 2.4 mL) was added shortly followed by TMDS (1.5 eq., 52.4 µL, 0.30 mmol). The reaction mixture was stirred at room temperature for 15 minutes and then cooled to –78 °C. After 5 minutes at this temperature, a solution of purified mCPBA[3] (1.1 equiv., 38 mg, 0.22 mmol, CAUTION – see comment in section 5) in CH_2Cl_2 (2.4 mL) was added and the reaction mixture was stirred for a further 10 minutes. $LiAlH_4$ (1M in Et_2O, 800 µL, 0.80 mmol, 4 eq.) was added slowly. The reaction mixture was removed from the cooling bath and stirred for 2 hours. Et_2O (2 mL) was added and the reaction mixture was cooled to 0 °C. H_2O (30 µL) was added followed by 10% aq. NaOH (30 µL) and a further portion of H_2O (90 µL). The heterogeneous mixture was stirred for 10 minutes before $MgSO_4$ was added and the mixture stirred for a further 10 minutes. The reaction mixture was filtered and then concentrated under reduced pressure. The crude residue was purified by FCC (pentane:EtOAc with MeOH or Et_3N as additives where noted).

General Procedure 6: Acylative transposition

To a 10 mL vial equipped with a magnetic stirring bar was added the amide (0.2 mmol). The vial was capped with a septum and evacuated and backfilled with N_2 three times. The N_2 line was then exchanged for a N_2 balloon. A solution of Vaska's catalyst (1.5 mol%, 2.4 mg) and DIPEA (1.2 eq., 41.6 µL, 0.24 mmol) in CH_2Cl_2 (83 mM, 2.4 mL) was added shortly followed by TMDS (1.5 eq., 52.4 µL, 0.30 mmol). The reaction mixture was stirred at room temperature for 15 minutes and then cooled to –20 °C. After 5 minutes at this temperature, either acid chloride (0.4 mmol) and DIPEA (1.0 eq., 34.7 µL, 0.20 mmol) or isocyanate (0.4 mmol) (and no further DIPEA) was added and the solution was stirred at room temperature for 30 minutes. Ethanolamine (36 µL, 0.6 mmol) was added and the mixture was stirred vigorously for 5 minutes. Cooled sat. aq. NH_4Cl was added and the aqueous phase was extracted with Et_2O (3 x 20 mL).

The combined organics were dried with Na_2SO_4, filtered and concentrated under reduced pressure. The crude residue was purified by FCC (cold pentane:Et_2O or pentane:EtOAc, with Et_3N used as an additive where noted - typically required for acyclic examples).

5.2 Starting material synthesis

Known compound synthesis:

The following amides were synthesized according to our previous literature reports:

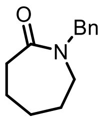

1a

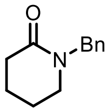

1b

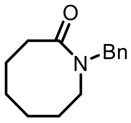

1c

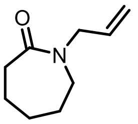

1d

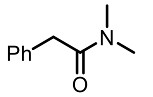

1o

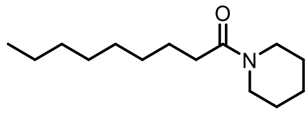

1p

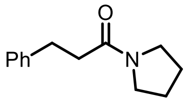

1q

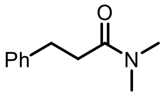

1w

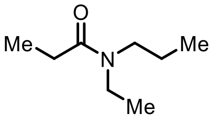

1x

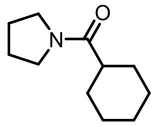

1y

Compound 1e

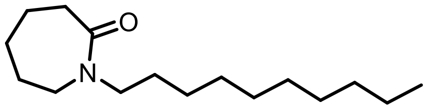

Following General Procedure 2, caprolactam (565 mg, 5.00 mmol) was reacted with 1-bromodecane (1.11 mL, 5.35 mmol) to afford the title compound as colourless oil (1.14 g, 90%) following purification by FCC (pentane:EtOAc 1:1 to 0:1).

IR (film) ν_{max}/cm^{-1} 2927, 2855, 1647

^{1}H NMR (400 MHz, $CDCl_3$) ^{1}H NMR (400 MHz, $CDCl_3$) δ 3.43 – 3.28 (m, 4H), 2.54 – 2.47 (m, 2H), 1.77 – 1.57 (m, 4H), 1.54 – 1.43 (m, 2H), 1.26 (d, J = 9.1 Hz, 16H), 0.93 – 0.82 (m, 3H).

^{13}C NMR (101 MHz, $CDCl_3$) ^{13}C NMR (101 MHz, $CDCl_3$) δ 175.6, 49.7, 48.4, 37.5, 32.0, 30.2, 29.7, 29.7, 29.6, 29.4, 28.9, 28.3, 27.1, 23.7, 22.8, 14.3.

HRMS (ES+) exact mass calculated for $[M+Na]^+$ ($C_{16}H_{31}NONa$) requires m/z 276.2298, found m/z 276.2310

Compound 1f

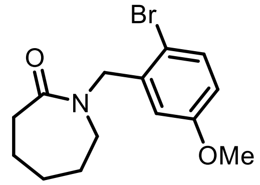

Following General Procedure 1, caprolactam (565 mg, 5.00 mmol) was reacted with 2-bromo-5-methoxybenzyl bromide (1.50 g, 5.35 mmol) to afford the title compound as an off-white solid (1.40 g, 90%) following purification by FCC (Et_2O).

mp 40-42 °C

IR (film) ν_{max}/cm^{-1} 2934, 1648, 1470

^{1}H NMR (400 MHz, $CDCl_3$) δ 7.41 (d, *J* = 8.7 Hz, 1H), 6.81 (d, *J* = 3.1 Hz, 1H), 6.68 (dd, *J* = 8.7, 3.1 Hz, 1H), 4.67 (s, 2H), 3.76 (s, 3H), 3.37 – 3.30 (m, 2H), 2.67 – 2.58 (m, 2H), 1.79 – 1.67 (m, 4H), 1.56 (qd, *J* = 5.7, 3.8 Hz, 2H).

^{13}C NMR (101 MHz, $CDCl_3$) δ 176.3, 159.4, 137.9, 133.5, 115.2, 114.5, 114.0, 55.6, 51.2, 49.5, 37.3, 30.1, 28.3, 23.6.

HRMS (ES+) exact mass calculated for $[M+Na]^+$ ($C_{14}H_{18}BrNO_2Na$) requires m/z 334.0413, found m/z 334.0414

Compound 1g

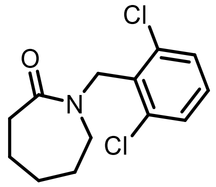

Following General Procedure 1, caprolactam (565 mg, 5.00 mmol) was reacted with 2,6-dichlorobenzyl bromide (1.28 g, 5.35 mmol) to afford the title compound as an off-white solid (0.972 mg, 72%) following purification by FCC (Et_2O).

mp 80-84 °C

IR (film) ν_{max}/cm^{-1} 2933, 1650, 1437

^{1}H NMR (400 MHz, $CDCl_3$) δ 7.32 (dd, *J* = 8.6, 1.0 Hz, 2H), 7.19 (ddt, *J* = 8.6, 7.4, 0.9 Hz, 1H), 4.97 – 4.91 (m, 2H), 3.20 – 3.11 (m, 2H), 2.61 – 2.52 (m, 2H), 1.75 – 1.58 (m, 4H), 1.36 – 1.27 (m, 2H).

^{13}C NMR (101 MHz, $CDCl_3$) δ 175.6, 137.1, 132.8, 129.6, 128.7, 46.9, 44.7, 37.4, 30.1, 28.0, 23.4.

HRMS (ES+) exact mass calculated for $[M+Na]^+$ ($C_{13}H_{15}Cl_2NONa$) requires m/z 294.0423, found m/z 294.0432

Compound 1h

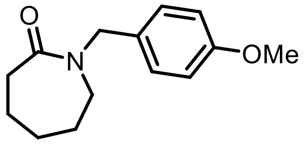

Following General Procedure 2, caprolactam (565 mg, 5.00 mmol) was reacted with 4-methoxybenzyl chloride (0.825 µL, 6.1 mmol) to afford the title compound as a white solid (1.1 g, 96%) following purification by FCC (EtOAc). Data were in agreement with the literature.[2]

^{1}H NMR (400 MHz, $CDCl_3$) δ 7.21 – 7.14 (m, 2H), 6.87 – 6.78 (m, 2H), 4.50 (s, 2H), 3.83 – 3.74 (m, 3H), 3.29 – 3.22 (m, 2H), 2.57 (dt, J = 6.3, 1.9 Hz, 2H), 1.68 (dd, J = 6.2, 3.2 Hz, 4H), 1.46 (q, J = 5.2 Hz, 2H).

^{13}C NMR (101 MHz, $CDCl_3$) δ 176.0, 159.0, 130.2, 129.6, 114.0, 55.4, 50.5, 48.8, 37.3, 30.1, 28.3, 23.5.

Compound 1i

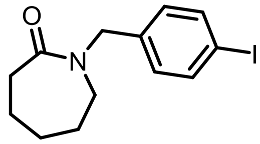

Following General Procedure 1, caprolactam (565 mg, 5.00 mmol) was reacted with 4-iodobenzyl bromide (1.59 g, 5.35 mmol) to afford the title compound as a yellow oil (1.55 g, 94%) following purification by FCC (Et_2O).

IR (film) ν_{max}/cm^{-1} 2931, 1640, 1485

^{1}H NMR (400 MHz, $CDCl_3$) δ 7.67 – 7.58 (m, 2H), 7.06 – 6.96 (m, 2H), 4.51 (s, 2H), 3.30 – 3.23 (m, 2H), 2.63 – 2.53 (m, 2H), 1.69 (dt, J = 7.6, 2.9 Hz, 4H), 1.56 – 1.43 (m, 2H).

^{13}C NMR (101 MHz, $CDCl_3$) δ 176.1, 137.9, 137.7, 130.3, 92.8, 50.9, 49.2, 37.3, 30.1, 28.3, 23.5.

HRMS (ES+) exact mass calculated for $[M+Na]^+$ ($C_{13}H_{16}INONa$) requires m/z 352.0169, found m/z 352.0173

Compound 1j

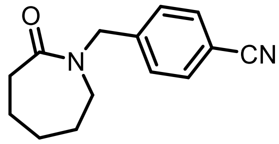

Following General Procedure 1, caprolactam (565 mg, 5.00 mmol) was reacted with 4-cyanobenzyl bromide (1.05 g, 5.35 mmol) to afford the title compound as an off-white solid (0.942 mg, 83%) following purification by FCC (Et_2O).

mp 96-98 °C

IR (film) ν_{max}/cm^{-1} 2934, 2229, 1642

^{1}H NMR (400 MHz, $CDCl_3$) δ 7.64 – 7.55 (m, 2H), 7.36 (dt, *J* = 8.0, 0.7 Hz, 2H), 4.62 (s, 2H), 3.34 – 3.27 (m, 2H), 2.66 – 2.57 (m, 2H), 1.71 (dt, *J* = 5.9, 2.9 Hz, 4H), 1.53 (tt, *J* = 7.0, 2.7 Hz, 2H).
^{13}C NMR (101 MHz, $CDCl_3$) δ 176.3, 143.7, 132.5, 128.7, 118.9, 111.4, 51.3, 49.6, 37.2, 30.0, 28.3, 23.5.
HRMS (ES+) exact mass calculated for $[M+Na]^+$ ($C_{14}H_{16}N_2ONa$) requires m/z 251.1155, found m/z 251.1158

Compound S1-1

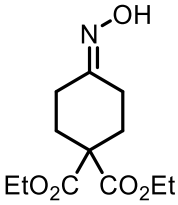

A solution of diethyl 4-oxocyclohexane-1,1-dicarboxylate[4] (3.00 g, 12.4 mmol) and $NH_2OH.HCl$ (1.72 g, 24.7 mmol) in pyridine (12 mL, 1 M) was heated to 60 °C for 30 min. The reaction mixture was cooled to room temperature and H_2O (100 mL) was added. The resulting solution was extracted with EtOAc (3 x 200 mL). The combined organics were washed with sat. aq. $CuSO_4$ (2 x 100 mL) and then H_2O (2 x 100 mL) before being dried with $MgSO_4$, filtered and concentrated under reduced pressure. The crude oil was purified by FCC (silica, 4:1 pentane:EtOAc) to yield the title compound as a white solid (2.87 g, 90%)
mp 46–50 °C
IR (film) ν_{max}/cm^{-1} 3248, 2983, 1731, 1248
^{1}H NMR (400 MHz, $CDCl_3$) δ 4.26 – 4.13 (m, 4H), 2.62 (dd, *J* = 7.9, 5.3 Hz, 2H), 2.31 (dd, *J* = 7.7, 5.0 Hz, 2H), 2.19 (d, *J* = 6.3 Hz, 2H), 2.14 (t, *J* = 6.7 Hz, 2H), 1.25 (tdd, *J* = 6.9, 4.5, 2.3 Hz, 6H).
^{13}C NMR (101 MHz, $CDCl_3$) δ 171.1, 158.2, 61.7, 54.5, 31.1, 29.9, 28.1, 28.1, 20.8, 14.2.
HRMS (ES+) exact mass calculated for $[M+H]^+$ ($C_{12}H_{20}NO_5$) requires m/z 258.1336, found m/z 258.1341

Compound S1-2

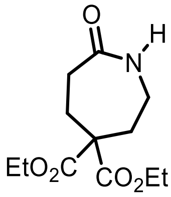

Following a modified literature procedure,[5] to a solution of **S1-1** (4.77 g, 18.6 mmol) in anhydrous dioxane (135 mL, 0.13 M) at 0 °C, $SOCl_2$ (13.5 mL, 186 mmol) was added dropwise. The resulting solution was stirred at 0 °C for 15 minutes and then 1.5 h at room temperature. H_2O (160 mL) was added and the biphasic mixture was extracted with $CHCl_3$ (3 x 150 mL). The combined organics were dried $MgSO_4$, filtered and concentrated under reduced pressure. The resulting orange oil was purified by FCC (silica, 19:1 EtOAc:

MeOH) to yield the title compound as a yellow solid (2.92 g, 61%)

mp 76-80 °C

IR (film) ν_{max}/cm^{-1} 3248, 2982, 1730, 1674, 1236.

^{1}H NMR (400 MHz, $CDCl_3$) δ 6.09 (s, 1H), 4.15 (q, *J* = 7.1 Hz, 4H), 3.26 (td, *J* = 5.9, 3.2 Hz, 2H), 2.48 – 2.39 (m, 2H), 2.23 – 2.18 (m, 2H), 2.15 – 2.10 (m, 2H), 1.19 (t, *J* = 7.1 Hz, 6H);

^{13}C NMR (101 MHz, $CDCl_3$) (101 MHz, $CDCl_3$) δ 177.7, 170.9, 61.9, 57.6, 38.7, 34.6, 31.9, 27.7, 14.2.

HRMS (ES+) exact mass calculated for $[M+H]^+$ ($C_{12}H_{20}NO_5$) requires m/z 258.1336, found m/z 258.1338

Compound 1k

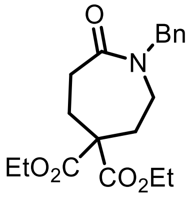

Following General Procedure 1, lactam **S1-2** (1.50 g, 5.80 mmol) was reacted with benzyl bromide (2.08 mL, 17 mmol) and NaH (60% dispersion in mineral oil, 350 mg, 8.8 mmol) to afford the title compound as an off-white solid (1.36 g, 67%) following purification by FCC (pentane:EtOAc 1:1).

mp 46-48 °C

IR (film) ν_{max}/cm^{-1} 2981, 1730, 1652, 1246.

^{1}H NMR (400 MHz, $CDCl_3$)

δ 7.36 – 7.18 (m, 5H), 4.57 (s, 2H), 4.25 – 4.11 (m, 4H), 3.36 (dd, *J* = 6.6, 3.6 Hz, 2H), 2.63 (dt, *J* = 8.5, 1.6 Hz, 2H), 2.30 – 2.23 (m, 2H), 2.03 (d, *J* = 7.7 Hz, 2H), 1.27 – 1.18 (m, 6H).

^{13}C NMR (101 MHz, $CDCl_3$) δ 174.7, 170.9, 137.5, 128.8, 128.3, 127.6, 61.8, 57.1, 51.0, 44.5, 33.2, 32.8, 28.1, 14.1.

HRMS (ES+) exact mass calculated for $[M+H]^+$ ($C_{19}H_{26}NO_5$) requires m/z 348.1806, found m/z 348.1807

Compound S1-3

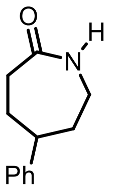

To a solution of 4-phenylcyclohexanone (1.04 g, 6.00 mmol) in EtOH (18 mL), was added $NH_2OH.HCl$ (834 mg, 12 mmol) and NaOAc (984 mg, 12 mmol). The mixture was warmed to 80 °C and stirred for 1 hour. H_2O was added and solution was extracted with EtOAc (3 x 50 mL). The combined organics were dried with Na_2SO_4, filtered and concentrated under reduced pressure. The crude residue was purified by

FCC (2:1 to 1:1 pentane:Et_2O).

1,4-dioxane (30 mL) was added to the resulting oxime followed by dropwise addition of $SOCl_2$ (3 mL) to the mixture at 0 °C. The reaction mixture was allowed to come naturally to room temperature and stirred for a further hour. H_2O was added and the biphasic mixture was extracted with EtOAc (3 x 50 mL). The combined organics were dried with Na_2SO_4, filtered and concentrated under reduced pressure. The crude reside was purified by FCC (19:1 CH_2Cl_2:MeOH) to afford the title compound as an orange solid (550 mg, 48%). Data were in agreement with the literature.[6]

^{1}H NMR (400 MHz, $CDCl_3$) δ 7.40 – 7.26 (m, 2H), 7.24 – 7.15 (m, 3H), 6.60 (s, 1H), 3.46 – 3.24 (m, 2H), 2.77 (tt, *J* = 12.1, 3.5 Hz, 1H), 2.68 – 2.53 (m, 2H), 2.01 (dddd, *J* = 15.7, 7.2, 3.2, 1.5 Hz, 2H), 1.87 – 1.67 (m, 2H).

^{13}C NMR (101 MHz, $CDCl_3$) δ 178.9, 146.4, 128.8, 126.9, 126.7, 49.0, 42.3, 37.5, 35.9, 30.6.

Compound 1l

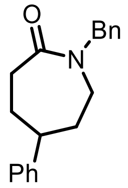

Following General Procedure 1, lactam **S1-3** (540 mg, 2.86 mmol) was reacted with benzyl bromide (363 μL, 3.06 mmol) to afford the title compound as an off-white solid (413 mg, 52%) following purification by FCC (Et_2O).

IR (film) ν_{max}/cm^{-1} 1644, 1495, 1452.

^{1}H NMR (400 MHz, $CDCl_3$) δ 7.38 – 7.23 (m, 7H), 7.23 – 7.17 (m, 1H), 7.14 – 7.10 (m, 2H), 4.82 (d, *J* = 14.6 Hz, 1H), 4.48 (d, *J* = 14.6 Hz, 1H), 3.55 (dd, *J* = 15.4, 10.9 Hz, 1H), 3.30 (ddd, *J* = 15.4, 6.4, 1.7 Hz, 1H), 2.82 – 2.66 (m, 3H), 2.04 (dtdd, *J* = 13.0, 4.7, 3.3, 1.7 Hz, 1H), 1.93 – 1.72 (m, 2H), 1.52 (dddd, *J* = 14.2, 12.5, 11.0, 1.7 Hz, 1H).

^{13}C NMR (101 MHz, $CDCl_3$) δ 175.5, 146.2, 137.9, 128.7, 128.3, 127.6, 126.8, 126.7, 51.2, 48.4, 48.2, 36.6, 35.8, 30.9.

HRMS (ES+) exact mass calculated for $[M+H]^+$ ($C_{19}H_{22}NO$) requires m/z 280.1696, found m/z 280.1704

Compound 1m

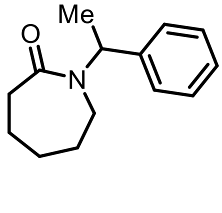

Following General Procedure 1, caprolactam (340 mg, 3.00 mmol) was reacted with (1-bromo)ethylbenzene (440 µL, 3.2 mmol, 1.1 eq.) for 24 hours before addition of a further portion of electrophile (1.1 eq.) and the reaction was left for an additional 16 hours. The title compound was afforded as a yellow oil (103 mg, 16%) following purification by FCC (pentane:EtOAc 3:7).

IR (film) ν_{max}/cm^{-1} 2930, 1638

^{1}H NMR (400 MHz, $CDCl_3$ δ 7.38 – 7.20 (m, 5H), 6.05 (q, *J* = 6.8 Hz, 1H), 3.09 – 3.02 (m, 2H), 2.60 (t, *J* = 5.2 Hz, 2H), 1.82 – 1.52 (m, 4H), 1.48 (d, *J* = 6.9 Hz, 3H), 1.36 (d, *J* = 6.1 Hz, 2H).

^{13}C NMR (101 MHz, $CDCl_3$) δ 175.9, 141.1, 128.5, 127.6, 127.3, 50.8, 43.9, 37.9, 30.1, 29.3, 23.7, 16.4.

HRMS (ES+) exact mass calculated for $[M+H]^+$ ($C_{14}H_{20}NO$) requires m/z 218.1539, found m/z 218.1581

Compound 1n

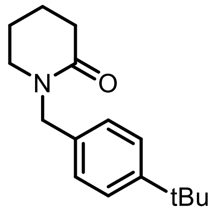

Following General Procedure 2, valerolactam (496 mg, 5.00 mmol) was reacted with 4-tertbutylbenzyl chloride (1.03 mL, 5.35 mmol) to afford the title compound as an off-white solid (938 mg, 76%) following purification by FCC (pentane:EtOAc 1:1 to 0:1).

mp 70-72 °C

IR (film) ν_{max}/cm^{-1} 2957, 1642

^{1}H NMR (400 MHz, $CDCl_3$) δ 7.37 – 7.29 (m, 2H), 7.22 – 7.13 (m, 2H), 4.56 (s, 2H), 3.20 (t, *J* = 5.5 Hz, 2H), 2.46 (dd, *J* = 6.8, 5.7 Hz, 2H), 1.86 – 1.71 (m, 4H), 1.30 (s, 9H).

^{13}C NMR (101 MHz, $CDCl_3$) δ 169.9, 150.3, 134.4, 127.9, 125.6, 49.9, 47.4, 34.6, 32.6, 31.5, 23.3, 21.6.

HRMS (ES+) exact mass calculated for $[M+Na]^+$ ($C_{16}H_{23}NONa$) requires m/z 268.1672, found m/z 268.1679

Compound 1r

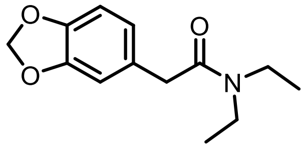

Following General Procedure 3, homopiperonylic acid (450 mg, 2.5 mmol) was reacted with diethylamine (310 µL, 3.0 mmol) to afford the title compound as a colorless oil (526 mg, 90%) following purification by FCC (Et_2O).

IR (film) ν_{max}/cm^{-1} 1638, 1491, 1245, 1039

^{1}H NMR (400 MHz, $CDCl_3$) δ 6.79 – 6.72 (m, 2H), 6.67 (ddt, J = 7.9, 1.6, 0.7 Hz, 1H), 5.92 (s, 2H), 3.59 (s, 2H), 3.37 (q, J = 7.1 Hz, 2H), 3.30 (q, J = 7.1 Hz, 2H), 1.11 (td, J = 7.1, 2.2 Hz, 6H).

^{13}C NMR (101 MHz, $CDCl_3$) δ 170.2, 147.9, 146.5, 129.3, 121.8, 109.4, 108.4, 101.1, 42.4, 40.5, 40.3, 14.4, 13.1.

HRMS (ES+) exact mass calculated for $[M+H]^+$ ($C_{13}H_{18}NO_3$) requires m/z 236.1281, found m/z 236.1291

Compound 1s

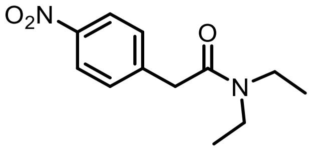

Following General Procedure 3, 4-nitrophenylacetic acid (453 mg, 2.5 mmol) was reacted with diethylamine (310 µL, 3.0 mmol) to afford the title compound as a colorless oil (479 mg, 81%) following purification by FCC (Et_2O). All data were in agreement with the literature.[7]

^{1}H NMR (400 MHz, $CDCl_3$) δ 8.22 – 8.13 (m, 2H), 7.47 – 7.39 (m, 2H), 3.78 (s, 2H), 3.40 (q, J = 7.1 Hz, 2H), 3.33 (q, J = 7.1 Hz, 2H), 1.15 (dt, J = 16.9, 7.2 Hz, 6H).

^{13}C NMR (101 MHz, $CDCl_3$) δ 168.7, 147.1, 143.3, 130.1, 123.8, 42.6, 40.6, 40.3, 14.5, 13.1.

Compound 1t

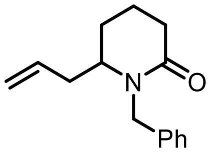

Following a literature procedure[8], to a solution of N-Bn glutarimide (500 mg, 2.46 mmol) in THF (12.3 mL), cooled to –78 °C, was added $LiBEt_3H$ (1M in THF, 3.70 mL, 3.70 mmol). The solution was stirred for 30 minutes and sat. aq. $NaHCO_3$ was added and the mixture warmed to 0 °C. H_2O_2 (30% aq., 1.85 mL) was added and the mixture was stirred for a further 30 minutes before warming to room temperature. The volatiles were removed and CH_2Cl_2 (20 mL) was added. The layers were separated and the aqueous phase was extracted with CH_2Cl_2 (3 x 20 mL). The combined organics were dried with Na_2SO_4, filtered and concentrated under reduced pressure. CH_2Cl_2 (9.5 mL) was added, followed by allyltrimethylsilane (780 µL, 4.92 mmol). The solution was cooled to –78 °C and $BF_3.Et_2O$ (610 µL, 4.92 mmol) was added in a dropwise fashion. The solution was stirred at this temperature for a further 30 minutes and then stirred overnight at room temperature. The reaction mixture was cooled to 0 °C and sat. aq. $NaHCO_3$ was added. The resulting biphasic mixture was extracted with CH_2Cl_2 (3 x 25 mL) and the combined organics were dried with Na_2SO_4, filtered and concentrated under reduced pressure. The crude residue was purified by FCC (pentane:Et_2O 1:2 to 0:1) to yield the title compound as a colorless oil (358 mg, 64%). Data were in

agreement with the literature.[8]

^{1}H NMR (400 MHz, $CDCl_3$) δ 7.35 – 7.29 (m, 2H), 7.28 – 7.18 (m, 3H), 5.71 – 5.56 (m, 1H), 5.41 (d, *J* = 15.2 Hz, 1H), 5.13 – 5.03 (m, 2H), 3.98 (d, *J* = 15.2 Hz, 1H), 3.35 (dq, *J* = 9.1, 4.4 Hz, 1H), 2.54 – 2.40 (m, 3H), 2.27 (dddt, *J* = 14.2, 9.9, 7.9, 1.1 Hz, 1H), 1.98 – 1.63 (m, 4H).

^{13}C NMR (101 MHz, $CDCl_3$) δ 170.4, 137.6, 134.1, 128.6, 127.8, 127.2, 118.2, 54.9, 47.4, 36.9, 32.0, 26.2, 17.2.

Compound 1u

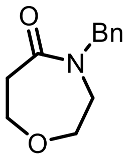

Following General Procedure 1, oxazepane (576 mg, 5.00 mmol) was reacted with benzyl bromide (640 µL, 5.35 mmol) to afford the title compound as a light-yellow oil (796 mg, 78%) following purification by FCC (pentane:EtOAc 1:1). Data were in agreement with the literature.[9]

^{1}H NMR (400 MHz, $CDCl_3$) δ 7.37 – 7.21 (m, 5H), 4.60 (s, 2H), 3.84 – 3.75 (m, 2H), 3.61 – 3.51 (m, 2H), 3.43 – 3.37 (m, 2H), 2.86 – 2.78 (m, 2H).

^{13}C NMR (101 MHz, $CDCl_3$) δ 174.6, 137.4, 128.9, 128.4, 127.7, 70.4, 65.6, 51.6, 50.9, 41.3.

Compound 1v

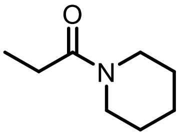

To a solution of piperidine (1.00 mL, 10.1 mmol) in CH_2Cl_2 (0.1 M), cooled to 0 °C, was added propanoyl chloride (648 µL, 7.5 mmol). The reaction mixture was allowed to come naturally to room temperature over ~1 hour and then stirred at room temperature for a further 4 hours. Sat. aq. $NaHCO_3$ (25 mL) was added and the resulting biphasic mixture was extracted with CH_2Cl_2 (3 x 50 mL). The combined organics were dried with Na_2SO_4, filtered and concentrated under reduced pressure. The crude residue was purified by FCC (Et_2O) to afford the title compound as a colorless oil (649 mg, 61%). Data were in agreement with the literature.[10]

^{1}H NMR (400 MHz, $CDCl_3$) δ 3.53 (t, *J* = 5.7 Hz, 2H), 3.37 (t, *J* = 5.4 Hz, 2H), 2.37 – 2.26 (m, 2H), 1.65 – 1.57 (m, 2H), 1.52 (d, *J* = 5.7 Hz, 4H), 1.17 – 1.05 (m, 3H).

^{13}C NMR (101 MHz, $CDCl_3$) δ 172.2, 46.6, 42.7, 26.7, 26.6, 25.7, 24.7, 9.7.

Compound 1x

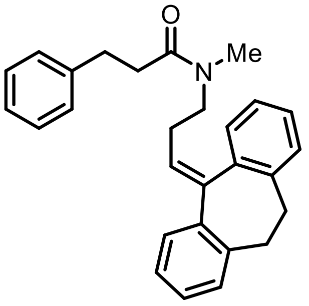

To a solution of nortriptyline hydrochloride (1.50 g, 5.0 mmol) in CH_2Cl_2 (50 mL), cooled to 0 °C, was added DIPEA (1.31 mL, 7.5 mmol) and 3-phenylpropanoyl chloride (1.11 mL, 7.5 mmol). The solution was warmed to room temperature and stirred for 2 hours. Sat. aq. NH_4Cl (30 mL) was added and the layers were separated. The aqueous phase was extracted with CH_2Cl_2 (2 x 50 mL) and the combined organics were dried with Na_2SO_4, filtered and the solvent was removed under reduced pressure. The crude residue was purified by FCC (1:1 to 0:1 pentane:Et_2O) to yield the title compound as a yellow oil (1.97 g, 99%).

IR (film) ν_{max}/cm^{-1} 2925, 1647, 1486

^{1}H NMR (400 MHz, $CDCl_3$, mixture of rotamers) δ 7.30 – 7.01 (m, 13H), 5.83 (t, *J* = 7.4 Hz, 0.5H), 5.77 (t, *J* = 7.7 Hz, 0.5H), 3.46 (td, *J* = 7.2, 3.1 Hz, 1H), 3.41 – 3.18 (m, 3H), 2.90 (ddd, *J* = 26.6, 9.2, 7.2 Hz, 3H), 2.81 – 2.68 (m, 4H), 2.60 – 2.51 (m, 1H), 2.44 (td, *J* = 7.3, 1.6 Hz, 1H), 2.34 (m, 2H).

^{13}C NMR (101 MHz, $CDCl_3$, mixture of rotamers) δ 172.0, 172.0, 146.1, 144.5, 141.6, 141.6, 141.1, 140.7, 139.97, 139.6, 139.5, 139.4, 137.1, 137.1, 130.3, 130.1, 128.8, 128.6, 128.5, 128.5, 128.3, 128.3, 128.2, 128.0, 127.9, 127.6, 127.5, 127.3, 126.5, 126.2, 126.2, 126.0, 125.9, 49.5, 47.5, 35.7, 35.3, 35.0, 33.9, 33.9, 33.6, 32.1, 32.0, 31.6, 31.4, 28.6, 27.7.

HRMS (ES+) exact mass calculated for $[M+Na]^+$ ($C_{28}H_{29}NONa$) requires m/z 418.2141, found m/z 418.2147

Compound S1-4

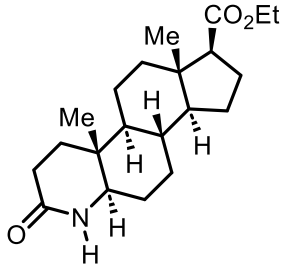

To a solution of 3-oxo-4-aza-5α-androstan-17β-carboxylic acid (400 mg, 1.25 mmol) in DMF (7.4 mL) was added K_2CO_3 (1.04 g, 7.5 mmol) and ethyl iodide (510 µL, 6.3 mmol). The resulting suspension was stirred vigorously overnight before H_2O (20 mL) and EtOAc (20 mL) were added.

The layers were separated and the aqueous phase was further extracted with EtOAc (2 x 20 mL). The combined organics were washed with brine (20 mL), dried with Na_2SO_4, filtered and concentrated under reduced pressure. The crude residue was purified by FCC (1:0 to 19:1 EtOAc:MeOH) to yield the title compound as a white solid (401 mg, 92%).

IR (film) ν_{max}/cm^{-1} 3192, 2934, 1731, 1683

^{1}H NMR (400 MHz, $CDCl_3$) δ 5.80 (s, 1H), 4.24 – 4.02 (m, 2H), 3.05 (dd, *J* = 12.0, 4.1 Hz, 1H), 2.44 – 2.37 (m, 2H), 2.32 (t, *J* = 9.4 Hz, 1H), 2.13 (dddd, *J* = 13.8, 11.5, 9.0, 2.8 Hz, 1H), 2.02 (dt, *J* = 12.4, 2.9 Hz, 1H), 1.91 – 1.83 (m, 1H), 1.83 – 1.63 (m, 3H), 1.62 – 1.47 (m, 2H), 1.47 – 1.31 (m, 4H), 1.26 (q, *J* = 6.7 Hz, 5H), 1.17 – 0.95 (m, 2H), 0.90 (s, 3H), 0.80 (ddd, *J* = 12.0, 10.4, 4.0 Hz, 1H), 0.67 (s, 3H).

^{13}C NMR (101 MHz, $CDCl_3$) δ 174.0, 172.3, 60.9, 60.1, 55.6, 55.3, 51.4, 44.3, 38.4, 35.9, 35.3, 33.5, 29.7, 28.7, 27.5, 24.5, 23.6, 21.2, 14.6, 13.7, 11.5.

HRMS (ES+) exact mass calculated for $[M+H]^+$ ($C_{21}H_{34}NO_3$) requires m/z 348.2533, found m/z 348.2534

Compound 1y

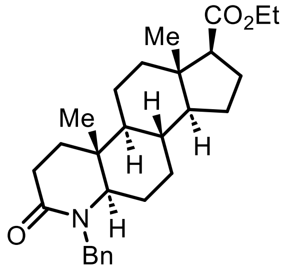

Following General Procedure 1, Compound S1-4 (350 mg, 1.0 mmol) was reacted afford the title compound as a white solid (176 mg, 40%) following purification by FCC (2:1 to 0:1 pentane:EtOAc).

IR (film) ν_{max}/cm^{-1} 2949, 1731, 1645

^{1}H NMR (400 MHz, $CDCl_3$) δ 7.31 – 7.25 (m, 2H), 7.23 – 7.19 (m, 1H), 7.19 – 7.14 (m, 2H), 5.02 (d, *J* = 15.8 Hz, 1H), 4.47 (d, *J* = 15.8 Hz, 1H), 4.16 (dq, *J* = 10.8, 7.1 Hz, 1H), 4.07 (dq, *J* = 10.9, 7.1 Hz, 1H), 3.10 (dd, *J* = 12.5, 3.5 Hz, 1H), 2.65 – 2.54 (m, 2H), 2.29 (t, *J* = 9.4 Hz, 1H), 2.10 (dddd, *J* = 14.1, 11.7, 9.1, 2.9 Hz, 1H), 2.04 – 1.96 (m, 1H), 1.88 (tdd, *J* = 16.6, 6.5, 3.7 Hz, 2H), 1.81 – 1.72 (m, 1H), 1.72 – 1.56 (m, 3H), 1.49 – 1.38 (m, 1H), 1.38 – 1.14 (m, 8H), 1.01 (ddd, *J* = 12.5, 10.7, 7.1 Hz, 1H), 0.93 (s, 3H), 0.85 – 0.72 (m, 2H), 0.64 (s, 3H).

^{13}C NMR (101 MHz, $CDCl_3$) δ 174.0, 171.3, 138.8, 128.6, 126.9, 126.8, 65.1, 60.1, 55.5, 55.3, 52.0, 45.4, 44.2, 38.4, 36.6, 34.6, 33.1, 30.3, 29.3, 25.6, 24.4, 23.6, 21.1, 14.6, 13.6, 12.6.

HRMS (ES+) exact mass calculated for $[M+H]^+$ ($C_{28}H_{40}NO_3$) requires m/z 438.3003, found m/z 438.3009

MP 136-138 °C

5.3 Aminoketone synthesis

Compound 3a

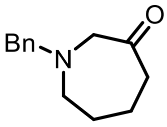

Following General Procedure 4, lactam **1a** (40.6 mg, 0.20 mmol) was reacted to afford the title compound as a colorless oil (25.5 mg, 63%) following purification by FCC (8:1 pentane:EtOAc).

IR (film) ν_{max}/cm^{-1} 2932, 1709.

^{1}H NMR (400 MHz, $CDCl_3$) δ 7.32 – 7.13 (m, 5H), 3.64 (s, 2H), 3.18 (s, 2H), 2.65 (dddd, *J* = 10.1, 7.2, 4.4, 2.2 Hz, 4H), 1.74 – 1.60 (m, 4H).

^{13}C NMR (101 MHz, $CDCl_3$) 214.5, 138.9, 128.7, 128.4, 127.3, 66.8, 62.1, 57.1, 42.7, 29.8, 23.7.

HRMS (ES+) exact mass calculated for $[M+Na]^+$ ($C_{13}H_{17}NONa$) requires m/z 226.1202, found m/z 226.1195

This procedure could be up-scaled following minor modifications:

To a 100 mL Schlenk flask equipped with a magnetic stirring bar was added the amide (1.0 g, 5.0 mmol) and Vaska's complex (14.6 mg, 0.019 mmol, 0.38 mol%). The flask was evacuated and back-filled with N_2 three times and then sealed and a N_2 balloon was fitted. Dry, degassed CH_2Cl_2 (15 mL) was added followed by DIPEA (1.05 mL, 6.0 mmol) then TMDS (1.33 mL, 7.5 mmol). The reaction mixture was stirred at room temperature for 15 minutes before adding CH_2Cl_2 (45 mL) and then cooling to –78 °C. After 5 minutes at this temperature, a solution of purified mCPBA[3] (950 mg, 5.5 mmol, CAUTION – see comment in section 5) in CH_2Cl_2 (60 mL) was added and the mixture was stirred for 20 minutes. The –78 °C bath was replaced with a water bath and the reaction was stirred overnight at room temperature. The reaction mixture was then diluted with EtOAc and sat. aq. $NaHCO_3$ was added and the aqueous layer is extracted with EtOAc (3 x 20 mL). The combined organics were dried with Na_2SO_4, filtered and concentrated under reduced pressure. The crude residue was purified by FCC (pentane:Et_2O 9:1 to 3:1) to yield the title compound as a colorless oil (608 mg, 60%).

Compound 3b

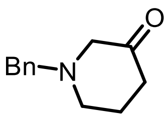

Following a modification to General Procedure 4, lactam **1b** (37.9 mg, 0.20 mmol) was reacted in the usual manner however, following stirring with mCPBA at –78 °C for 10 minutes, pH 5 aq. buffer (NaOAc/AcOH,

2.4 mL) was added and the reaction mixture was stirred for 2 hours at room temperature. Following the standard work-up procedure and purification by FCC (5:1 pentane:EtOAc), the title compound was afforded as a colorless oil (20.7 mg, 55%). Data were in agreement with the literature.[11]

^{1}H NMR (400 MHz, $CDCl_3$) δ 7.24 – 7.06 (m, 5H), 3.44 (s, 2H), 2.87 (s, 2H), 2.55 – 2.46 (m, 2H), 2.26 – 2.18 (m, 2H), 1.86 – 1.75 (m, 2H).

^{13}C NMR (101 MHz, $CDCl_3$) δ 207.2, 137.4, 129.1, 128.5, 127.5, 64.7, 62.7, 51.7, 38.9, 24.1.

Compound 3c

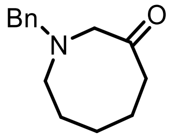

Following General Procedure 4, lactam **1c** (43.5 mg, 0.20 mmol) was reacted to afford the title compound as a colorless oil (28.1 mg, 65%) following purification by FCC (20:1 pentane:EtOAc) then PTLC (15:1 hexane:EtOAc).

IR (film) ν_{max}/cm^{-1} 2928, 1708

^{1}H NMR (400 MHz, $CDCl_3$) δ 7.43 – 7.24 (m, 5H), 3.65 (s, 2H), 2.98 (s, 2H), 2.66 – 2.60 (m, 2H), 2.56 (t, *J* = 6.2 Hz, 2H), 1.80 – 1.72 (m, 2H), 1.68 (ddt, *J* = 9.2, 4.5, 3.0 Hz, 2H), 1.44 – 1.33 (m, 2H).

^{13}C NMR (101 MHz, $CDCl_3$) δ 218.5, 139.1, 129.2, 128.6, 127.5, 66.1, 63.5, 58.7, 40.0, 30.3, 26.8, 26.2.

HRMS (ES+) exact mass calculated for $[M+Na]^+$ ($C_{14}H_{19}NONa$) requires m/z 240.1359, found m/z 240.1357

Compound 3d

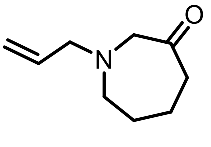

Following General Procedure 4, lactam **1d** (30.6 mg, 0.20 mmol) was reacted to afford the title compound as a colorless oil (13.9 mg, 47%) following purification by FCC (pentane:acetone 9:1).

IR (film) ν_{max}/cm^{-1} 2932, 1709.

^{1}H NMR (400 MHz, $CDCl_3$) δ 5.82 (ddt, *J* = 16.7, 10.2, 6.3 Hz, 1H), 5.23 – 5.11 (m, 2H), 3.23 (s, 2H), 3.16 (dt, *J* = 6.3, 1.4 Hz, 2H), 2.73 – 2.60 (m, 4H), 1.80 – 1.63 (m, 4H).

^{13}C NMR (101 MHz, $CDCl_3$) δ 214.5, 135.5, 118.0, 66.8, 61.1, 57.3, 42.9, 30.1, 23.9.

HRMS (ES+) exact mass calculated for $[M+H]^+$ ($C_9H_{16}NO$) requires m/z 154.1226, found m/z 154.1224

Compound 3e

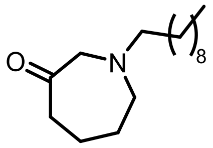

Following General Procedure 4, lactam **1e** (50.6 mg, 0.20 mmol) was reacted to afford the title compound as a colorless oil (21.8 mg, 43%) following purification by FCC (pentane: Et_2O 3:1).

IR (film) ν_{max}/cm^{-1} 2927, 1711

^{1}H NMR (400 MHz, $CDCl_3$) δ 3.24 (s, 2H), 2.72 – 2.65 (m, 2H), 2.65 – 2.60 (m, 2H), 2.55 – 2.47 (m, 2H), 1.79 – 1.60 (m, 4H), 1.43 (dt, *J* = 9.8, 6.7 Hz, 2H), 1.33 – 1.20 (m, 14H), 0.91 – 0.83 (m, 3H).

^{13}C NMR (101 MHz, $CDCl_3$) δ 215.1, 67.1, 57.5, 57.4, 43.0, 32.0, 29.9, 29.8, 29.7, 29.7, 29.5, 27.7, 27.4, 23.9, 22.8, 14.3.

HRMS (ES+) exact mass calculated for $[M+H]^+$ ($C_{16}H_{32}NO$) requires m/z 254.2478, found m/z 254.2486

Compound 3f

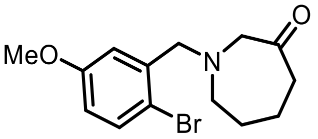

Following a modification to General Procedure 4, lactam **1f** (62.2 mg, 0.20 mmol) was allowed to react for 45 minutes before cooling to –78 °C and allowed to react for 24 hours following mCPBA addition. The title compound was afforded as a colorless oil (37.1 mg, 60%) following purification by FCC (pentane:acetone 10:1 to 6:1).

IR (film) ν_{max}/cm^{-1} 2935, 1707, 1473

^{1}H NMR (400 MHz, $CDCl_3$) δ 7.42 (d, *J* = 8.7 Hz, 1H), 7.08 – 7.02 (m, 1H), 6.69 (dd, *J* = 8.7, 3.1 Hz, 1H), 3.80 (s, 3H), 3.73 (s, 2H), 3.30 (s, 2H), 2.80 – 2.69 (m, 4H), 1.82 – 1.67 (m, 4H).

^{13}C NMR (101 MHz, $CDCl_3$) δ 214.5, 159.1, 139.1, 133.6, 116.4, 114.9, 114.3, 66.8, 61.3, 57.3, 55.6, 42.9, 29.8, 23.7.

HRMS (ES+) exact mass calculated for $[M+H]^+$ ($C_{14}H_{19}BrNO_2$) requires m/z 312.0594, found m/z 312.0595

Compound 3g

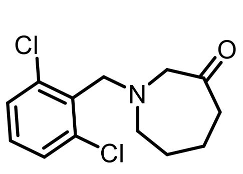

Following a modification to General Procedure 4, lactam **1g** (54.0 mg, 0.20 mmol) was allowed to react

for 1 hour before cooling to –78 °C and allowed to react for 24 hours following mCPBA addition. The title compound was afforded as a colorless oil (26.8 mg, 50%) following purification by FCC (pentane: Et_2O 5:1 to 1:1).

IR (film) ν_{max}/cm^{-1} 2932, 1710, 1436, 765

^{1}H NMR (400 MHz, $CDCl_3$) δ 7.33 – 7.29 (m, 2H), 7.15 (dd, *J* = 8.5, 7.5 Hz, 1H), 3.96 (s, 2H), 3.30 (s, 2H), 2.82 (dd, *J* = 6.1, 4.3 Hz, 2H), 2.70 – 2.61 (m, 2H), 1.77 – 1.62 (m, 4H).

^{13}C NMR (101 MHz, $CDCl_3$) δ 215.3, 137.0, 134.7, 129.2, 128.6, 65.6, 56.6, 55.0, 42.6, 29.3, 23.6.

HRMS (ES+) exact mass calculated for $[M+H]^+$ ($C_{13}H_{16}Cl_2NO$) requires m/z 272.0604, found m/z 272.0610

Compound 3h

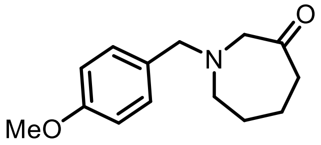

Following General Procedure 4, lactam **1h** (46.4 mg, 0.20 mmol) was reacted to afford the title compound as a colorless oil (27.9 mg, 60%) following purification by FCC (pentane: Et_2O 4:1 to 3:1).

IR (film) ν_{max}/cm^{-1} 2934, 1707, 1512, 1247

^{1}H NMR (400 MHz, $CDCl_3$) δ 7.29 – 7.21 (m, 2H), 6.90 – 6.82 (m, 2H), 3.80 (s, 3H), 3.63 (s, 2H), 3.22 (s, 2H), 2.69 (dt, *J* = 10.1, 3.8 Hz, 4H), 1.78 – 1.65 (m, 4H).

^{13}C NMR (101 MHz, $CDCl_3$) 214.7, 159.0, 131.0, 130.0, 113.9, 66.8, 61.6, 57.0, 55.4, 42.8, 30.0, 23.8.

HRMS (ES+) exact mass calculated for $[M+H]^+$ ($C_{14}H_{20}NO_2$) requires m/z 234.1489, found m/z 234.1484

Compound 3i

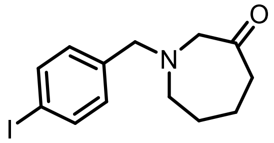

Following a modification to General Procedure 4, lactam **1i** (65.8 mg, 0.20 mmol) was allowed to react for 45 minutes before cooling to –78 °C and allowed to react for 20 hours following mCPBA addition. The title compound was afforded as a colorless oil (37.2 mg, 57%) following purification by FCC (pentane: Et_2O 4:1 to 3:1).

IR (film) ν_{max}/cm^{-1} 2931, 1707

^{1}H NMR (400 MHz, $CDCl_3$) δ 7.69 – 7.61 (m, 2H), 7.16 – 7.05 (m, 2H), 3.63 (s, 2H), 3.23 (s, 2H), 2.76 – 2.63 (m, 4H), 1.72 (dq, *J* = 6.0, 2.6 Hz, 4H).

^{13}C NMR (101 MHz, $CDCl_3$) δ 214.1, 138.8, 137.6, 130.7, 92.7, 67.0, 61.6, 57.2, 42.8, 29.9, 23.8.

HRMS (ES+) exact mass calculated for $[M+Na]^+$ ($C_{13}H_{16}INONa$) requires m/z 352.0169, found m/z 352.0163

Compound 3j

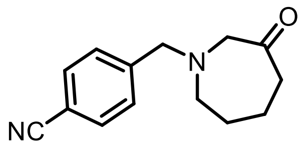

Following a modification to General Procedure 4, lactam **1j** (45.6 mg, 0.20 mmol) was allowed to react for 2 hours before cooling to –78 °C and allowed to react for 26 hours following mCPBA addition. The title compound was afforded as a low melting-point off-white solid (21.1 mg, 46%) following purification by FCC (pentane:EtOAc 1:0 to 3:1 to 3:2).

IR (film) ν_{max}/cm^{-1} 2936, 2228, 1708

^{1}H NMR (400 MHz, $CDCl_3$) δ 7.67 – 7.54 (m, 2H), 7.52 – 7.42 (m, 2H), 3.75 (s, 2H), 3.25 (s, 2H), 2.77 – 2.65 (m, 4H), 1.80 – 1.67 (m, 4H).

^{13}C NMR (101 MHz, $CDCl_3$) δ 213.6, 144.8, 132.4, 129.2, 1190, 111.3, 67.1, 61.7, 57.4, 42.9, 29.9, 23.69.

HRMS (ES+) exact mass calculated for $[M+H]^+$ ($C_{14}H_{17}N_2O$) requires m/z 229.1335, found m/z 229.1342

Compound 3k

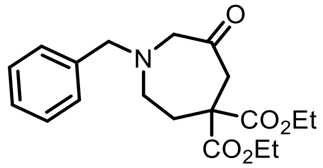

Following a modification to General Procedure 4, lactam **1k** (69.4 mg, 0.20 mmol) was allowed to react for 3 hours before cooling to –78 °C and allowed to react for 24 hours following mCPBA addition. The title compound was afforded as a colorless oil (23.3 mg, 34%) following purification by FCC (pentane:EtOAc 5:1 to 3:1).

IR (film) ν_{max}/cm^{-1} 1732, 1244

^{1}H NMR (400 MHz, $CDCl_3$) δ 7.35 – 7.25 (m, 5H), 4.20 (qd, J = 7.2, 1.3 Hz, 4H), 3.65 (s, 2H), 3.26 (s, 2H), 3.19 (s, 2H), 2.79 (t, J = 6.0 Hz, 2H), 2.24 (dd, J = 6.7, 5.3 Hz, 2H), 1.24 (t, J = 7.1 Hz, 6H).

^{13}C NMR (101 MHz, $CDCl_3$) δ 210.2, 170.8, 138.3, 128.9, 128.6, 127.6, 65.8, 62.1, 61.8, 54.2, 52.4, 46.7, 34.0, 14.1.

HRMS (ES+) exact mass calculated for $[M+H]^+$ ($C_{19}H_{26}NO_5$) requires m/z 348.1806, found m/z 348.1808

Compound 3l

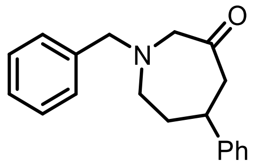

Following General Procedure 4, lactam **1l** (55.8 mg, 0.20 mmol) was reacted to afford the title compound as a colorless oil (20.2 mg, 36%) following purification by FCC (pentane:Et_2O 4:1 to 3:1).

IR (film) ν_{max}/cm^{-1} 1707

^{1}H NMR (400 MHz, $CDCl_3$) δ 7.42 – 7.19 (m, 10H), 3.80 – 3.69 (m, 2H), 3.46 – 3.33 (m, 2H), 3.21 (d, *J* = 18.2 Hz, 1H), 3.00 (dtd, *J* = 13.4, 4.2, 2.0 Hz, 1H), 2.91 (tt, *J* = 10.8, 3.6 Hz, 1H), 2.74 (ddd, *J* = 11.6, 3.7, 1.3 Hz, 1H), 2.54 (ddd, *J* = 13.6, 11.0, 2.9 Hz, 1H), 2.08 – 1.93 (m, 1H), 1.88 (dtdd, *J* = 10.9, 4.4, 3.1, 1.2 Hz, 1H).

^{13}C NMR (101 MHz, $CDCl_3$) δ 213.0, 146.5, 138.7, 128.9, 128.8, 128.6, 127.5, 126.8, 126.6, 66.9, 62.6, 55.8, 49.4, 41.9, 38.5.

HRMS (ES+) exact mass calculated for $[M+Na]^+$ ($C_{19}H_{21}NONa$) requires m/z 302.1515, found m/z 302.1514

Compound 3m

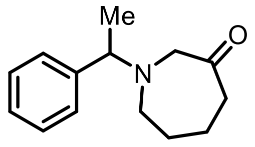

Following General Procedure 4, lactam **1m** (29.6 mg, 0.14 mmol) was reacted, with increased catalyst and TMDS loading to 2 mol% and 1.8 eq. respectively, to afford the title compound as a colorless oil (12 mg, 39%) following purification by FCC (pentane:EtOAc 9:1).

IR (film) ν_{max}/cm^{-1} 1708

^{1}H NMR (400 MHz, $CDCl_3$) ^{1}H NMR (400 MHz, $CDCl_3$) δ 7.43 – 7.18 (m, 5H), 3.85 (q, *J* = 6.7 Hz, 1H), 3.23 (s, 2H), 2.80 – 2.58 (m, 4H), 1.68 (dqd, *J* = 8.5, 4.3, 2.7 Hz, 4H), 1.37 (d, *J* = 6.7 Hz, 3H).

^{13}C NMR (101 MHz, $CDCl_3$) δ 215.3, 143.7, 128.4, 127.6, 127.2, 64.1, 63.3, 54.3, 42.6, 30.4, 24.1, 17.3.

HRMS (ES+) exact mass calculated for $[M+H]^+$ ($C_{14}H_{20}NO$) requires m/z 218.1539, found m/z 218.1541

Compound 3n

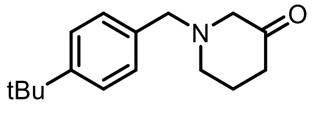

Following a modification to General Procedure 4, lactam **1n** (49.0 mg, 0.20 mmol) was reacted in the usual

manner however, following stirring with mCPBA at –78 °C for 10 minutes, pH 5 aq. buffer (NaOAc/AcOH, 2.4 mL) was added and the reaction mixture was stirred for 2 hours at room temperature. Following the standard work-up procedure and purification by FCC (5:1 pentane:EtOAc), the title compound was afforded as a colorless oil (21.1 mg, 43%).

IR (film) ν_{max}/cm^{-1} 2963, 1722, 1674

^{1}H NMR (400 MHz, $CDCl_3$) δ 7.38 – 7.29 (m, 2H), 7.24 – 7.16 (m, 2H), 3.56 (s, 2H), 3.00 (s, 2H), 2.70 – 2.61 (m, 2H), 2.36 (td, *J* = 7.0, 0.8 Hz, 2H), 2.01 – 1.90 (m, 2H), 1.32 (s, 9H).

^{13}C NMR (101 MHz, $CDCl_3$) δ 207.4, 150.4, 134.3, 128.9, 125.4, 64.7, 62.4, 51.8, 38.9, 34.6, 31.5, 24.2.

HRMS (ES+) exact mass calculated for $[M+H]^+$ ($C_{16}H_{24}NO$) requires m/z 246.1852, found m/z 246.1856

Compound 3o

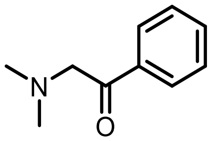

Following General Procedure 4, amide **1o** (32.6 mg, 0.20 mmol) was reacted to afford the title compound as a colorless oil (10.3 mg, 32%) following purification by FCC (pentane:Et_2O 2:1 to 0:1 with 1% Et_3N). Data were in agreement with the literature.[12]

^{1}H NMR (400 MHz, $CDCl_3$) δ 8.01 – 7.95 (m, 2H), 7.58 – 7.53 (m, 1H), 7.50 – 7.41 (m, 2H), 3.76 (s, 2H), 2.38 (s, 6H).

^{13}C NMR (101 MHz, $CDCl_3$) δ 197.0, 136.2, 133.3, 128.7, 128.3, 65.7, 45.9.

Compound 3p

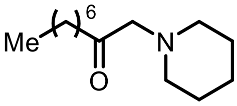

Following General Procedure 4, amide **1p** (47.8 mg, 0.20 mmol) was reacted to afford the title compound as a colorless oil (18.2 mg, 38%) following purification by FCC (1st column: pentane:CH_2Cl_2 1:1 to 0:1 with 1% Et_3N; 2nd column: 19:1 CH_2Cl_2:MeOH).

IR (film) ν_{max}/cm^{-1} 2957, 2928, 1719.

^{1}H NMR (400 MHz, $CDCl_3$) δ 3.33 (s, 2H), 2.64 – 2.50 (m, 4H), 2.40 (t, *J* = 7.5 Hz, 2H), 1.87 – 1.71 (m, 4H), 1.66 – 1.49 (m, 2H), 1.34 – 1.20 (m, 10H), 0.90 – 0.81 (m, 3H).

^{13}C NMR (101 MHz, $CDCl_3$) δ 209.1, 65.8, 54.4, 40.5, 32.0, 29.5, 29.4, 29.3, 24.1, 23.8, 22.8, 14.2.

HRMS (ES+) exact mass calculated for $[M+Na]^+$ ($C_{14}H_{27}NONa$) requires m/z 248.1985, found m/z 248.1980

Compound 3q

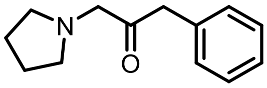

Following a modification to General Procedure 4, lactam **1q** (40.6 mg, 0.20 mmol) was reacted in the usual manner however following stirring with mCPBA at –78 °C for 10 minutes, pH 5 aq. buffer (NaOAc/AcOH, 2.4 mL) was added and the reaction mixture was stirred for 2 hours at room temperature. The title compound was afforded as a colorless oil (13.9 mg, 34%) following purification by FCC (cold pentane:Et_2O 1:1 to 0:1 with 1% Et_3N).

IR (film) ν_{max}/cm^{-1} 2961, 1723

^{1}H NMR (400 MHz, $CDCl_3$) δ 7.40 – 7.15 (m, 5H), 3.73 (s, 2H), 3.39 (s, 2H), 2.59 – 2.50 (m, 4H), 1.82 – 1.76 (m, 4H).

^{13}C NMR (101 MHz, $CDCl_3$) δ 206.3, 134.3, 129.5, 128.8, 127.1, 64.7, 54.3, 47.8, 23.9.

HRMS (ES+) exact mass calculated for $[M+H]^+$ ($C_{13}H_{18}NO$) requires m/z 204.1380, found m/z 204.1383

Compound 3r

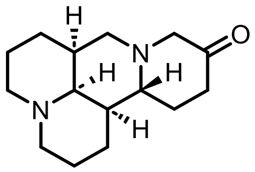

Before use, commercial matrine (thermoscientific) was dissolved in Et_2O (100 mg in 20 mL) and washed with sat. aq. $NaHCO_3$ (2 x 10 mL). The Et_2O layer was dried with Na_2SO_4, filtered and concentrated under reduced pressure to yield pure matrine as a white solid. This step was critical for ensuring complete, reproducible reduction by Vaska's complex.

Following a modification to General Procedure 4, matrine (39.7 mmol, 0.16 mmol) was reacted with increased loading of Vaska's complex (2.4 mg, 1.9 mol%) and TMDS (53.3 µL, 0.30 mmol), and following stirring with mCPBA at –78 °C for 10 minutes, pH 5 aq. buffer (NaOAc/AcOH, 2.4 mL) was added and the reaction mixture was stirred for 2 hours at room temperature. Following the standard work-up procedure, analysis of the crude mixture by ^{1}H NMR revealed 52% of the title compound, using 1,2,4,5-tetramethylbenzene as internal standard. Purification by FCC (3:1 to 1:1 pentane:EtOAc with 1% Et_3N) afforded the title compound as a colorless oil (11.1, 28%)

IR (film) ν_{max}/cm^{-1} 2937, 1727, 1103

^{1}H NMR (400 MHz, $CDCl_3$) δ 3.24 (dd, *J* = 14.7, 2.1 Hz, 1H), 2.92 (d, *J* = 14.7 Hz, 1H), 2.89 – 2.71 (m,

4H), 2.50 (ddt, J = 14.8, 4.4, 2.3 Hz, 1H), 2.39 – 2.18 (m, 3H), 2.07 (t, J = 3.1 Hz, 1H), 1.93 (ddt, J = 18.6, 10.7, 2.9 Hz, 4H), 1.83 – 1.65 (m, 2H), 1.59 – 1.37 (m, 7H).

^{13}C NMR (101 MHz, $CDCl_3$). δ 207.1, 66.1, 63.9, 57.7, 57.5, 56.3, 55.9, 41.5, 39.1, 35.3, 29.2, 28.2, 26.9, 21.7, 21.5.

HRMS (ES+) exact mass calculated for $[M+H]^+$ ($C_{15}H_{25}N_2O$) requires m/z 249.1961, found m/z 249.1968

Compound 3s

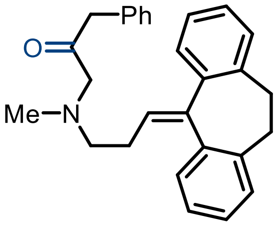

Following a modification to General Procedure 4, amide **1x** (79.2 mg, 0.20 mmol) was reacted and, following stirring with mCPBA at –78 °C for 10 minutes, pH 5 aq. buffer (NaOAc/AcOH, 2.4 mL) was added and the reaction mixture was stirred for 2 hours at room temperature. Following purification by FCC (4:1 to 3:1 pentane:EtOAc) the title compound was afforded as a colorless oil (29.4 mg, 37%)

IR (film) ν_{max}/cm^{-1} 2920, 1720, 1602

^{1}H NMR (400 MHz, $CDCl_3$) δ 7.32 – 7.10 (m, 12H), 7.06 – 7.02 (m, 1H), 5.86 (t, J = 7.4 Hz, 1H), 3.70 (s, 2H), 3.34 (d, J = 39.3 Hz, 2H), 3.15 (s, 2H), 2.97 (s, 1H), 2.88 – 2.72 (m, 0H), 2.51 (q, J = 6.0 Hz, 2H), 2.28 (q, J = 7.1 Hz, 2H), 2.19 (s, 3H).

^{13}C NMR (101 MHz, $CDCl_3$) δ 207.3, 143.9, 141.3, 140.1, 139.5, 137.2, 134.2, 130.1, 129.5, 129.1, 128.8, 128.7, 128.3, 128.2, 127.6, 127.2, 127.1, 126.2, 125.9, 66.2, 57.5, 47.4, 42.5, 33.9, 32.2, 27.6.

HRMS (ES+) exact mass calculated for $[M+H]^+$ ($C_{28}H_{30}NO$) requires m/z 396.2322, found m/z 396.2329

Compound 3t

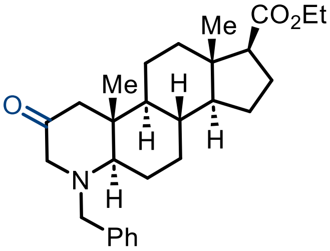

Following a modification to General Procedure 4, Amide **1y** (43.7 mg, 0.10 mmol) was reacted and, following stirring with mCPBA at –78 °C for 10 minutes, pH 5 aq. buffer (NaOAc/AcOH, 1.2 mL) was added and the reaction mixture was stirred for 2 hours at room temperature. Following the standard work-up procedure, analysis of the crude mixture by ^{1}H NMR revealed 71% of the title compound, using 1,2,4,5-

tetramethylbenzene as internal standard. Purification by FCC (9:1 to 5:1 pentane:EtOAc) afforded the title compound as an amorphous white solid (21.5 mg, 49%).

IR (film) ν_{max}/cm^{-1} 2942, 1729

^{1}H NMR (400 MHz, $CDCl_3$) δ 7.32 – 7.18 (m, 5H), 4.23 – 4.03 (m, 3H), 3.26 – 3.15 (m, 2H), 2.64 (d, *J* = 14.5 Hz, 1H), 2.49 (dd, *J* = 14.6, 2.0 Hz, 1H), 2.39 – 2.28 (m, 2H), 2.20 – 1.95 (m, 4H), 1.87 – 1.64 (m, 3H), 1.48 (dq, *J* = 12.1, 3.3 Hz, 1H), 1.41 – 1.21 (m, 8H), 1.14 – 0.84 (m, 6H), 0.66 (s, 3H).

^{13}C NMR (101 MHz, $CDCl_3$) δ 207.6, 174.1, 139.3, 128.6, 128.5, 127.1, 68.8, 64.1, 60.1, 57.1, 55.7, 55.3, 53.4, 52.4, 44.1, 40.9, 38.4, 34.9, 30.6, 25.7, 24.5, 23.7, 21.1, 14.6, 14.9, 13.6.

HRMS (ES+) exact mass calculated for $[M+Na]^+$ ($C_{28}H_{39}NO_3Na$) requires m/z 460.2822, found m/z 460.2808

5.4 Aminoalcohol synthesis

Compound 4a

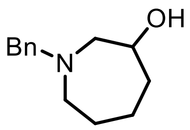

Following General Procedure 5, lactam **1a** (40.6 mg, 0.20 mmol) was reacted to afford the title compound as a colorless oil (24.8 mg, 60%) following purification by FCC (EtOAc:MeOH 1:0 to 9:1).

IR (film) ν_{max}/cm^{-1} 3392, 2928, 1397.

^{1}H NMR (400 MHz, $CDCl_3$) δ 7.36 – 7.23 (m, 5H), 3.79 (dtd, *J* = 5.6, 4.0, 1.5 Hz, 1H), 3.71 (s, 2H), 2.84 (ddt, *J* = 13.3, 5.6, 1.3 Hz, 1H), 2.79 – 2.71 (m, 2H), 2.45 (ddd, *J* = 11.6, 6.1, 4.5 Hz, 1H), 1.88 – 1.75 (m, 2H), 1.73 – 1.63 (m, 1H), 1.61 – 1.43 (m, 3H).

^{13}C NMR (101 MHz, $CDCl_3$) δ 139.2, 129.1, 128.5, 127.4, 67.7, 64.2, 57.8, 55.0, 37.6, 28.4, 20.9.

HRMS (ES+) exact mass calculated for $[M+H]^+$ ($C_{13}H_{20}NO$) requires m/z 206.1539, found m/z 206.1547

Compound 4b

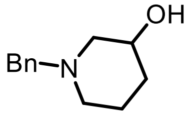

Following General Procedure 5, lactam **1b** (37.8 mg, 0.20 mmol) was reacted to afford the title compound as a colorless oil (23.4 mg, 61%) following purification by FCC (pentane:EtOAc 1:1 to 0:1 with 1% Et_3N).

IR (film) ν_{max}/cm^{-1} 3306, 2938, 1454.

^{1}H NMR (400 MHz, $CDCl_3$) δ 7.36 – 7.11 (m, 5H), 3.81 (p, *J* = 4.4 Hz, 1H), 3.51 (s, 2H), 2.47 (s, 4H), 2.31 – 2.21 (m, 1H), 1.78 (ddt, *J* =fz 17.2, 9.2, 4.4 Hz, 1H), 1.67 – 1.44 (m, 3H).

^{13}C NMR (101 MHz, $CDCl_3$) δ 138.3, 129.2, 128.4, 127.2, 66.4, 63.1, 60.3, 53.6, 31.8, 21.7.
HRMS (ES+) exact mass calculated for $[M+H]^+$ ($C_{12}H_{18}NO$) requires m/z 192.1383, found m/z 192.1390

Compound 4c

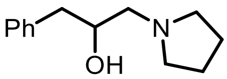

Following General Procedure 5, amide **1p** (40.6 mg, 0.20 mmol) was reacted to afford the title compound as a colorless oil (26.5 mg, 65%) following purification by FCC (EtOAc:MeOH 1:0 to 9:1 with 1% Et_3N).
IR (film) ν_{max}/cm^{-1} 3432, 2964, 2804.
^{1}H NMR (400 MHz, $CDCl_3$) δ 7.33 – 7.18 (m, 5H), 3.91 (dddd, *J* = 10.2, 7.0, 5.6, 3.1 Hz, 1H), 3.38 (s, 1H), 2.83 (dd, *J* = 13.7, 7.0 Hz, 1H), 2.74 – 2.58 (m, 4H), 2.45 (tdd, *J* = 6.8, 3.3, 1.3 Hz, 2H), 2.32 (dd, *J* = 12.0, 3.1 Hz, 1H), 1.76 (td, *J* = 5.4, 2.4 Hz, 4H).
^{13}C NMR (101 MHz, $CDCl_3$) δ 138.6, 129.5, 128.5, 126.4, 69.4, 61.6, 54.1, 41.7, 23.7.
HRMS (ES+) exact mass calculated for $[M+Na]^+$ ($C_{13}H_{19}NONa$) requires m/z 228.1359, found m/z 228.1365

Compound 4d

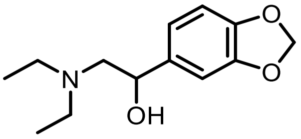

Following General Procedure 5, amide **1q** (47.0 mg, 0.20 mmol) was reacted to afford the title compound as a colorless oil (14.9 mg, 31%) following purification by FCC (cold pentane:Me_2CO 6:1 to 4:1 with 1% Et_3N).
IR (film) ν_{max}/cm^{-1} 3408, 2971, 1489, 1039.
^{1}H NMR (400 MHz, $CDCl_3$) δ 6.90 (d, *J* = 1.6 Hz, 1H), 6.85 – 6.71 (m, 2H), 5.93 (s, 2H), 4.54 (dd, *J* = 10.5, 3.6 Hz, 1H), 2.71 (dq, *J* = 13.0, 7.3 Hz, 2H), 2.64 – 2.49 (m, 3H), 2.39 (dd, *J* = 12.8, 10.5 Hz, 1H), 1.06 (t, *J* = 7.1 Hz, 6H).
^{13}C NMR (101 MHz, $CDCl_3$) δ 147.8, 146.9, 136.9, 119.3, 108.2, 106.6, 101.0, 69.2, 62.0, 47.0, 12.2.
HRMS (ES+) exact mass calculated for $[M+Na]^+$ ($C_{13}H_{19}NO_3Na$) requires m/z 260.1257, found m/z 260.1259

Compound 4e

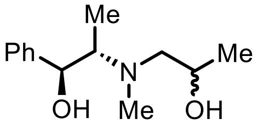

Following General Procedure 5, (1S,2S)-(+)-pseudoephedrine (44.4 mg, 0.20 mmol) was reacted, with increased TMDS (92 μL, 5.2 mmol, 2.6 eq.), to afford the title compound as a mixture of diastereomers as a colorless oil (15.9 mg, 35%) following purification by FCC (1st column: pentane:EtOAc:MeOH 5:1:0 to 0:9:1 with 1% Et_3N; 2nd column: pentane:EtOAc 1:1 to 0:1 with 1% Et_3N). Crude d.r. was determined to be 2.2:1 by ^{1}H NMR.

IR (film) ν_{max}/cm^{-1} 3359, 3310, 2969, 1456, 1040

^{1}H NMR (400 MHz, $CDCl_3$) δ 7.40 – 7.23 (m, 5H), 4.33 (dd, *J* = 9.7, 6.4 Hz, 1H), 3.96 (dtp, *J* = 9.4, 6.2, 2.9 Hz, 1H), 3.90 – 3.46 (m, 2H), 2.84 – 2.70 (m, 1H), 2.61 – 2.49 (m, 1H), 2.42 (s, 2H), 2.41 – 2.34 (m, 1H), 2.32 (s, 1H), 1.20 (dd, *J* = 6.2, 1.9 Hz, 3H), 0.75 (dd, *J* = 6.7, 5.9 Hz, 3H).

^{13}C NMR (101 MHz, $CDCl_3$) δ 141.8, 141.7, 128.4, 128.0, 127.5, 75.4, 74.9, 67.3, 65.3, 64.8, 64.2, 63.62, 59.9, 38.7, 34.8, 20.9, 20.8, 8.2, 8.0.

HRMS (ES+) exact mass calculated for $[M+Na]^+$ ($C_{13}H_{21}NO_2Na$) requires m/z 246.1465, found m/z 246.1458

Note: $LiAlH_4$ was applied as a 2M solution in THF, in place of the 1M solution in Et_2O typically used, due to availability of the commercial reagent.

Compound 4f

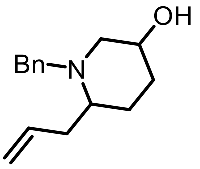

Following General Procedure 5, lactam **1s** (229 mg, 1.0 mmol) was reacted to afford the title compound as a two diastereomers (2:1 d.r., 161 mg, 70% total yield) following purification by FCC (1:1 to 0:1 pentane:EtOAc), diastereomer 4f-A (48 mg) and diastereomer 4f-B (113 mg).

4f-A:

IR (film) ν_{max}/cm^{-1} 3388, 2940, 1453

^{1}H NMR (400 MHz, $CDCl_3$) δ 7.34 – 7.27 (m, 4H), 7.26 – 7.21 (m, 1H), 5.96 – 5.81 (m, 1H), 5.15 – 5.04 (m, 2H), 4.10 (d, *J* = 13.2 Hz, 1H), 3.74 (dp, *J* = 4.8, 2.4 Hz, 1H), 3.21 (d, *J* = 13.3 Hz, 1H), 2.73 (ddd, *J* = 11.6, 4.8, 1.7 Hz, 1H), 2.57 – 2.29 (m, 4H), 2.18 (dd, *J* = 11.6, 2.1 Hz, 1H), 1.74 (dtdd, *J* = 10.1, 6.4, 3.5, 1.8 Hz, 2H), 1.62 – 1.45 (m, 2H).

^{13}C NMR (101 MHz, $CDCl_3$) δ 139.1, 135.2, 129.0, 128.5, 127.1, 117.2, 65.6, 60.1, 57.7, 57.4, 36.3, 30.6, 25.9.

HRMS (ES+) exact mass calculated for $[M+H]^+$ ($C_{15}H_{22}NO$) requires m/z 232.1696, found m/z 232.1693

4f-B:

IR (film) ν_{max}/cm^{-1} 3350, 2938, 1453

^{1}H NMR (400 MHz, $CDCl_3$) δ 7.34 – 7.27 (m, 4H), 7.24 (ddd, *J* = 8.6, 5.2, 3.5 Hz, 1H), 5.81 (dddd, *J* = 16.8, 10.3, 7.6, 6.4 Hz, 1H), 5.14 – 5.02 (m, 2H), 3.90 (d, *J* = 13.2 Hz, 1H), 3.68 (ddt, *J* = 10.8, 7.2, 3.3 Hz, 1H), 3.37 (d, *J* = 13.2 Hz, 1H), 2.85 (ddd, *J* = 11.3, 3.3, 1.2 Hz, 1H), 2.52 – 2.29 (m, 3H), 2.08 (dd, *J* = 11.3, 7.1 Hz, 2H), 1.90 – 1.75 (m, 2H), 1.58 – 1.44 (m, 1H), 1.44 – 1.33 (m, 1H).

^{13}C NMR (101 MHz, $CDCl_3$) δ 139.1, 135.9, 129.1, 128.4, 127.1, 116.8, 66.7, 58.7, 58.3, 56.9, 32.9, 30.6, 26.4.

HRMS (ES+) exact mass calculated for $[M+Na]^+$ ($C_{15}H_{21}NONa$) requires m/z 254.1515, found m/z 254.1522

(±)-pseudoconhydrine

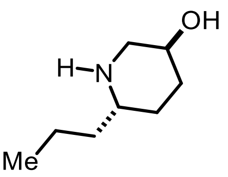

To a solution of **4e-B** (54 mg, 0.24) in MeOH (18 mL) was added Pd/C (27 mg), Pd black (27 mg) and 1M aq. HCl (0.47 mL, 0.47 mmol). The resulting mixture was sparged with N_2 via a balloon for 5 minutes and then sparged with H_2 via a balloon for a further 5 minutes. The mixture was stirred under a H_2 atmosphere for 5 hours. The resulting suspension was passed through a celite plug and the volatiles were removed under reduced pressure. Sat. aq. K_2CO_3 was added to the crude residue and the resulting aqueous phase was extracted with CH_2Cl_2 (3 x 20 mL). The combined organics were dried with Na_2SO_4, filtered and concentrated under reduced pressure to yield the (±)-pseudoconhydrine as a white solid (32 mg, 95%). Data were in agreement with the literature.[13]

mp 72-74 °C

IR (film) ν_{max}/cm^{-1} 3146, 2956, 1052

^{1}H NMR (400 MHz, $CDCl_3$) δ 3.60 (tdd, *J* = 11.2, 4.6, 1.9 Hz, 1H), 3.20 (ddt, *J* = 11.3, 4.9, 2.4 Hz, 1H), 2.48 – 2.35 (m, 2H), 2.03 (dqq, *J* = 11.9, 3.4, 1.9 Hz, 1H), 1.73 (dq, *J* = 13.4, 3.5 Hz, 3H), 1.39 – 1.23 (m, 5H), 1.18 – 1.05 (m, 1H), 0.90 (ddd, *J* = 7.2, 5.1, 2.6 Hz, 3H).

^{13}C NMR (101 MHz, $CDCl_3$) δ 68.3, 55.7, 54.2, 38.7, 34.2, 31.4, 19.5, 14.3.

HRMS (ES+) exact mass calculated for $[M+H]^+$ ($C_8H_{18}NO$) requires m/z 144.1383, found m/z 144.1379

Compound 4g

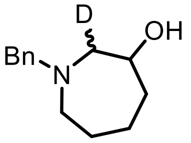

Following a modification to General Procedure 4, $NaBD_4$ (34 mg, 0.40 mmol) in MeOH (0.50 mL) was employed as a reductant in place of $LiAlH_4$. Following stirring for 2 hours, sat. aq. $NaHCO_3$ was added and the resulting biphasic mixture was extracted with CH_2Cl_2 (3 x 20 mL). The combined organics were dried with Na_2SO_4, filtered and concentrated under reduced pressure. The crude residue was purified by FCC (1:1 pentane:Et_2O, 1% Et_3N) to yield the title compound as a colourless oil (18 mg, 44%, d.r. 1.6:1).*

IR (film) ν_{max}/cm^{-1} 3403, 2929, 1454

^{1}H NMR (400 MHz, $CDCl_3$) δ 7.38 – 7.24 (m, 5H), 3.78 (dt, *J* = 5.7, 2.7 Hz, 1H), 3.71 (s, 2H), 3.65 – 3.57 (m, 1H), 2.85 – 2.68 (m, 2H), 2.51 – 2.39 (m, 1H), 1.90 – 1.70 (m, 2H), 1.76 – 1.61 (m, 1H), 1.58 – 1.43 (m, 3H).

^{13}C NMR (101 MHz, $CDCl_3$) δ 139.3 (app. d, *J* = 2.9 Hz), 129.1, 128.5, 127.3, 67.6 (app. d, *J* = 8.4 Hz), 64.1 (app. d, *J* = 3.6 Hz), 57.7 – 57.1 (m), 54.9, 37.6, 28.4, 20.9.

HRMS (ES+) exact mass calculated for $[M+H]^+$ ($C_{13}H_{19}DNO$) requires m/z 207.1602, found m/z 207.1604

5.5 Synthesis of enaminones and derivatives

Compound 5a

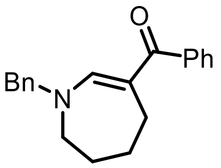

Following General Procedure 6, lactam **1a** (40.6 mg, 0.20 mmol) was reacted with benzoyl chloride (47 µL, 0.40 mmol) to afford the title compound as a low-melting point off-white solid (41.2 mg, 71%) following purification by FCC (cold pentane:Et_2O 1:1 to 0:1).

IR (film) ν_{max}/cm^{-1} 2929, 1579, 1560.

^{1}H NMR (400 MHz, $CDCl_3$) δ 7.49 – 7.41 (m, 2H), 7.38 – 7.29 (m, 6H), 7.19 – 7.16 (m, 2H), 7.13 (s, 1H),

---

* Diastereomeric ratio unable to be determined by ^{1}H NMR therefore ^{13}C NMR employed using the resonances corresponding to the secondary alcohol carbon.

4.20 (s, 2H), 3.41 – 3.33 (m, 2H), 2.80 – 2.70 (m, 2H), 1.91 – 1.73 (m, 4H).

^{13}C NMR (101 MHz, $CDCl_3$) δ 196.3, 157.7, 142.1 137.1, 129.3, 129.0, 128.5, 128.1, 127.9, 127.5, 112.9, 62.9, 51.6, 28.1, 26.3, 24.3.

HRMS (ES+) exact mass calculated for $[M+Na]^+$ ($C_{20}H_{21}NONa$) requires m/z 314.1515, found m/z 314.1521

Compound 5b

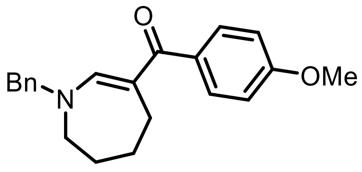

Following General Procedure 6, lactam **1a** (40.6 mg, 0.20 mmol) was reacted with 4-methoxybenzoyl chloride (68.2 mg, 0.40 mmol) to afford the title compound as a colorless oil (30.3 mg, 47%) following purification by FCC (cold Et_2O).

IR (film) ν_{max}/cm^{-1} 1603, 1579, 1558, 1250.

^{1}H NMR (400 MHz, $CDCl_3$) δ 7.48 – 7.43 (m, 2H), 7.42 – 7.26 (m, 3H), 7.22 – 7.17 (m, 2H), 7.15 (s, 1H), 6.89 – 6.81 (m, 2H), 4.21 (s, 2H), 3.82 (s, 3H), 3.41 – 3.33 (m, 2H), 2.79 – 2.69 (m, 2H), 1.90 – 1.72 (m, 4H).

^{13}C NMR (101 MHz, $CDCl_3$) δ 195.6, 160.8, 157.0, 137.3, 134.4, 130.6, 128.9, 128.0, 127.5, 113.2, 112.8, 62.8, 55.4, 51.6, 28.2, 26.3, 24.6.

HRMS (ES+) exact mass calculated for $[M+Na]^+$ ($C_{21}H_{23}NO_2Na$) requires m/z 344.1621, found m/z 344.1612

Compound 5c

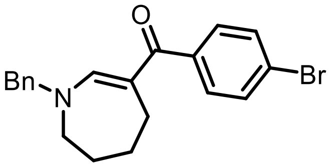

Following General Procedure 6, lactam **1a** (40.6 mg, 0.20 mmol) was reacted with 4-bromobenzoyl chloride (87.8 mg, 0.40 mmol) to afford the title compound as a colorless oil (44.8 mg, 61%) following purification by FCC (cold pentane:Et_2O 1:1 to 0:1).

IR (film) ν_{max}/cm^{-1} 1633, 1572, 1555.

^{1}H NMR (500 MHz, $CDCl_3$) δ 7.49 – 7.42 (m, 2H), 7.38 – 7.27 (m, 5H), 7.19 – 7.11 (m, 2H), 7.06 (s, 1H), 4.21 (s, 2H), 3.41 – 3.35 (m, 2H), 2.76 – 2.70 (m, 2H), 1.89 – 1.74 (m, 4H).

^{13}C NMR (126 MHz, $CDCl_3$) δ 194.8, 157.5, 140.8, 136.8, 131.1, 130.2, 129.0, 128.2, 127.6, 123.6, 112.7, 62.9, 51.7, 28.0, 26.2, 24.2.

HRMS (ES+) exact mass calculated for $[M+H]^+$ ($C_{20}H_{21}BrNO$) requires m/z 370.0801, found m/z 370.0791

Compound 5d

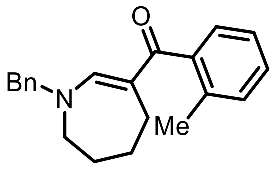

Following General Procedure 6, lactam **1a** (40.6 mg, 0.20 mmol) was reacted with 2-methylbenzoyl chloride (52.0 µL, 0.40 mmol) to afford the title compound as a white solid (17.9 mg, 29%) following purification by FCC (cold pentane:Et_2O 2:1).

mp 66-68 °C

IR (film) ν_{max}/cm^{-1} 1568, 1359.

^{1}H NMR (400 MHz, $CDCl_3$) δ 7.36 – 7.28 (m, 3H), 7.23 – 7.17 (m, 1H), 7.16 – 7.08 (m, 5H), 6.96 (s, 1H), 4.13 (s, 2H), 3.39 – 3.30 (m, 2H), 2.80 – 2.72 (m, 2H), 2.26 (s, 3H), 1.90 – 1.73 (m, 4H).

^{13}C NMR (101 MHz, $CDCl_3$) δ 197.2, 157.6, 142.0, 136.9, 134.9, 130.3, 129.0, 128.1, 128.1, 127.5, 127.2, 125.2, 113.9, 63.0, 51.5, 28.1, 26.3, 23.5, 19.4.

HRMS (ES+) exact mass calculated for $[M+Na]^+$ ($C_{21}H_{23}NONa$) requires m/z 328.1672, found m/z 328.1664

Compound 5e

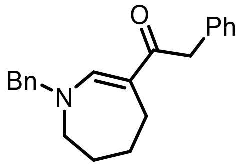

Following General Procedure 6, lactam **1a** (40.6 mg, 0.20 mmol) was reacted with phenylacetyl chloride (53 µL, 0.40 mmol) to afford the title compound as a colorless oil (17.0 mg, 28%) following purification by FCC (cold pentane:Et_2O 3:1 to 1:1).

IR (film) ν_{max}/cm^{-1} 2930, 1588, 1575, 1360

^{1}H NMR (400 MHz, $CDCl_3$) δ 7.53 (s, 1H), 7.39 – 7.24 (m, 5H), 7.25 – 7.16 (m, 2H), 7.18 – 7.13 (m, 2H), 4.30 (s, 2H), 3.84 (s, 2H), 3.29 (dd, J = 6.5, 4.6 Hz, 2H), 2.60 (dd, J = 7.5, 4.2 Hz, 2H), 1.81 – 1.66 (m, 4H).

^{13}C NMR (101 MHz, $CDCl_3$) δ 195.7, 153.5, 137.8, 137.2, 129.0, 128.9, 128.6, 128.1, 127.6, 126.3, 112.0, 62.9, 51.3, 44.9, 28.0, 26.1, 23.9.

HRMS (ES+) exact mass calculated for $[M+H]^+$ ($C_{21}H_{24}NO$) requires m/z 306.1852, found m/z 306.1896

Compound 5f

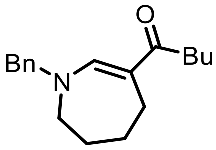

Following General Procedure 6, lactam **1a** (40.6 mg, 0.20 mmol) was reacted with pentanoyl chloride (48 µL, 0.40 mmol) to afford the title compound as a colourless oil (19.8 mg, 37%) following purification by FCC (cold pentane:EtOAc 3:1).

IR (film) ν_{max}/cm^{-1} 2932, 1594

^{1}H NMR (400 MHz, $CDCl_3$) δ 7.44 (s, 1H), 7.40 – 7.34 (m, 2H), 7.34 – 7.30 (m, 1H), 7.28 – 7.25 (m, 2H), 4.35 (s, 2H), 3.31 (dd, *J* = 6.5, 4.7 Hz, 2H), 2.60 (dd, *J* = 7.5, 4.1 Hz, 2H), 2.52 – 2.44 (m, 2H), 1.75 (ddt, *J* = 10.6, 9.1, 5.4 Hz, 4H), 1.58 (tt, *J* = 9.0, 6.9 Hz, 2H), 1.32 (dt, *J* = 14.6, 7.4 Hz, 2H), 0.90 (t, *J* = 7.3 Hz, 3H).

^{13}C NMR (101 MHz, $CDCl_3$) δ 198.9, 152.2, 137.9, 129.0, 128.0, 127.5, 112.8, 62.8, 51.4, 37.1, 28.9, 28.1, 26.2, 23.9, 23.0, 14.2.

HRMS (ES+) exact mass calculated for $[M+H]^+$ ($C_{18}H_{26}NO$) requires m/z 272.2009, found m/z 272.2010

Compound 5g

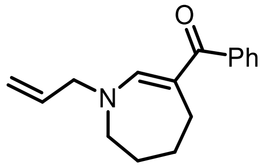

Following General Procedure 6, lactam **1a** (30.6 mg, 0.20 mmol) was reacted with benzoyl chloride (47 µL, 0.40 mmol) to afford the title compound as a colorless oil (35.7 mg, 74%) following purification by FCC (cold pentane:Et_2O 1:1 to 0:1).

IR (film) ν_{max}/cm^{-1} 1582, 1562, 1257

^{1}H NMR (400 MHz, $CDCl_3$) δ 7.43 – 7.39 (m, 2H), 7.36 – 7.29 (m, 3H), 6.95 (s, 1H), 5.73 (ddt, *J* = 17.0, 10.3, 5.8 Hz, 1H), 5.21 – 5.09 (m, 2H), 3.60 (dt, *J* = 5.8, 1.5 Hz, 2H), 3.40 – 3.32 (m, 2H), 2.77 – 2.69 (m, 2H), 1.86 (qd, *J* = 4.4, 2.2 Hz, 4H).

^{13}C NMR (101 MHz, $CDCl_3$) δ 196.1, 157.4, 142.0, 133.5, 129.2, 128.5, 127.8, 118.2, 112.8, 61.5, 51.4, 28.2, 26.2, 24.2.

HRMS (ES+) exact mass calculated for $[M+Na]^+$ ($C_{16}H_{19}NONa$) requires m/z 264.1359, found m/z 264.1364

Compound 5h

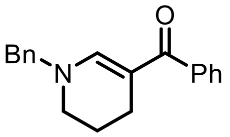

Following General Procedure 6, lactam **1b** (37.8 mg, 0.20 mmol) was reacted with benzoyl chloride (47 µL, 0.40 mmol) to afford the title compound as a colorless oil (30.5 mg, 55%) following purification by FCC (cold pentane:Et_2O 1:1).

IR (film) ν_{max}/cm^{-1} 1581, 1557, 1354.

^{1}H NMR (400 MHz, $CDCl_3$) δ 7.50 – 7.46 (m, 2H), 7.41 – 7.26 (m, 6H), 7.19 (s, 1H), 7.17 – 7.13 (m, 2H), 4.25 (s, 2H), 3.13 – 3.06 (m, 2H), 2.51 (t, *J* = 6.2 Hz, 2H), 1.92 – 1.81 (m, 2H).

^{13}C NMR (101 MHz, $CDCl_3$) δ 193.4, 152.5, 141.2, 136.3, 129.3, 129.0, 128.4, 128.1, 128.0, 127.4, 108.1, 60.2, 46.2, 21.0, 19.6.

HRMS (ES+) exact mass calculated for $[M+Na]^+$ ($C_{19}H_{19}NONa$) requires m/z 300.1359, found m/z 300.1354

Compound 5i

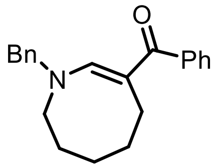

Following General Procedure 6, lactam **1c** (43.4 mg, 0.20 mmol) was reacted with benzoyl chloride (47 µL, 0.40 mmol) to afford the title compound as an off-white solid (50.3 mg, 82%) following purification by FCC (cold pentane:Et_2O 1:1 to 0:1).

mp 62-66 °C

IR (film) ν_{max}/cm^{-1} 1578, 1560.

^{1}H NMR (400 MHz, $CDCl_3$) δ 7.47 – 7.40 (m, 2H), 7.39 – 7.26 (m, 7H), 7.20 – 7.13 (m, 2H), 4.17 (s, 2H), 3.63 (t, *J* = 6.4 Hz, 2H), 2.98 (t, *J* = 7.0 Hz, 2H), 1.80 (p, *J* = 6.9 Hz, 2H), 1.71 (p, *J* = 6.4 Hz, 2H), 1.66 – 1.54 (m, 2H).

^{13}C NMR (101 MHz, $CDCl_3$) δ 196.7, 158.0, 142.2, 136.9, 129.1, 129.0, 128.5, 128.2, 127.9, 127.8, 109.3, 62.5, 47.9, 29.8, 29.0, 22.3, 21.9.

HRMS (ES+) exact mass calculated for $[M+Na]^+$ ($C_{21}H_{23}NONa$) requires m/z 328.1672, found m/z

328.1668

Compound 5j

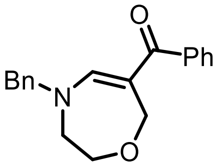

Following a modification to General Procedure 6, lactam **1t** (41.1 mg, 0.20 mmol) was reacted in the usual manner however the enamine generation phase was left for 3 hours before addition of benzoyl chloride (47 μL, 0.40 mmol). Following the standard work-up procedure, the title compound was afforded as an off-white solid (14.6 mg, 25%) following purification by FCC (cold Et_2O).

mp 68-72 °C

IR (film) ν_{max}/cm^{-1} 1633, 1583, 1565, 1372, 1323

^{1}H NMR (400 MHz, $CDCl_3$) δ 7.48 – 7.44 (m, 2H), 7.40 – 7.30 (m, 6H), 7.23 (d, *J* = 0.7 Hz, 1H), 7.20 – 7.16 (m, 2H), 4.77 (s, 2H), 4.28 (s, 2H), 3.84 – 3.76 (m, 2H), 3.45 – 3.38 (m, 2H).

^{13}C NMR (101 MHz, $CDCl_3$) δ 195.4, 155.5, 141.0, 136.2, 129.8, 129.2, 128.5, 128.4, 128.1, 127.6, 110.9, 70.3, 69.6, 63.1, 54.4.

HRMS (ES+) exact mass calculated for $[M+H]^+$ ($C_{19}H_{20}NO_2$) requires m/z 294.1489, found m/z 294.1494

Compound 5k

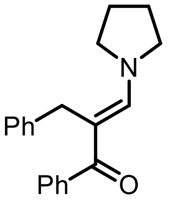

Following General Procedure 6, amide **1p** (40.6 mg, 0.20 mmol) was reacted with benzoyl chloride (47 μL, 0.40 mmol) to afford the title compound as a colorless oil (42.0 mg, 72%) following purification by FCC (cold pentane:Et_2O 3:1 to 1:1).

IR (film) ν_{max}/cm^{-1} 1624, 1560, 1308.

^{1}H NMR (400 MHz, $CDCl_3$) δ 7.54 – 7.44 (m, 2H), 7.40 – 7.34 (m, 3H), 7.32 – 7.21 (m, 6H), 7.17 – 7.12 (m, 1H), 4.06 (s, 2H), 3.46 – 3.37 (m, 4H), 1.87 – 1.75 (m, 4H).

^{13}C NMR (101 MHz, $CDCl_3$) δ 196.1, 152.9, 142.9, 142.0, 128.9, 128.2, 128.2, 127.7, 127.6, 125.2, 109.0, 51.2, 29.7, 25.1.

HRMS (ES+) exact mass calculated for $[M+Na]^+$ ($C_{20}H_{21}NONa$) requires m/z 314.1515, found m/z 314.1508

Compound 5l

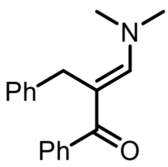

Following General Procedure 6, amide **1v** (35.4 mg, 0.20 mmol) was reacted with benzoyl chloride (47 μL, 0.40 mmol) to afford the title compound as a colorless oil (26.7 mg, 50%) following purification by FCC (cold pentane:Et_2O 3:1 to 1:1 with 1% Et_3N).

IR (film) ν_{max}/cm^{-1} 1631, 1581, 1562, 1330.

^{1}H NMR (400 MHz, $CDCl_3$) δ 7.54 – 7.46 (m, 2H), 7.41 – 7.34 (m, 3H), 7.32 – 7.23 (m, 2H), 7.22 (ddd, *J* = 8.0, 1.8, 0.9 Hz, 2H), 7.17 – 7.13 (m, 1H), 7.10 (s, 1H), 4.08 (s, 2H), 2.93 (s, 6H).

^{13}C NMR (101 MHz, $CDCl_3$) δ 196.7, 156.9, 142.7, 142.2, 129.3, 128.5, 128.5, 128.0, 127.9, 125.6, 108.6, 43.3, 29.8.

HRMS (ES+) exact mass calculated for $[M+Na]^+$ ($C_{18}H_{19}NONa$) requires m/z 288.1359, found m/z 288.1364

Compound 5m

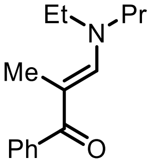

Following General Procedure 6, amide **1x** (25.8 mg, 0.20 mmol) was reacted with benzoyl chloride (47 μL, 0.40 mmol) to afford the title compound as a colorless oil (20.3 mg, 47%) following purification by FCC (cold pentane:Et_2O 3:1 to 1:1).

IR (film) ν_{max}/cm^{-1} 1625, 1580, 1563, 1325.

^{1}H NMR (400 MHz, $CDCl_3$) δ 7.45 – 7.28 (m, 5H), 6.91 (s, 1H), 3.30 (q, *J* = 7.2 Hz, 2H), 3.20 – 3.10 (m, 2H), 2.09 (s, 3H), 1.62 – 1.49 (m, 2H), 1.16 (t, *J* = 7.1 Hz, 3H), 0.87 (t, *J* = 7.4 Hz, 3H).

^{13}C NMR (101 MHz, $CDCl_3$) δ 197.2, 155.3, 142.4, 129.0, 128.4, 127.9, 105.3, 55.2, 47.4, 22.8, 14.9, 11.1, 11.0.

HRMS (ES+) exact mass calculated for $[M+Na]^+$ ($C_{15}H_{21}NONa$) requires m/z 254.1515, found m/z 254.1517

Compound 5n

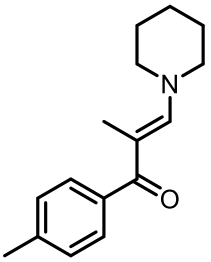

Following General Procedure 6, amide **1u** (28.2 mg, 0.20 mmol) was reacted with 4-methylbenzoyl chloride (53 μL, 0.40 mmol) to afford the title compound as a white solid (24.5 mg, 50%) following purification by FCC (cold pentane:Et_2O 3:1 to 1:1 with 1% Et_3N). *Note: residual acid chloride was present following FCC (<15% by ^{1}H NMR) – further purification was not performed due to high instability on silica gel. Given the excess of $LiAlH_4$ used in the subsequent step, this impurity is not proposed to impact the reduction. Adjusting for the impurity, the isolated yield corresponds to 45%.*

mp 64-70 °C

IR (film) ν_{max}/cm^{-1} 2937, 1579, 1316

^{1}H NMR (400 MHz, $CDCl_3$) δ 7.33 – 7.29 (m, 2H), 7.17 – 7.09 (m, 2H), 6.90 (d, *J* = 0.8 Hz, 1H), 3.41 – 3.34 (m, 4H), 2.36 (d, *J* = 2.2 Hz, 3H), 2.08 (d, *J* = 0.7 Hz, 3H), 1.71 – 1.53 (m, 6H).

^{13}C NMR (101 MHz, $CDCl_3$) δ 197.1, 155.0, 139.4, 139.1, 128.6, 128.5, 105.4, 52.3, 26.6, 24.3, 21.4, 12.0.

HRMS (ES+) exact mass calculated for $[M+Na]^+$ ($C_{16}H_{21}NONa$) requires m/z 266.1515, found m/z 266.1522

Tolperisone

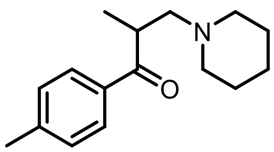

To a solution of enaminone **5n** (15.0 mg, 0.062 mmol) in THF (2.4 mL), cooled to 0 °C, was added $LiAlH_4$ (1M in THF, 185 μL, 0.185 mmol). The resulting mixture was stirred for 2.5 hours before being cooled to 0 °C once more. EtOAc (~100 μL) was added dropwise and the resulting mixture was stirred for 10 minutes. $Na_2SO_4.10H_2O$ was added and the heterogeneous mixture was stirred for a further 10 minutes. The mixture was filtered and the filtrate was concentrated under reduced pressure. This crude residue was purified by FCC (2:1 to 1:1 pentane:Et_2O with 1% Et_3N) to yield the title compound as a colorless oil (11.1 mg, 73%).

IR (film) ν_{max}/cm^{-1} 2935, 1680

^{1}H NMR (400 MHz, $CDCl_3$) δ 7.92 – 7.83 (m, 2H), 7.29 – 7.23 (m, 2H), 3.69 (td, *J* = 7.1, 5.9 Hz, 1H), 2.80 (dd, *J* = 12.5, 7.3 Hz, 1H), 2.41 (s, 3H), 2.40 – 2.31 (m, 5H), 1.48 (p, *J* = 5.8 Hz, 4H), 1.36 (dtd, *J* =

9.2, 5.5, 2.2 Hz, 2H), 1.17 (d, J = 7.0 Hz, 3H).

^{13}C NMR (101 MHz, $CDCl_3$) δ 203.8, 143.7, 134.5, 129.4, 128.6, 62.6, 55.1, 38.9, 26.2, 24.5, 21.8, 17.0.

HRMS (ES+) exact mass calculated for $[M+H]^+$ ($C_{16}H_{24}NO$) requires m/z 246.1852, found m/z 246.1864

Compound 5o

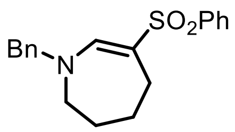

Following General Procedure 6, lactam **1a** (40.6 mg, 0.20 mmol) was reacted with benzenesulfonyl chloride (51.0 µL, 0.40 mmol) to afford the title compound as an off-white solid (16.3 mg, 25%) following purification by FCC (cold pentane:Et_2O 2:1).

mp 80-84 °C

IR (film) ν_{max}/cm^{-1} 1634, 1135, 1086

^{1}H NMR (400 MHz, $CDCl_3$) δ 7.89 – 7.80 (m, 2H), 7.63 (s, 1H), 7.57 – 7.43 (m, 3H), 7.39 – 7.29 (m, 3H), 7.28 – 7.22 (m, 2H), 4.37 (s, 2H), 3.19 – 3.11 (m, 2H), 2.34 (td, J = 4.9, 2.1 Hz, 2H), 1.64 (p, J = 2.8 Hz, 4H).

^{13}C NMR (101 MHz, $CDCl_3$) δ 148.4, 142.7, 137.3, 131.9, 129.0, 128.9, 128.1, 127.6, 127.2, 105.4, 63.1, 52.4, 28.2, 26.8, 26.7.

HRMS (ES+) exact mass calculated for $[M+H]^+$ ($C_{19}H_{22}NO_2S$) requires m/z 328.1366, found m/z 328.1372

Compound 5p

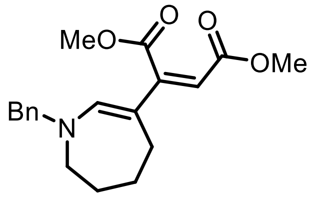

Following General Procedure 6, amide **1a** (20 mg, 0.10 mmol) was reacted with dimethyl acetylene dicarboxylate (37 µL, 0.30 mmol) to afford the title compound as a colorless oil (26.5 mg, 81%) following purification by FCC (pentane:Et_2O 3:1 to 0:1).

IR (film) ν_{max}/cm^{-1} 2981, 1718, 1695, 1599, 1255.

^{1}H NMR (400 MHz, $CDCl_3$) δ 7.83 (s, 1H), 7.39 – 7.23 (m, 5H), 6.94 (dd, J = 10.0, 6.3 Hz, 1H), 4.40 (s, 2H), 4.18 (ddd, J = 15.2, 8.5, 4.4 Hz, 1H), 3.74 (s, 3H), 3.64 (s, 3H), 2.70 – 2.61 (m, 1H), 2.50 (dtd, J = 13.1, 9.9, 3.0 Hz, 1H), 2.34 – 2.24 (m, 1H), 1.94 (ddtd, J = 14.2, 8.6, 5.6, 3.1 Hz, 1H), 1.80 (dddd, J = 12.3, 9.0, 6.0, 4.0 Hz, 2H), 1.65 – 1.53 (m, 1H).

^{13}C NMR (101 MHz, $CDCl_3$) δ 170.1, 169.1, 150.0, 144.5, 136.7, 129.6, 129.0, 128.2, 127.8, 92.0, 60.8,

52.1, 51.4, 43.7, 29.4, 28.4, 23.6.

HRMS (ES+) exact mass calculated for $[M+Na]^+$ ($C_{19}H_{23}NO_4$) requires m/z 330.1700, found m/z 330.1703

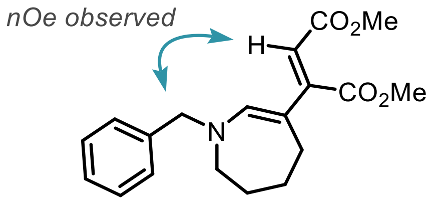

Compound 5q

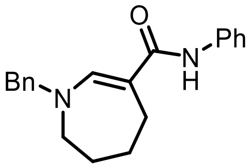

Following General Procedure 6, lactam **1a** (40.6 mg, 0.20 mmol) was reacted with phenyl isocyanate (48 μL, 0.40 mmol) to afford the title compound as an off-white solid (36.1 mg, 59%) following purification by FCC (cold pentane:Et_2O 2:1).

mp 132-142 °C

IR (film) ν_{max}/cm^{-1} 3305, 1597, 1499, 1311

^{1}H NMR (400 MHz, $CDCl_3$) δ 7.62 (s, 1H), 7.57 – 7.49 (m, 2H), 7.37 – 7.20 (m, 8H), 7.07 – 6.98 (m, 1H), 4.30 (s, 2H), 3.24 – 3.17 (m, 2H), 2.59 – 2.52 (m, 2H), 1.89 – 1.77 (m, 2H), 1.76 – 1.65 (m, 2H).

^{13}C NMR (101 MHz, $CDCl_3$) δ 169.2, 148.8, 139.4, 138.0, 128.9, 128.8, 127.8, 127.6, 123.2, 120.1, 101.2, 62.9, 52.2, 28.2, 27.0, 26.8.

HRMS (ES+) exact mass calculated for $[M+H]^+$ ($C_{20}H_{23}N_2O$) requires m/z 307.1805, found m/z 307.1815

Compound 5r

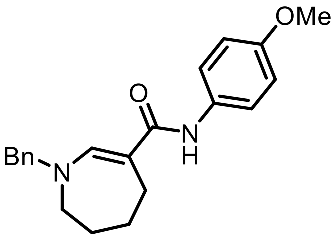

Following General Procedure 6, lactam **1a** (40.6 mg, 0.20 mmol) was reacted with 4-methoxyphenyl isocyanate (52 μL, 0.40 mmol) to afford the title compound as a waxy white solid (23.8 mg, 35%) following purification by FCC (cold pentane:Et_2O 2:1).

mp 90-94 °C

IR (film) ν_{max}/cm^{-1} 1618, 1510, 1220.

^{1}H NMR (400 MHz, $CDCl_3$) δ 7.60 (s, 1H), 7.47 – 7.38 (m, 2H), 7.37 – 7.23 (m, 5H), 7.12 (s, 1H), 6.88 – 6.78 (m, 2H), 4.30 (s, 2H), 3.77 (s, 3H), 3.24 – 3.16 (m, 2H), 2.58 – 2.50 (m, 2H), 1.88 – 1.78 (m, 2H), 1.71 (ddt, J = 12.0, 8.7, 4.5 Hz, 2H).

^{13}C NMR (101 MHz, $CDCl_3$) δ 169.2, 155.8, 148.5, 138.1, 132.5, 128.8, 127.7, 127.6, 122.0, 114.1, 101.2, 62.8, 55.6, 52.2, 28.3, 27.0, 26.9.

HRMS (ES+) exact mass calculated for $[M+Na]^+$ ($C_{21}H_{24}N_2O_2Na$) requires m/z 359.1730, found m/z 359.1736

Compound 5s

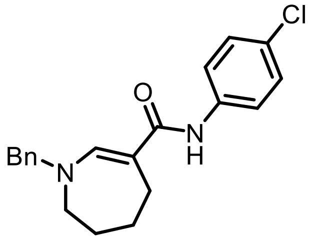

Following General Procedure 6, lactam **1a** (40.6 mg, 0.20 mmol) was reacted with 4-chlorophenyl isocyanate (52 μL, 0.40 mmol) to afford the title compound as a white solid (52 mg, 76%) following purification by FCC (cold pentane:Et_2O 2:1).

mp 130-136 °C

IR (film) ν_{max}/cm^{-1} 1618, 1594, 1493, 1307.

^{1}H NMR (400 MHz, $CDCl_3$) δ 7.61 (s, 1H), 7.52 – 7.43 (m, 2H), 7.37 – 7.29 (m, 2H), 7.29 – 7.20 (m, 5H), 4.31 (d, J = 1.9 Hz, 2H), 3.25 – 3.18 (m, 2H), 2.57 – 2.50 (m, 2H), 1.88 – 1.78 (m, 2H), 1.71 (pd, J = 5.7, 3.4 Hz, 2H).

^{13}C NMR (101 MHz, $CDCl_3$) δ 169.2, 149.1, 138.0, 137.8, 128.9, 128.8, 128.0, 127.8, 127.6, 121.3, 100.7, 62.9, 52.2, 28.2, 26.9, 26.8.

HRMS (ES+) exact mass calculated for $[M+H]^+$ ($C_{20}H_{22}ClN_2O$) requires m/z 341.1415, found m/z 341.1420

Compound 5t

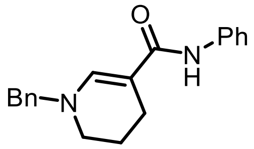

Following General Procedure 6, lactam **1b** (37.8 mg, 0.20 mmol) was reacted with phenyl isocyanate (48 μL, 0.40 mmol, 1 hour reaction instead of 30 min) to afford the title compound as a colorless oil (29.7 mg, 51%) following purification by FCC (pentane:Et_2O 1:1 to 0:1).

IR (film) ν_{max}/cm^{-1} 3055, 1648, 1622, 1597, 1575, 1310

^{1}H NMR (400 MHz, $CDCl_3$) δ 7.66 (d, *J* = 0.9 Hz, 1H), 7.59 – 7.52 (m, 2H), 7.37 – 7.26 (m, 5H), 7.24 – 7.20 (m, 2H), 7.07 – 6.95 (m, 2H), 4.29 (s, 2H), 3.01 (dd, *J* = 6.8, 4.5 Hz, 2H), 2.34 (t, *J* = 6.2 Hz, 2H), 1.96 – 1.85 (m, 2H).

^{13}C NMR (101 MHz, $CDCl_3$) δ 166.9, 144.9, 139.3, 137.2, 128.9, 128.8, 127.9, 127.7, 123.1, 119.9, 96.6, 59.9, 45.1, 21.5, 20.6.

HRMS (ES+) exact mass calculated for $[M+Na]^+$ ($C_{19}H_{20}N_2ONa$) requires m/z 315.1468, found m/z 315.1462

Compound 5u

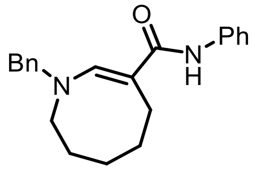

Following General Procedure 6, lactam **1c** (43.5 mg, 0.20 mmol) was reacted with phenyl isocyanate (48 µL, 0.40 mmol) to afford the title compound as a colorless oil (48.3 mg, 75%) following purification by FCC (cold pentane:Et_2O 1:1 with Et_3N).

mp 150-152 °C

IR (film) ν_{max}/cm^{-1} 3303, 2931, 1644, 1613, 1597.

^{1}H NMR (400 MHz, $CDCl_3$) δ 7.72 (s, 1H), 7.55 – 7.47 (m, 2H), 7.38 – 7.24 (m, 7H), 7.17 (s, 1H), 7.02 (tt, *J* = 7.3, 1.2 Hz, 1H), 4.31 (s, 2H), 3.52 (t, *J* = 6.0 Hz, 2H), 2.77 (t, *J* = 6.9 Hz, 2H), 1.81 (p, *J* = 6.6 Hz, 2H), 1.74 – 1.59 (m, 4H).

^{13}C NMR (101 MHz, $CDCl_3$) δ 169.8, 149.0, 139.6, 137.9, 128.9, 128.8, 127.9, 127.9, 123.0, 119.9, 97.9, 62.4, 47.2, 29.8, 29.8, 24.2, 21.7.

HRMS (ES+) exact mass calculated for $[M+Na]^+$ ($C_{21}H_{24}N_2ONa$) requires m/z 343.1781, found m/z 343.1789

5.6 Derivatizations and additional enamine reactivity

Compound 6a

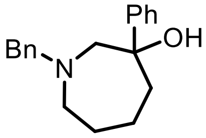

To a solution of aminoketone **3a** (25 mg, 0.123 mmol) in THF (1.23 mL), cooled to 0 °C, was added

PhMgBr (3 M in Et_2O, 62 μL, 0.185 mmol). The resulting solution was stirred for 2 hours before the addition of sat. aq. NH_4Cl. The resulting biphasic mixture was extracted with Et_2O (3 x 10 mL). The combined organics were dried with Na_2SO_4, filtered and concentrated under reduced pressure. The crude residue was purified by FCC (9:1 to 4:1 pentane:EtOAc) to yield the title compound as a colourless oil (29.1 mg, 84%).

IR (film) ν_{max}/cm^{-1} 3378, 2931

^{1}H NMR (400 MHz, $CDCl_3$) δ 7.49 – 7.41 (m, 2H), 7.39 – 7.26 (m, 7H), 7.26 – 7.21 (m, 1H), 5.11 (s, 1H), 3.80 (d, *J* = 13.0 Hz, 1H), 3.73 (d, *J* = 13.0 Hz, 1H), 3.18 (d, *J* = 13.4 Hz, 1H), 2.78 (tdd, *J* = 11.2, 7.1, 1.2 Hz, 1H), 2.63 (dt, *J* = 13.4, 1.6 Hz, 1H), 2.56 (ddd, *J* = 11.4, 7.2, 2.3 Hz, 1H), 2.02 – 1.90 (m, 1H), 1.90 – 1.81 (m, 1H), 1.81 – 1.71 (m, 2H), 1.73 – 1.54 (m, 2H).

^{13}C NMR (101 MHz, $CDCl_3$) δ 147.8, 138.7, 129.2, 128.6, 128.2, 127.5, 126.6, 124.5, 73.3, 64.4, 63.1, 53.3, 45.0, 28.2, 21.3.

HRMS (ES+) exact mass calculated for $[M+Na]^+$ ($C_{19}H_{23}NONa$) requires m/z 304.1672, found m/z 304.1677

Compound 6b

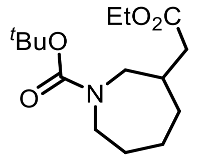

To a suspension of NaH (60% in paraffin oil, 11.8 mg, 0.296 mmol) in THF (2 mL), cooled to 0 °C, was added triethyl phosphonoacetate (59 μL, 0.30 mmol) and the mixture was stirred for 30 minutes. A solution of aminoketone **3a** (40 mg, 0.20 mmol) in THF (0.4 mL) was added and the mixture was stirred overnight at room temperature. H_2O was added and the resulting biphasic mixture was extracted with EtOAc (3 x 20 mL). The combined organics were dried with Na_2SO_4, filtered and concentrated under reduced pressure. The crude residue was purified by FCC (5:1 to 4:1 pentane:Et_2O) to yield the two geometric isomers of the acrylate product.

The mixture of isomers (34.2 mg, 0.125 mmol) was dissolved in EtOH (6.3 mL) and to this was added Pd/C (17 mg) and aq. HCl (1M, 0.25 mL). The solution was sparged with N_2 then sparged with H_2 for 5 minutes. The reaction was then stirred under H_2 atmosphere (double-skinned balloon pressure) for 5 hours. The mixture was passed through a celite plug and concentrated under reduced pressure. The resulting crude oil was dissolved in CH_2Cl_2 (2.5 mL) and Boc_2O (82 mg, 0.375 mmol) and Et_3N (56 μL, 0.375 mmol) were added. The solution was stirred for 2.5 hours and H_2O was added. The biphasic mixture was extracted with CH_2Cl_2 (3 x 20 ml) and the combined organics were dried with Na_2SO_4, filtered and concentrated

under reduced pressure. The crude residue was purified by FCC (3:1 to 2:1 pentane:EtOAc) to yield the title compound as a colourless oil (25.5 mg, 45% over 3 steps).

IR (film) ν_{max}/cm^{-1} 2978, 1736, 1694, 1168

^{1}H NMR (400 MHz, $CDCl_3$) δ 4.11 (qd, *J* = 7.2, 3.1 Hz, 2H), 3.62 – 3.47 (m, 2H), 3.37 (ddd, *J* = 14.1, 7.7, 4.7 Hz, 0H), 3.25 (tt, *J* = 13.8, 5.4 Hz, 1H), 2.96 (ddd, *J* = 27.1, 14.1, 8.6 Hz, 1H), 2.38 – 2.12 (m, 3H), 1.88 – 1.63 (m, 3H), 1.44 (d, *J* = 2.8 Hz, 11H), 1.24 (td, *J* = 7.1, 4.7 Hz, 4H).

^{13}C NMR (101 MHz, $CDCl_3$) δ 172.6, 155.8, 155.7, 79.4, 79.2, 60.4, 51.6, 51.4, 47.5, 47.3, 39.3, 39.3, 36.5, 36.3, 33.8, 32.8, 28.6, 28.3, 28.1, 25.3, 24.4, 14.4.

HRMS (ES+) exact mass calculated for $[M+H]^+$ ($C_{15}H_{28}NO_4$) requires m/z 286.2013, found m/z 286.2022

Compound 6c

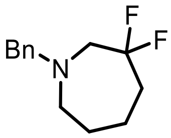

To a solution of aminoketone **3a** (40 mg, 0.20 mmol) in CH_2Cl_2 (0.67 mL), cooled to –78 °C by a dry ice/acetone bath, was added DeoxoFluor® (50% solution in THF, 212 µL, 0.50 mmol). The reaction mixture was allowed to come naturally to room temperature, with stirring, overnight. H_2O was added and the biphasic mixture was extracted with CH_2Cl_2 (3 x 20 mL). The combined organics were dried with Na_2SO_4, filtered and concentrated under reduced pressure. The crude residue was purified by FCC (10:1 to 5:1 pentane:EtOAc) to yield the title compound as a colourless oil (24.6 mg, 55%, 9.5 mg of aminoketone **3a** isolated).

IR (film) ν_{max}/cm^{-1} 2940, 1454, 1021

^{1}H NMR (400 MHz, $CDCl_3$) δ 7.39 – 7.22 (m, 5H), 3.75 (s, 2H), 3.03 (t, *J* = 14.1 Hz, 2H), 2.70 (t, *J* = 5.6 Hz, 2H), 2.15 (tdd, *J* = 16.2, 6.9, 3.6 Hz, 2H), 1.75 – 1.60 (m, 4H).

^{13}C NMR (101 MHz, $CDCl_3$) δ 139.2, 128.8, 128.4, 127.3, 125.9 (t, *J* = 241.7 Hz), 62.5 (t, *J* = 32.3), 56.8, 37.2 (t, *J* = 25.4 Hz), 30.0, 20.8 (t, *J* = 6.3 Hz).

^{19}F NMR (376 MHz, $CDCl_3$) δ -90.65.

HRMS (ES+) exact mass calculated for $[M+H]^+$ ($C_{13}H_{18}F_2N$) requires m/z 226.1402, found m/z 226.1405

Compound 6d

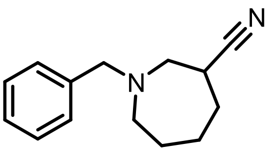

A solution of *p*-toluenesulfonylmethyl isocyanide (TosMIC, 36 mg, 0.18 mmol, 1.8 eq.) in DME (0.4 M)

was added to a solution of tBuOK (30 mg, 0.27 mmol, 2.7 eq.) in DME (1.6 M) at 0 °C. The resulting solution was stirred at 0 °C for 1 h and a solution of aminoketone **3a** (20 mg, 0.10 mmol, 1.0 eq.) in DME (0.45 M) was added. The resulting solution was allowed to warm up to room temperature and stirred for 18 h. Brine was added (5 mL) and the mixture was washed with EtOAc (3 x 5 mL). The combined organics were dried with Na_2SO_4, filtered and concentrated under reduced pressure. The crude residue was purified by FCC (pentane:EtOAc 9:1) to yield the title compound as a colorless oil (12.8 mg, 60%).

IR (film) ν_{max}/cm^{-1} 2938, 2237

^{1}H NMR (400 MHz, $CDCl_3$) δ 7.38 – 7.22 (m, 5H), 3.80 – 3.66 (m, 2H), 2.96 – 2.86 (m, 2H), 2.84 – 2.76 (m, 1H), 2.66 (ddd, *J* = 6.4, 5.3, 3.6 Hz, 2H), 2.01 – 1.87 (m, 2H), 1.85 – 1.77 (m, 1H), 1.73 – 1.61 (m, 3H).

^{13}C NMR (101 MHz, $CDCl_3$) δ 139.3, 128.9, 128.5, 127.3, 122.5, 62.9, 56.1, 55.3, 31.7, 31.7, 28.6, 23.9

HRMS (ES+) exact mass calculated for $[M+Na]^+$ ($C_{14}H_{18}N_2Na$) requires m/z 237.1362, found m/z 237.1371

Compound 6e

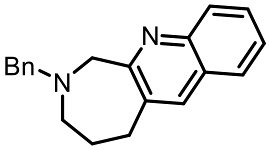

Following a modified literature procedure,[14] to a solution of aminoketone **3a** (40 mg, 0.20 mmol), 2-aminobenzaldehyde (24 mg, 0.20 mmol) in EtOH (2.0 mL) was added NaOH (10% w/w aq., 0.11 mL). The resulting solution was stirred for 30 hours then 1M HCl was added. Following no appearance of a desired precipitate, the solution was diluted with 1M NaOH and the pH was confirmed to be >10. The resulting aqueous phase was extracted with Et_2O and the combined organics were dried with Na_2SO_4, filtered and concentrated under reduced pressure. The crude residue was purified by FCC (1:1 to 2:3 pentane:Et_2O) to yield the title compound as a colourless oil (25 mg, 44%).

mp 84 – 90 °C

IR (film) ν_{max}/cm^{-1} 2927, 2842, 1621, 1568, 1494

^{1}H NMR (400 MHz, $CDCl_3$) δ 8.04 (dq, *J* = 8.4, 0.9 Hz, 1H), 7.87 (s, 1H), 7.75 (dd, *J* = 8.0, 1.5 Hz, 1H), 7.65 (ddd, *J* = 8.4, 6.9, 1.5 Hz, 1H), 7.50 (ddd, *J* = 8.1, 6.9, 1.2 Hz, 1H), 7.31 – 7.22 (m, 5H), 4.35 (s, 2H), 3.63 (s, 2H), 3.11 – 3.02 (m, 4H), 1.90 – 1.80 (m, 2H).

^{13}C NMR (101 MHz, $CDCl_3$) δ 161.6, 146.4, 139.2, 135.9, 134.7, 129.2, 129.0, 128.7, 128.3, 127.8, 127.1, 127.0, 126.4, 63.8, 58.2, 56.9, 35.0, 25.9.

HRMS (ES+) exact mass calculated for $[M+H]^+$ ($C_{20}H_{21}N_2$) requires m/z 289.1699, found m/z 289.1710

Compound 6f

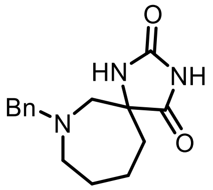

Following a modified literature procedure,[15] to a solution of KCN (19 mg, 0.30 mmol) and $NH_4(CO_3)_2$ (190 mg, 2.0 mmol) in H_2O (0.40 mL) was added a solution of aminoketone **3a** (40 mg, 0.20 mmol) in EtOH (0.26 mL). The mixture was heated to 60 °C and stirred for 16 hours. H_2O (2.4 mL) was added and the aqueous layer was extracted with EtOAc (3 x 20 mL). The combined organics were washed with brine (10 mL), dried with Na_2SO_4, filtered and concentrated under reduced pressure to yield the title compound as a white solid (49 mg, 91%).

mp 155-160 °C

IR (film) ν_{max}/cm^{-1} 3253, 1769, 1719

^{1}H NMR (400 MHz, $CDCl_3$) δ 8.88 (s, 1H), 7.36 – 7.21 (m, 5H), 6.33 (s, 1H), 3.73 (d, *J* = 13.1 Hz, 1H), 3.61 (d, *J* = 13.1 Hz, 1H), 3.00 (d, *J* = 13.8 Hz, 1H), 2.86 (dtd, *J* = 11.9, 6.8, 3.4 Hz, 1H), 2.73 (dt, *J* = 13.8, 1.4 Hz, 1H), 2.54 (ddd, *J* = 11.4, 6.0, 4.9 Hz, 1H), 2.12 – 1.54 (m, 6H).

^{13}C NMR (101 MHz, $CDCl_3$) δ 177.2, 156.7, 138.7, 129.1, 128.7, 127.6, 66.2, 63.7, 58.8, 55.3, 38.1, 28.8, 21.7.

HRMS (ES+) exact mass calculated for $[M+H]^+$ ($C_{15}H_{20}N_3O_2$) requires m/z 274.1560, found m/z 274.1550

Compound S1-5

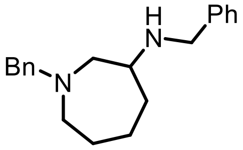

To a solution of aminoketone 3a (39.1 mg, 0.19 mmol) in CH_2Cl_2 (0.64 mL) was added $BnNH_2$ (21 µL, 0.19 mmol), AcOH (0.22 µL, 0.38 mmol) and $NaBH(OAc)_3$ (102 mg, 0.48 mmol). The resulting mixture was stirred at room temperature overnight. Sat. aq. K_2CO_3 was added and the mixture was diluted with CH_2Cl_2. The biphasic mixture was separated and the aqueous phase was extracted with CH_2Cl_2 (3 x 20 mL). The combined organics were dried with Na_2SO_4, filtered and concentrated under reduced pressure. The crude residue was purified by FCC (1:1 to 1:0 pentane:Et_2O, 1% Et_3N) to yield the title compound as a colourless oil (47.7 mg, 85%).

IR (film) ν_{max}/cm^{-1} 2927, 1494

^{1}H NMR (400 MHz, $CDCl_3$) δ 7.41 – 7.17 (m, 10H), 3.66 (d, *J* = 1.5 Hz, 2H), 3.56 (s, 2H), 2.78 – 2.56 (m, 5H), 1.85 (ddt, *J* = 13.8, 11.6, 4.9 Hz, 1H), 1.76 – 1.62 (m, 3H), 1.62 – 1.32 (m, 2H).

^{13}C NMR (101 MHz, $CDCl_3$) δ 141.0, 140.3, 129.0, 128.4, 128.3, 128.1, 127.0, 126.8, 63.8, 58.8, 57.1, 56.8, 51.2, 35.0, 29.3, 22.9.

HRMS (ES+) exact mass calculated for $[M+Na]^+$ ($C_{20}H_{26}N_2Na$) requires m/z 317.2001, found m/z 317.1988

Compound 6g

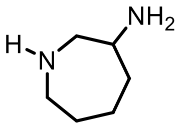

To a solution of **S1-5** (29.4 mg, 0.10 mmol) in EtOH (4 mL) was added Pd/C (15 mg). The resulting suspension was sparged with H_2 gas for 5 minutes and then stirred under a H_2 atmosphere for 16 hours. The reaction mixture was passed through a Celite® plug and the solvents were removed under reduced pressure to yield the title compound as a colourless oil (9.5 mg, 83%).

^{1}H NMR (400 MHz, MeOD) δ 3.22 (tdd, J = 7.4, 4.8, 3.5 Hz, 1H), 3.08 (ddd, J = 13.9, 3.7, 0.7 Hz, 1H), 2.98 – 2.88 (m, 2H), 2.81 (dd, J = 13.9, 7.1 Hz, 1H), 2.02 – 1.91 (m, 1H), 1.83 – 1.70 (m, 3H), 1.66 – 1.54 (m, 2H).

^{13}C NMR (101 MHz, MeOD) δ 52.9, 52.5, 49.3, 35.0, 29.9, 23.0.

Compound 1b

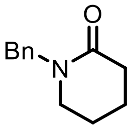

Following a modified literature procedure,[16] to a microwave vial was added amino ketone **3a** (40 mg, 0.20 mmol) and PhMe (4 mL). The vial was sealed and the resulting solution was degassed via a stream of argon for 5 minutes before addition of TBHP (110 µL, 5.5 M in decane, 0.59 mmol). The mixture was stirred at 90 °C for 3 hours then cooled to room temperature. H_2O was added and the biphasic mixture was extracted with EtOAc (3 x 20 mL). The combined organics were dried with Na_2SO_4, filtered and concentrated under reduced pressure. The crude residue was purified by FCC (4:1 pentane:Et_2O) to yield the title compound as a colourless oil (17.1 mg, 46%). All data were in agreement with those obtained previously in this work.

One-pot procedure from lactam 1a:

To a 10 mL vial equipped with a magnetic stirring bar was added amide **1a** (0.1 mmol) and Vaska's catalyst (1.5 mol%, 1.2 mg). The vial was capped with a septum and evacuated and backfilled with N_2 three times. The N_2 line was then exchanged for a N_2 balloon. CH_2Cl_2 (83 mM, 1.2 mL), DIPEA (1.2 eq., 21 µL, 0.12 mmol) and TMDS (1.5 eq., 26 µL, 0.15 mmol) were consecutively added. The reaction mixture was stirred at room temperature for 15 minutes and then cooled to –78 °C. After 5 minutes at this temperature, a

solution of hydrogen peroxide (~30 % in water, 6 eq., 61 µL, 0.6 mmol) and purified mCPBA[3] (1.1 equiv., 19 mg, 0.11 mmol) in CH_2Cl_2 (1.2 mL) was added and the vial was removed from the cooling bath and stirred at 60 °C for 6 hours. The reaction mixture was then diluted with CH_2Cl_2 and sat. aq. $NaHCO_3$ was added and the aqueous layer is extracted with CH_2Cl_2 (3 x 20 mL). The combined organics were dried with Na_2SO_4, filtered and concentrated under reduced pressure. The NMR yield was determined to be 33% by ^1H quantitative NMR using 1,2,4,5-tetramethylbenzene as internal standard.

Compound 4a

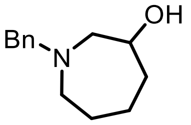

To a solution of lactam **1a** (41 mg, 0.20 mmol) in CH_2Cl_2 (2.4 mL) was added Vaska's catalyst (1.5 mol%, 2.4 mg), DIPEA (41.6 µL, 0.24 mmol) and then TMDS (52.4 µL, 0.30 mmol). The resulting solution was stirred for 15 minutes before addition to a solution of $Thex_2BH$ in THF (0.44 mmol, 0.11 M).[17] Then, following a modified literature procedure,[17] the mixture was stirred for 2 hours at 0 °C, MeOH was added (200 µL). Following stirring for 1 hour, the mixture was returned to 0 °C and NaOH (54 mg, 0.6 mmol) was added followed by H_2O_2 (34% aq, 50 µL, 0.48 mmol). The mixture was stirred at reflux for 1 hour, cooled to room temperature and diluted with H_2O. The aqueous phase was extracted with Et_2O (5 x 15 mL) and the combined organics were dried with Na_2SO_4, filtered and concentrated under reduced pressure. The resulting crude residue was purified by FCC (1:0 to 19:1 EtOAc:MeOH) to yield the title compound as a colourless oil (26 mg, 64%). All data were in agreement with those obtained previously in this work.

Compound 6h

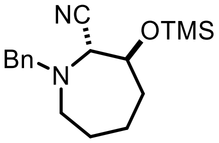

As a modification to General Procedure 4, lactam **1a** (41 mg, 0.20 mmol) was submitted to the standard conditions however, following addition of the *m*CPBA, the reaction was stirred at –78 °C for 10 minutes. TMSCN (100 µL, 0.80 mmol) was added and the reaction was warmed to –20 °C. The reaction was stirred at this temperature for 16 hours and then passed through a silica plug (eluting with Et_2O). The volatiles were removed under reduced pressure and the crude residue was purified by FCC (1:0 to 10:1 pentane:Et_2O, eluent kept at 0 °C) to yield the title compound as a colourless oil (19 mg, 31%).

IR (film) ν_{max}/cm^{-1} 2949, 1253, 842

^{1}H NMR (400 MHz, $CDCl_3$) δ 7.34 – 7.15 (m, 5H), 3.91 (ddd, *J* = 9.3, 7.1, 1.9 Hz, 1H), 3.73 (s, 2H), 3.50 (dd, *J* = 7.0, 0.8 Hz, 1H), 2.69 – 2.54 (m, 2H), 2.03 (dddd, *J* = 13.7, 11.1, 9.5, 2.5 Hz, 1H), 1.85 – 1.72 (m,

1H), 1.61 (ddt, J = 13.8, 6.8, 2.5 Hz, 1H), 1.54 – 1.43 (m, 2H), 1.43 – 1.26 (m, 1H), 0.07 (s, 9H).

^{13}C NMR (101 MHz, $CDCl_3$) δ 138.1, 129.1, 128.6, 127.7, 119.1, 76.3, 63.2, 60.8, 50.9, 34.7, 29.6, 24.6, 0.1.

HRMS (ES+) exact mass calculated for $[M+H]^+$ ($C_{17}H_{27}N_2OSi$) requires m/z 303.1887, found m/z 303.1901

Note: In order to obtain clean analytical data, purification was conducted with cooled (~ 0 °C) eluent. It was observed that if care was not taken in purification or if fewer eq. of TMSCN were used, the aminonitrile 6h was labile to desilylation and epimerization at the aminonitrile stereocentre, resulting in isolation of a mixture of diastereomeric β-hydroxy-α-aminonitriles. Prolonged purification or repeated attempts resulted in conversion to the aminoketone 3a.

Compound 6i

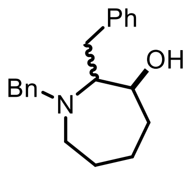

As a modification to General Procedure 4, lactam **1a** (41 mg, 0.20 mmol) was submitted to the standard conditions however, following addition of the mCPBA, the reaction was stirred at –78 °C for 10 minutes. BnMgCl (0.50 mL, 1.0 mmol) was added and the reaction allowed to warm naturally to room temperature. The reaction was stirred for 4 hours and worked up in the manner outlined in the general procedure. The crude residue was purified by FCC (3:1 to 0:1 pentane:Et_2O) to yield a diastereomeric mixture of the desired product as a colourless oil (32 mg, 53%, 2.6:1 dr).

IR (film) ν_{max}/cm^{-1} 3421, 2928, 1453

^{1}H NMR (400 MHz, $CDCl_3$) δ 7.24 – 7.01 (m, 10H), 3.76 – 3.65 (m, 1H), 3.61 (d, J = 13.2 Hz, 1H), 3.53 (d, J = 13.2 Hz, 1H), 3.44 (td, J = 4.4, 2.6 Hz, 1H), 3.12 – 3.02 (m, 1H), 2.95 – 2.82 (m, 1H), 2.80 – 2.41 (m, 3H), 1.80 – 1.27 (m, 5H).

^{13}C NMR (101 MHz, $CDCl_3$) δ 140.9, 140.1, 139.6, 129.3, 129.0, 128.9, 128.6, 128.5, 128.4, 128.4, 128.2, 127.3, 126.7, 126.2, 126.0, 72.0, 69.4, 69.0, 66.6, 61.9, 56.3, 51.0, 49.9, 35.4, 35.1, 33.9, 33.6, 28.3, 27.3, 21.3, 20.4.

HRMS (ES+) exact mass calculated for $[M+H]^+$ ($C_{20}H_{26}NO$) requires m/z 296.2009, found m/z 296.2013

Compound 6j

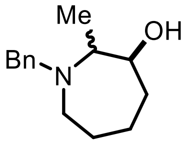

As a modification to General Procedure 4, lactam **1a** (41 mg, 0.20 mmol) was submitted to the standard conditions however, following addition of the mCPBA, the reaction was stirred at –78 °C for 10 minutes. $AlMe_3$ (0.10 mL, 2M in PhMe, 0.20 mmol) was added and the reaction allowed to warm naturally to room temperature. The reaction was stirred for 6 hours. Sat. aq. $NaHCO_3$ was added and the biphasic mixture was stirred vigorously for 10 minutes. The biphasic mixture was extracted with CH_2Cl_2 (3 x 20 mL). The crude residue was purified by FCC (2:1 to 1:1 pentane:Et_2O, 1% Et_3N) to yield the title compounds as colourless oils (A: 10.0 mg, B: 12.1 mg; 50%).

A:

IR (film) ν_{max}/cm^{-1} 3416, 2929, 1033

^{1}H NMR (400 MHz, $CDCl_3$) δ 7.37 – 7.22 (m, 5H), 3.74 – 3.67 (m, 2H), 3.64 (s, 1H), 3.57 (td, *J* = 4.6, 2.8 Hz, 1H), 3.12 – 3.03 (m, 1H), 2.69 – 2.64 (m, 1H), 2.61 (ddd, *J* = 12.7, 6.5, 4.2 Hz, 1H), 1.87 (dddd, *J* = 13.8, 6.9, 3.5, 1.8 Hz, 1H), 1.82 – 1.70 (m, 2H), 1.68 – 1.53 (m, 2H), 1.53 – 1.47 (m, 1H), 1.09 (d, *J* = 7.1 Hz, 3H).

^{13}C NMR (101 MHz, $CDCl_3$) δ 139.9, 129.0, 128.6, 127.3, 72.3, 62.1, 61.7, 49.5, 33.6, 28.5, 20.8, 10.9.

HRMS (ES+) exact mass calculated for $[M+H]^+$ ($C_{14}H_{22}NO$) requires m/z 220.1696, found m/z 220.1697

B:

IR (film) ν_{max}/cm^{-1} 3397, 2930, 1453, 1038

^{1}H NMR (400 MHz, $CDCl_3$) δ 7.34 – 7.29 (m, 4H), 7.23 (ddt, *J* = 6.4, 3.6, 2.3 Hz, 1H), 3.91 (d, *J* = 13.6 Hz, 1H), 3.76 (ddd, *J* = 7.1, 3.9, 2.3 Hz, 1H), 3.60 (d, *J* = 13.6 Hz, 1H), 3.06 (qd, *J* = 7.1, 2.3 Hz, 1H), 2.71 (ddd, *J* = 13.0, 7.6, 5.7 Hz, 1H), 2.50 (dt, *J* = 12.3, 5.9 Hz, 1H), 1.78 – 1.72 (m, 1H), 1.71 – 1.52 (m, 3H), 1.21 (d, *J* = 7.0 Hz, 3H).

^{13}C NMR (101 MHz, $CDCl_3$) δ 140.9, 128.5, 128.4, 126.9, 74.1, 59.7, 57.9, 51.7, 35.4, 27.9, 21.1, 13.8.

HRMS (ES+) exact mass calculated for $[M+Na]^+$ ($C_{14}H_{21}NONa$) requires m/z 242.1515, found m/z 242.1505

6. Reaction Troubleshooting and further comments

Q. Do I need to handle Vaska's complex and TMDS under an inert gas atmosphere?

A. No, Vaska's complex is stable at r.t. for prolonged periods with no special precautions taken. Faint decolouration is observed after a period of months if a flask is in regular use. No observable

loss in reactivity is observed for TMDS stored as supplied at room temperature and handled with no special precautions.

Q. I have attempted the carbonyl transposition reaction and obtained only starting material. Why is my reduction not working?

A. This tends to be very unusual given how effective Vaska's complex is at the reduction of tertiary amides. Note that under the conditions given in this study, Vaska's complex is unable to reduce primary or secondary amides. We have found that some substrates containing acidic impurities, resulting from their synthesis or storage, can hamper the efficiency of the reduction. In some cases, this can be rectified by performing a silica plug following by a basic (sat. aq. $NaHCO_3$) wash of an Et_2O solution of the substrate. Additionally, for every acidic X-H in the substrate (e.g -OH, -CO_2H, -NH) increase the equivalents of TMDS (one equivalent for each acidic X-H). These substrates may also require increasing the catalyst loading.

Q. I have attempted the carbonyl transposition reaction and I have obtained a complex mixture in my crude.

A.

- Monitor the reduction by ^1H NMR, utilising CD_2Cl_2, easily performed on 0.05 mmol. This can highlight a slow reduction phase or slow elimination to the enamine. Both of these can easily be resolved by prolonging both stages of the reaction.
- An additional sign that this could be your problem is observation of the "over-reduced" amine product – it is proposed that this results from the silylated hemi-aminal species remaining in the reaction mixture for the overnight stage and undergoing reduction.
- Some aminoketones are unstable in the reaction conditions. We found that for N-benzyl piperidine substrates, yields could be uplifted by switching the transposition phase to stirring for 2 h in a pH 5 aq. acetate buffer (see 3b and 3n) rather than stirring at room temperature overnight.
- Given the instability of some aminoketones, it could be worth attempting the reductive transposition ($LiAlH_4$ quench) - if the amino-alcohol is desirable - as this has shown to give higher yields and easier purification in some cases.

Other comments:

- For purification of acyclic enaminones, slurrying the silica with 1% Et_3N and using eluent containing 1% Et_3N dramatically uplifted the yield and avoided observation of the resulting

hydrolysis product.

- Purified mCPBA: CAUTION, 95-100% mCPBA has been shown to be detonated by shocks or sparked. Store in a freezer, well-sealed. Handle with care.[3]
- Aminoketones are often unstable to decomposition in air, particularly the 6-membered and acyclic series, and should be stored in a N_2 purged vial at –20 °C.

7. ESI References

(1) Lee, S.; Kang, G.; Chung, G.; Kim, D.; Lee, H. Y.; Han, S. Biosynthetically Inspired Syntheses of Secu′amamine A and Fluvirosaones A and B. *Angew. Chemie - Int. Ed.* **2020**, *59* (17), 6894–6901.

(2) Plaza, M.; Jandl, C.; Bach, T. Photochemical Deracemization of Allenes and Subsequent Chirality Transfer. *Angew. Chemie Int. Ed.* **2020**, *59* (31), 12785–12788.

(3) Horn, A.; Kazmaier, U. Purified MCPBA, a Useful Reagent for the Oxidation of Aldehydes. *European J. Org. Chem.* **2018**, *2018* (20), 2531–2536.

(4) Li, D. H.; Gamage, R. S.; Smith, B. D. Sterically Shielded Hydrophilic Analogs of Indocyanine Green. *J. Org. Chem.* **2022**, *87* (17), 11593–11601.

(5) Chen, M.; Dong, G. Direct Catalytic Desaturation of Lactams Enabled by Soft Enolization. *J. Am. Chem. Soc.* **2017**, *139* (23), 7757–7760.

(6) Lavinda, O.; Witt, C. H.; Woerpel, K. A. Origin of High Diastereoselectivity in Reactions of Seven-Membered-Ring Enolates. *Angew. Chemie - Int. Ed.* **2022**, *61* (14) https://doi.org/10.1002/anie.202114183

(7) Xiong, B.; Zhu, L.; Feng, X.; Lei, J.; Chen, T.; Zhou, Y.; Han, L. B.; Au, C. T.; Yin, S. F. Direct Amidation of Carboxylic Acids with Tertiary Amines: Amide Formation over Copper Catalysts through C–N Bond Cleavage. *European J. Org. Chem.* **2014**, *2014* (20), 4244–4247.

(8) Annadi, K.; Wee, A. G. H. An Alkylidene Carbene C-H Activation Approach toward the Enantioselective Syntheses of Spirolactams: Application to the Synthesis of (-)-Adalinine. *J. Org. Chem.* **2016**, *81* (3), 1021–1038.

(9) Fuentes de Arriba, Á. L.; Lenci, E.; Sonawane, M.; Formery, O.; Dixon, D. J. Iridium-Catalyzed Reductive Strecker Reaction for Late-Stage Amide and Lactam Cyanation. *Angew. Chemie Int. Ed.* **2017**, *56* (13), 3655–3659.

(10) Hwang, Y. C.; Chu, M.; Fowler, F. W. Synthesis of α-Substituted Amines. *J. Org. Chem.* **1986**, *51* (11), 2156.

(11) Huang, W. X.; Wu, B.; Gao, X.; Chen, M. W.; Wang, B.; Zhou, Y. G. Iridium-Catalyzed Selective Hydrogenation of 3-Hydroxypyridinium Salts: A Facile Synthesis of Piperidin-3-Ones. *Org. Lett.* **2015**, *17* (7), 1640–1643.

(12) Soheili, A.; Tambar, U. K. Tandem Catalytic Allylic Amination and [2,3]-Stevens Rearrangement of Tertiary Amines. *J. Am. Chem. Soc.* **2011**, *133* (33), 12956–12959.

(13) Moody, C. J.; Lightfoot, A. P.; Gallagher, P. T. Asymmetric Synthesis of 2-Substituted Piperidines. Synthesis of the Alkaloids (-)-Coniine and (+)-Pseudoconhydrine. *J. Org. Chem.* **1997**, *62* (3), 746–748.

(14) Kempter, G.; Hirschberg, S. Heterocyclen Aus Aminoketonen, V. Über Die Friedländer-Synthese Mit N, O Bzw. S

Enthaltenden Fünf- Und Sechsringketonen. *Chem. Ber.* **1965**, *98* (2), 419–427.

(15) Yan, N. L.; Santos-Martins, D.; Nair, R.; Chu, A.; Wilson, I. A.; Johnson, K. A.; Forli, S.; Morgan, G. J.; Petrassi, H. M.; Kelly, J. W. Discovery of Potent Coumarin-Based Kinetic Stabilizers of Amyloidogenic Immunoglobulin Light Chains Using Structure-Based Design. *J. Med. Chem.* **2021**, *64* (9), 6273–6299.

(16) Peng, X.; Wang, H. H.; Cao, F.; Zhang, H. H.; Lu, Y. M.; Hu, X. L.; Tan, W.; Wang, Z. TBHP Promoted Demethylation of α-Amino Carbonyl Compounds: A Concise Approach to Substituted γ-Lactams. *Org. Chem. Front.* **2019**, *6* (11), 1837–1841..

(17) Fisher, G. B.; Goralski, C. T.; Nicholson, L. W.; Hasha, D. L.; Zakett, D.; Singaram, B. Boranes in Synthesis. 5. The Hydroboration of Enamines with Mono- and Dialkylboranes. Asymmetric Synthesis of F-Amino Alcohols of Moderate Enantiomeric Purity from Aldehyde Enamines. *J. Org. Chem.* **1995**, *60* (7), 2026–2034.

8. NMR Spectra

Compound 1e (^{1}H)

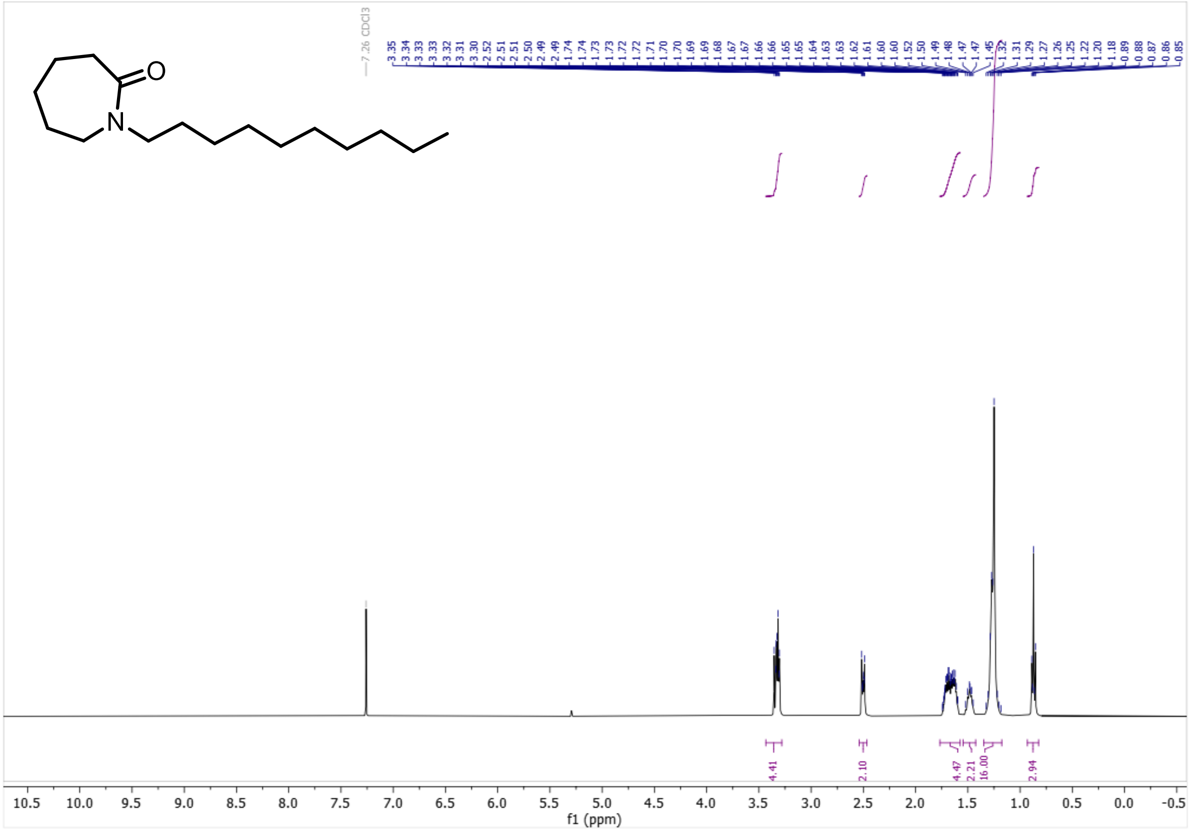

Compound 1e (^{13}C)

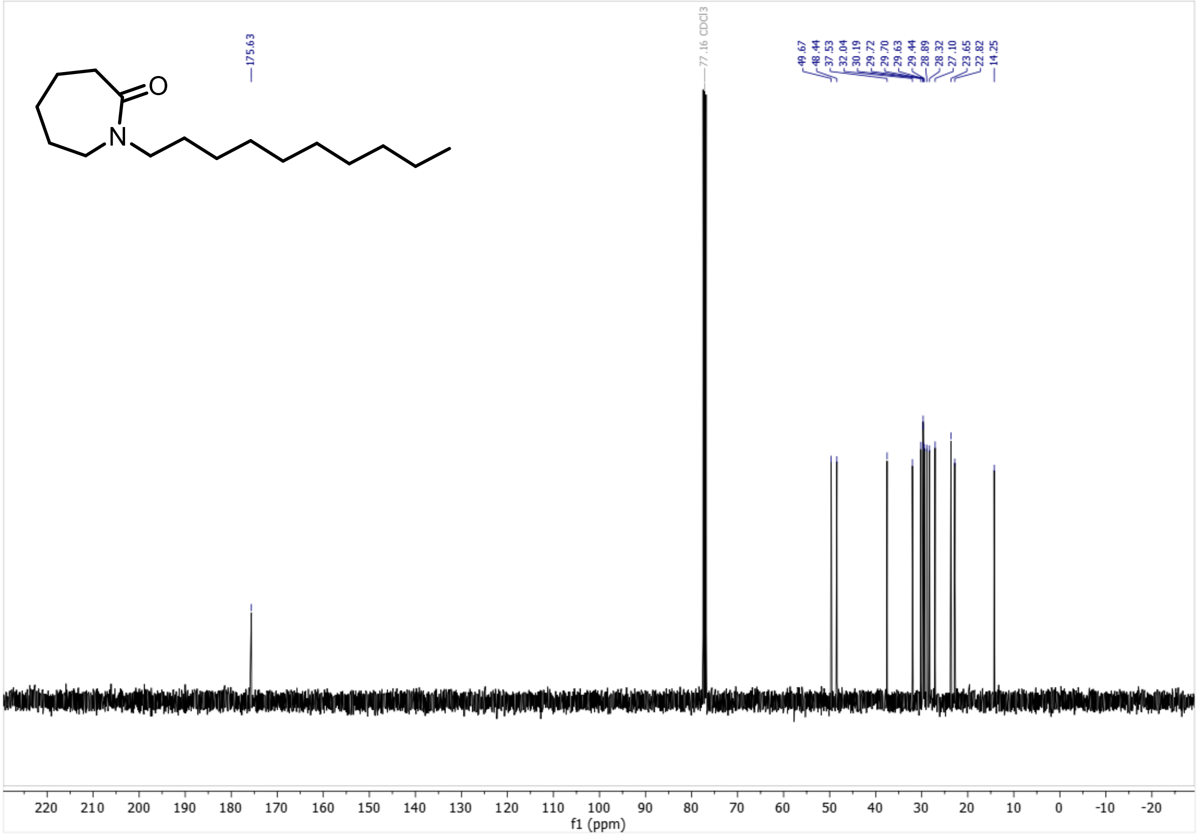

Compound 1f (^{1}H)

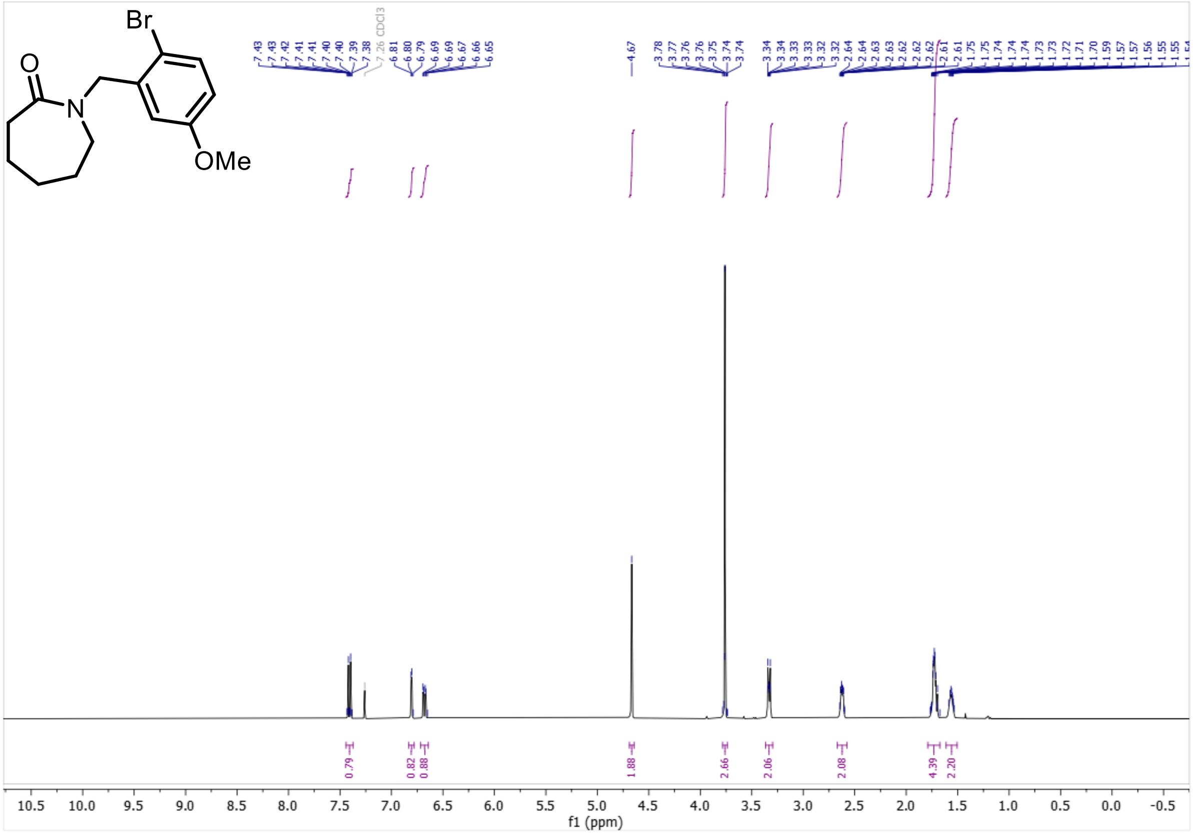

Compound 1f (^{13}C)

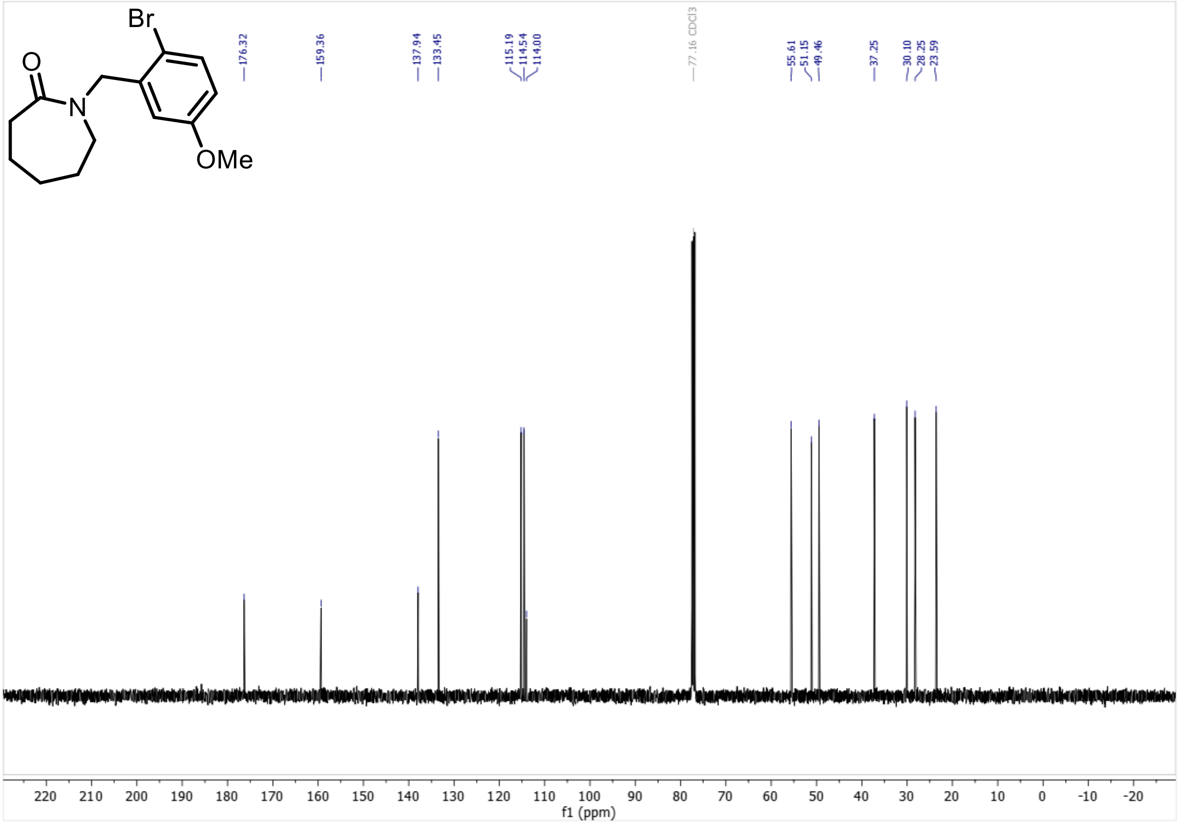

Compound 1g (^{1}H)

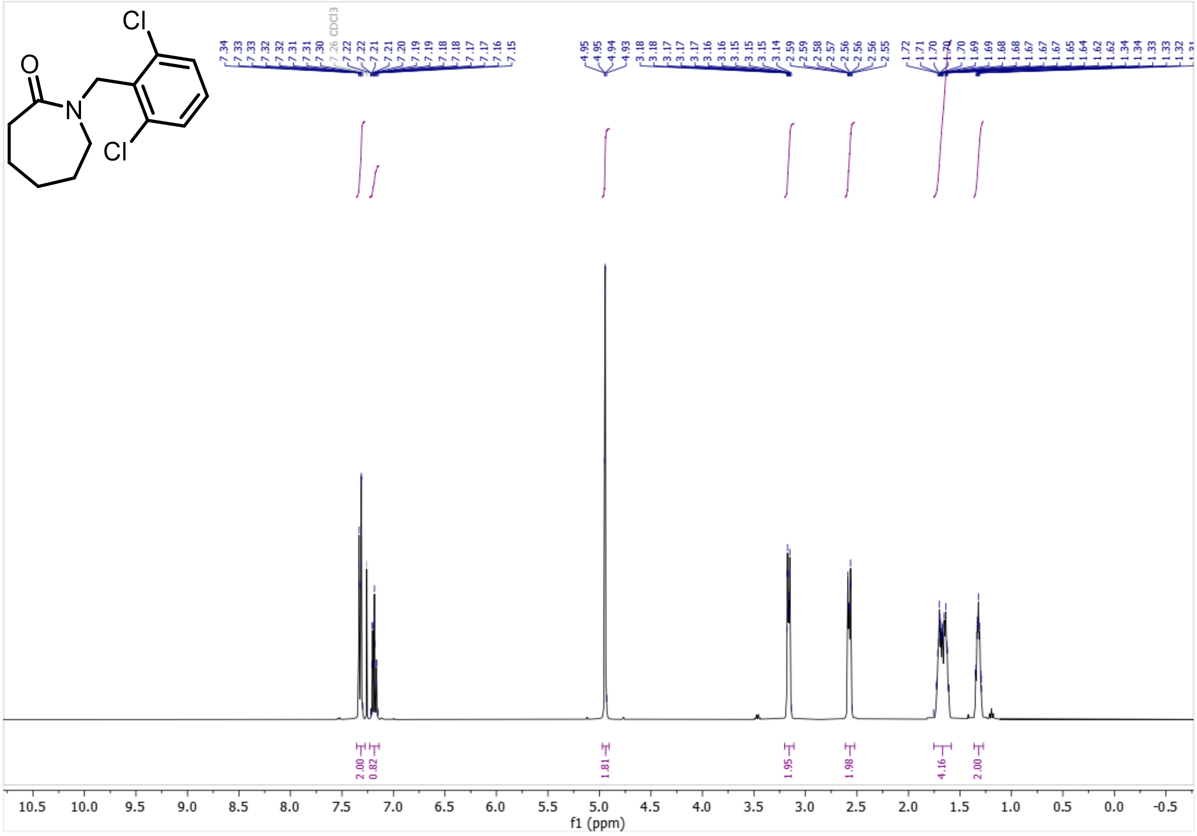

Compound 1g (^{13}C)

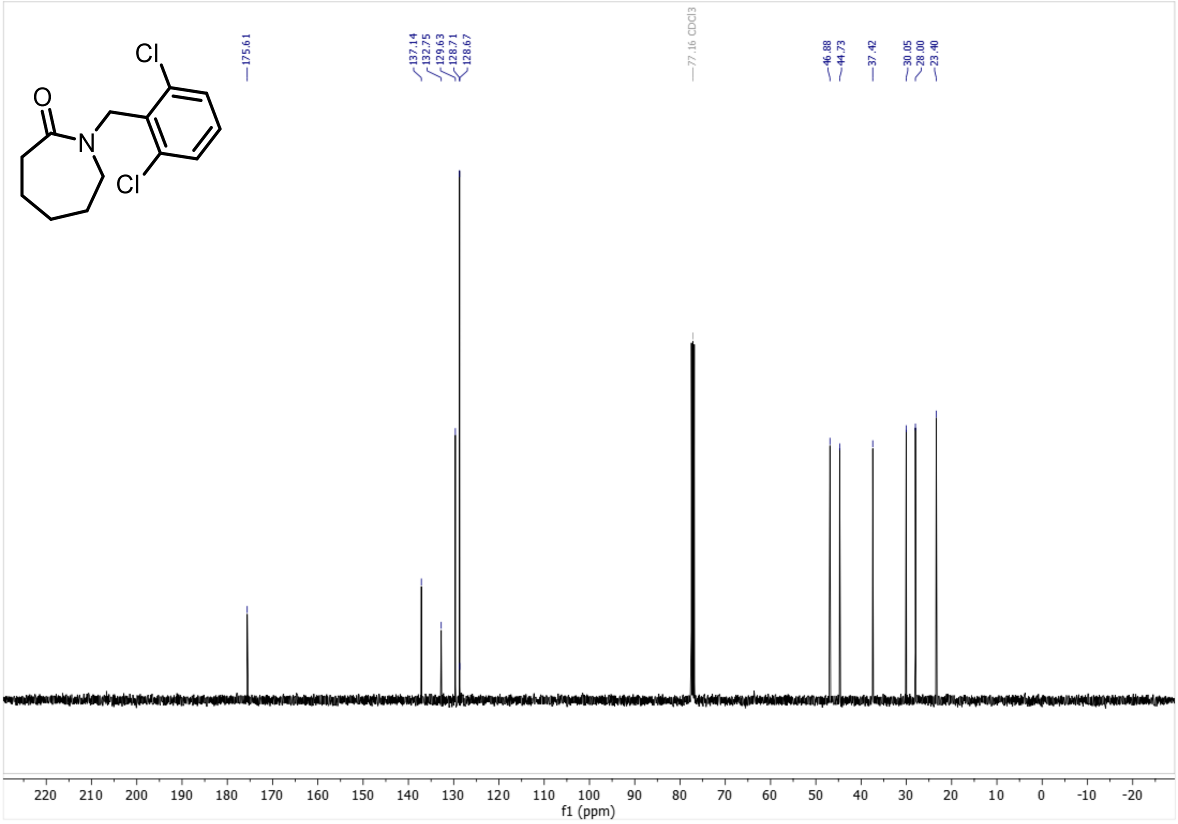

Compound 1i (^{1}H)

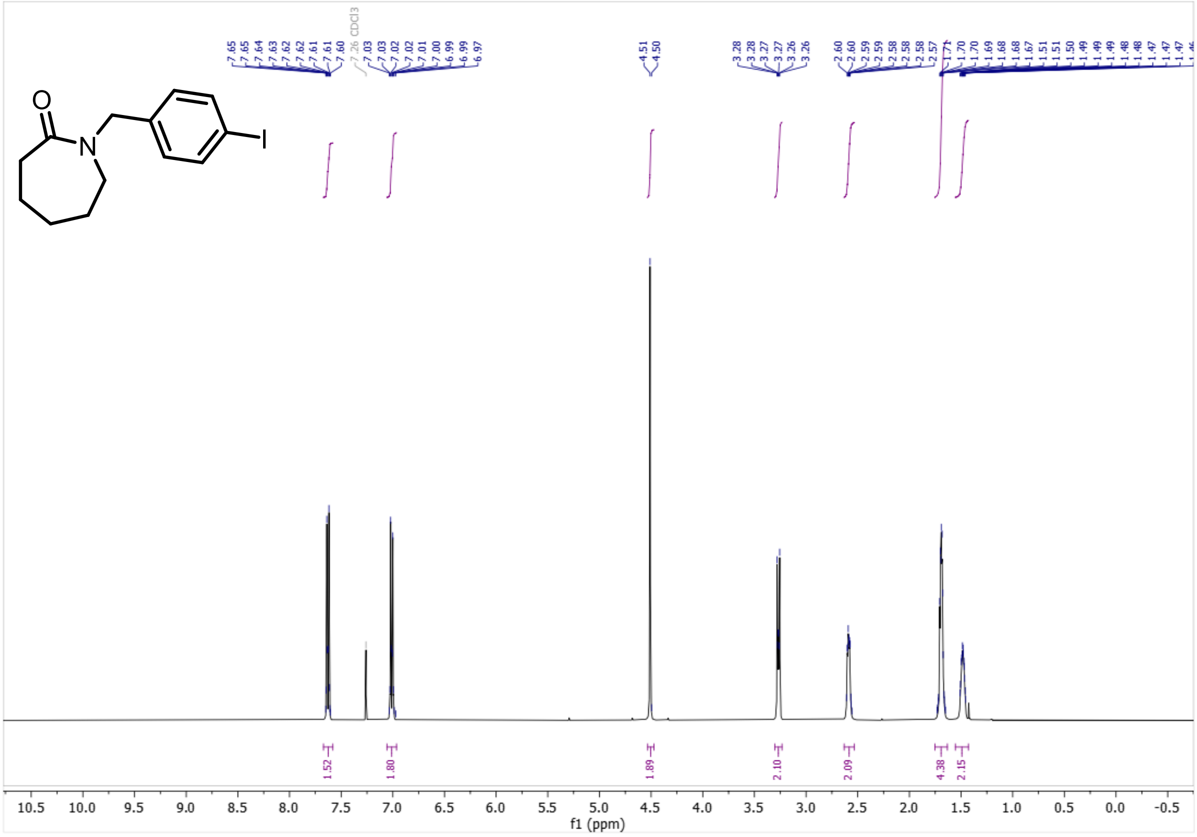

Compound 1i (^{13}C)

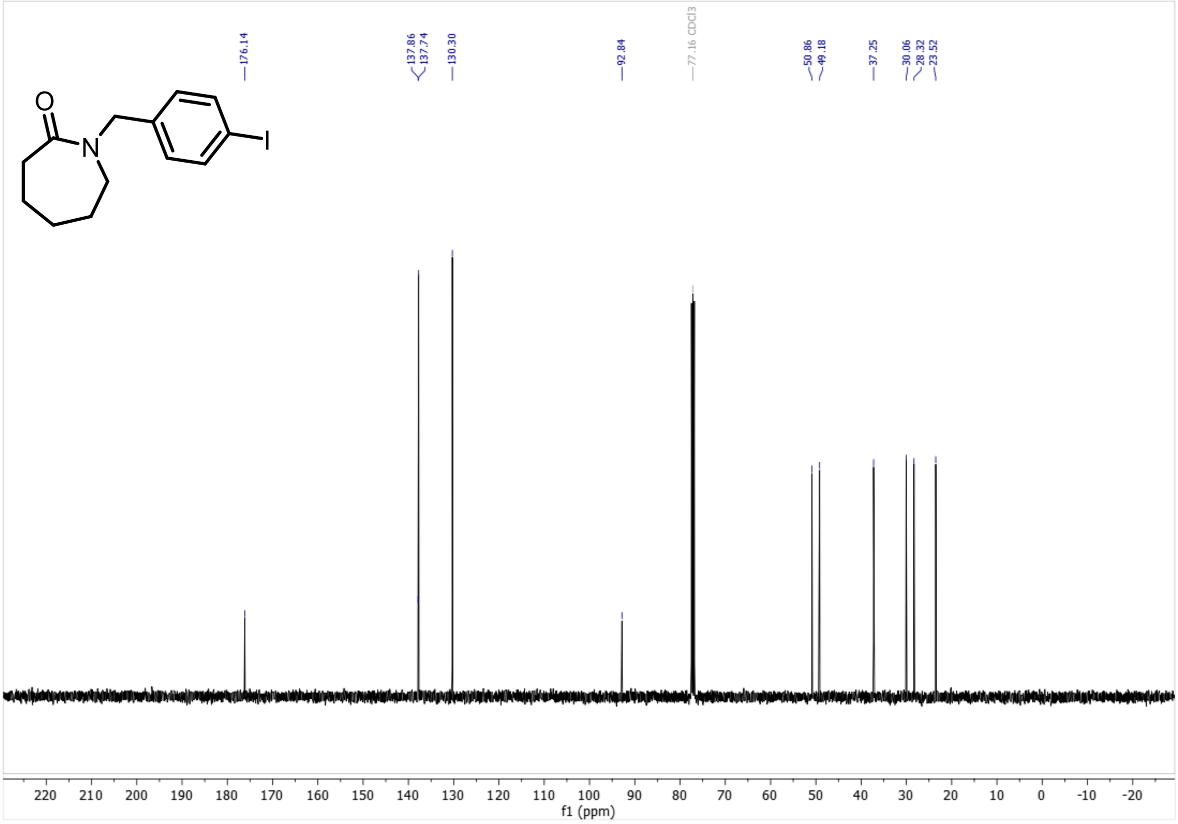

Compound 1j (^{1}H)

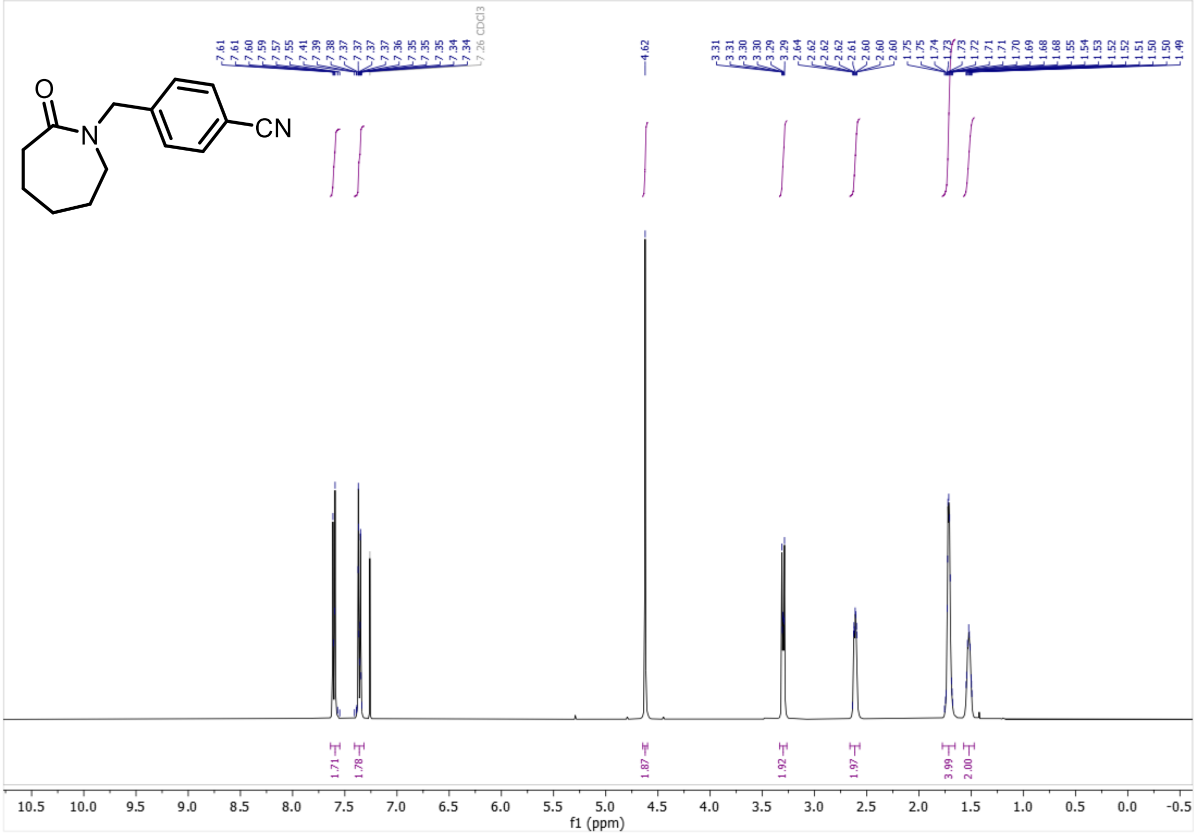

Compound 1j (^{13}C)

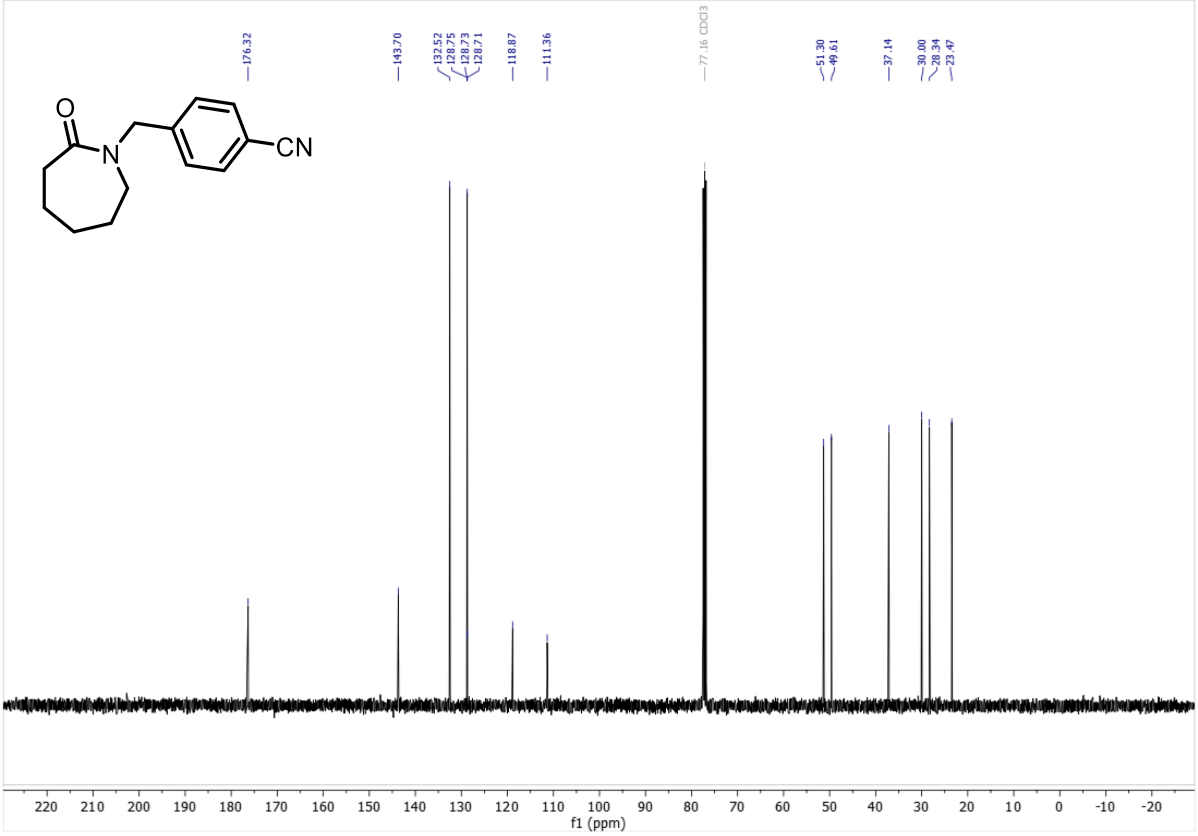

Compound S1-1 (^{1}H)

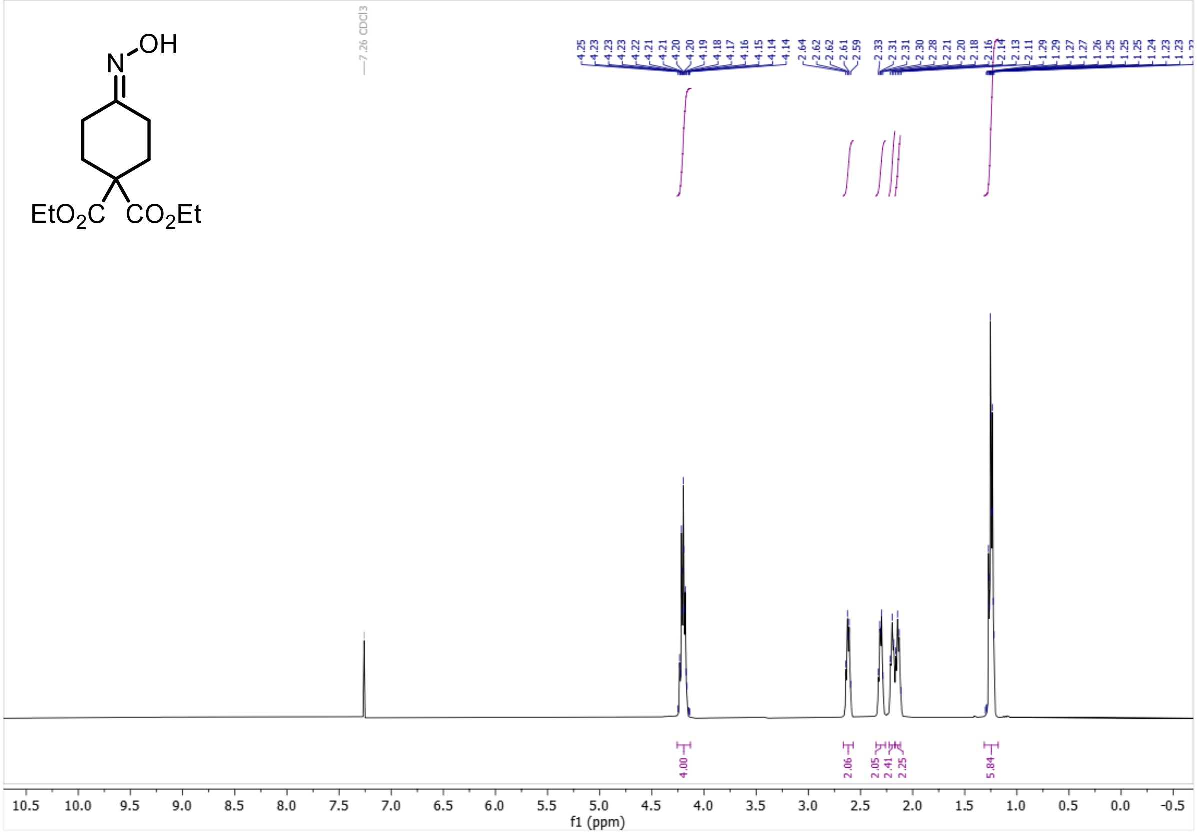

Compound S1-1 (^{13}C)

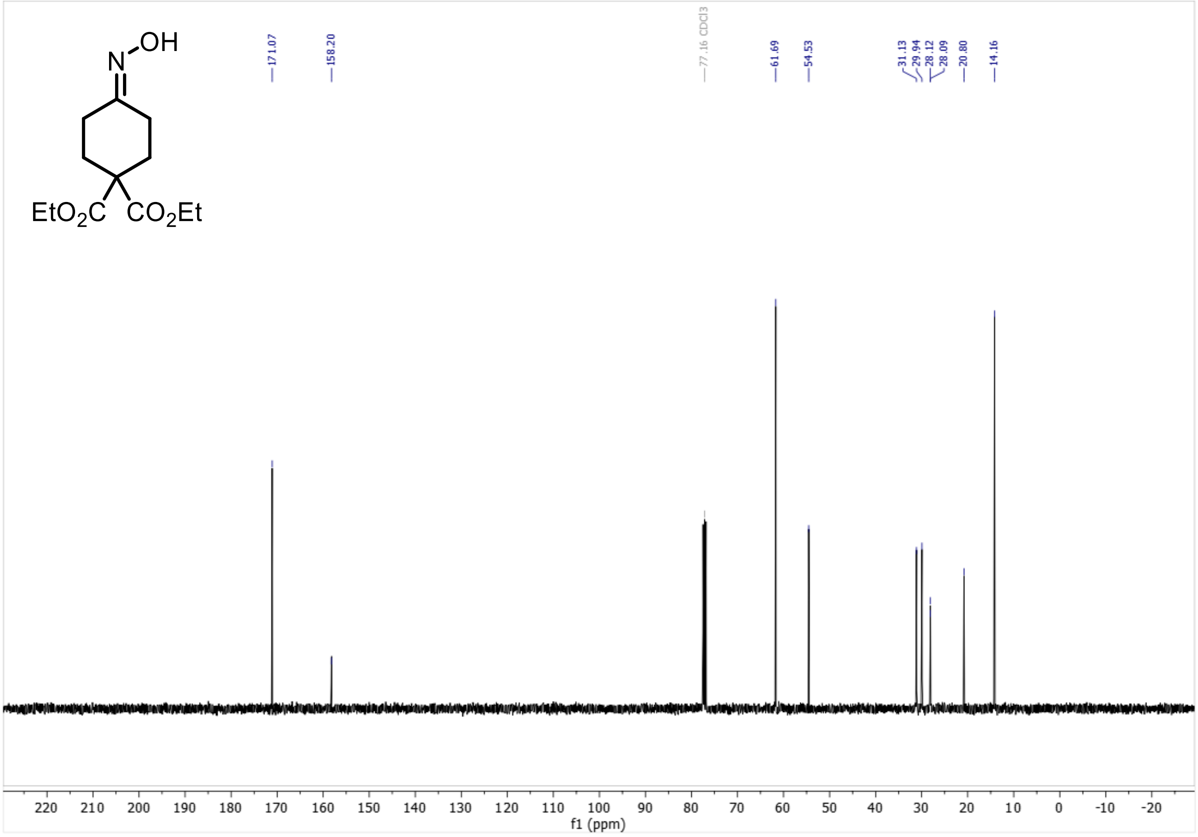

Compound S1-2 (¹H)

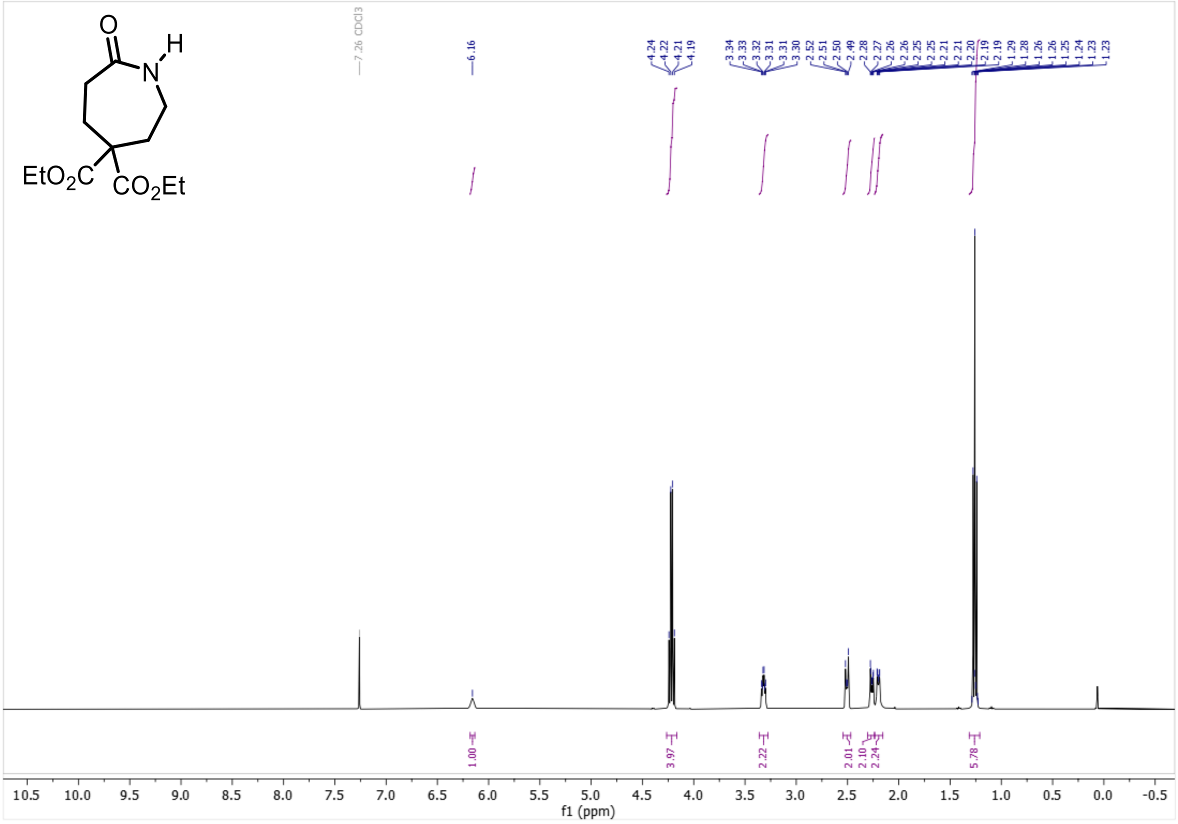

Compound S1-2 (¹³C)

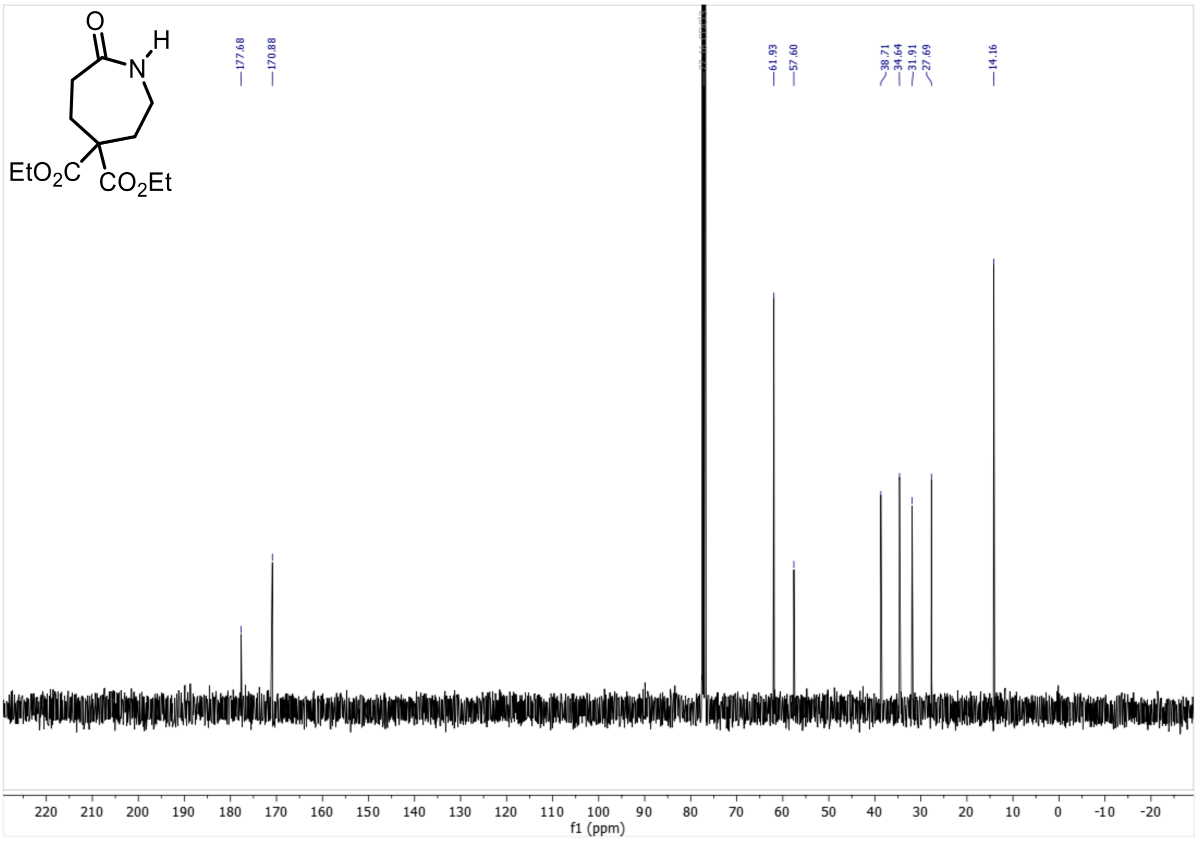

Compound 1k (^{1}H)

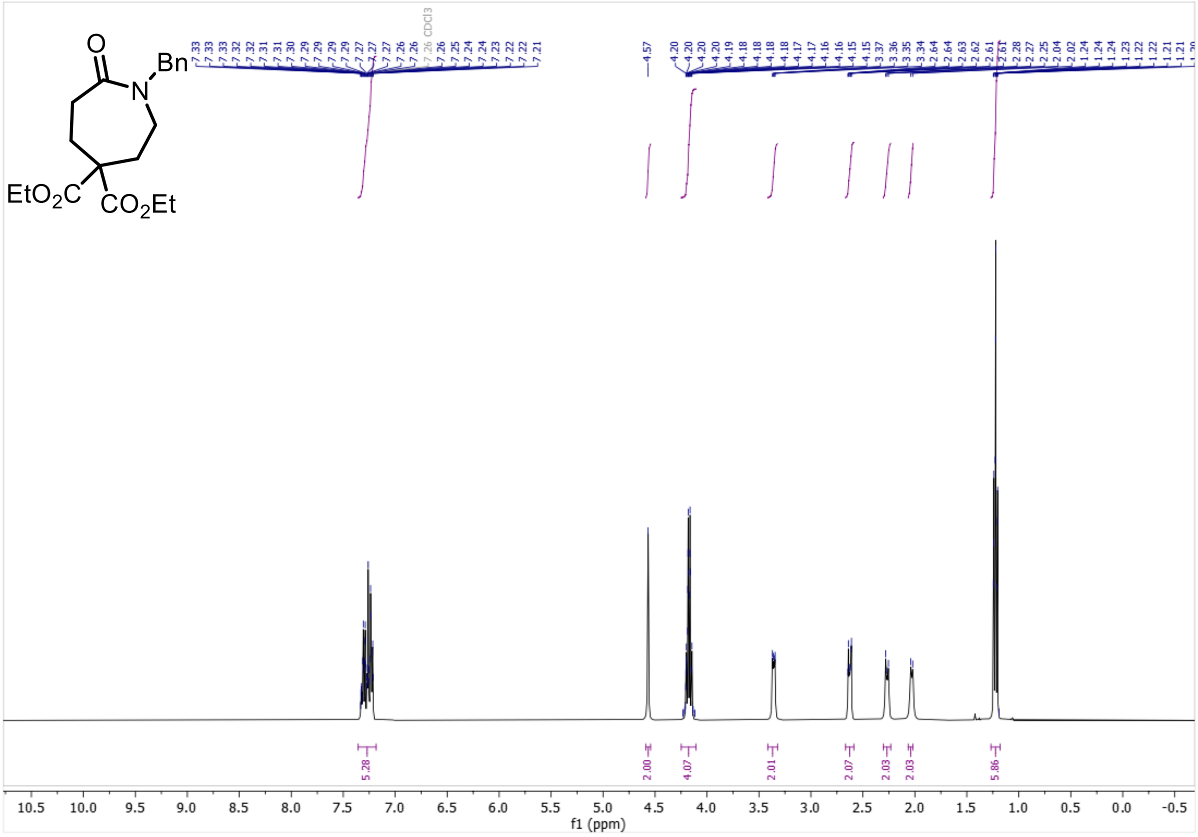

Compound 1k (^{13}C)

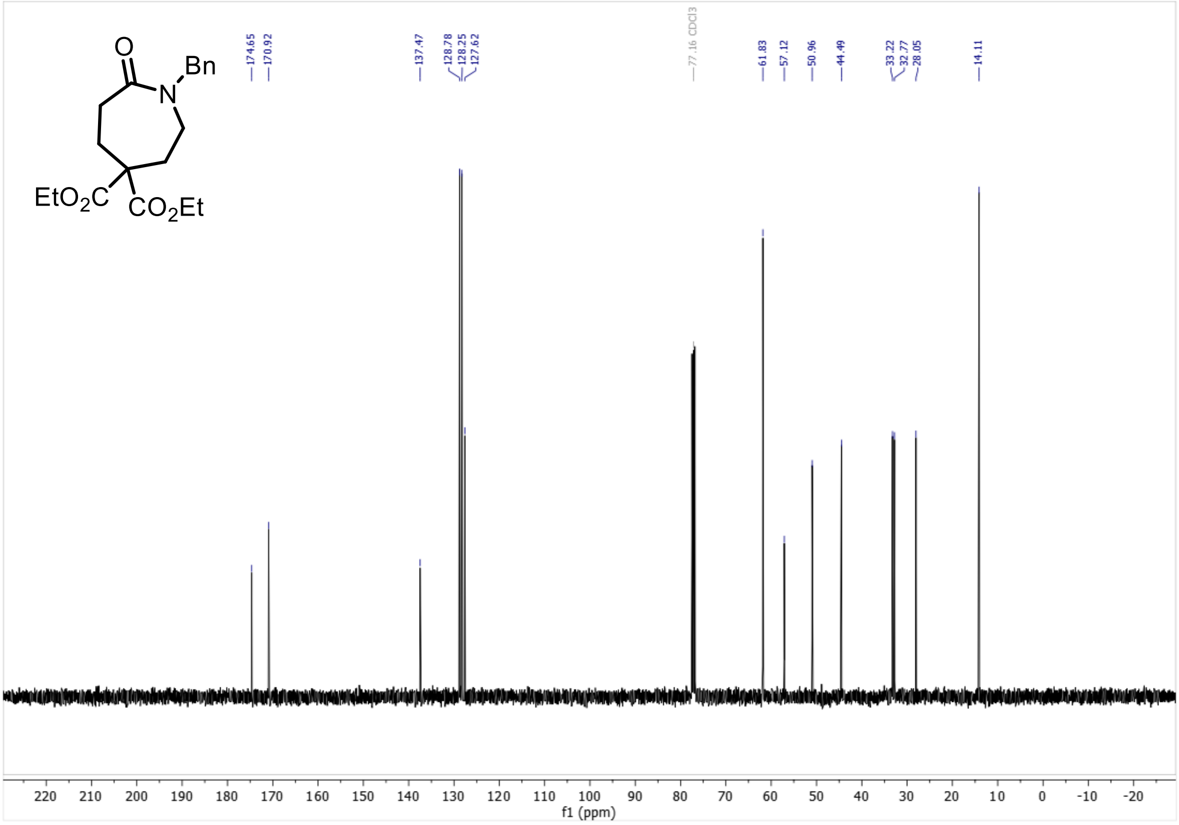

Compound 1l (^{1}H)

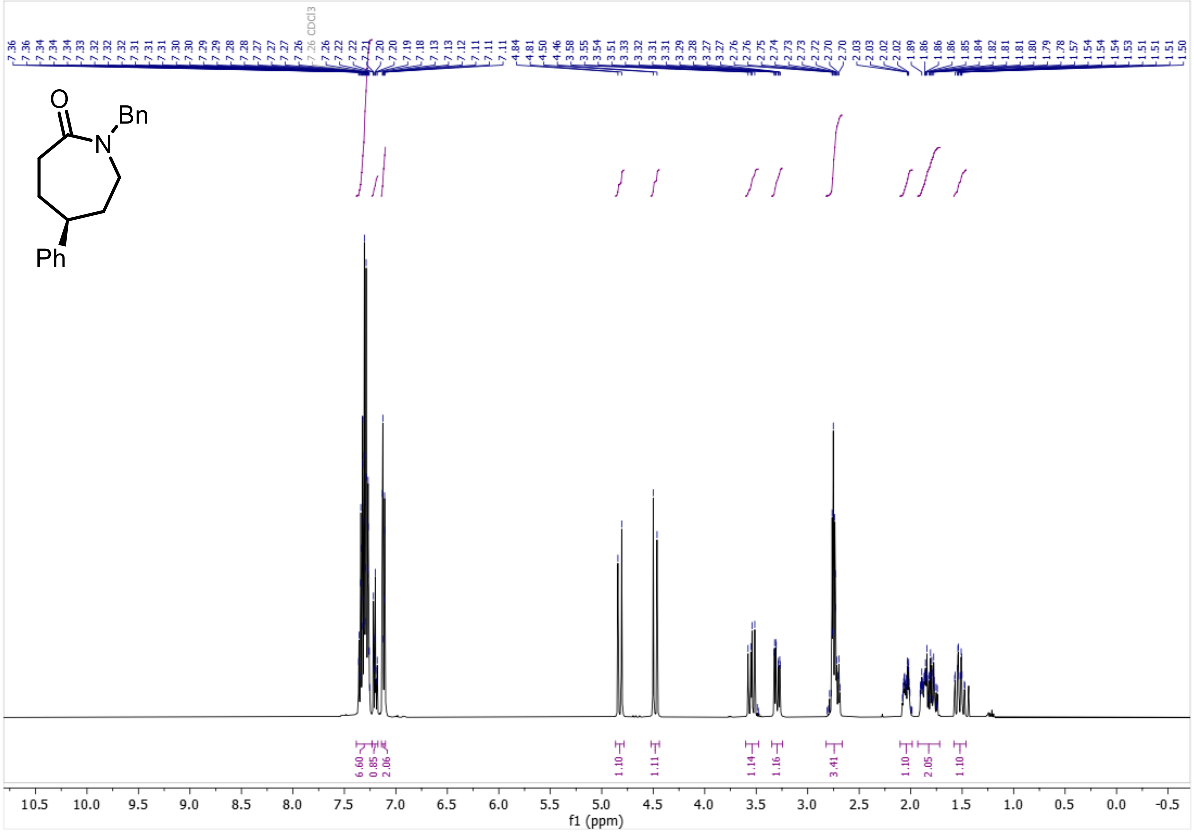

Compound 1l (^{13}C)

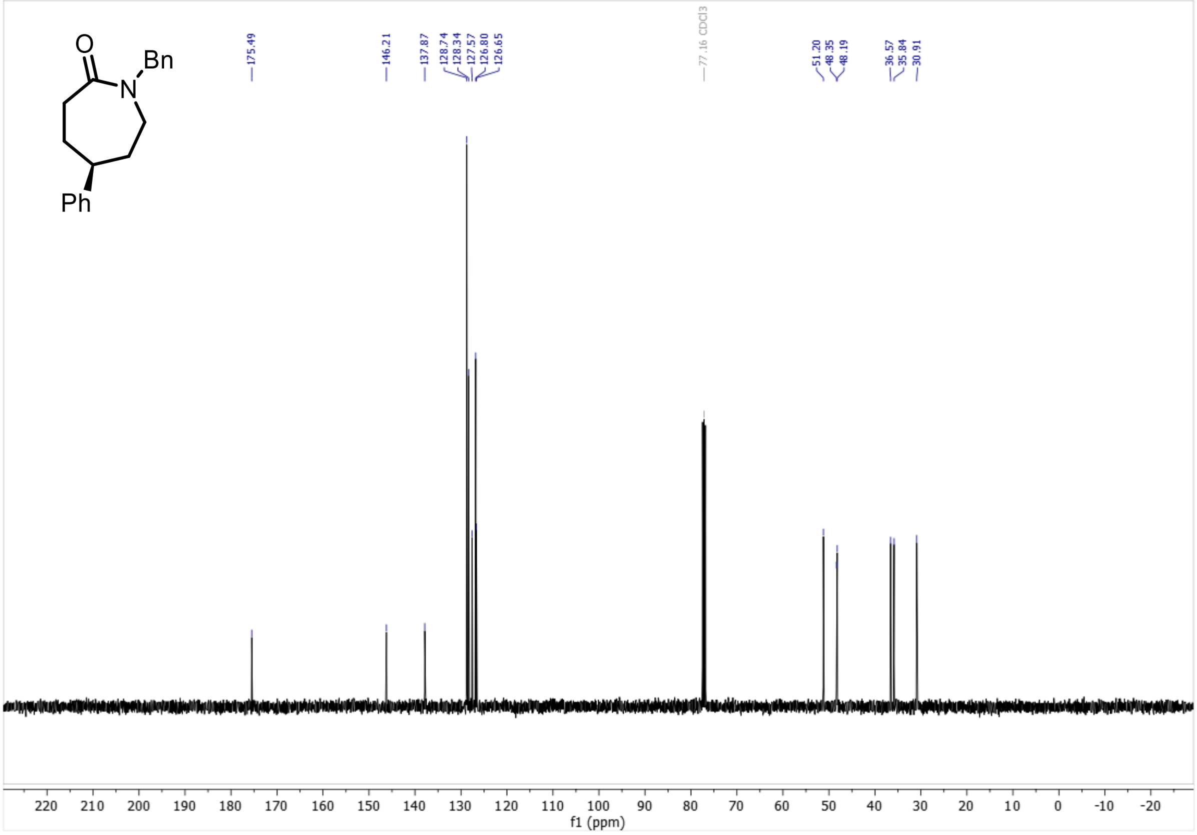

Compound 1m (^{1}H)

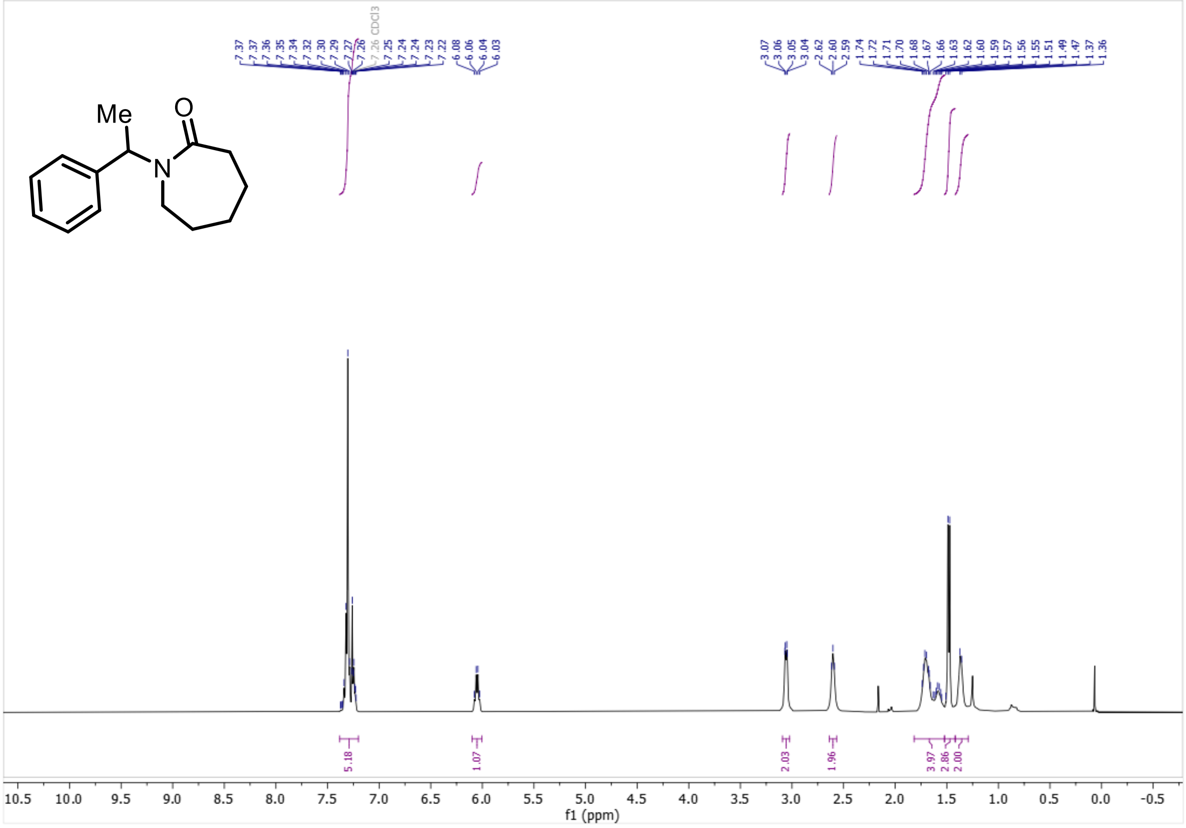

Compound 1m (^{13}C)

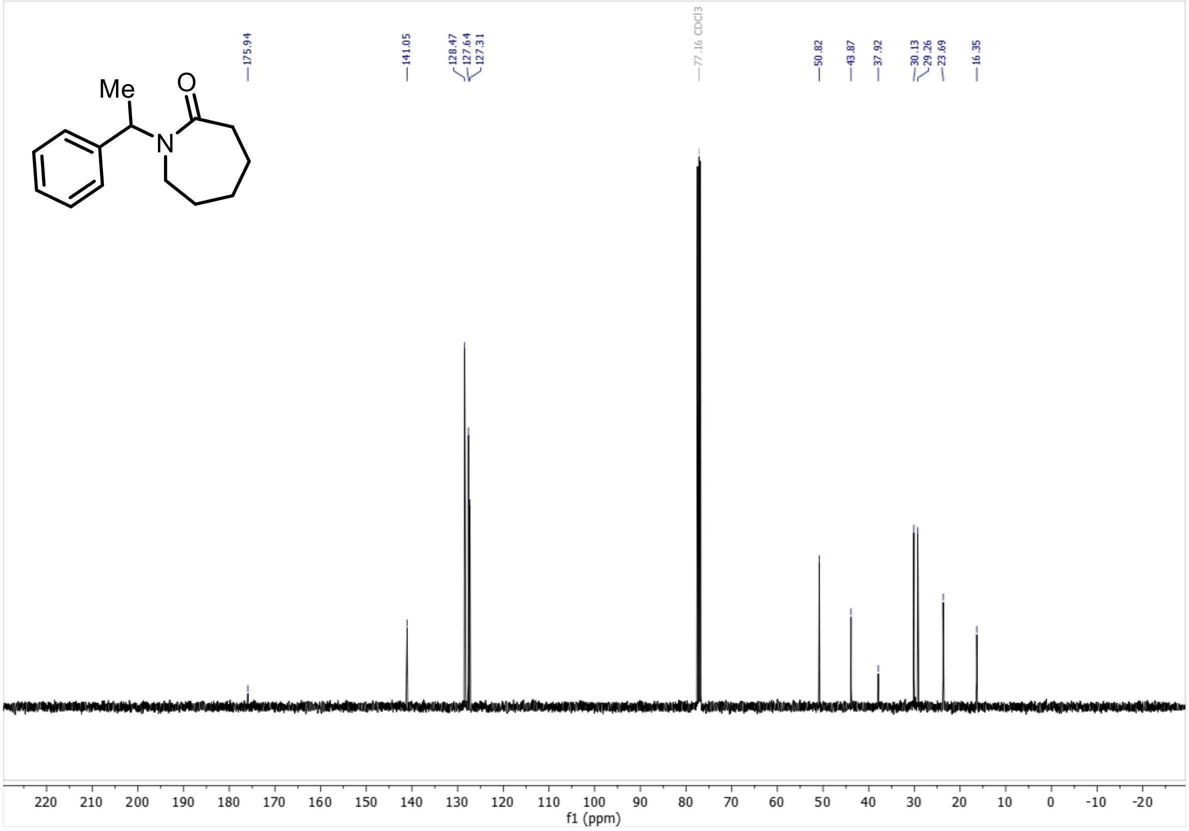

Compound 1n (^{1}H)

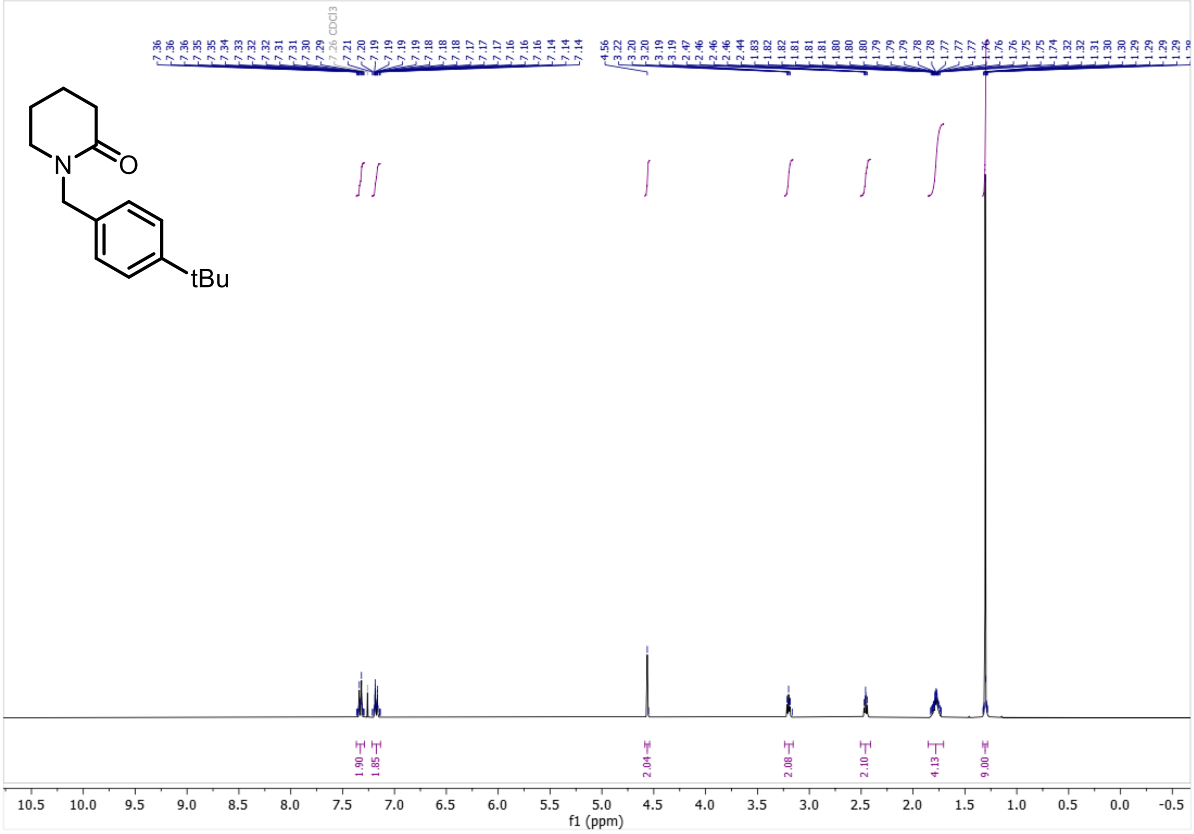

Compound 1n (^{13}C)

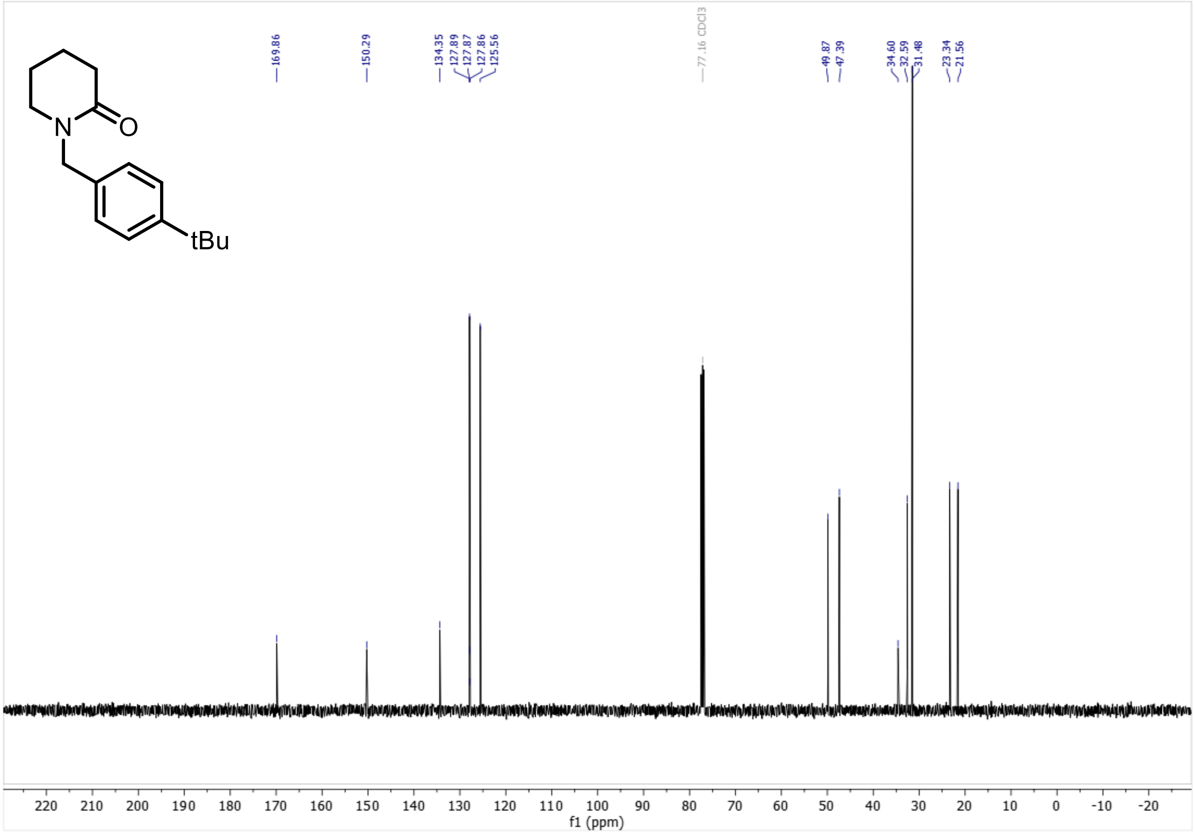

Compound 1r (^{1}H)

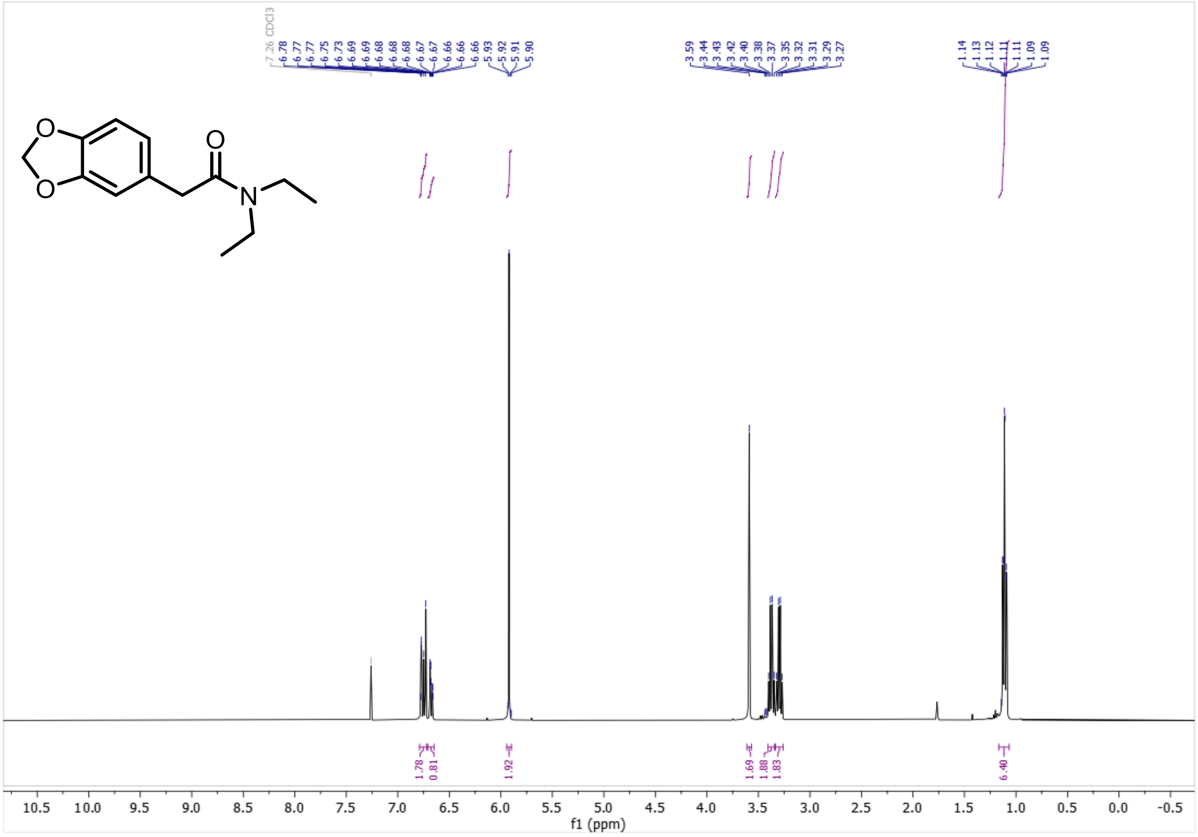

Compound 1r (^{13}C)

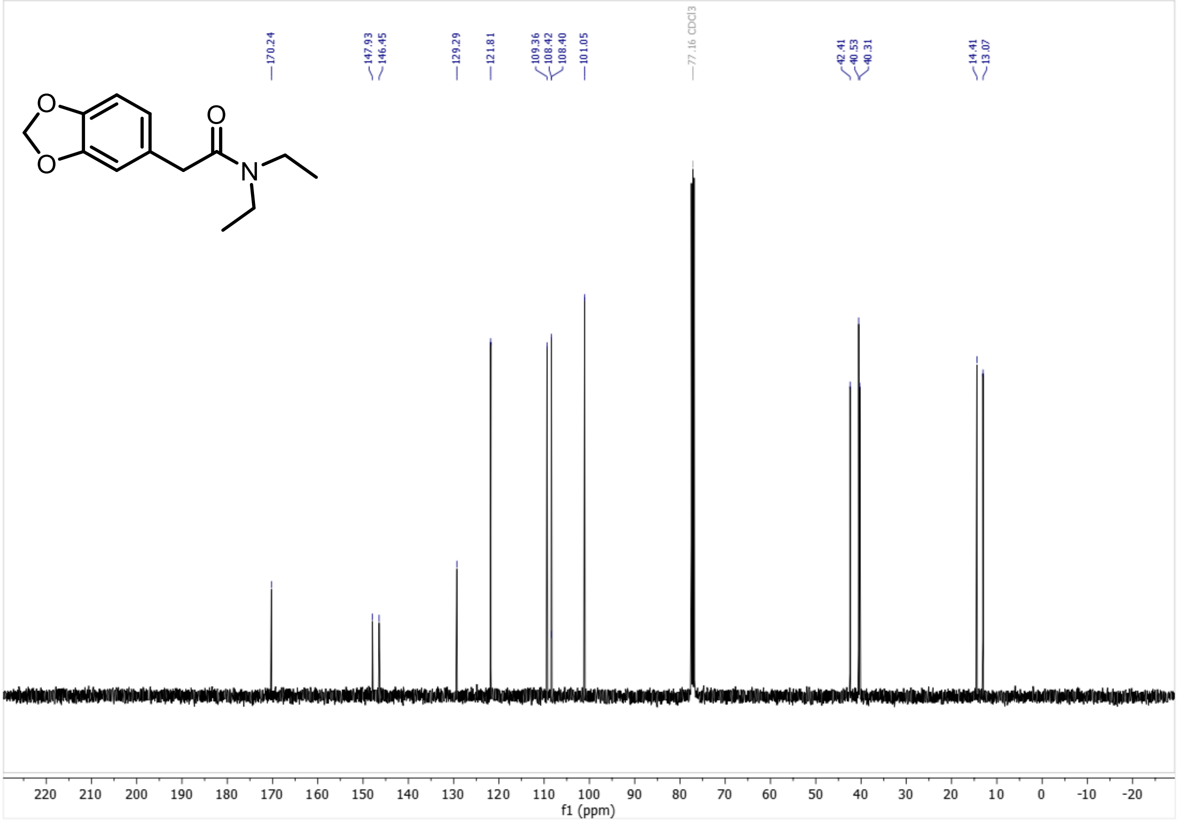

Compound 1x (^{1}H)

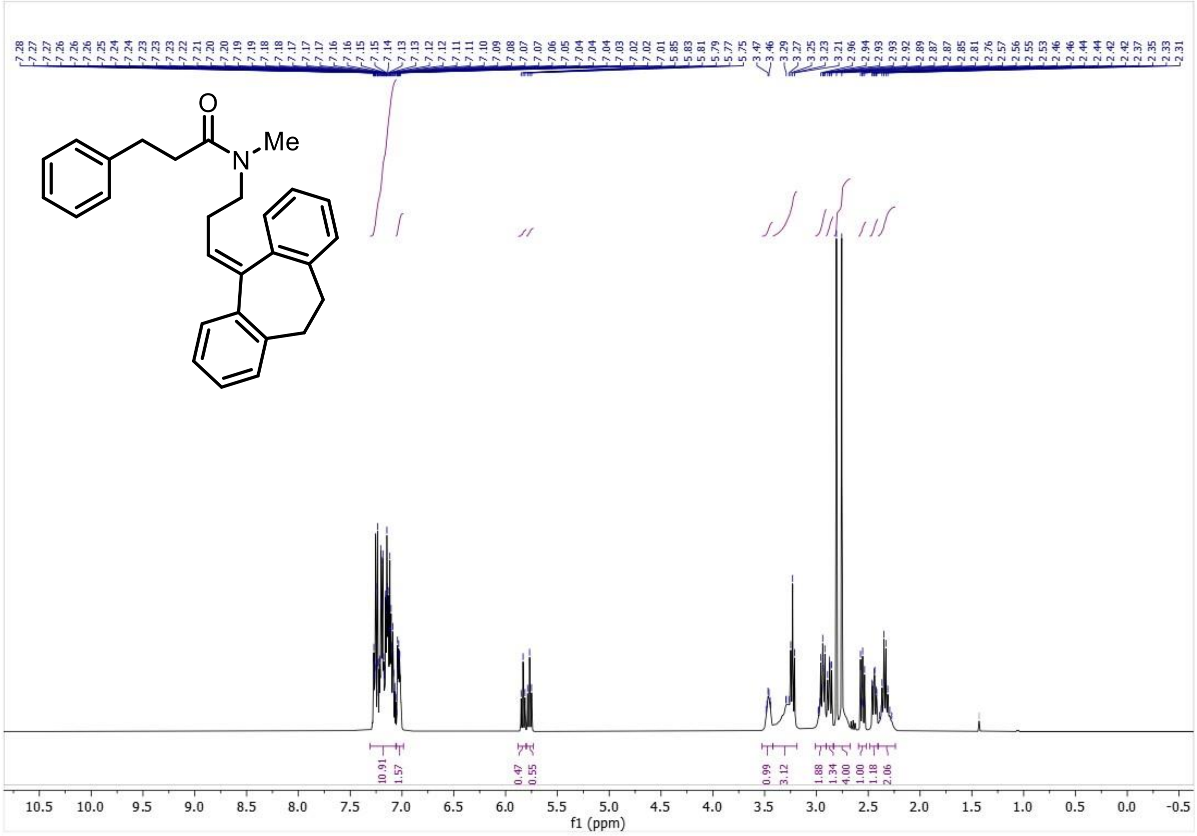

Compound 1x (^{13}C)

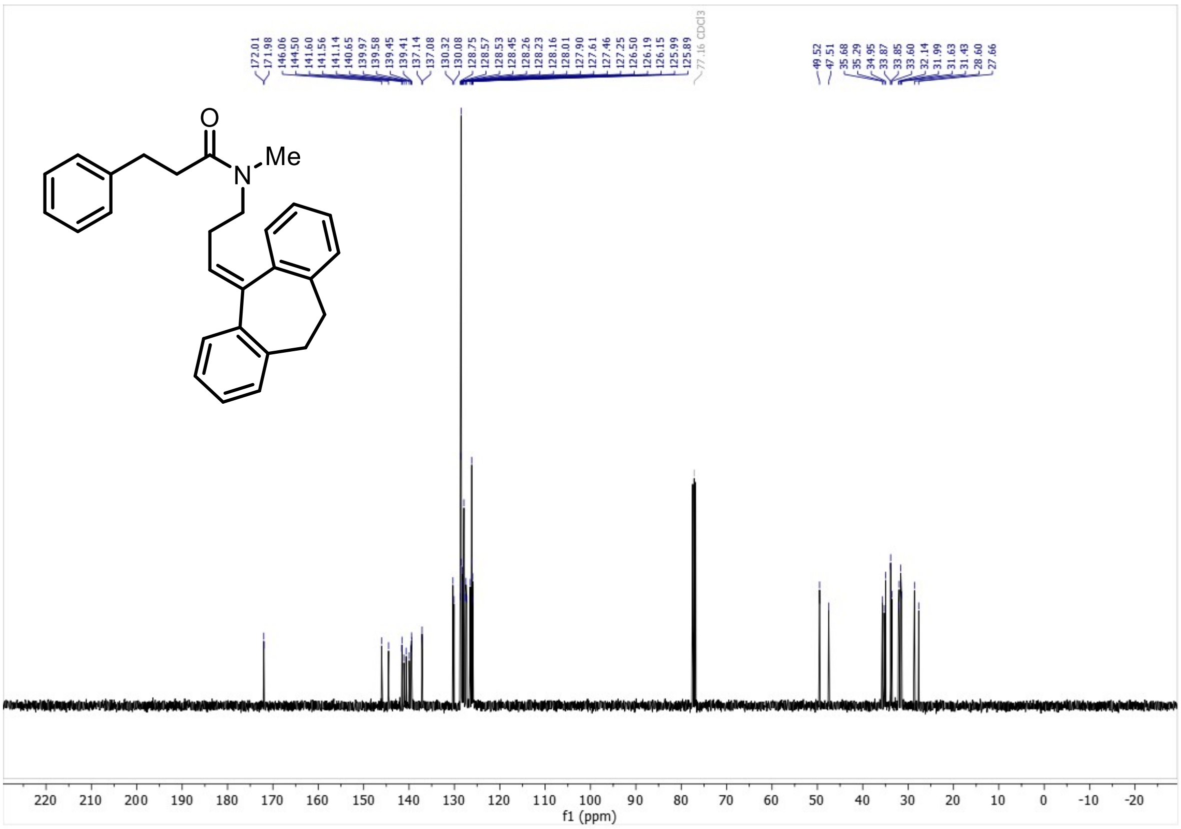

Compound S1-4 (^{1}H)

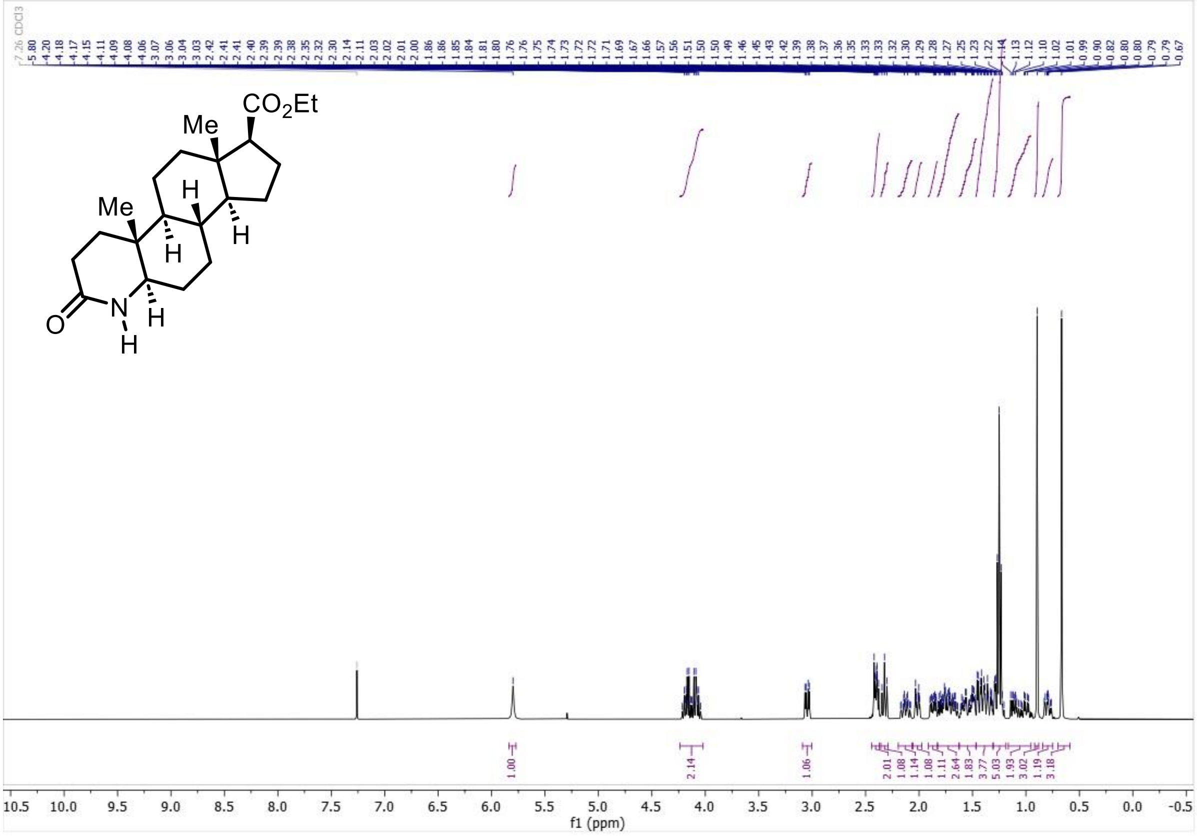

Compound S1-4 (^{13}C)

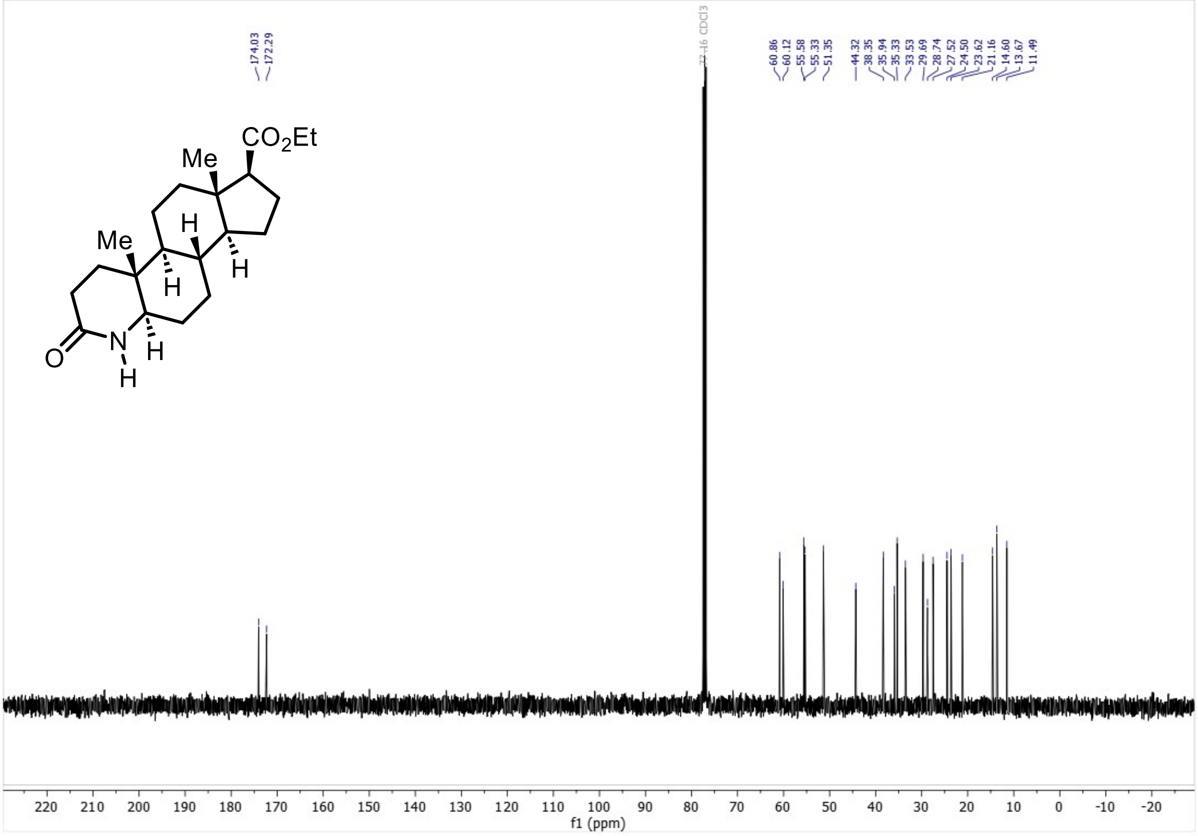

Compound 1y (^{1}H)

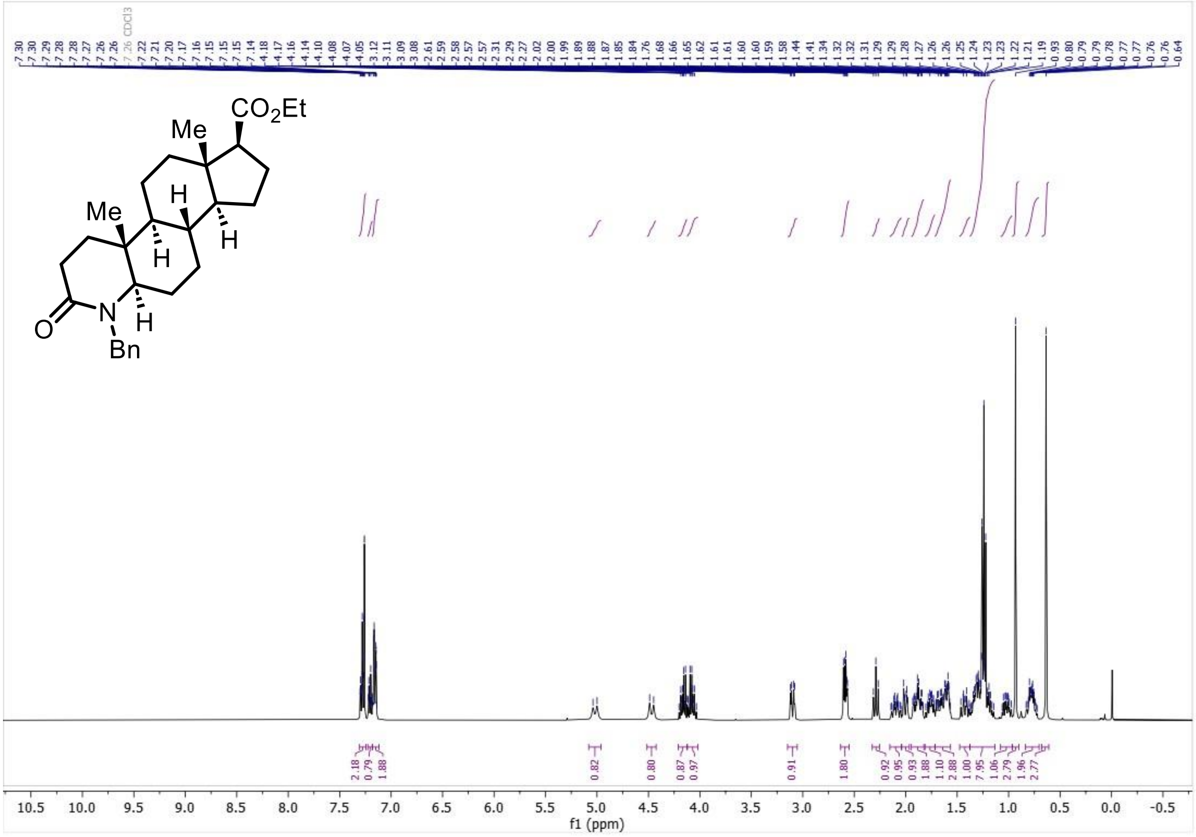

Compound 1y (^{13}C)

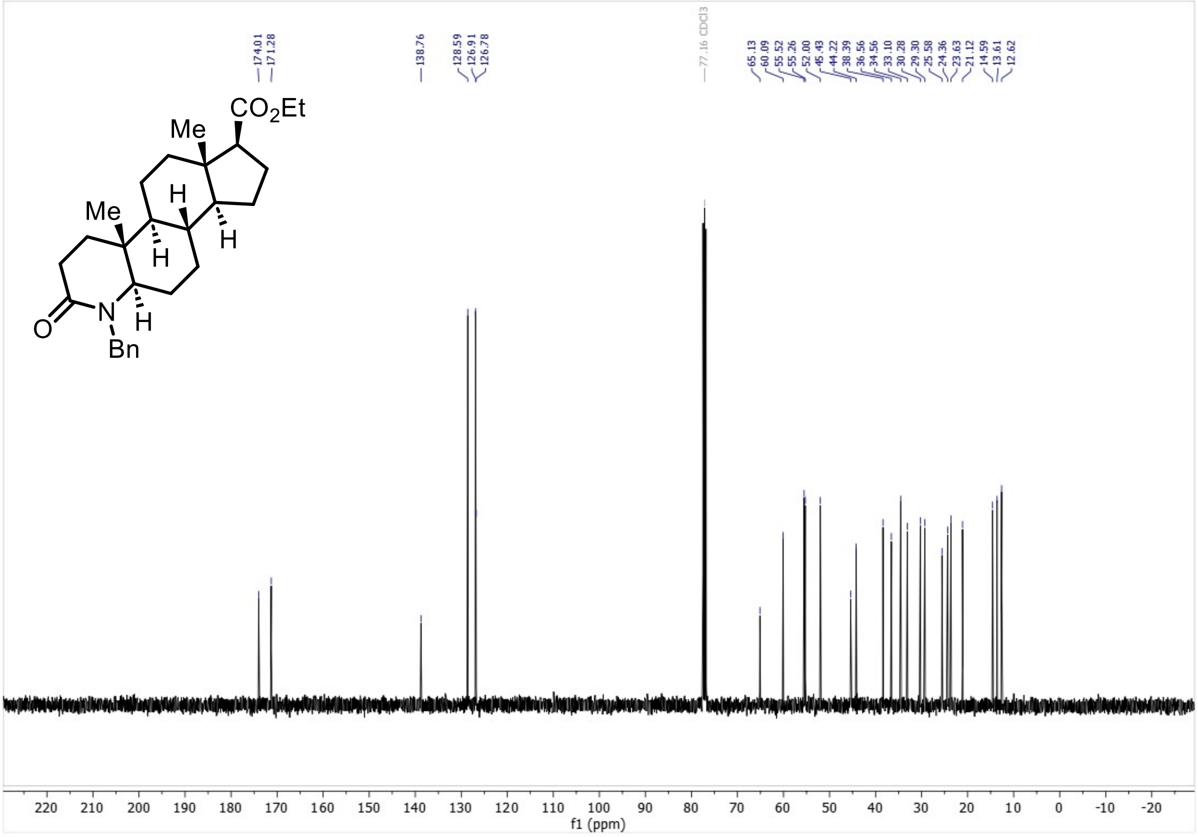

Compound 3a (^{1}H)

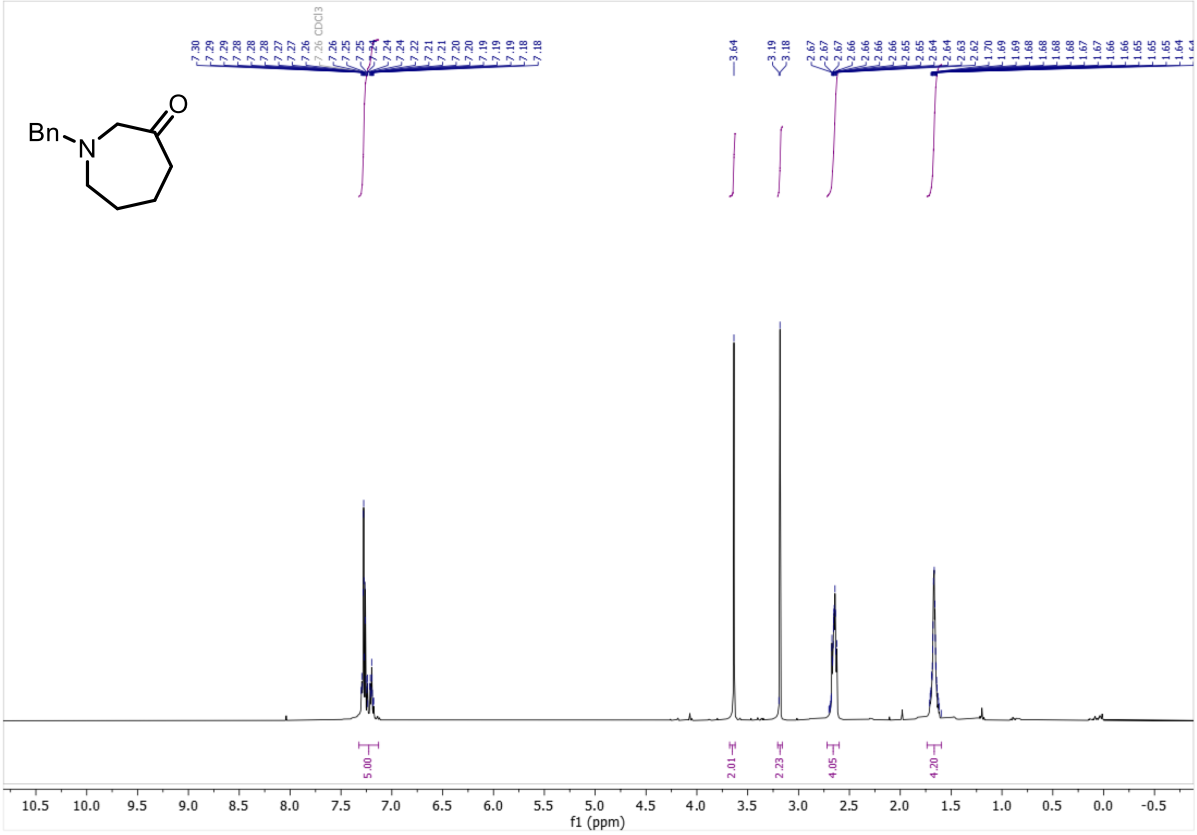

Compound 3a (^{13}C)

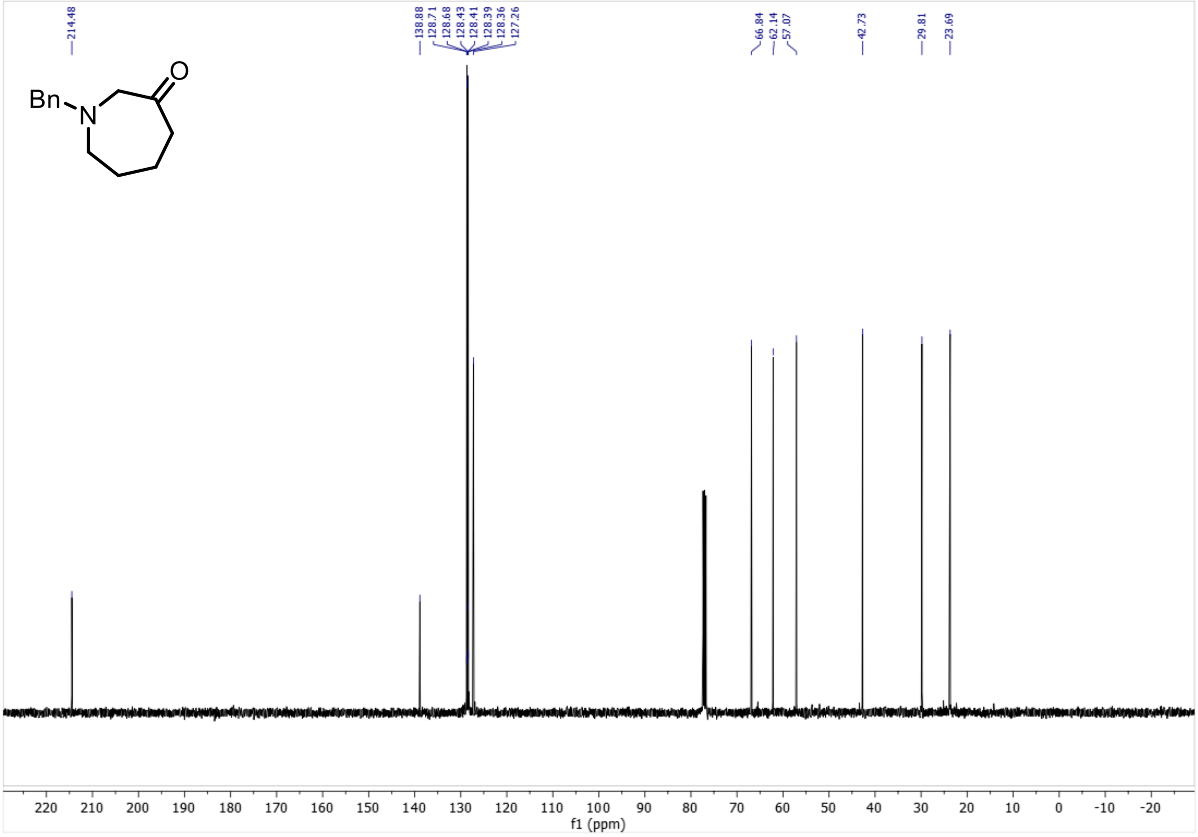

Compound 3c (^{1}H)

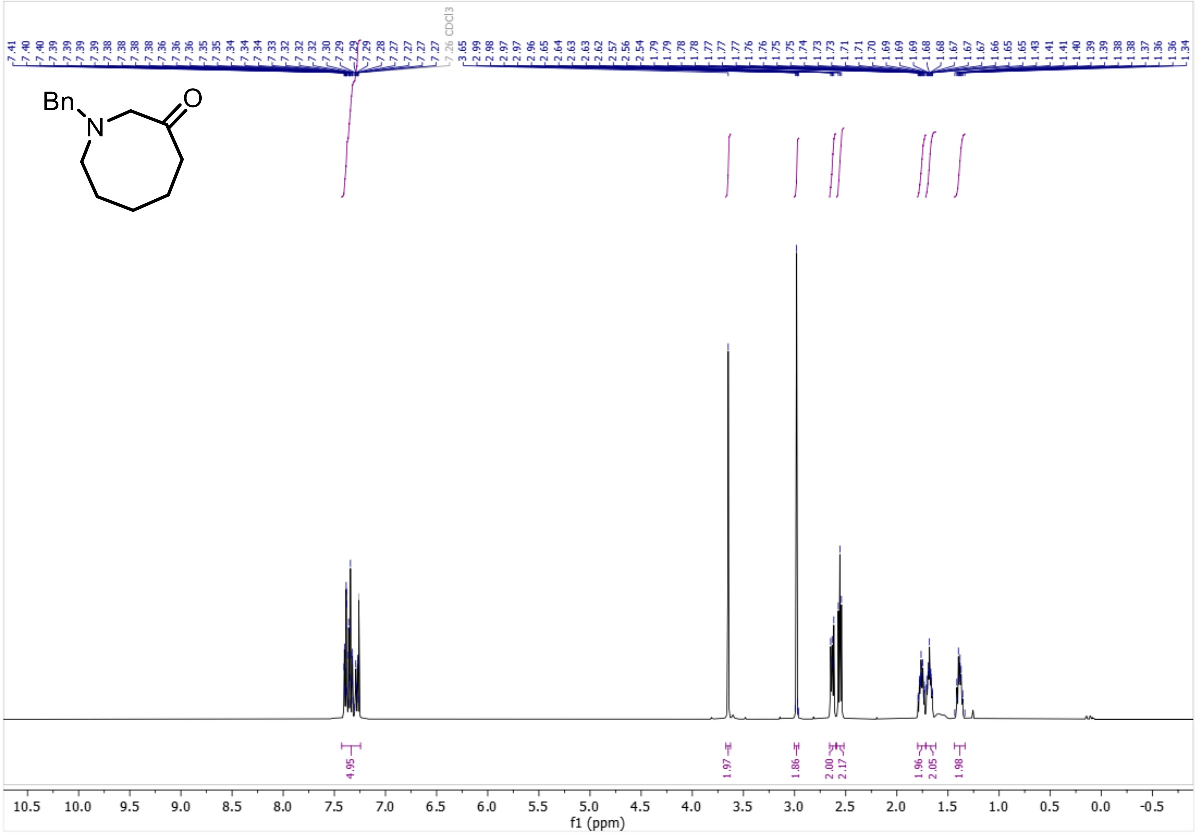

Compound 3c (^{13}C)

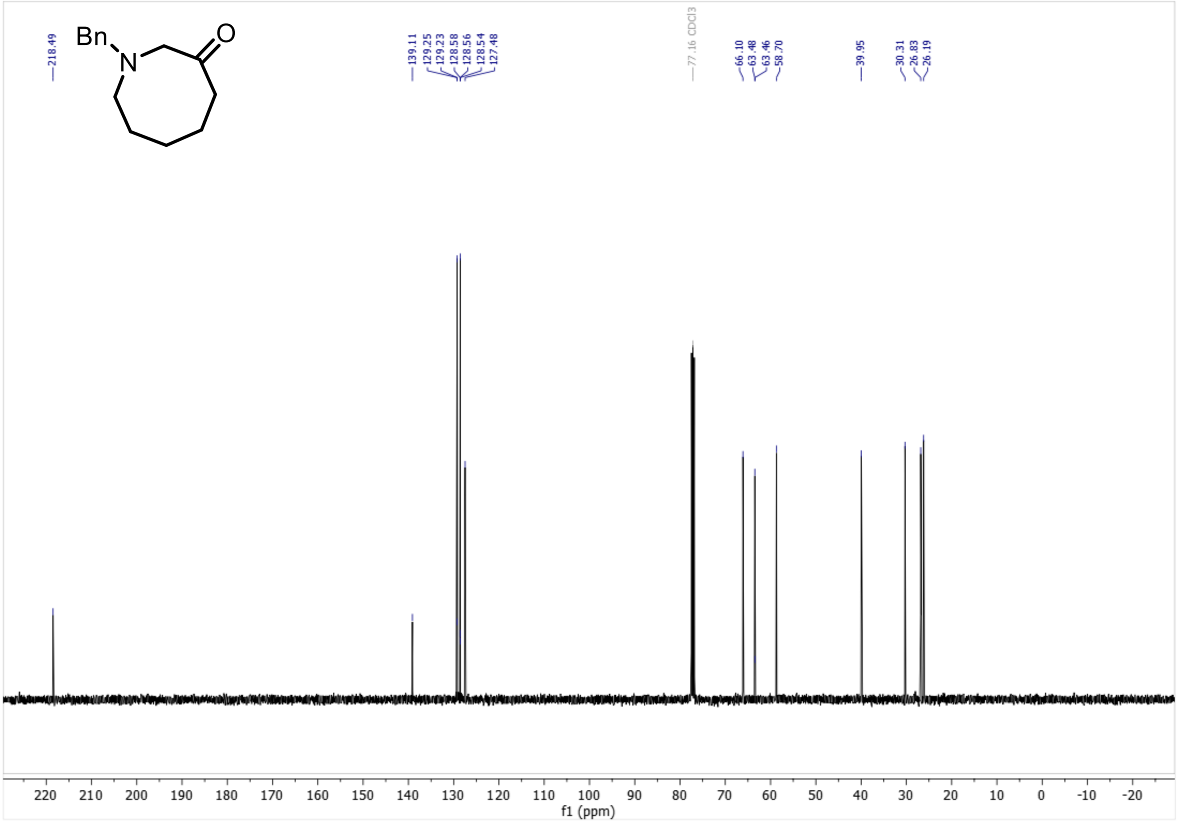

Compound 3d (^{1}H)

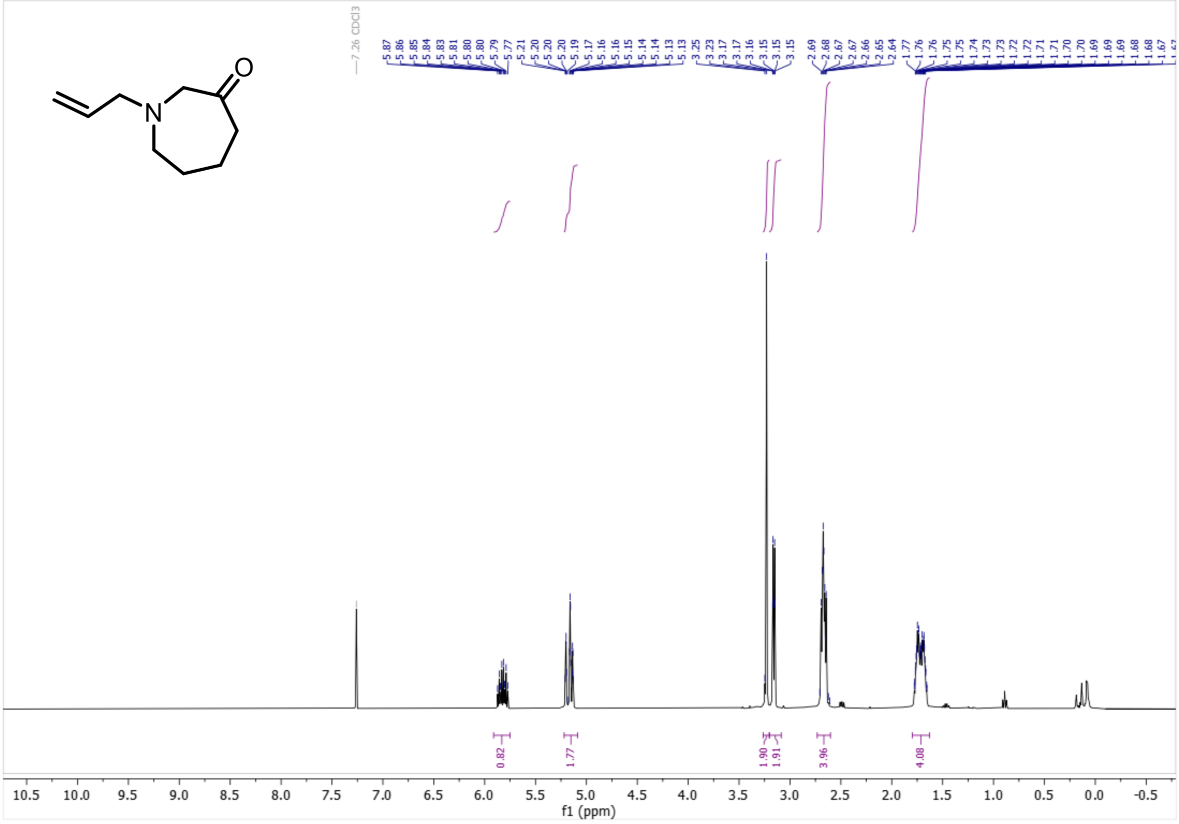

Compound 3d (^{13}C)

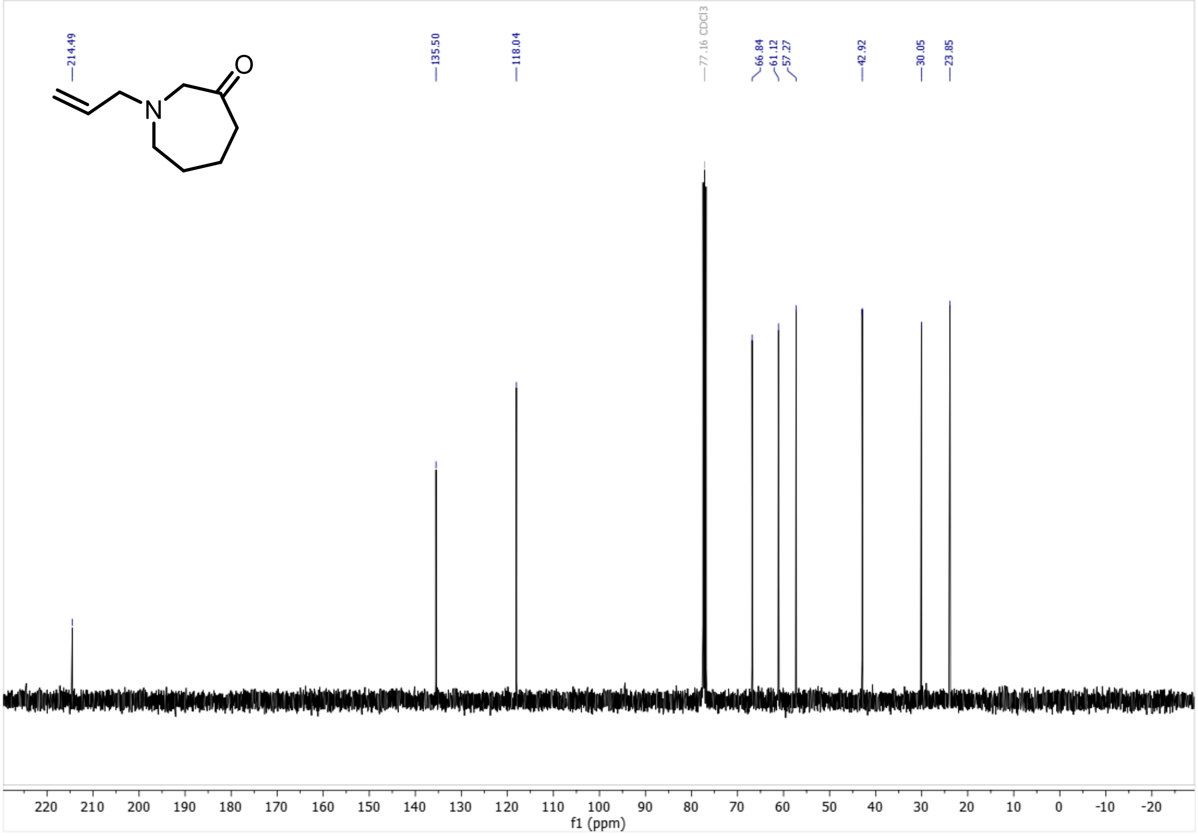

Compound 3e (¹H)

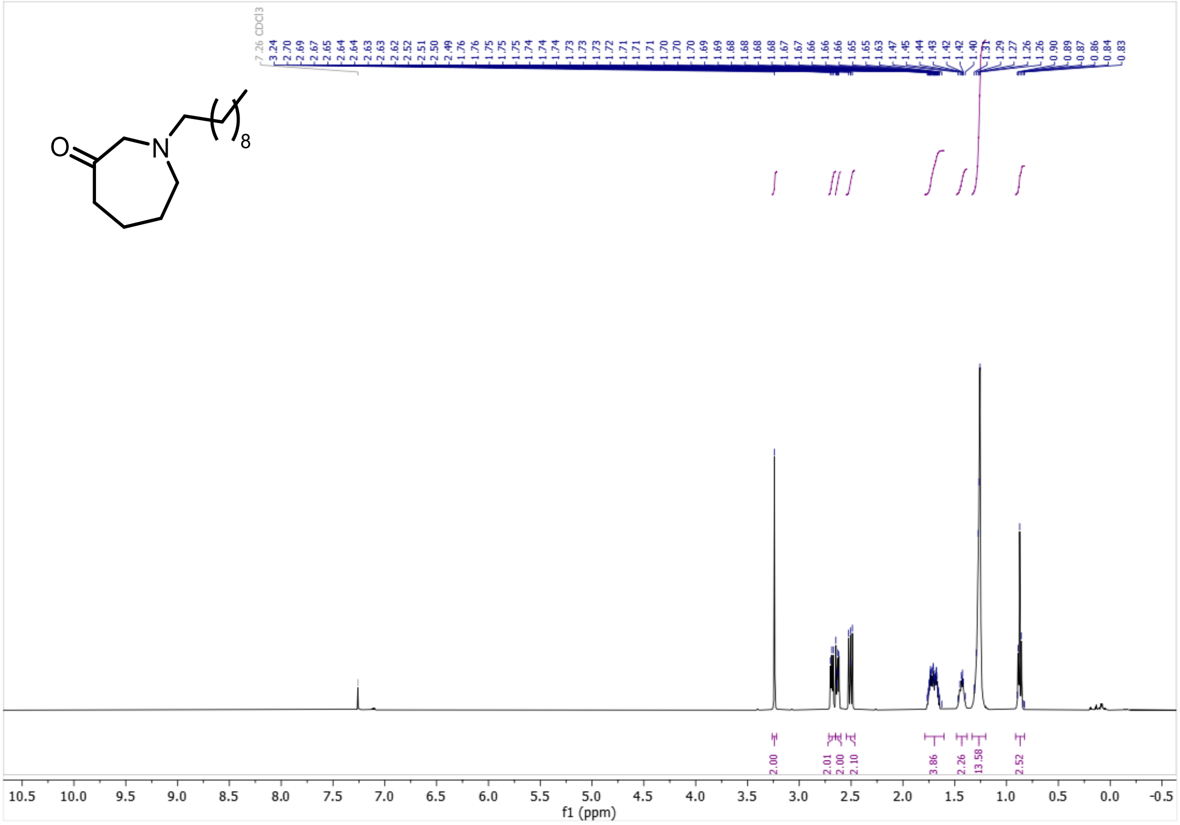

Compound 3e (¹³C)

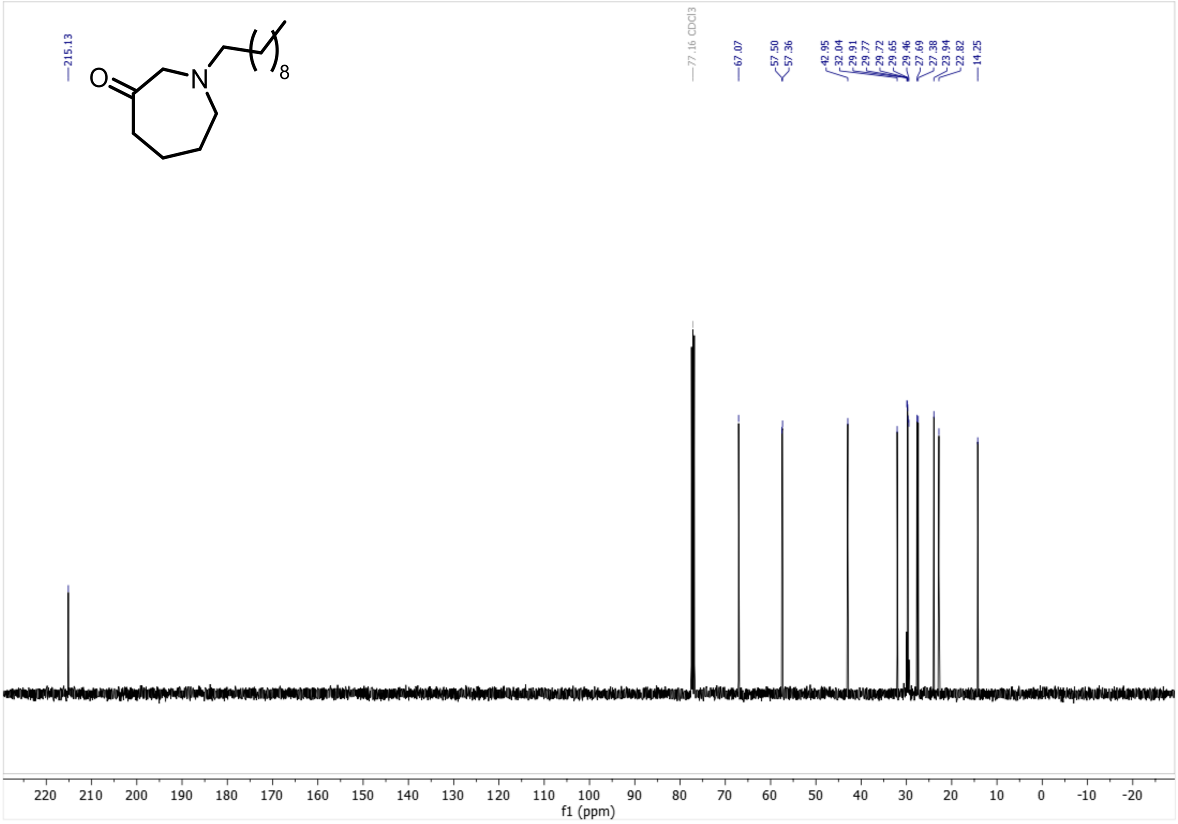

Compound 3f (^{1}H)

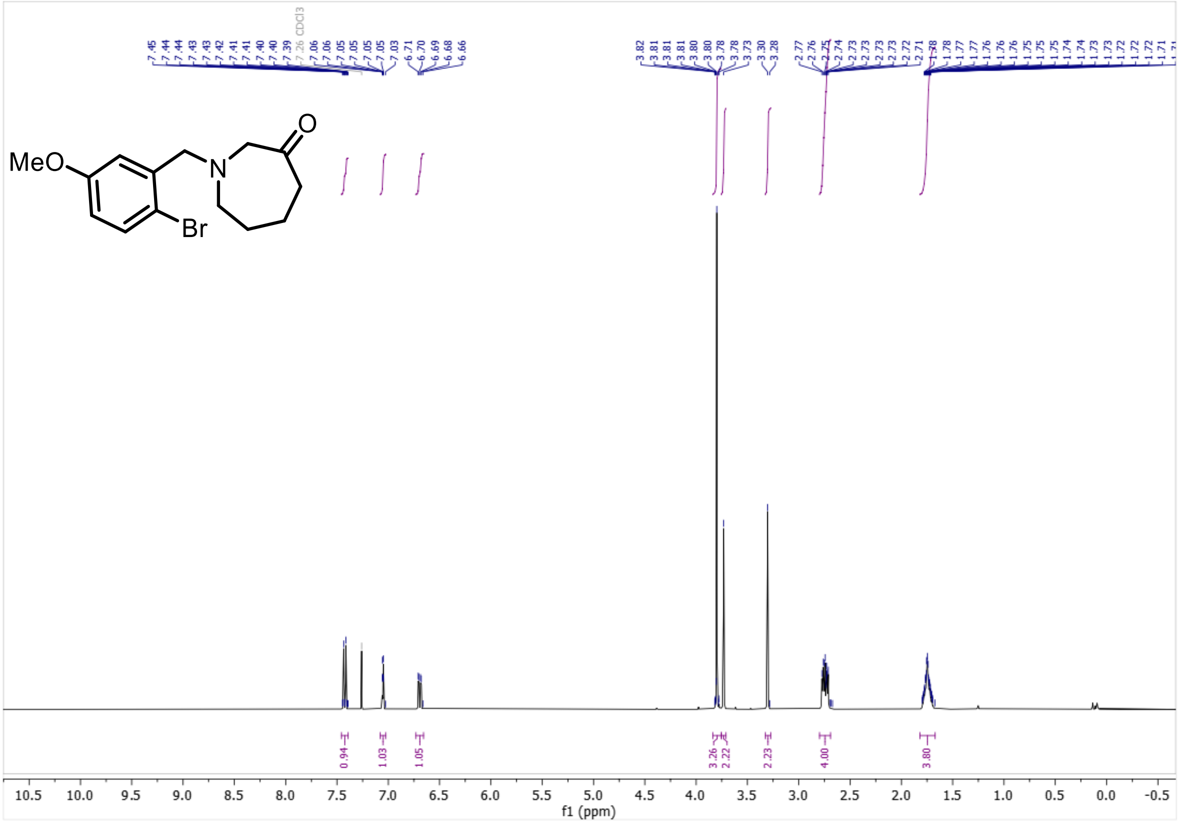

Compound 3f (^{13}C)

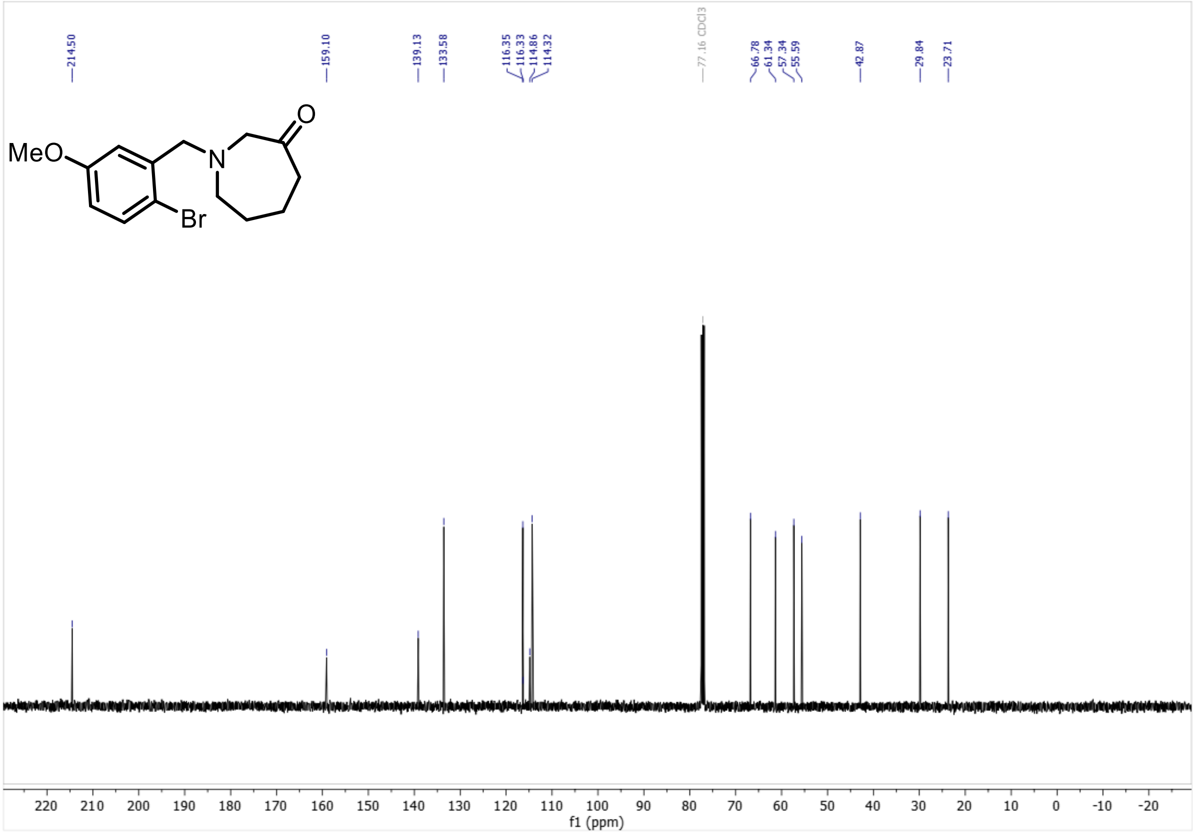

Compound 3g (^{1}H)

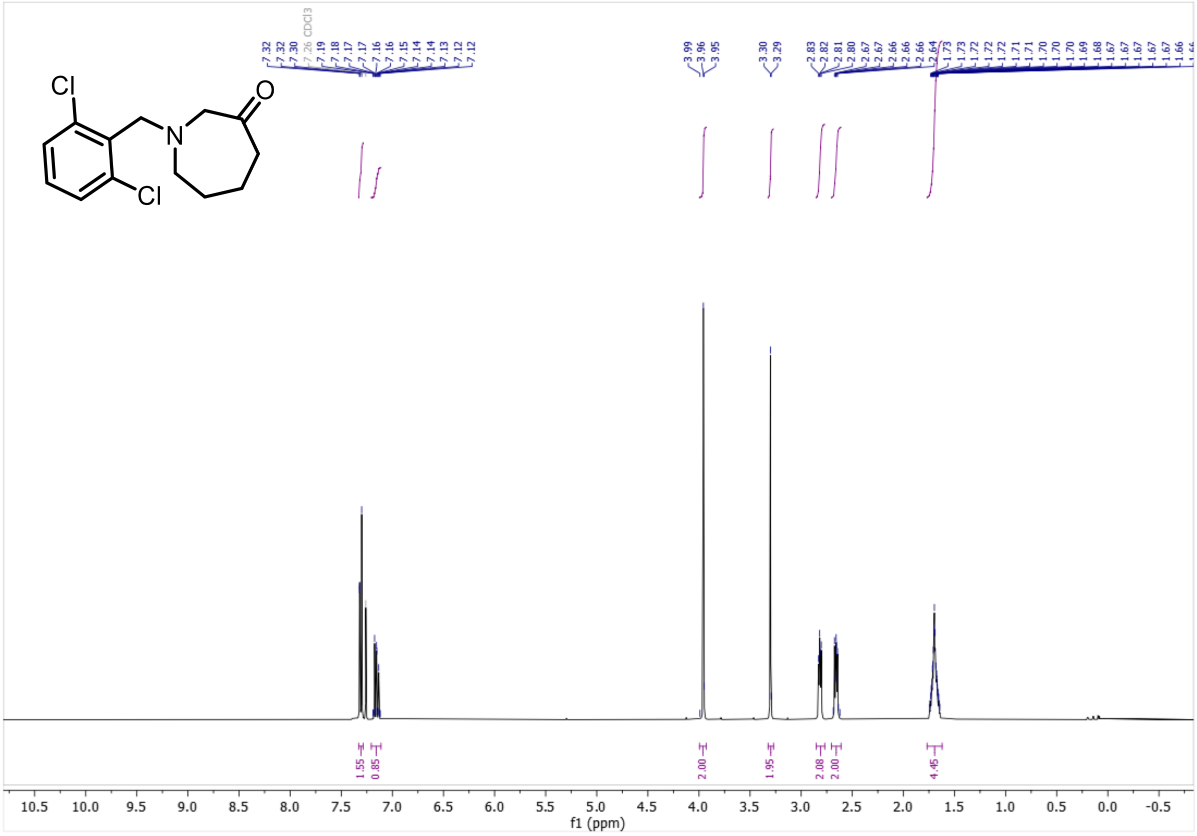

Compound 3g (^{13}C)

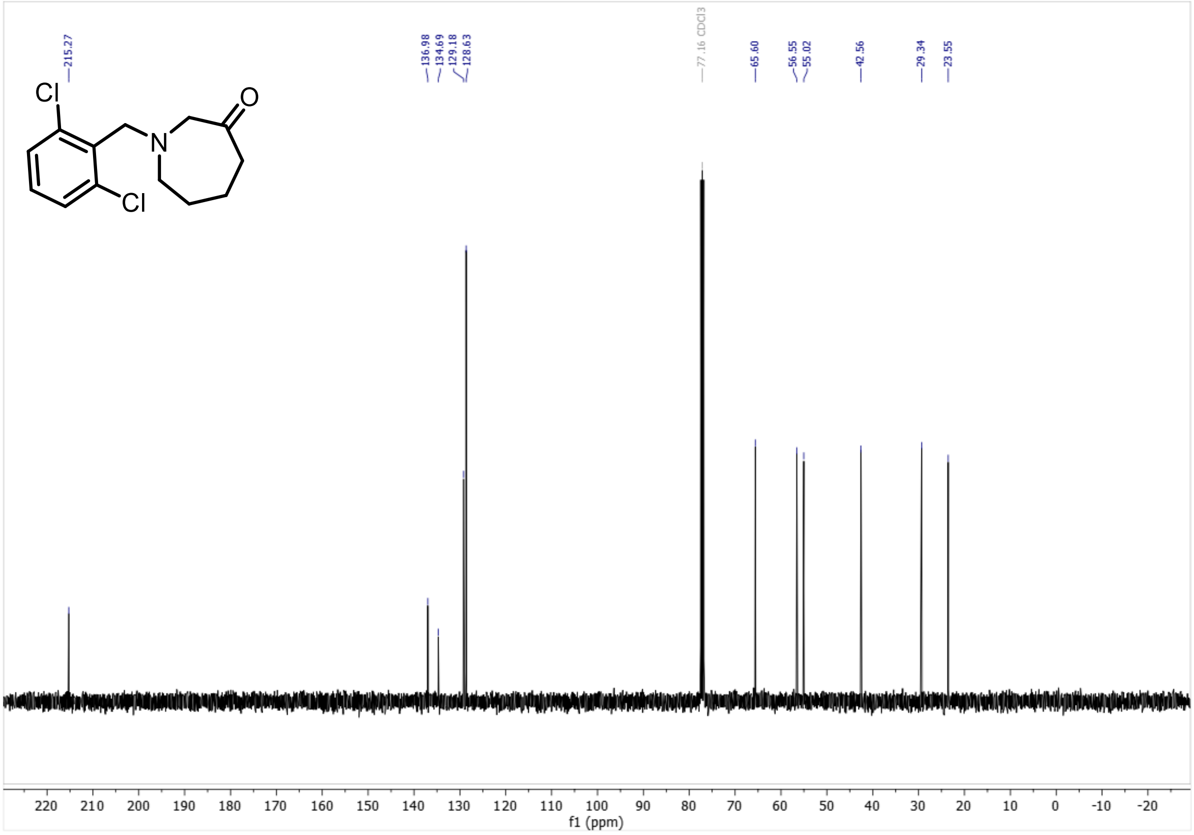

Compound 3h (^{1}H)

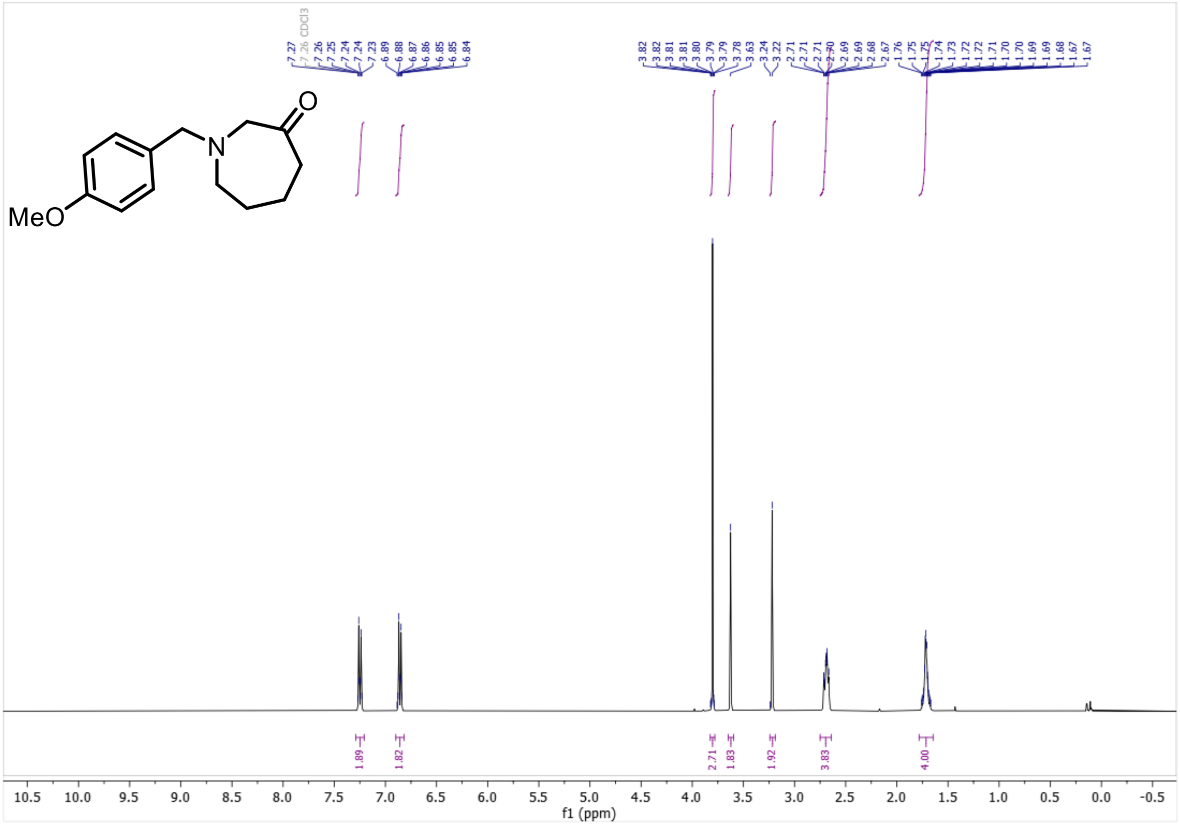

Compound 3h (^{13}C)

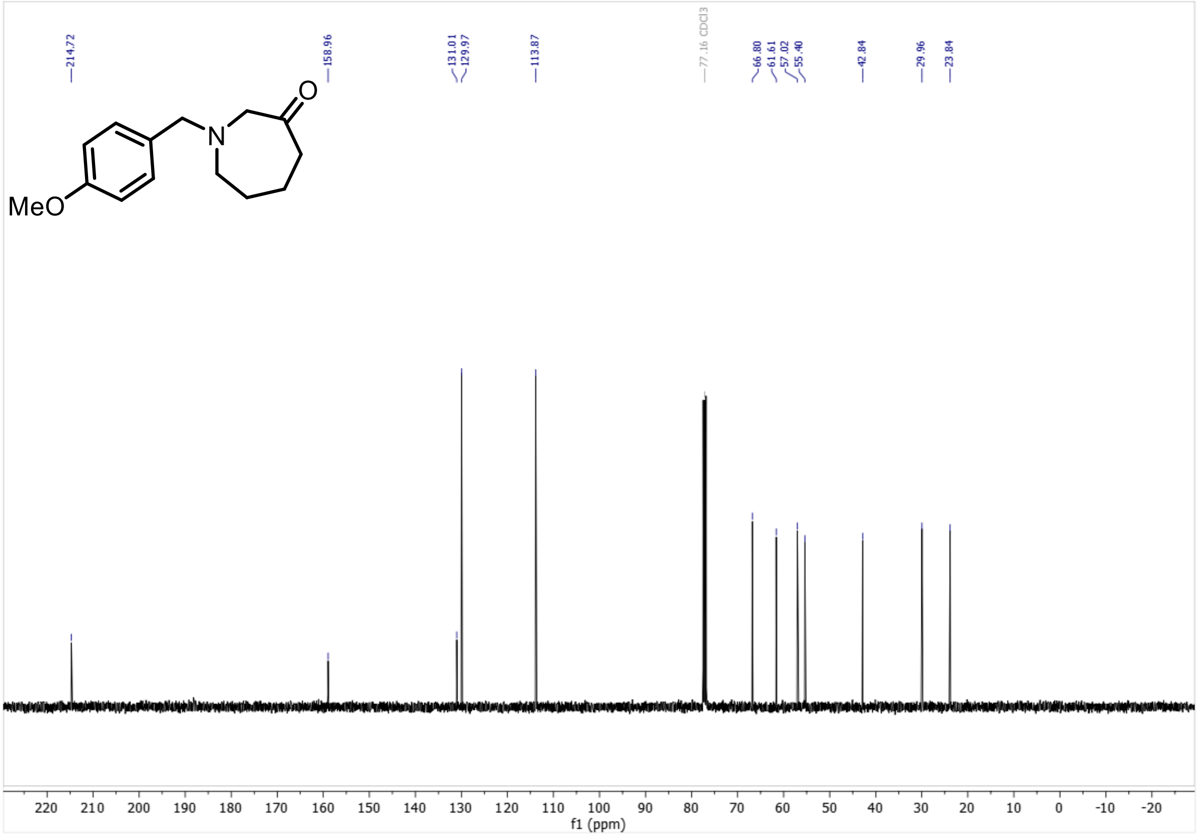

Compound 3i (^{1}H)

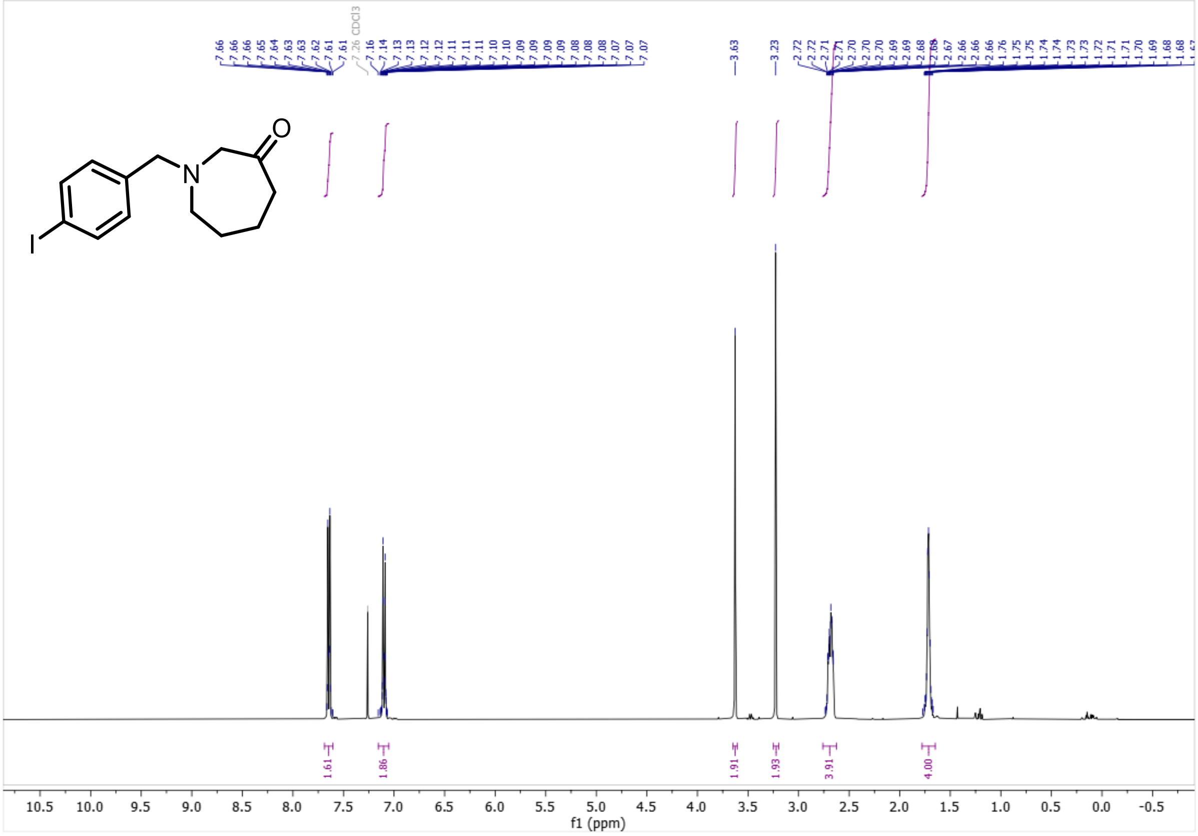

Compound 3i (^{13}C)

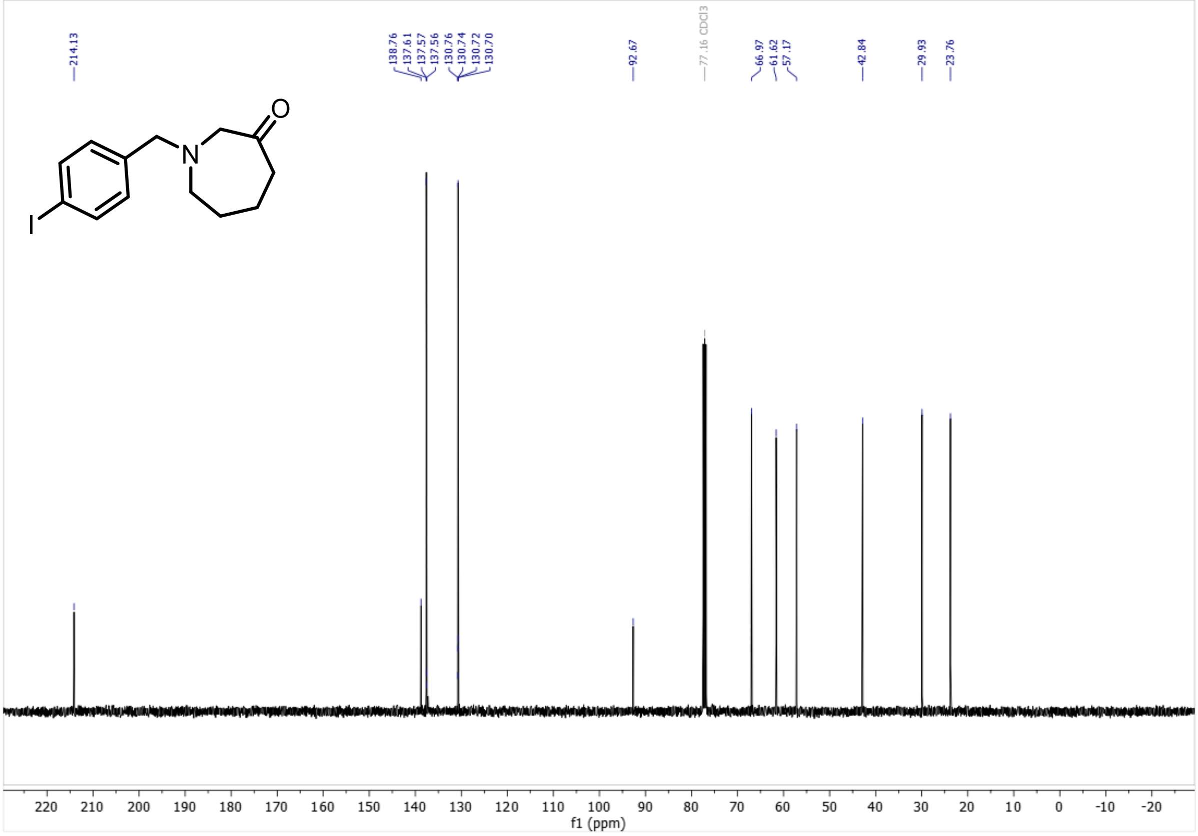

Compound 3j (^{1}H)

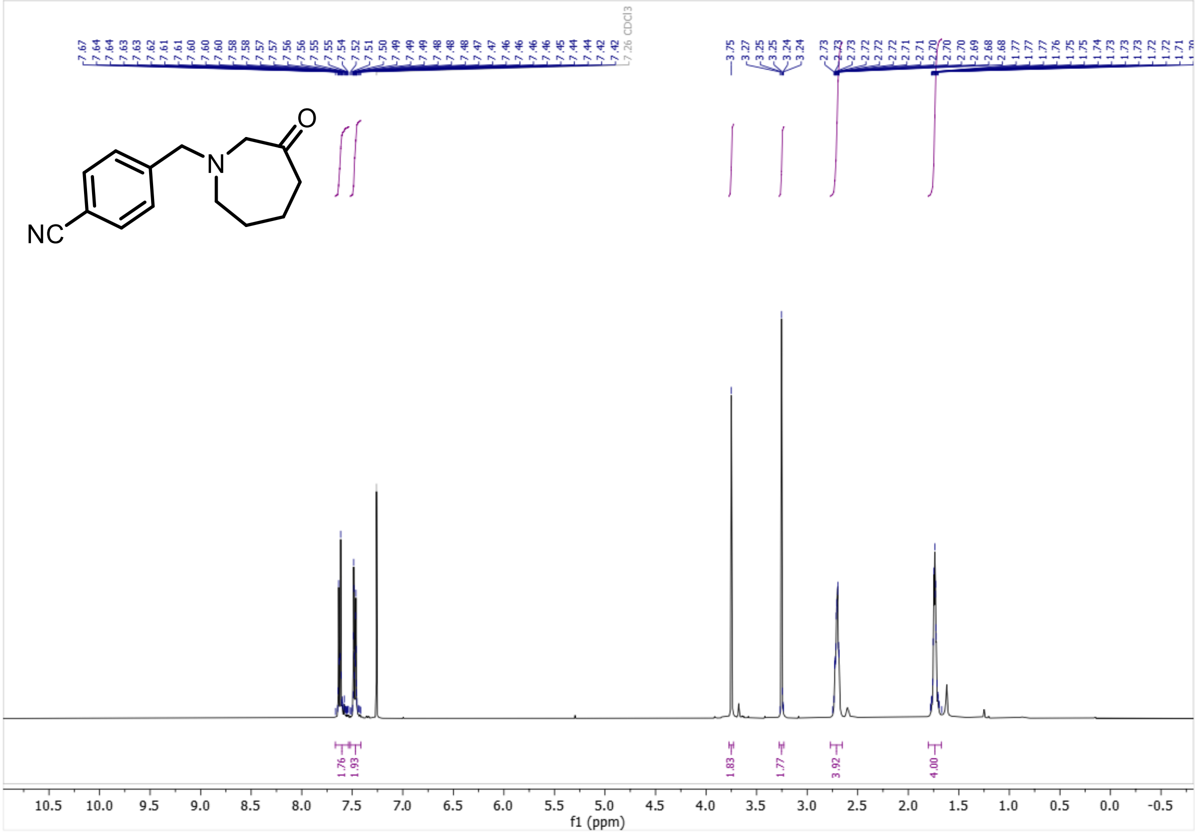

Compound 3j (^{13}C)

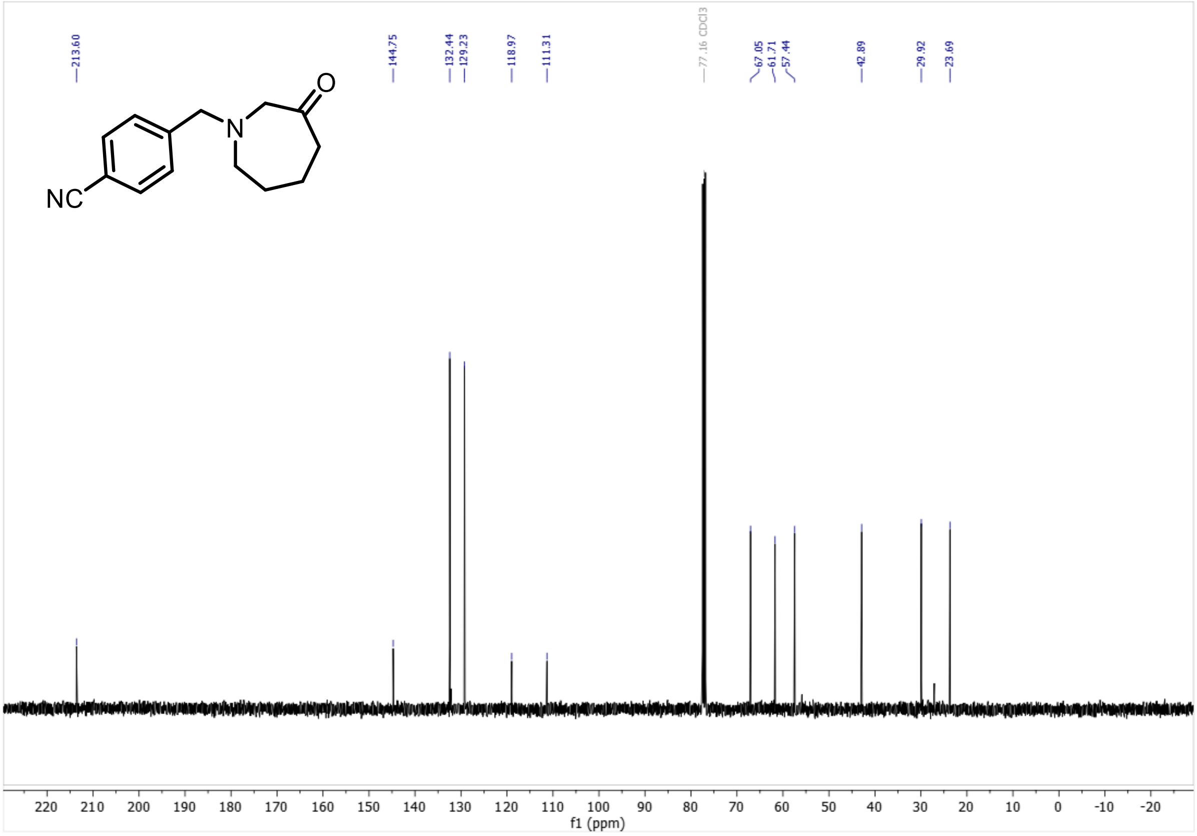

Compound 3k (^{1}H)

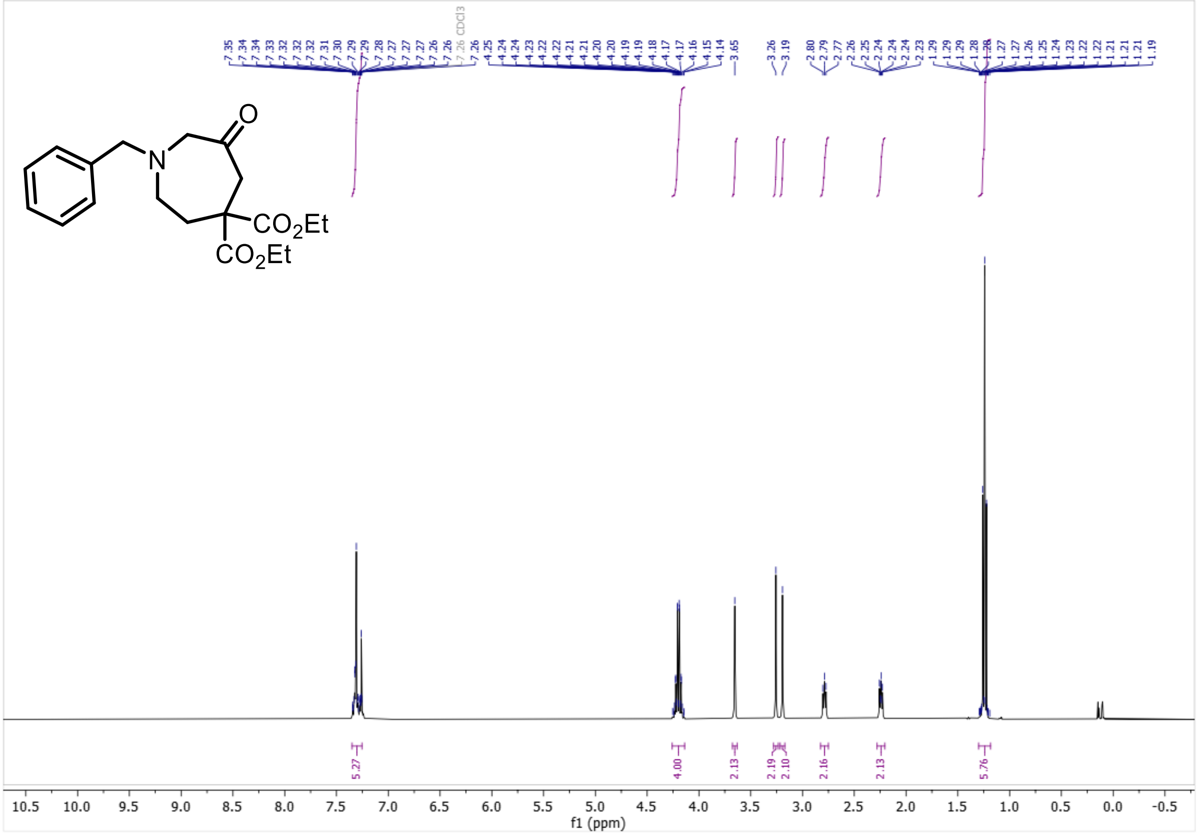

Compound 3k (^{13}C)

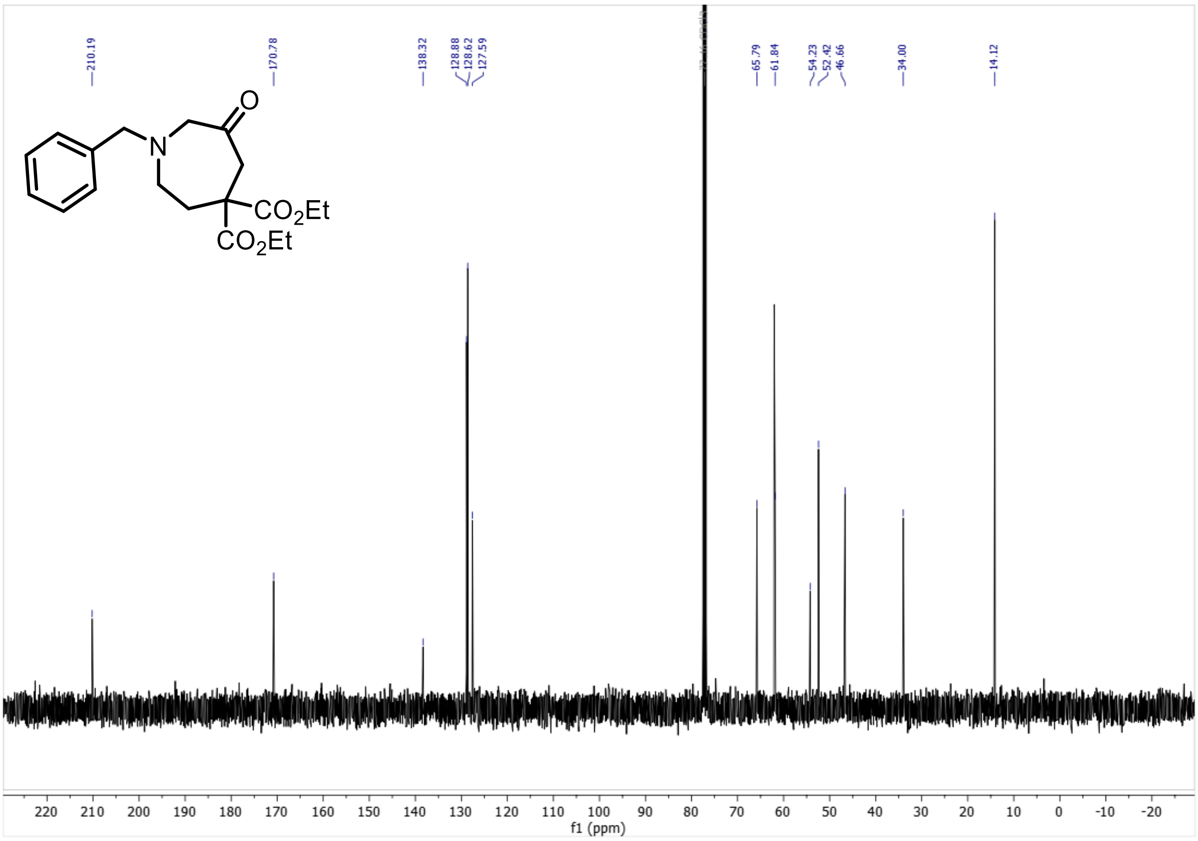

Compound 3l (^{1}H)

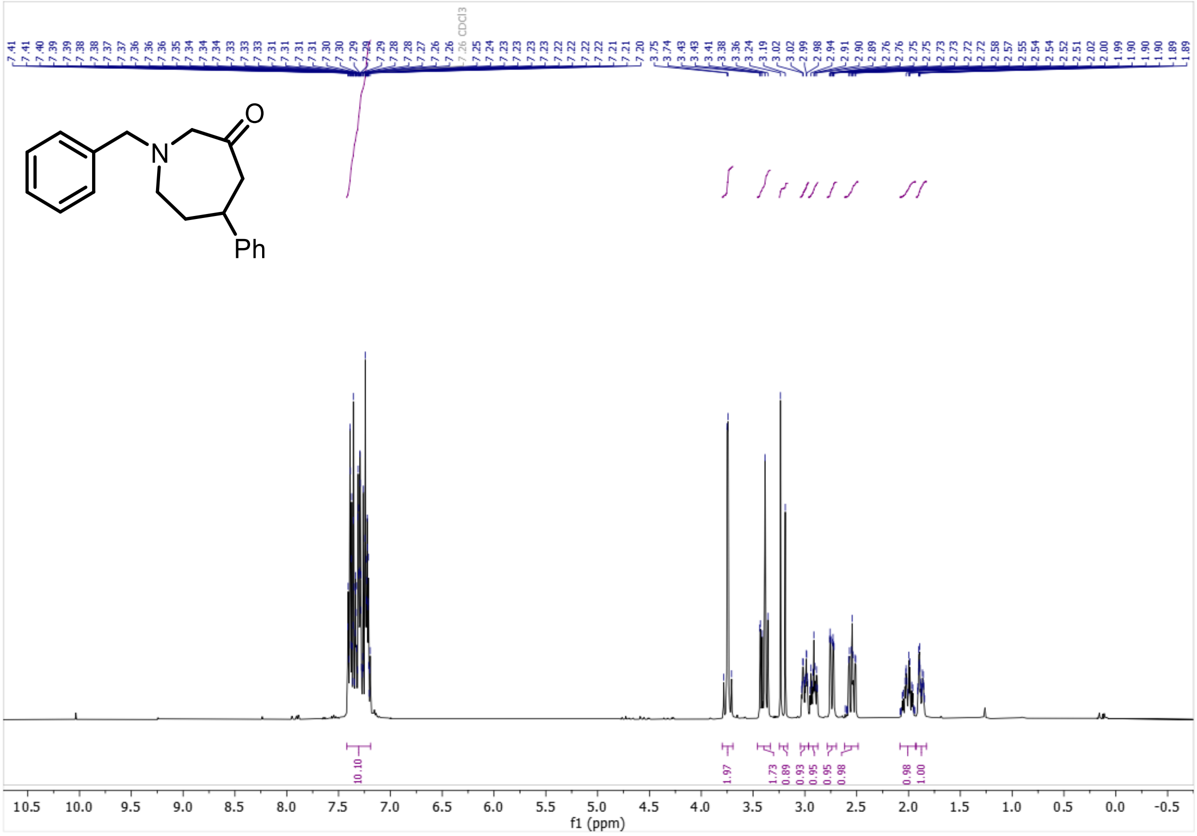

Compound 3l (^{13}C)

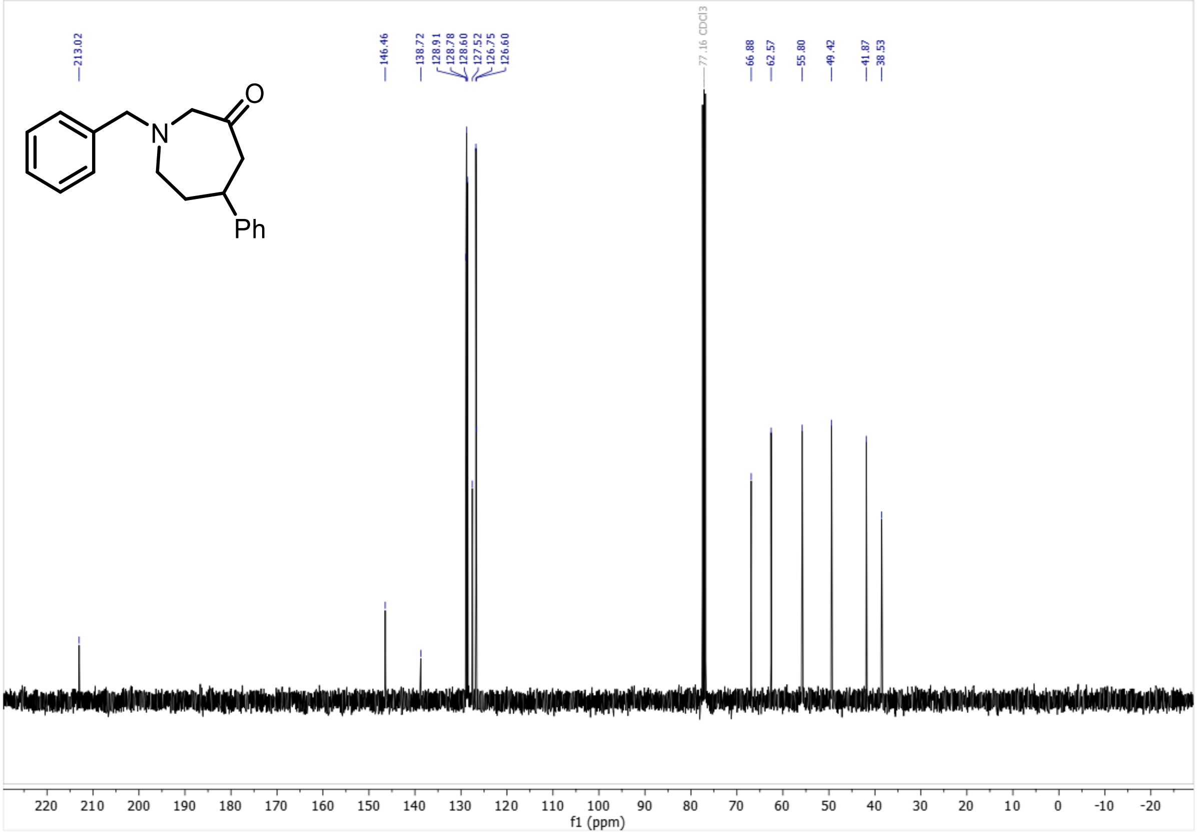

Compound 3m (^{1}H)

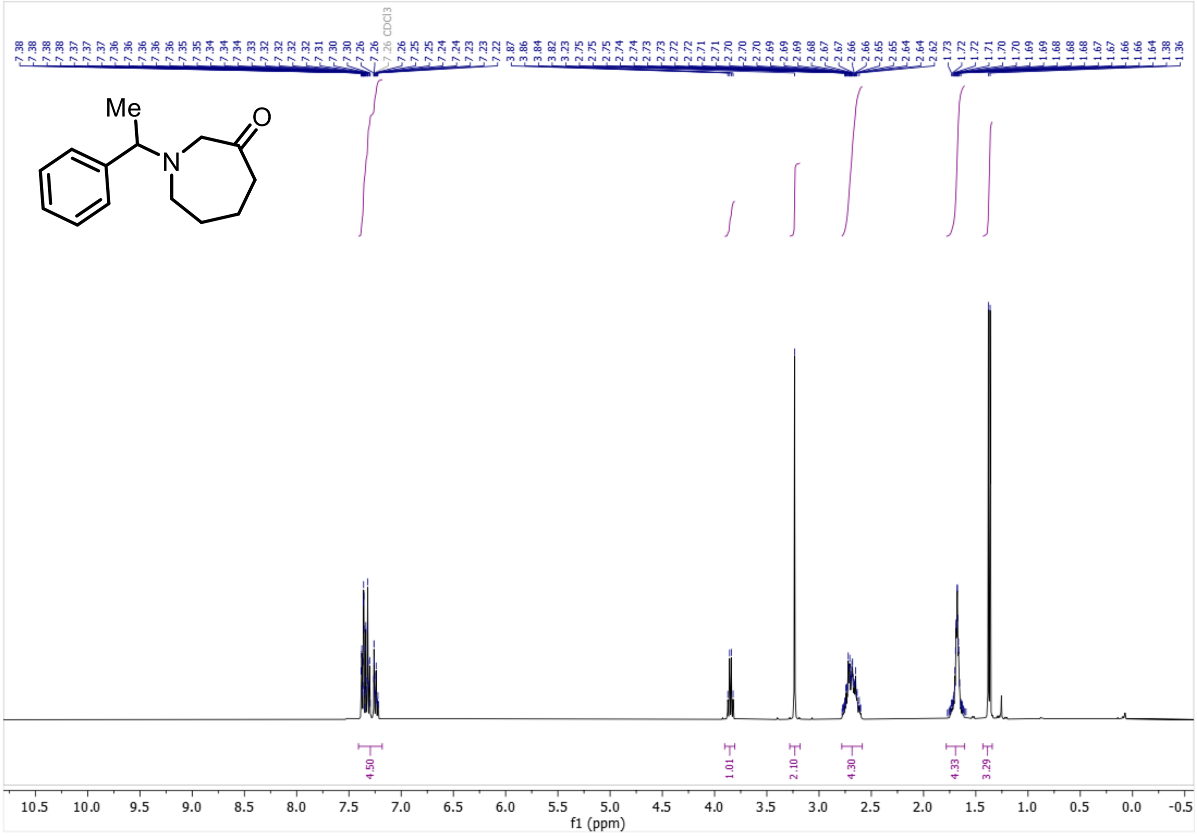

Compound 3m (^{13}C)

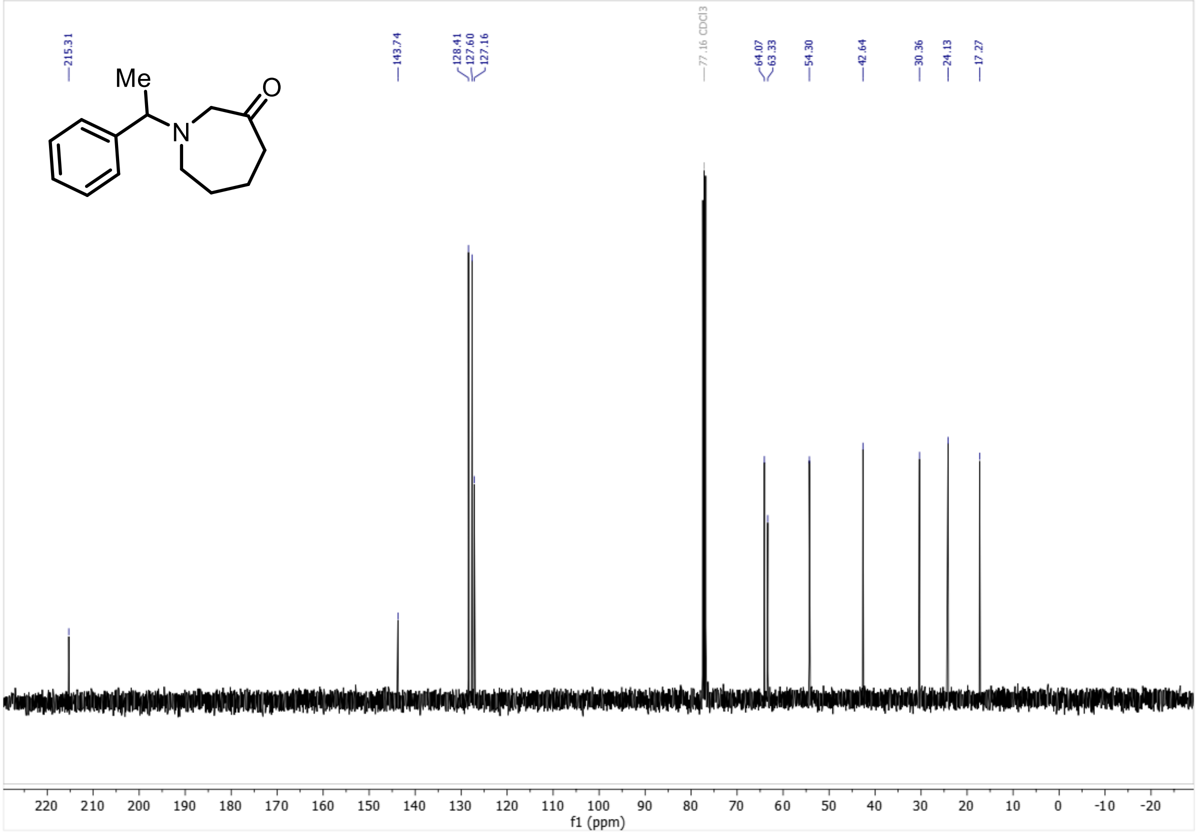

Compound 3n (^{1}H)

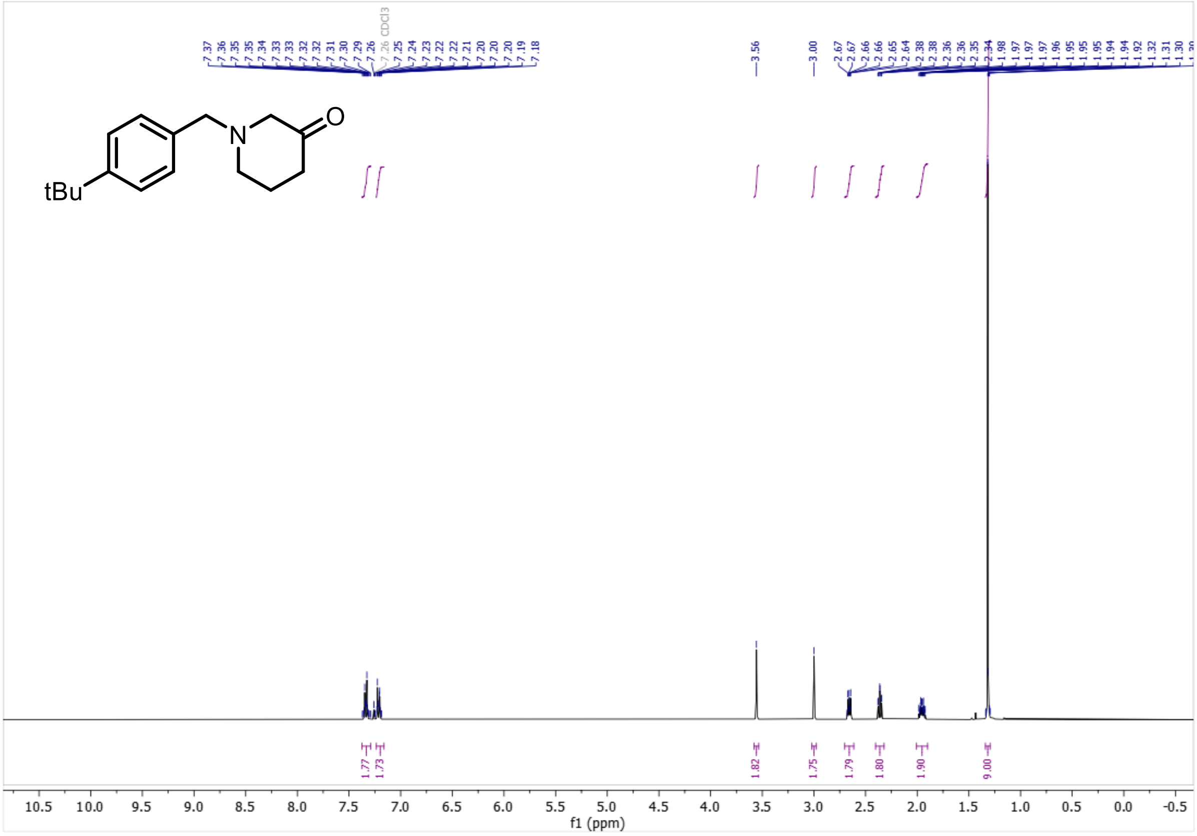

Compound 3n (^{13}C)

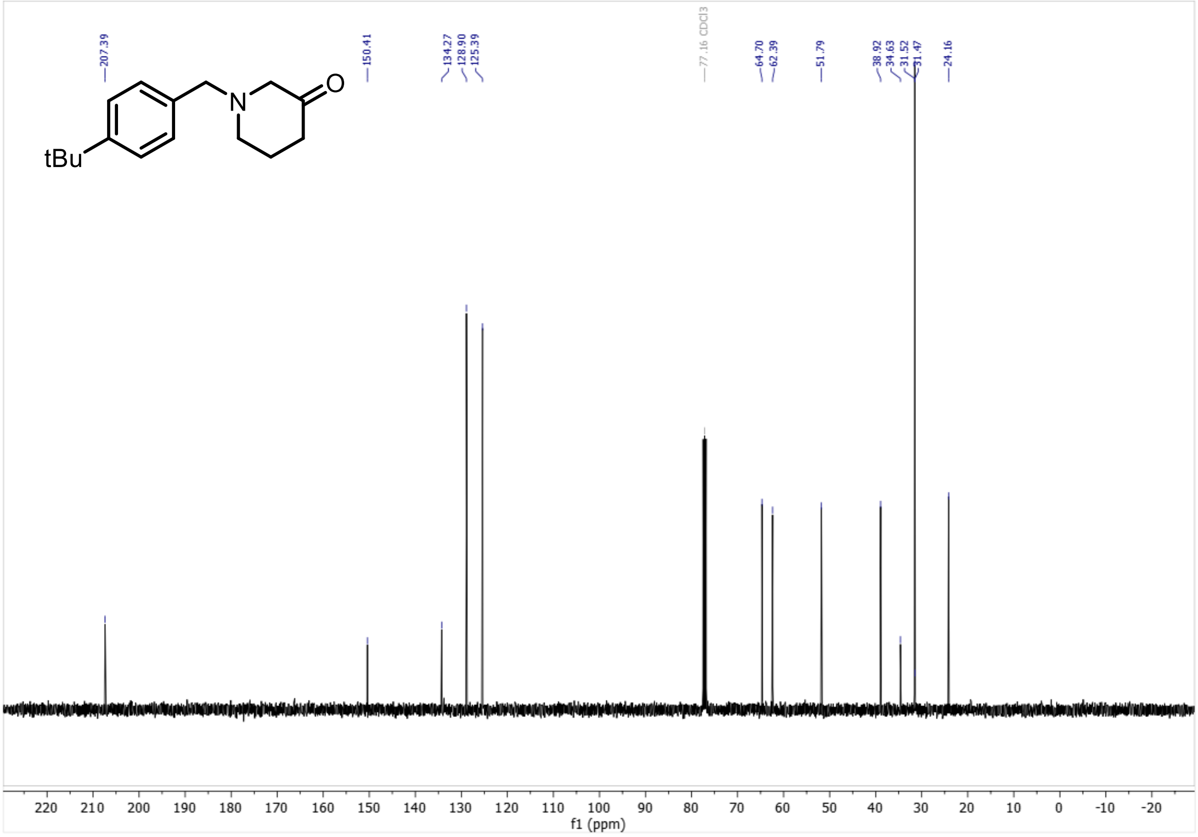

Compound 3p (^{1}H)

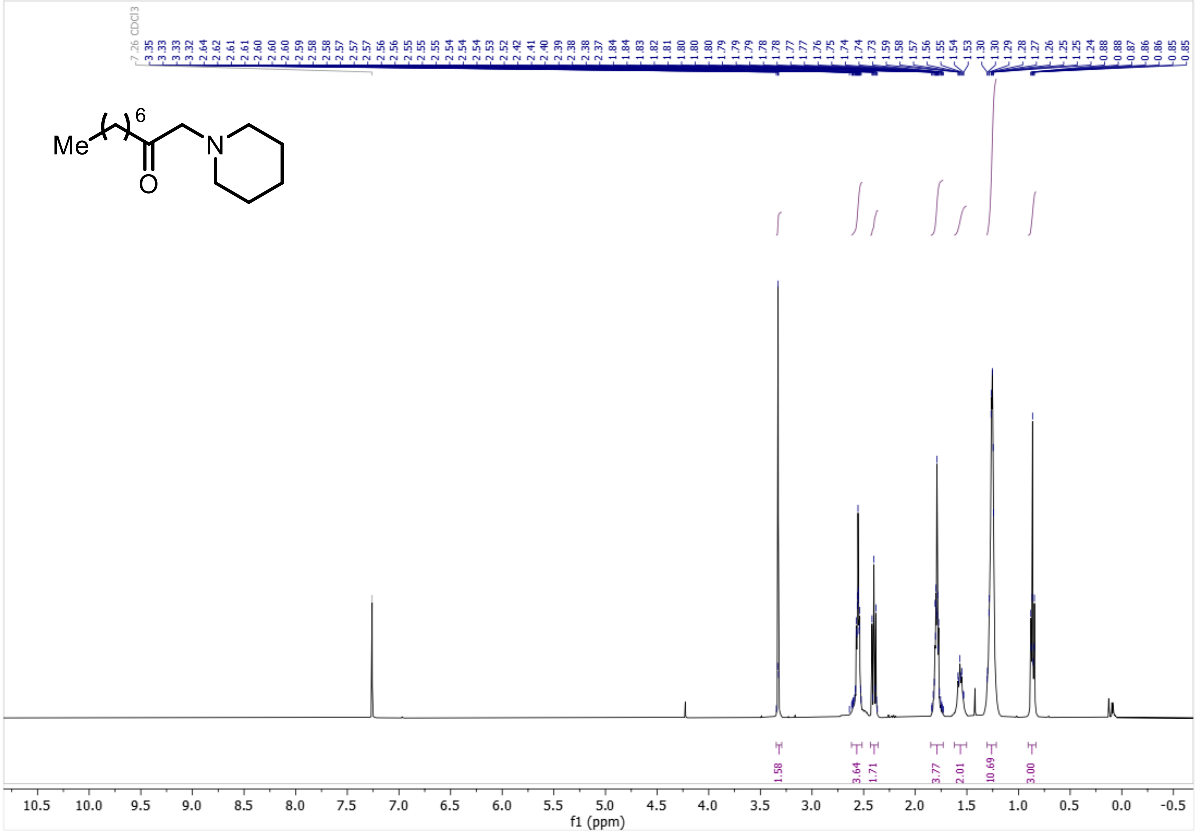

Compound 3p (^{13}C)

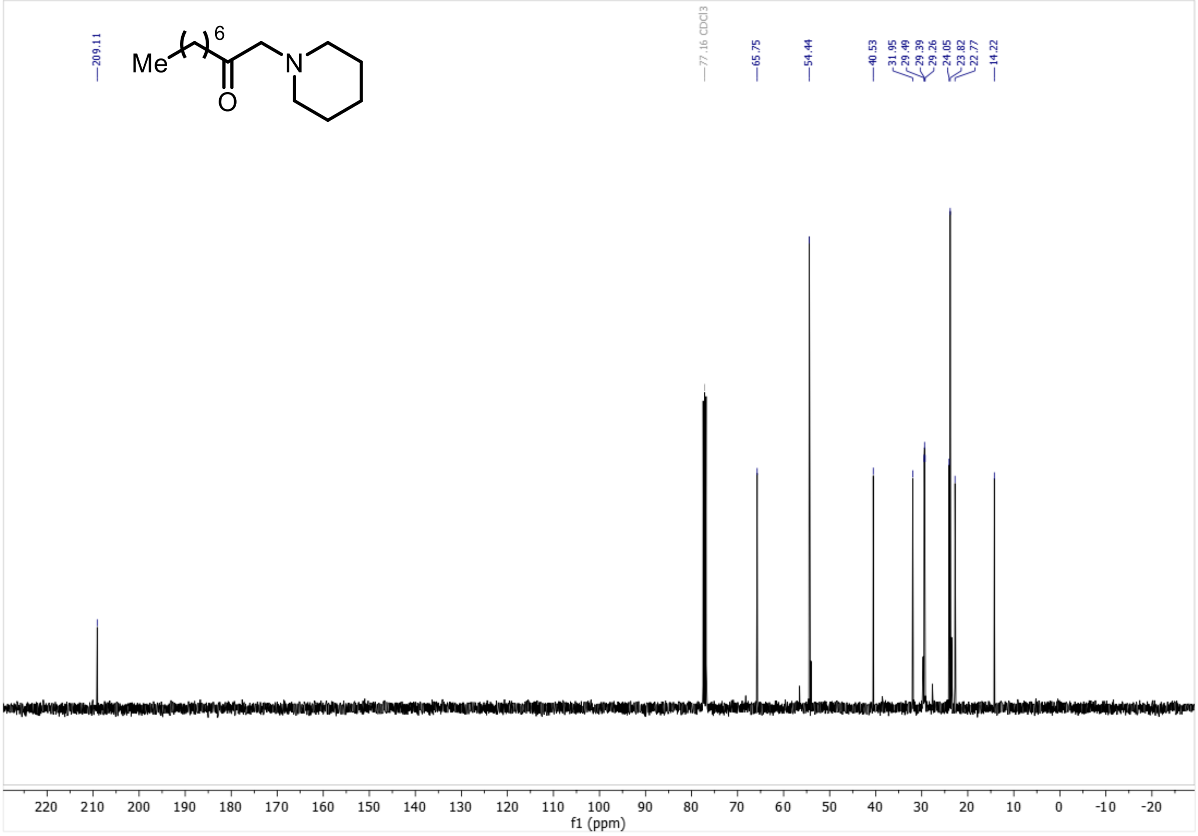

Compound 3q (^{1}H)

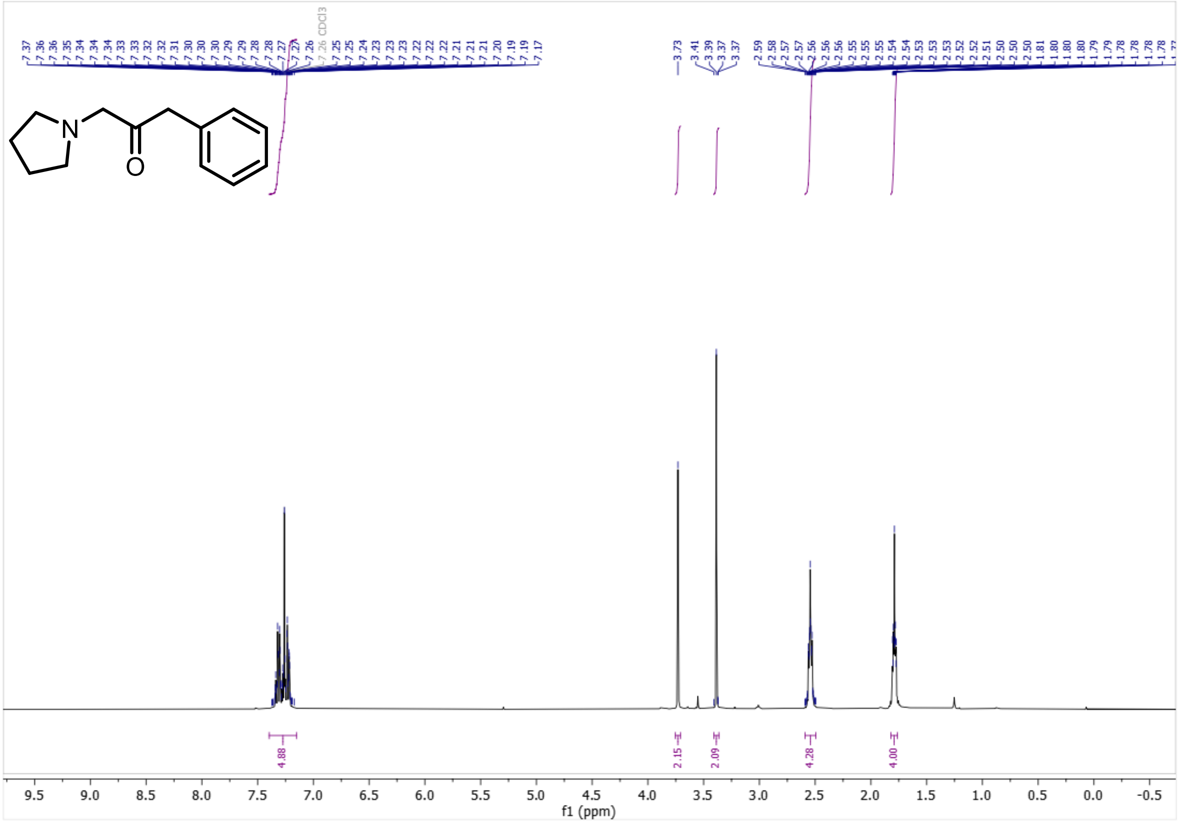

Compound 3q (^{13}C)

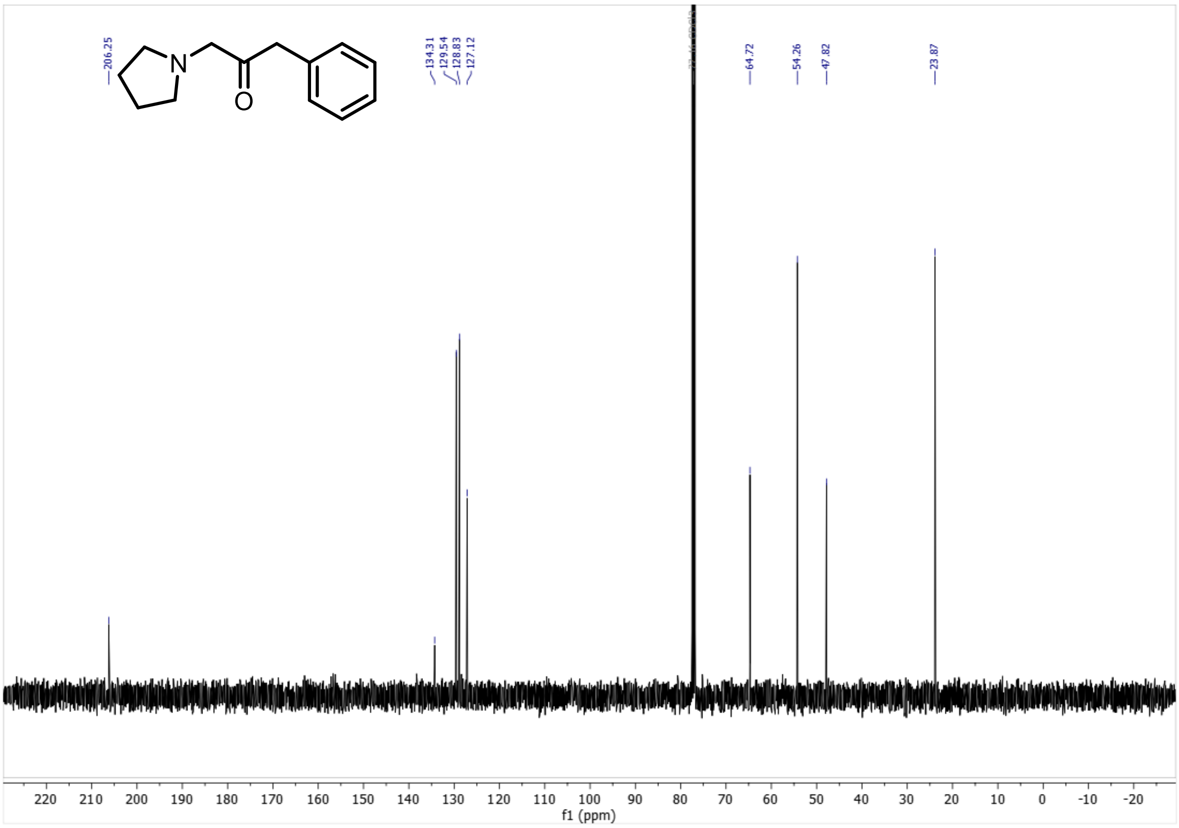

Compound 3r (^{1}H)

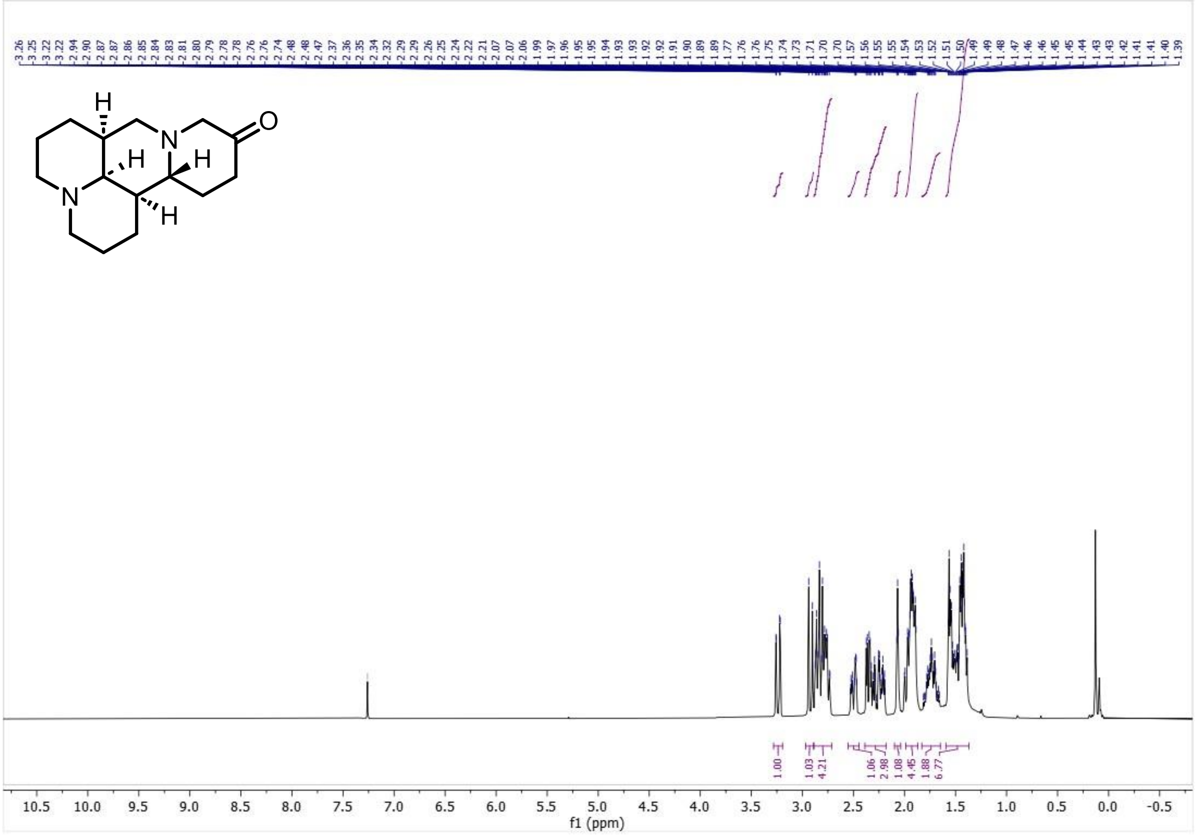

Compound 3r (^{13}C)

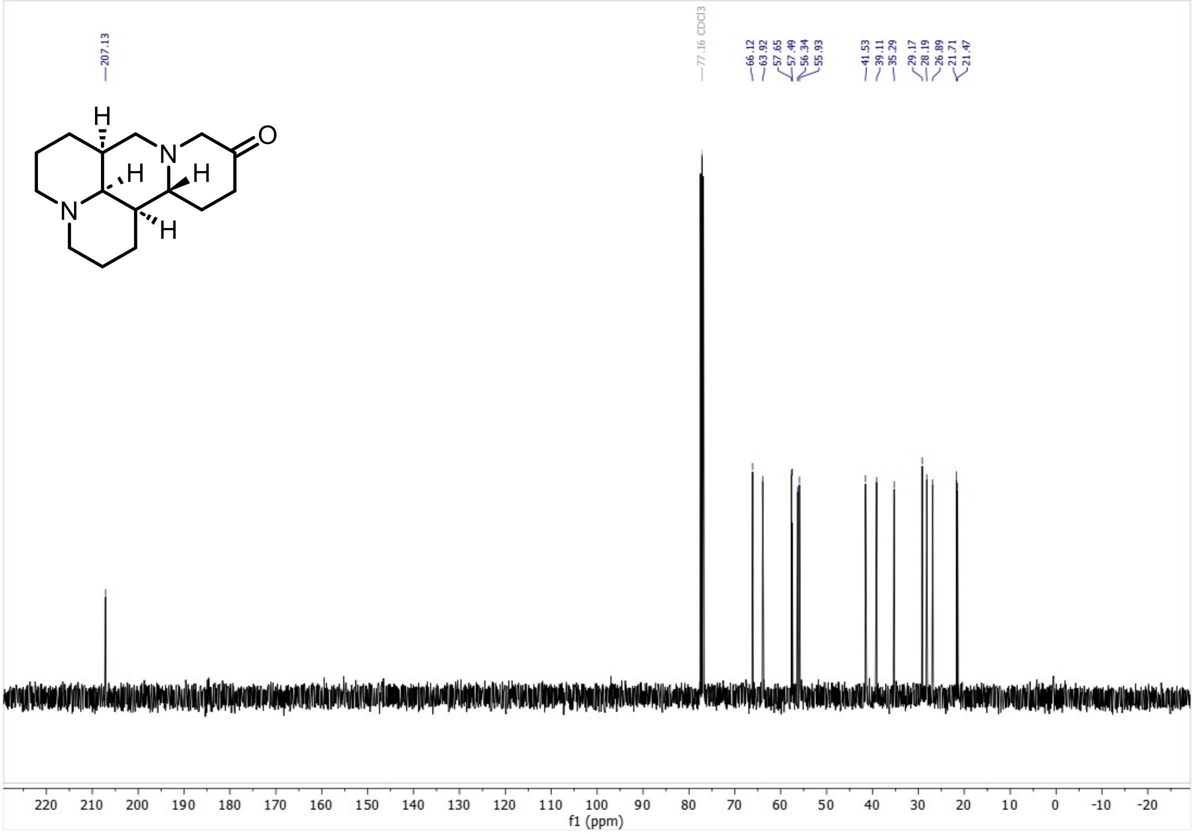

Compound 3s (^{1}H)

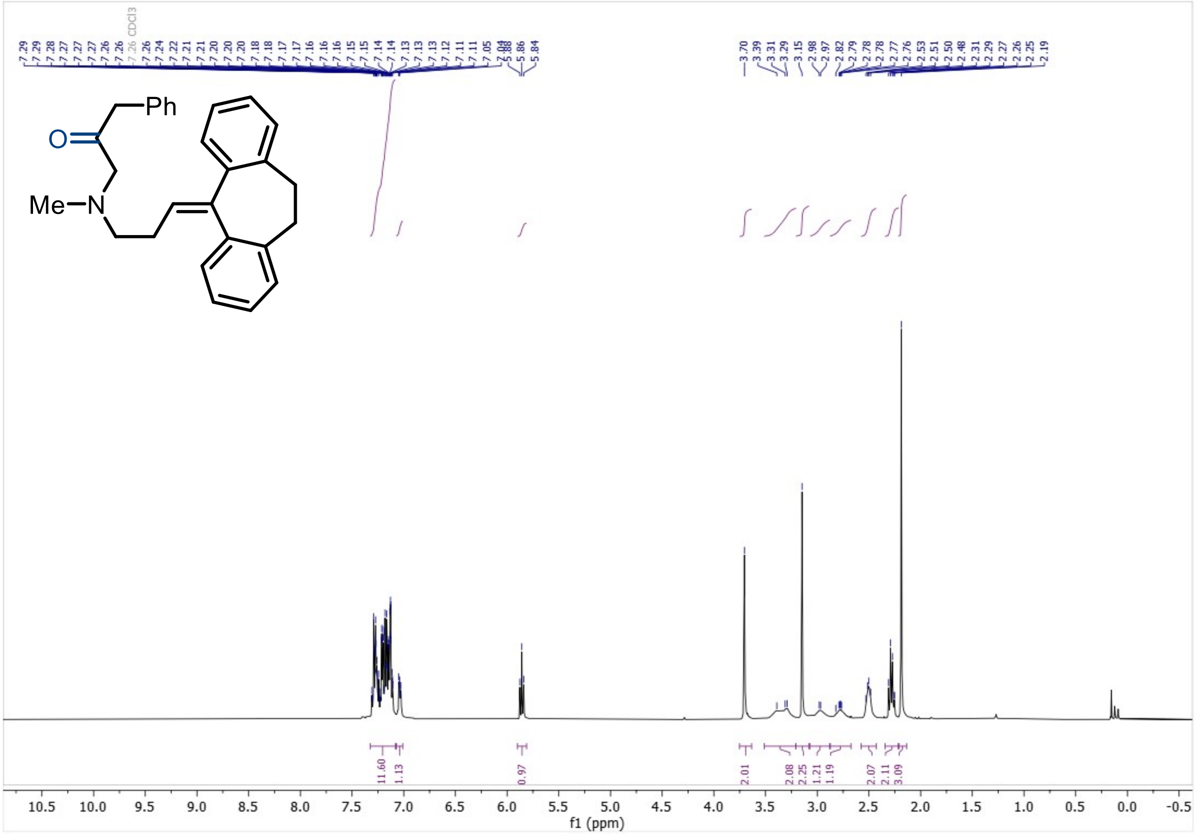

Compound 3s (^{13}C)

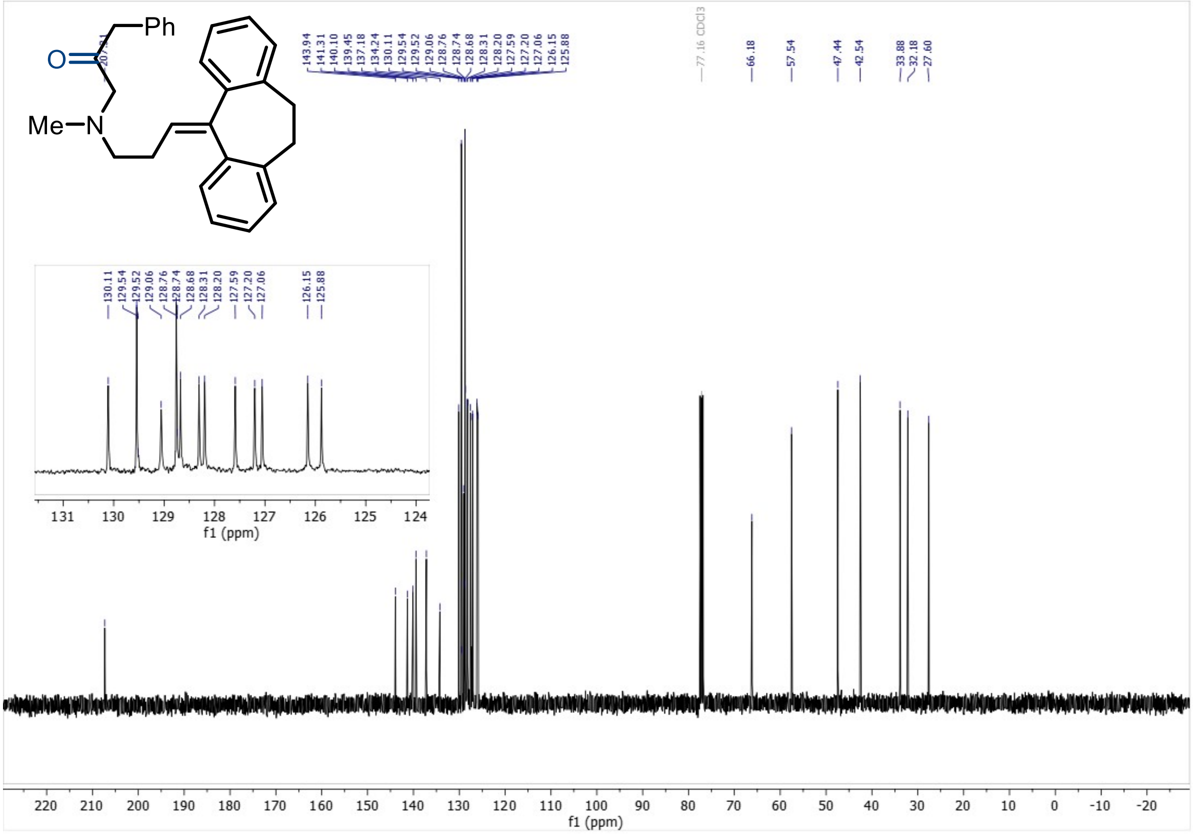

Compound 3t (^{1}H)

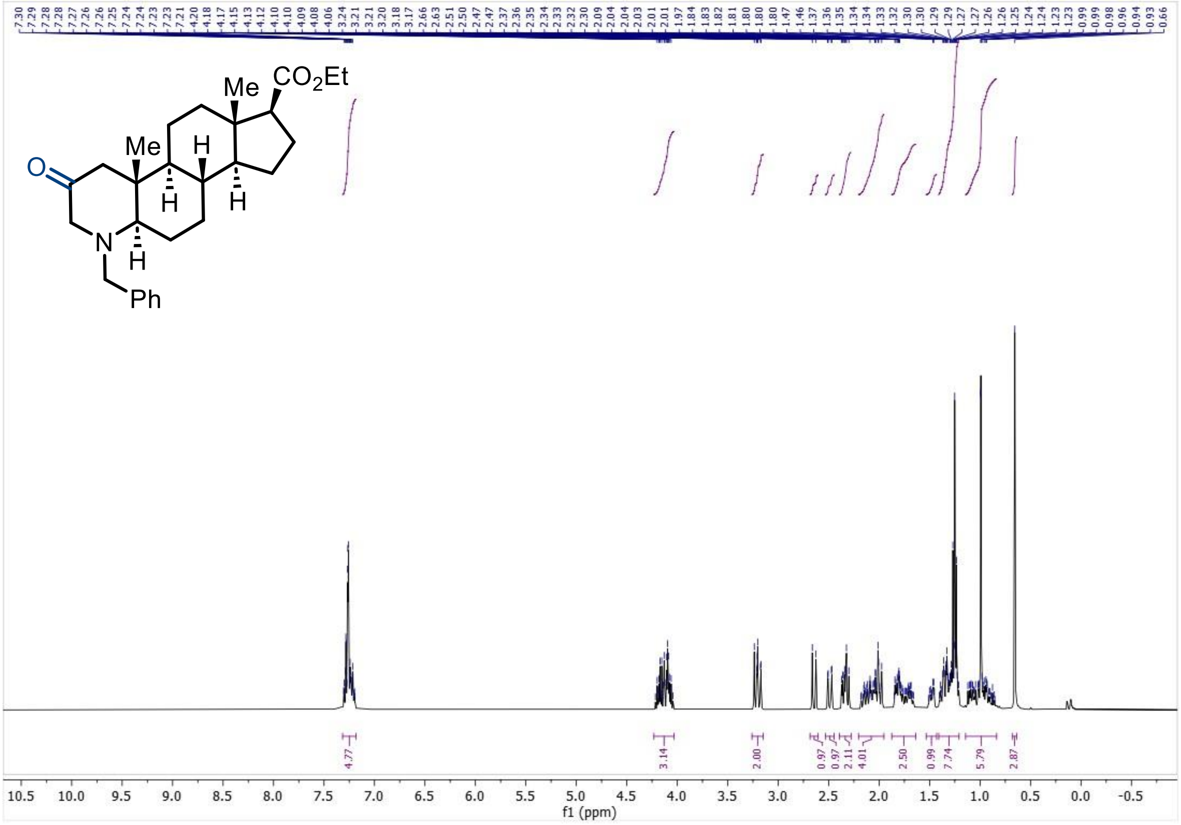

Compound 3t (^{13}C)

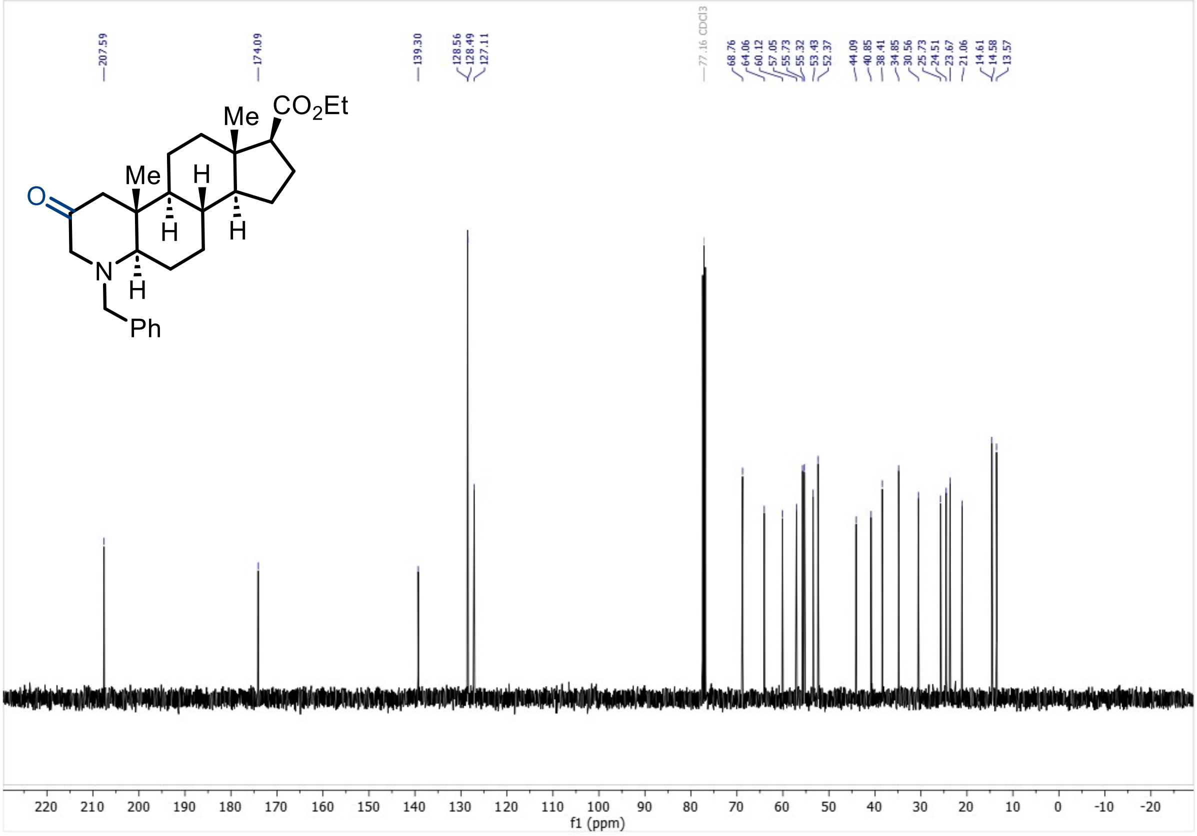

Compound 4a (^{1}H)

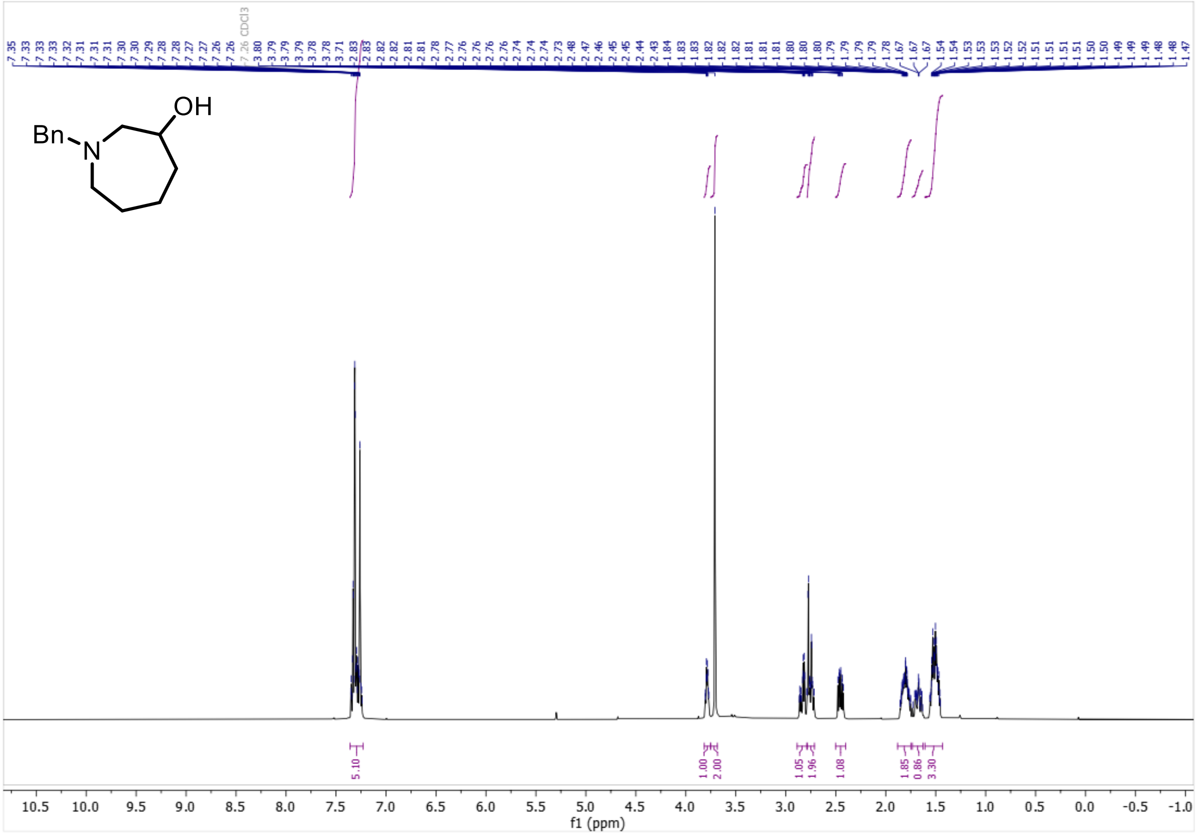

Compound 4a (^{13}C)

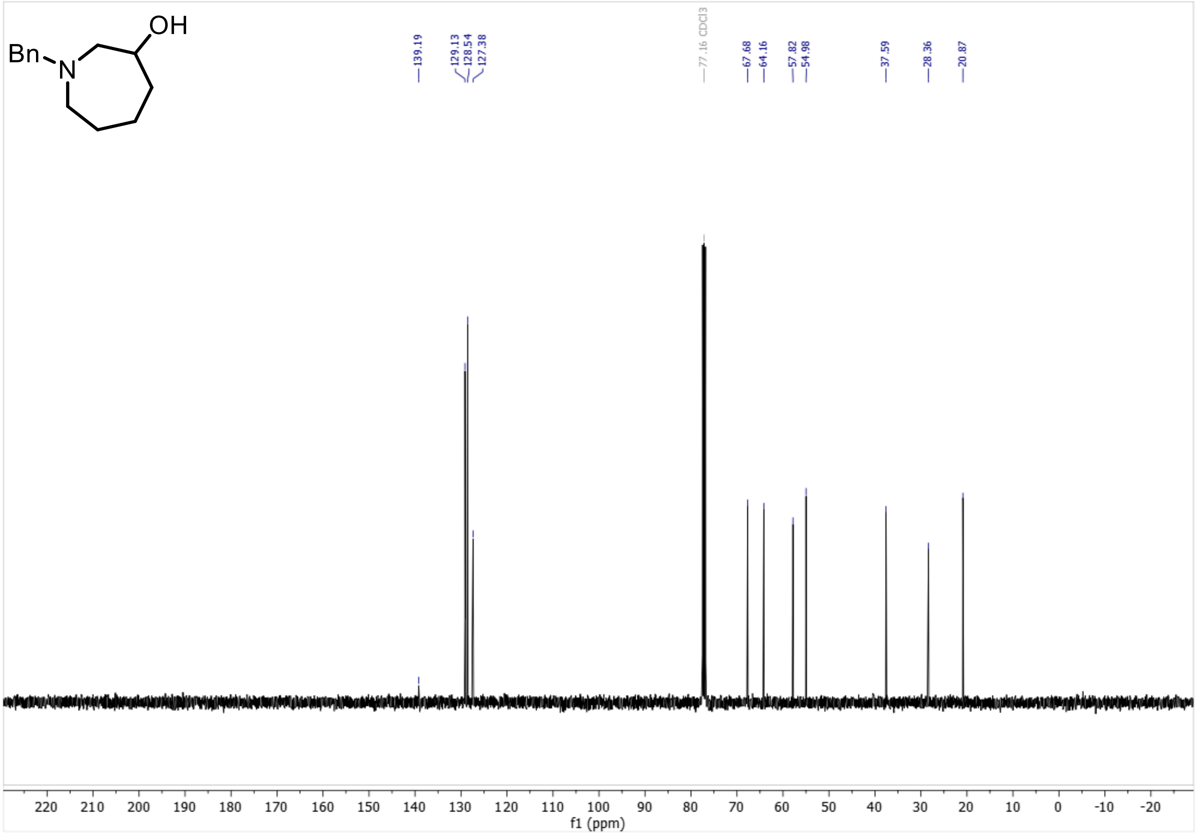

Compound 4b (^{1}H)

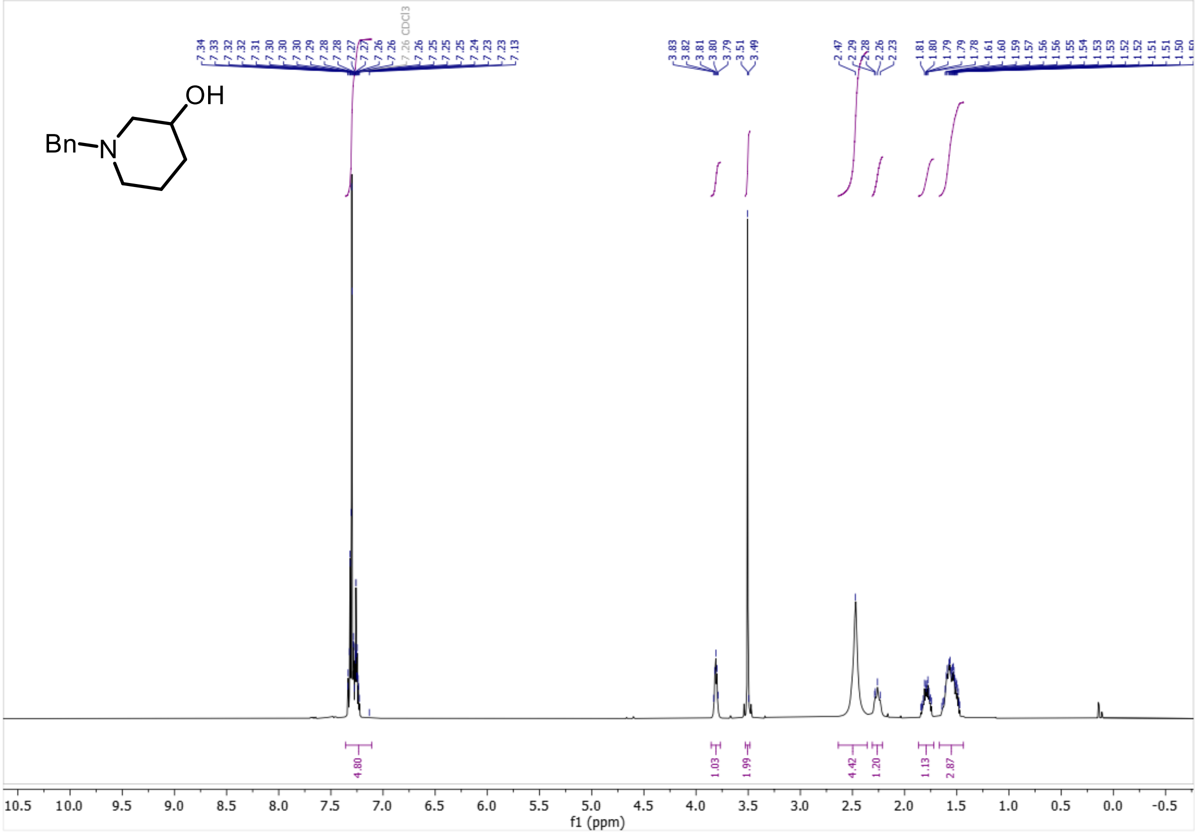

Compound 4b (^{13}C)

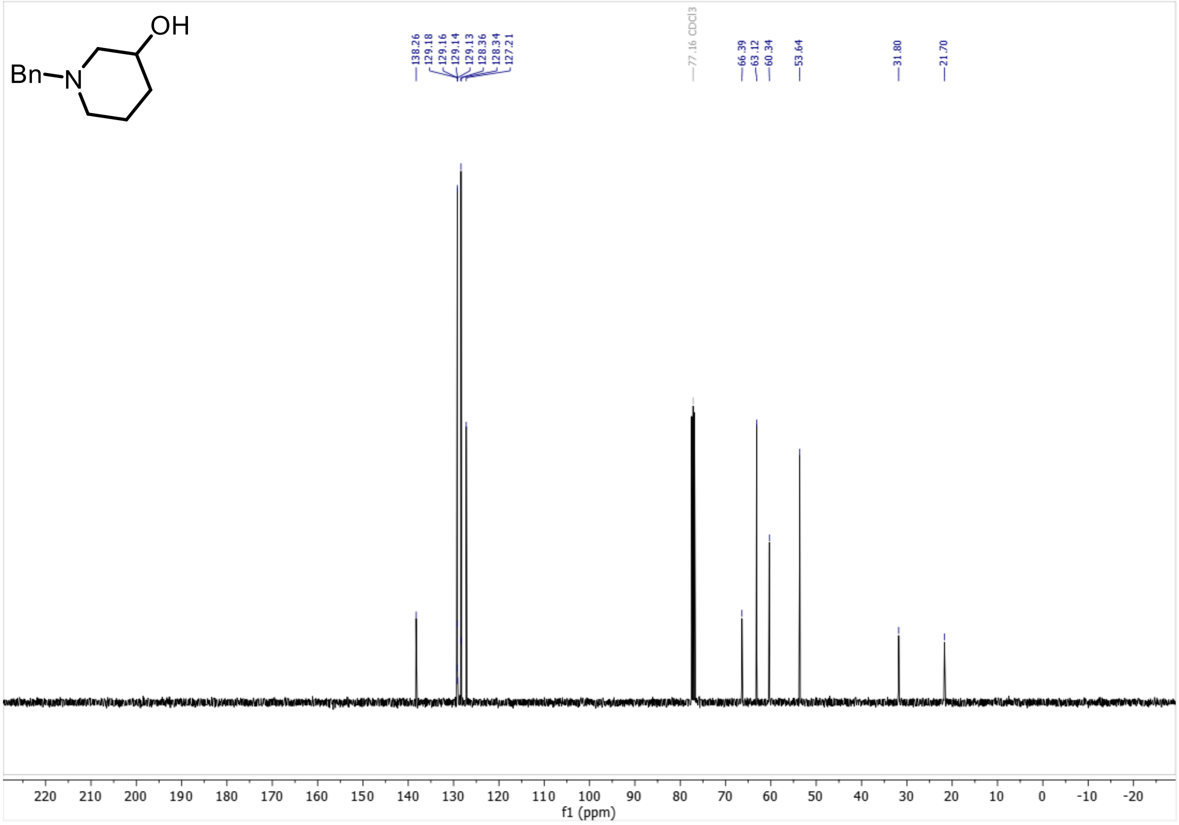

Compound 4c (^{1}H)

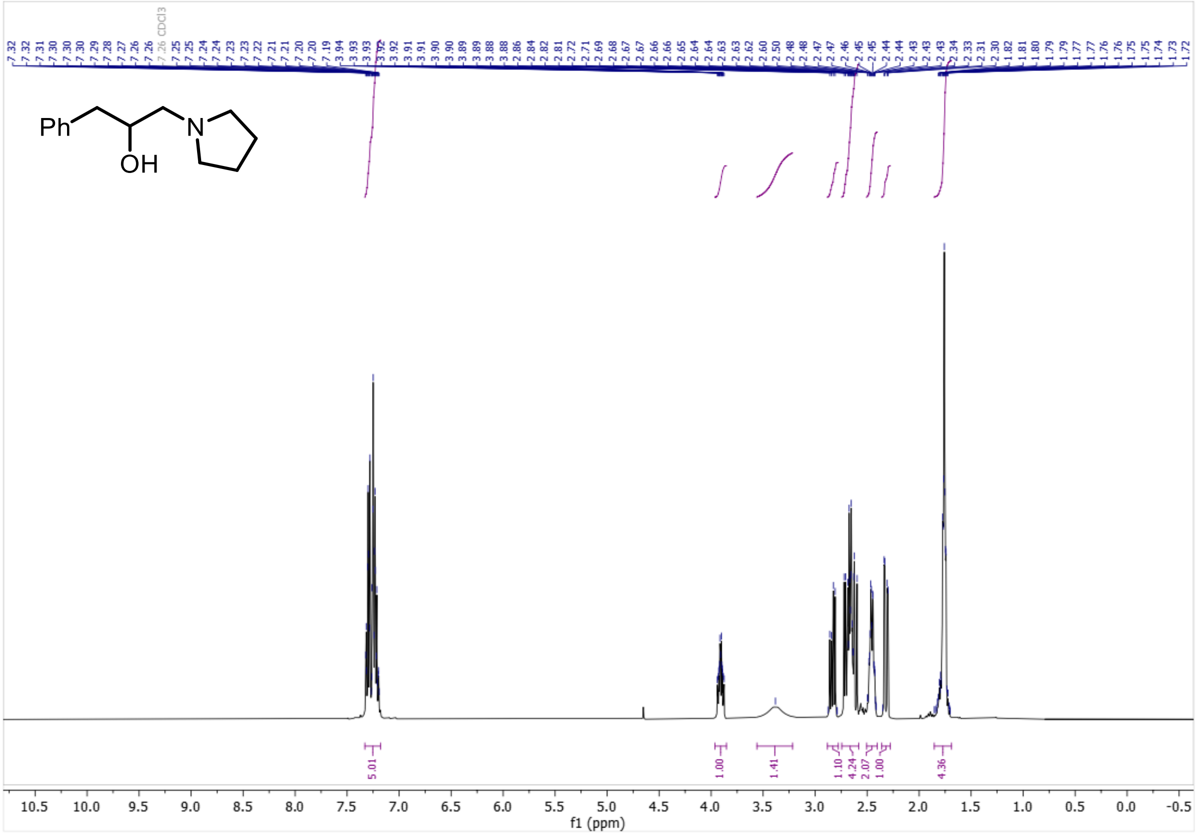

Compound 4c (^{13}C)

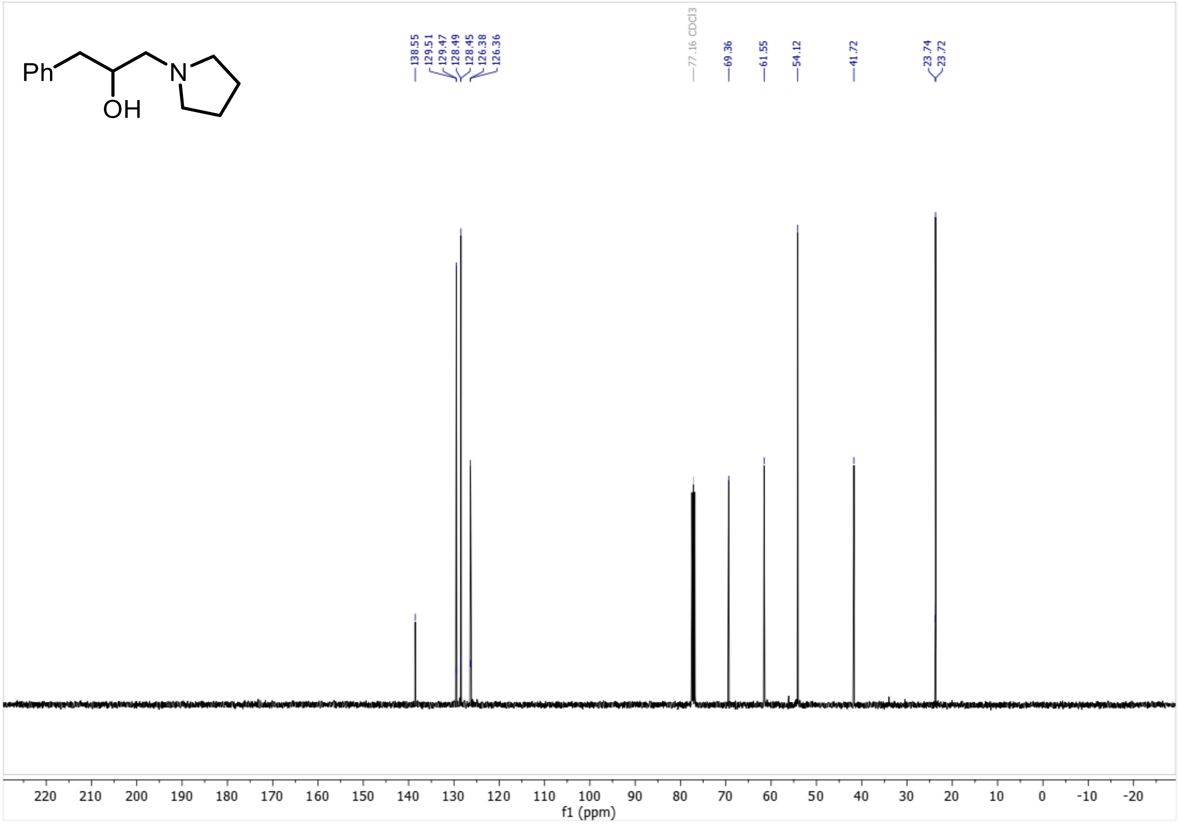

Compound 4d (^{1}H)

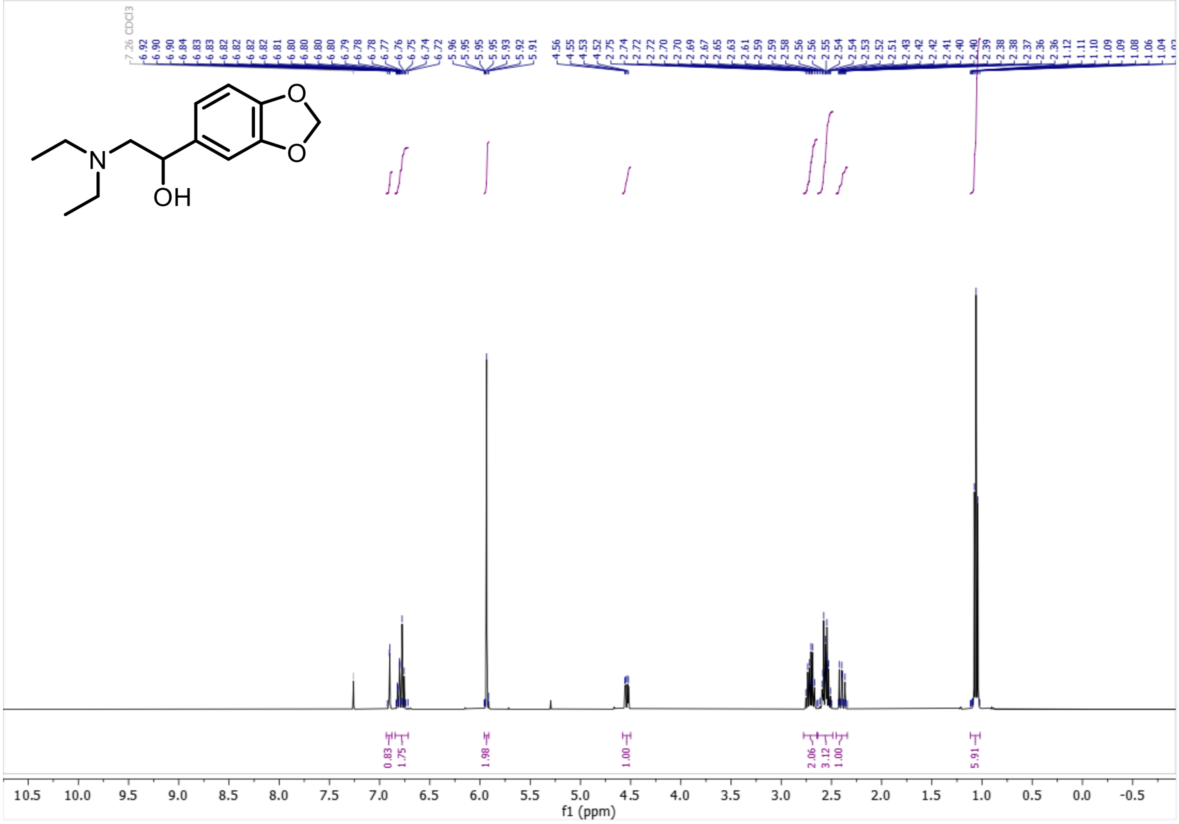

Compound 4d (^{13}C)

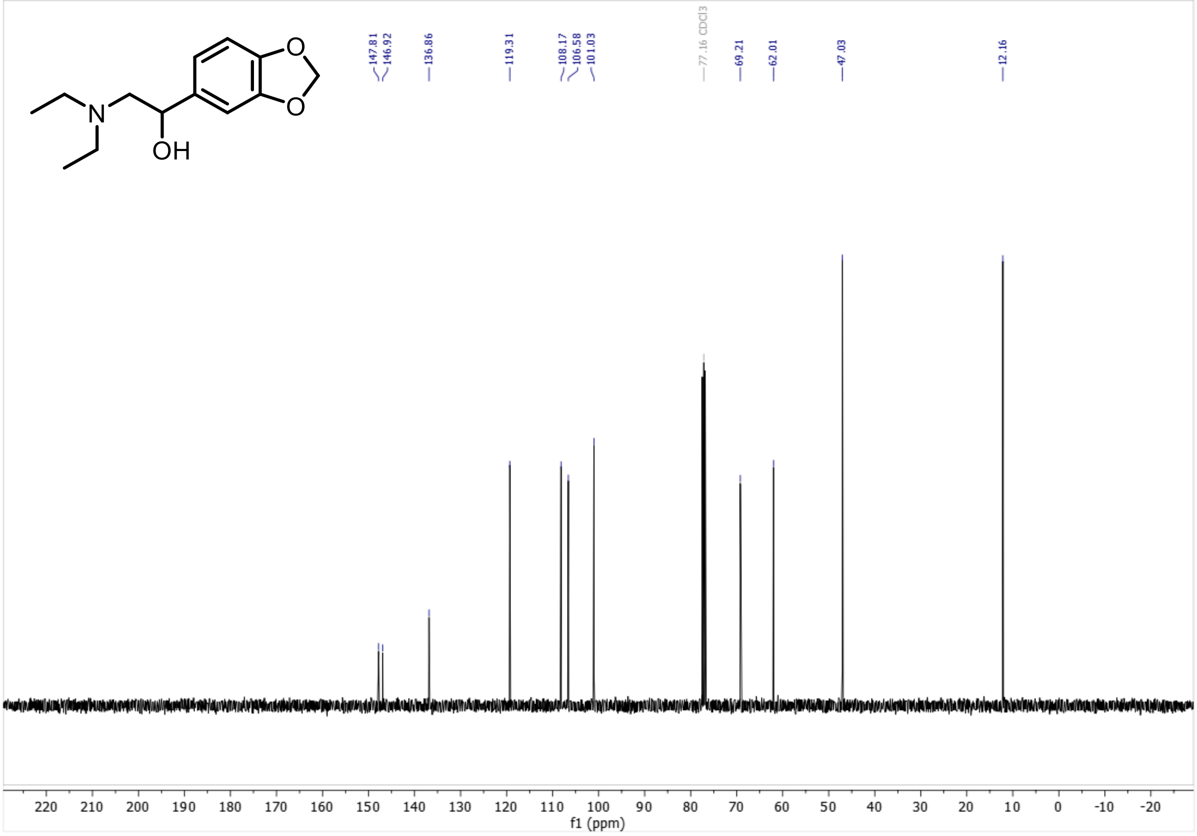

Compound 4e (^{1}H)

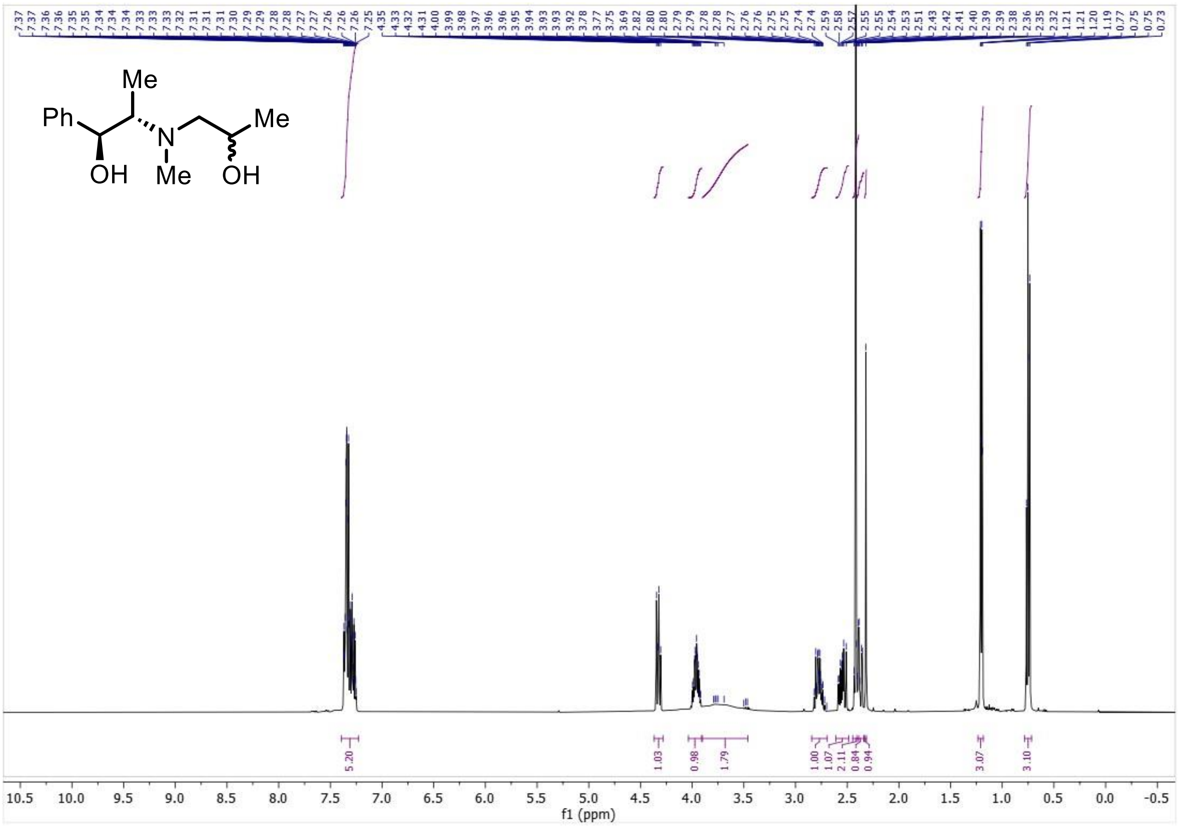

Compound 4e (^{13}C)

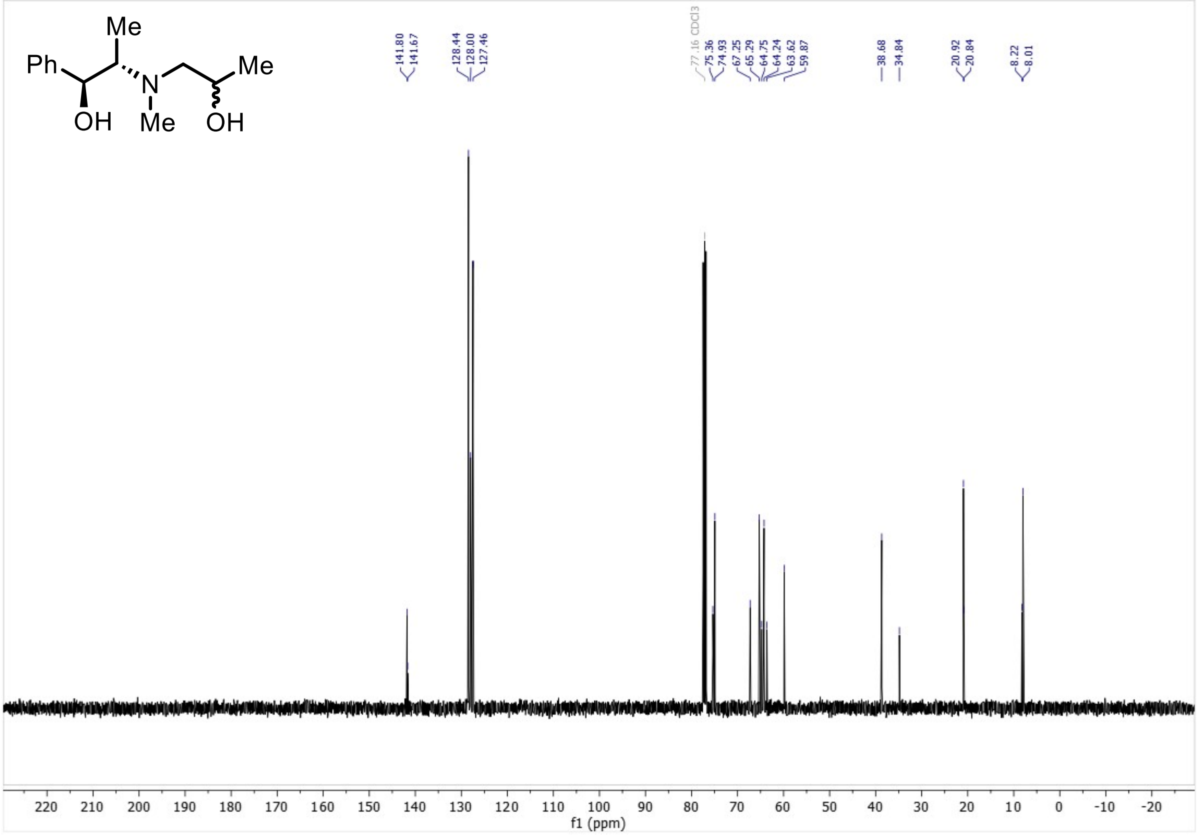

Compound 4f-A (^{1}H)

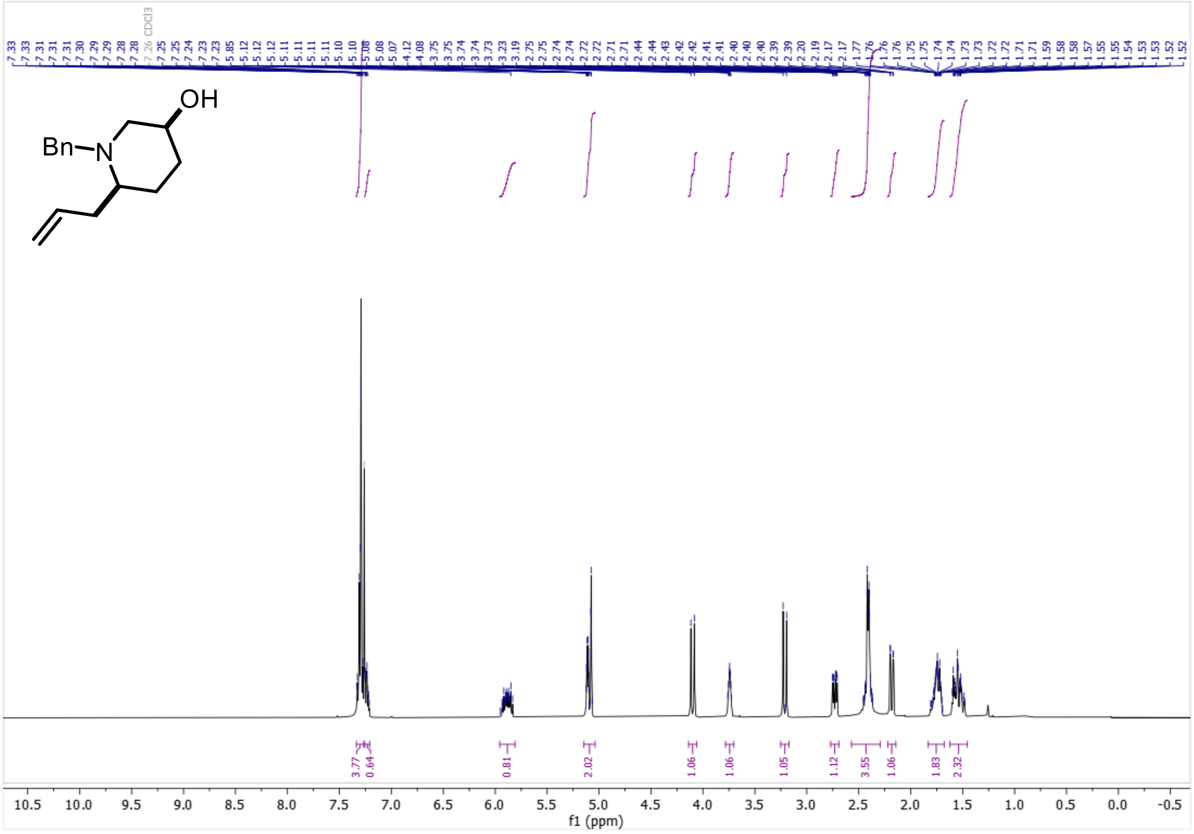

Compound 4f-A (^{13}C)

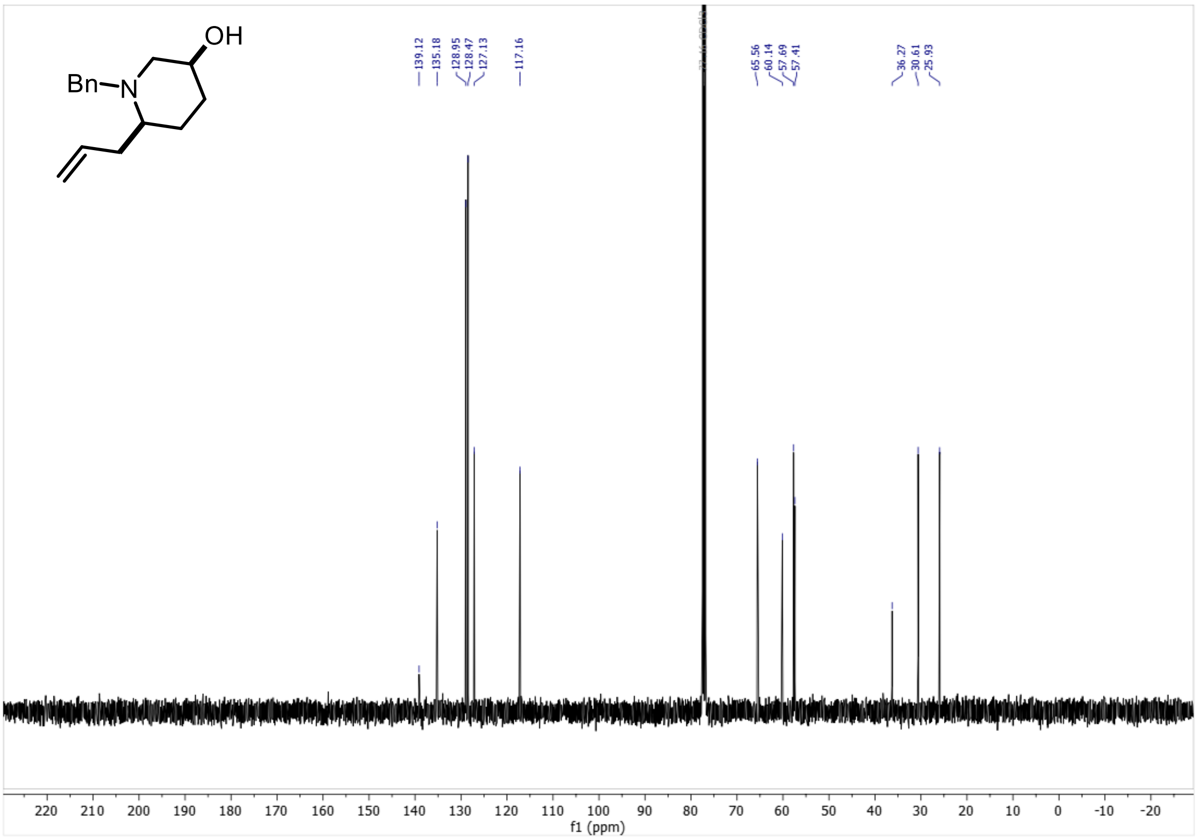

Compound 4f-B (^{1}H)

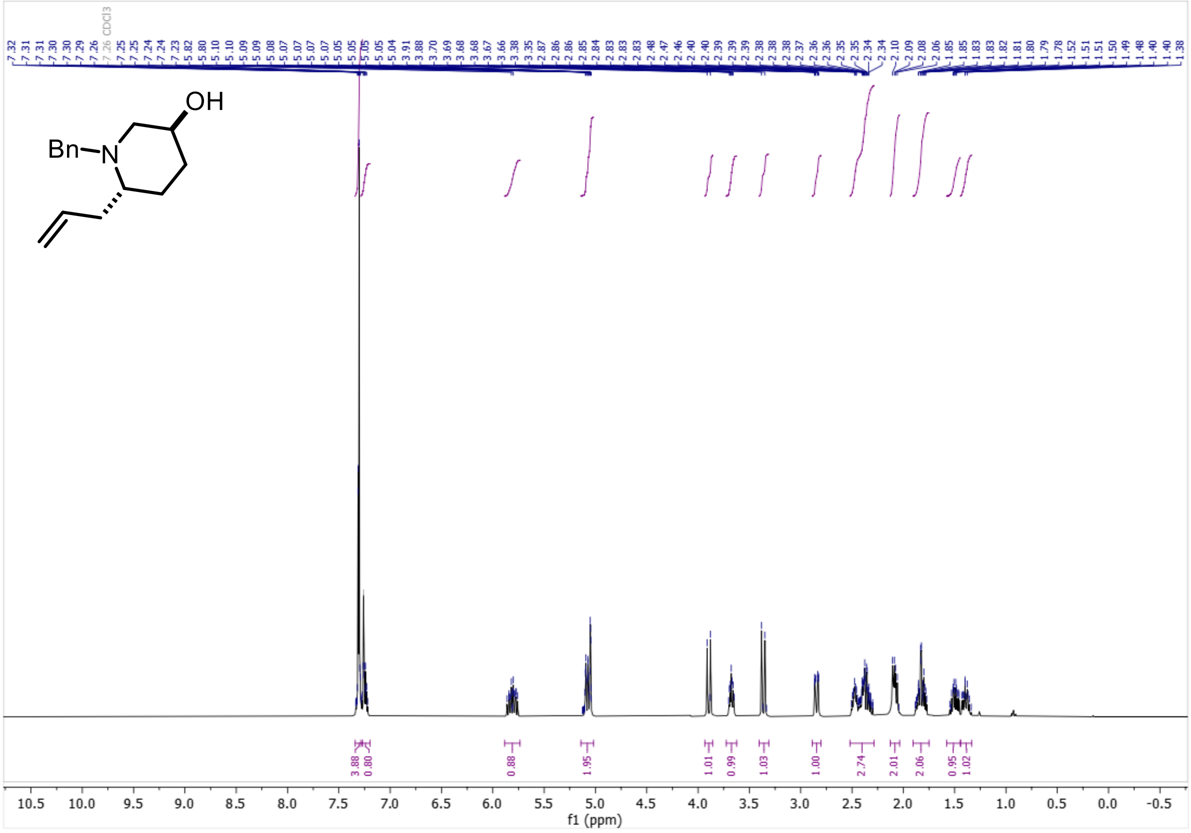

Compound 4f-B (^{13}C)

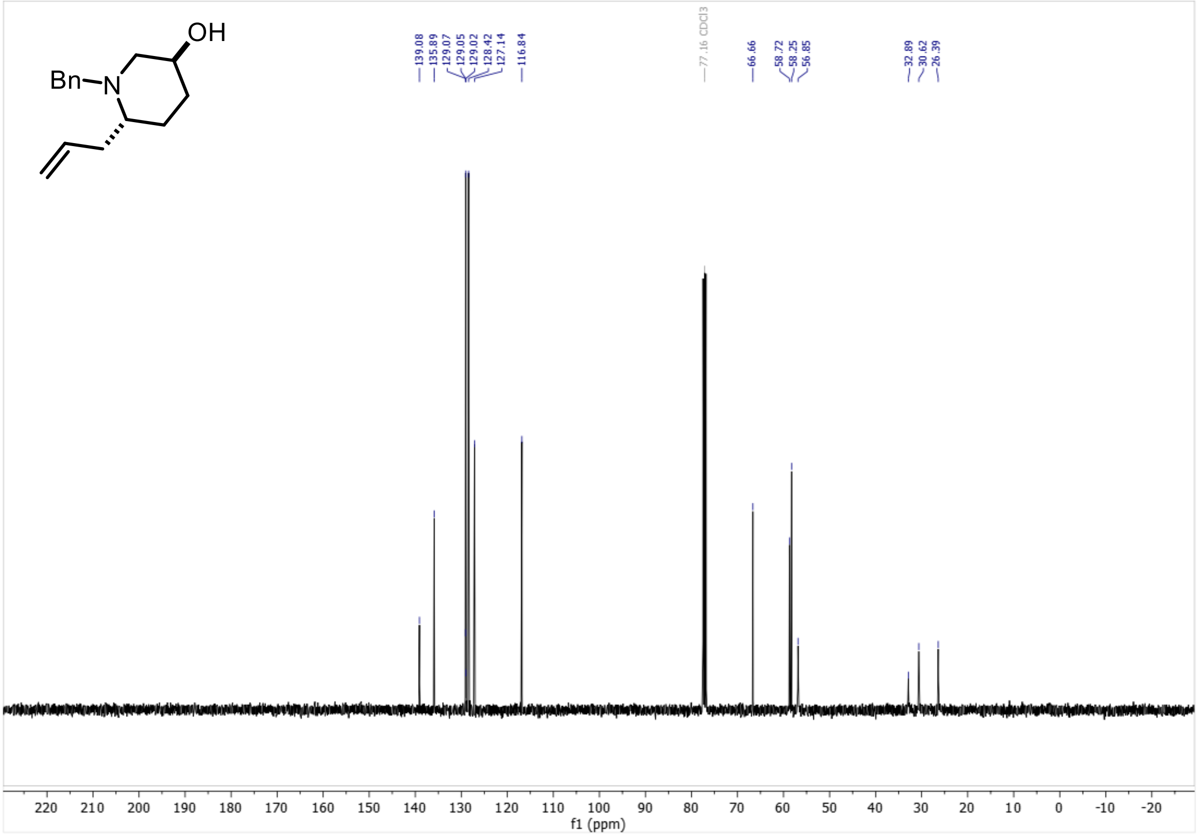

(±)-pseudoconhydrine (¹H)

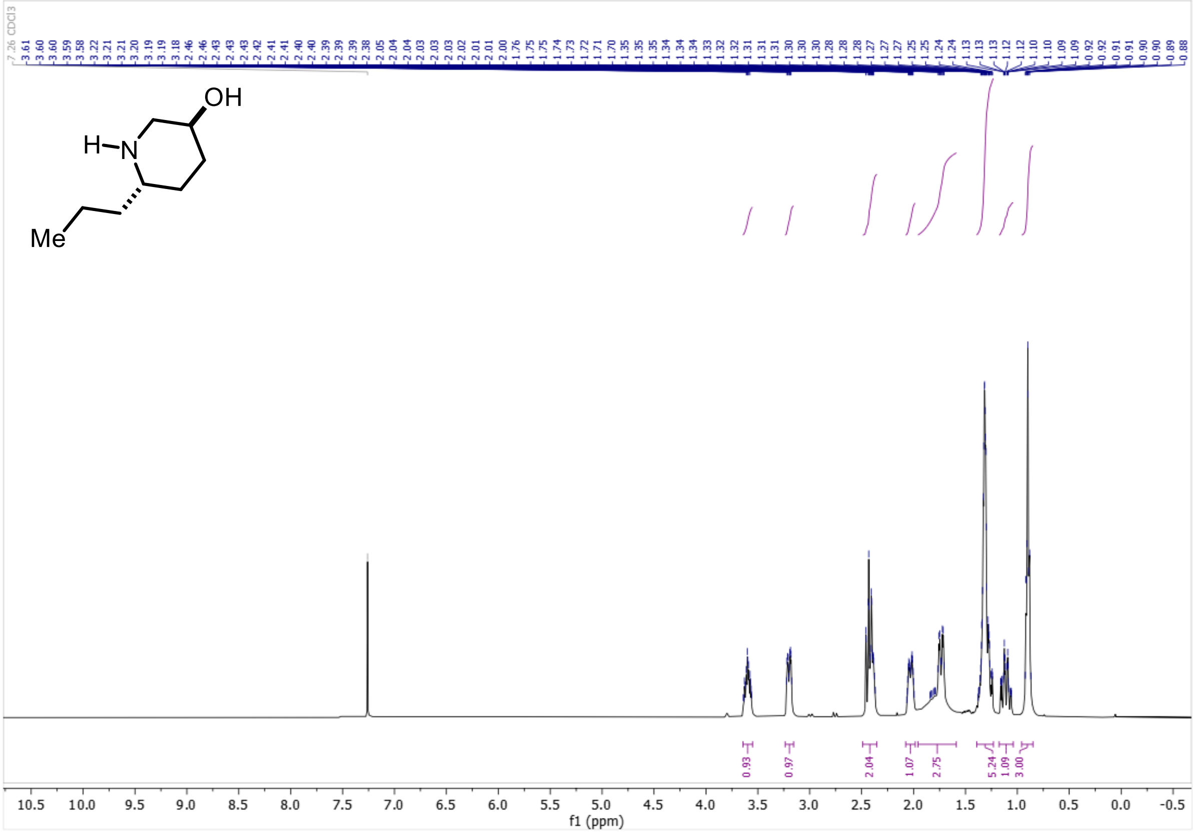

(±)-pseudoconhydrine (¹³C)

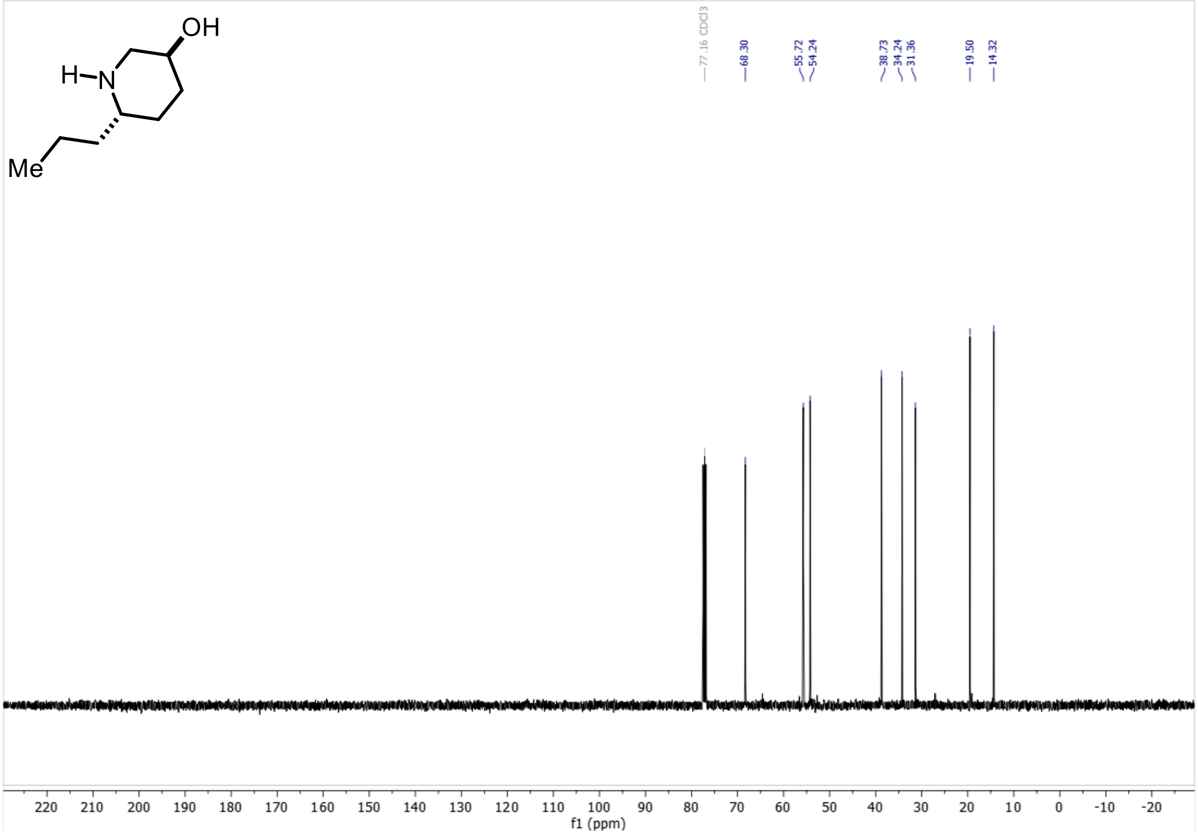

Compound 4g (^{1}H)

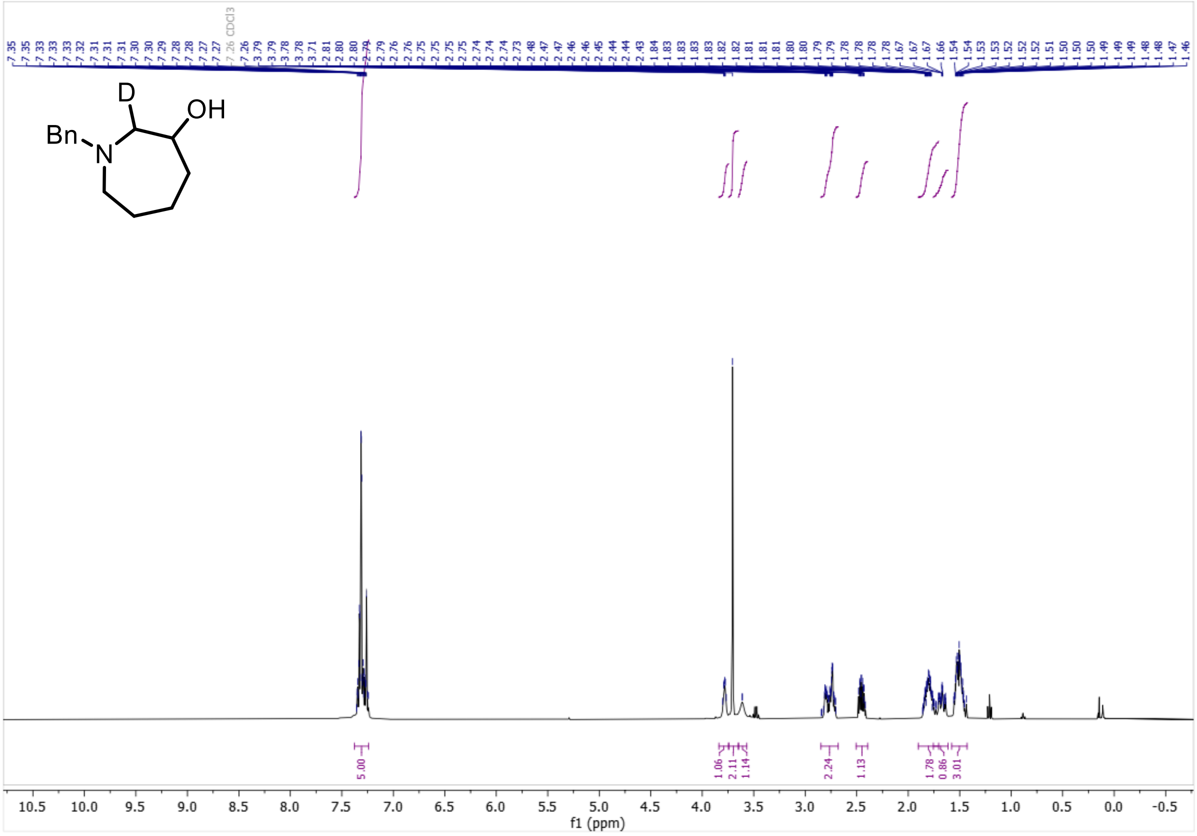

Compound 4g (^{13}C)

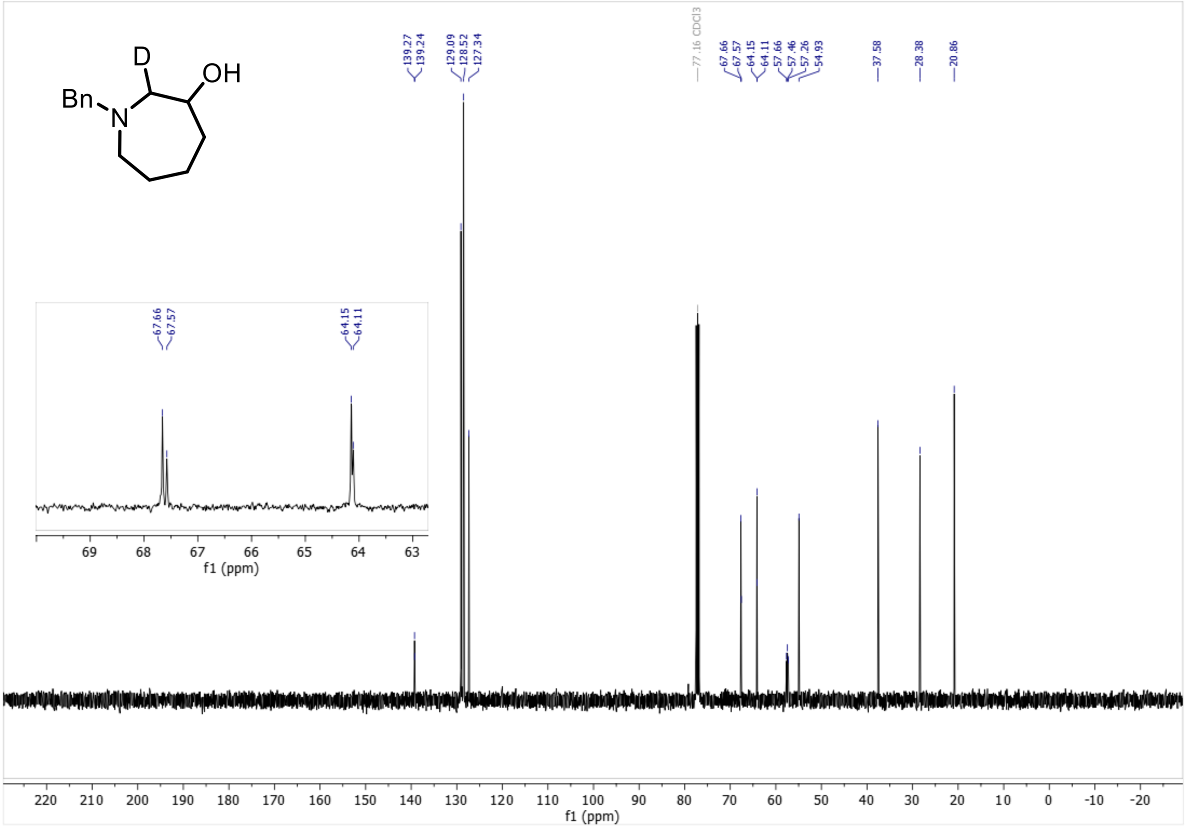

Compound 5a (¹H)

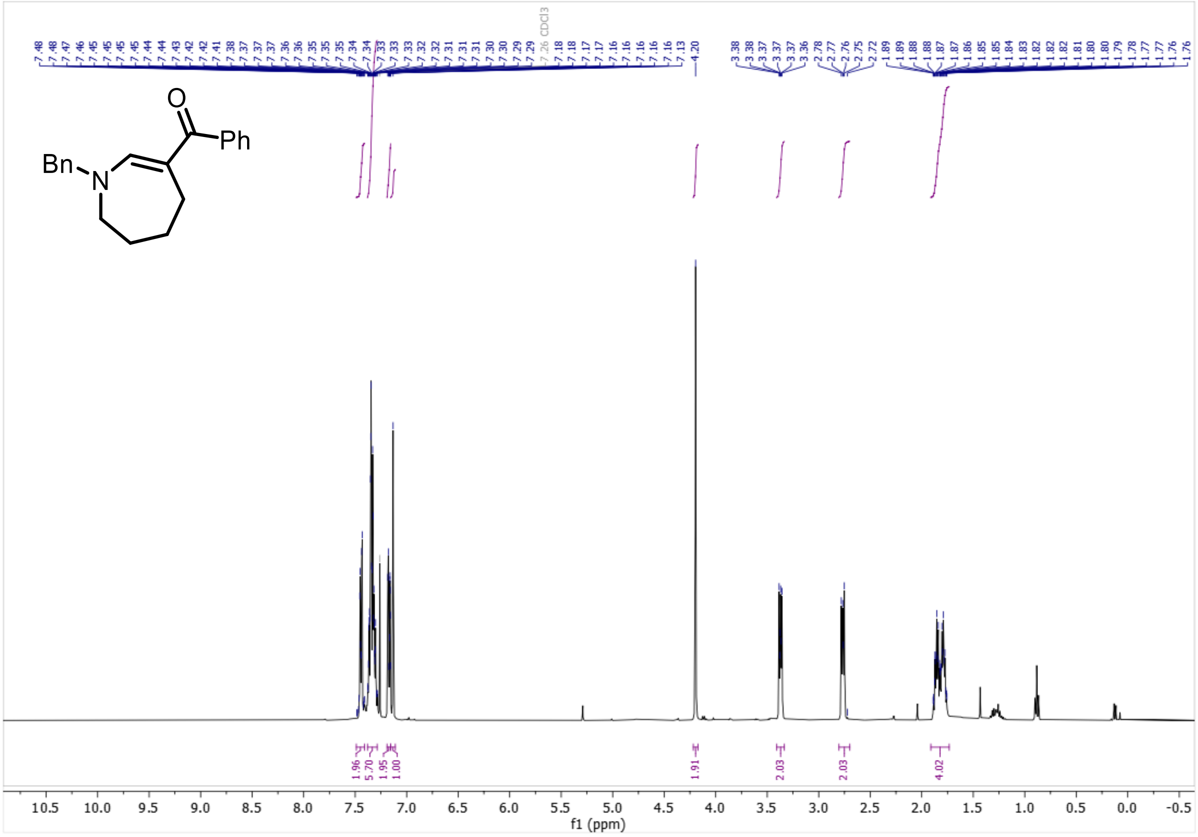

Compound 5a (¹³C)

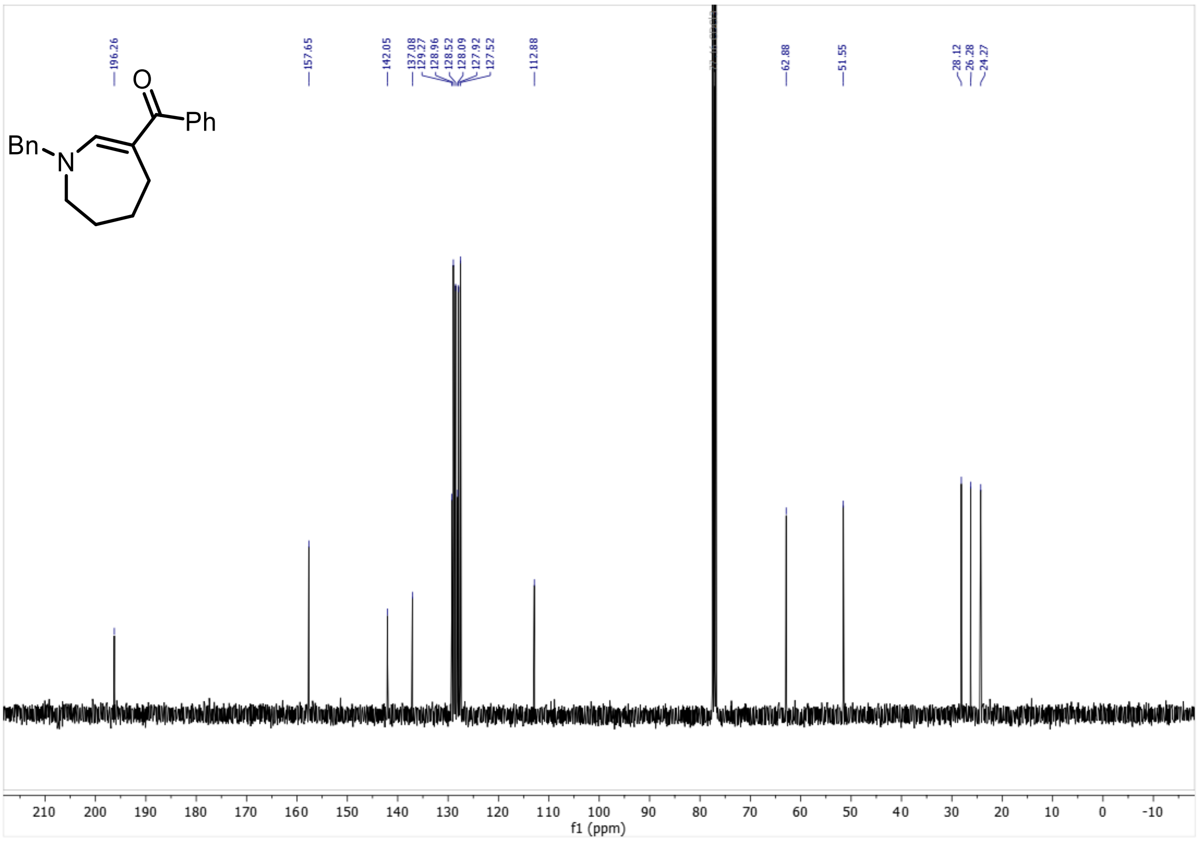

Compound 5b (^{1}H)

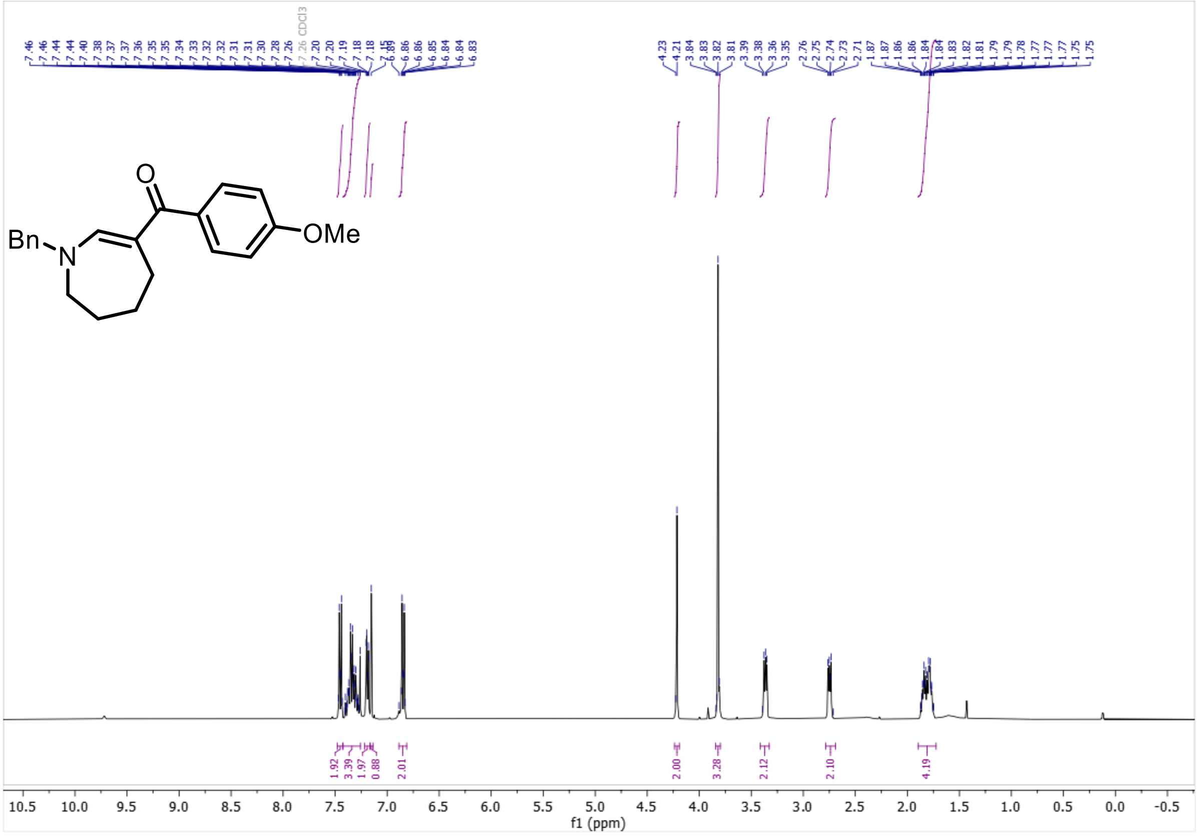

Compound 5b (^{13}C)

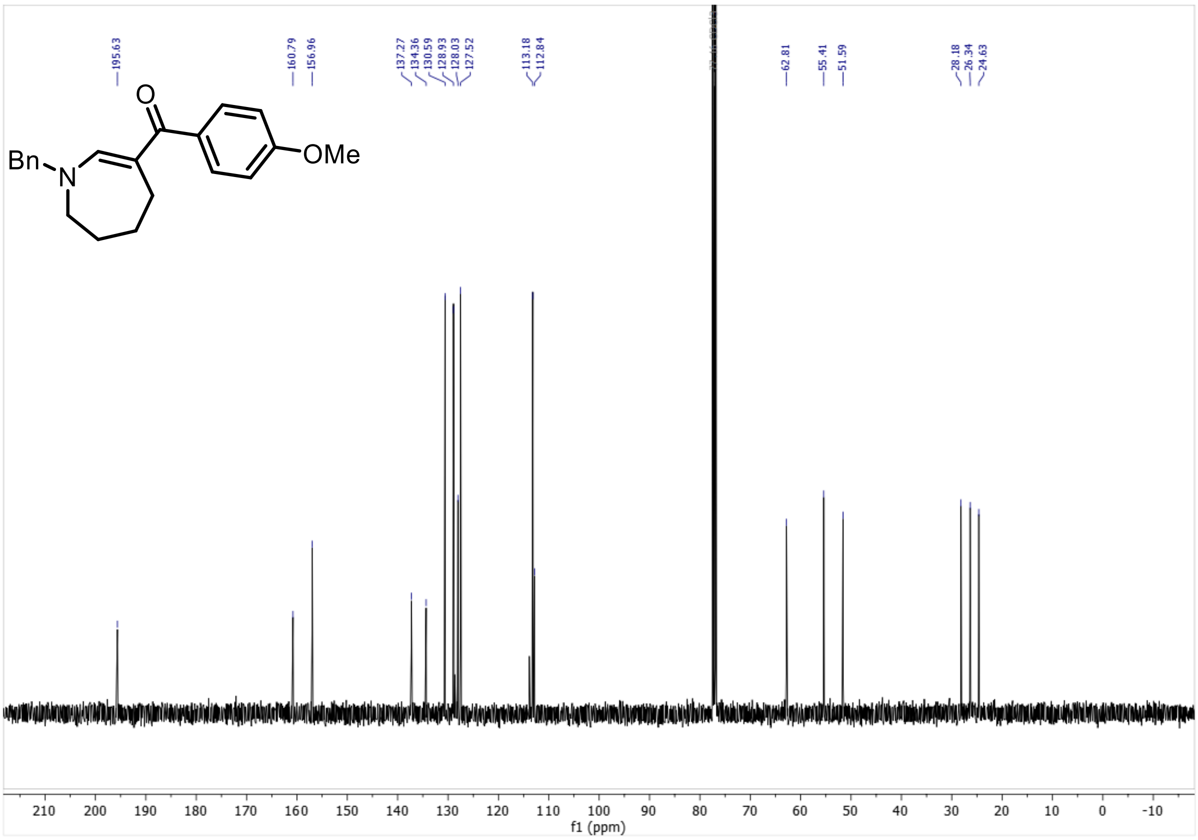

Compound 5c (^1H)

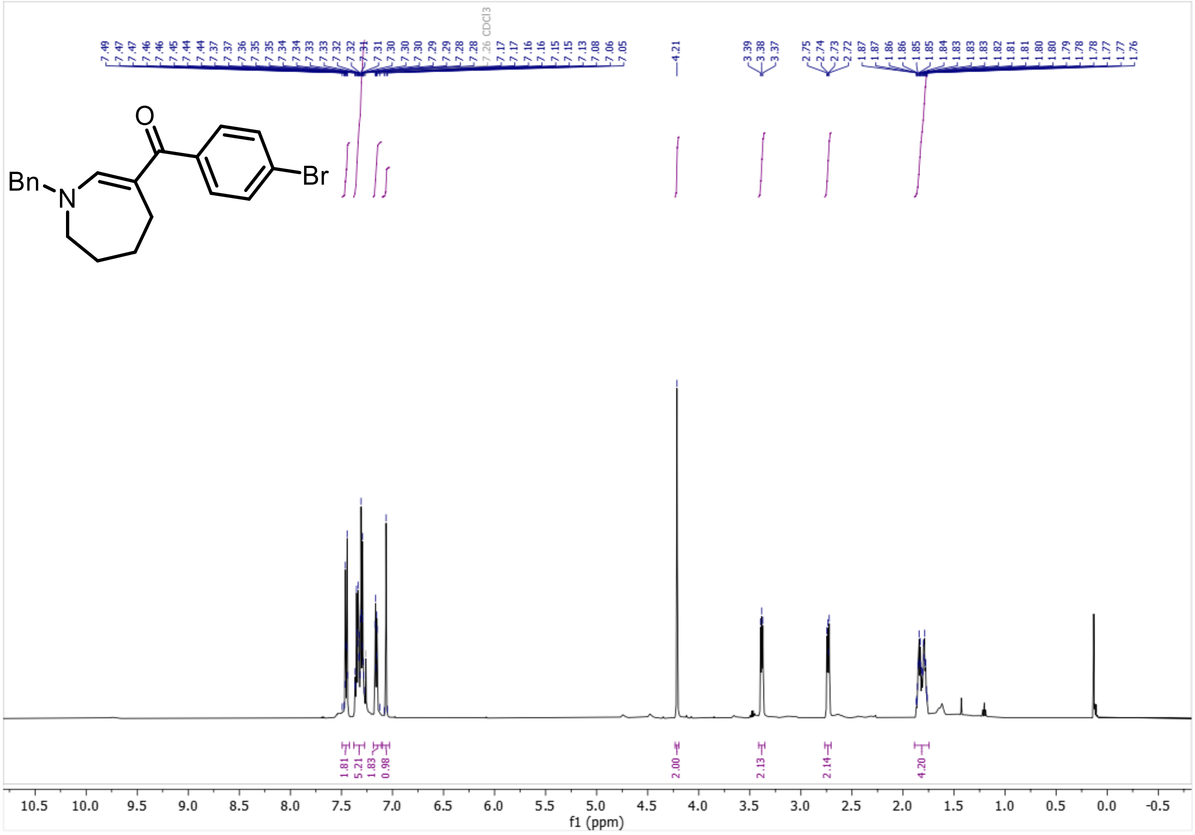

Compound 5c (^{13}C)

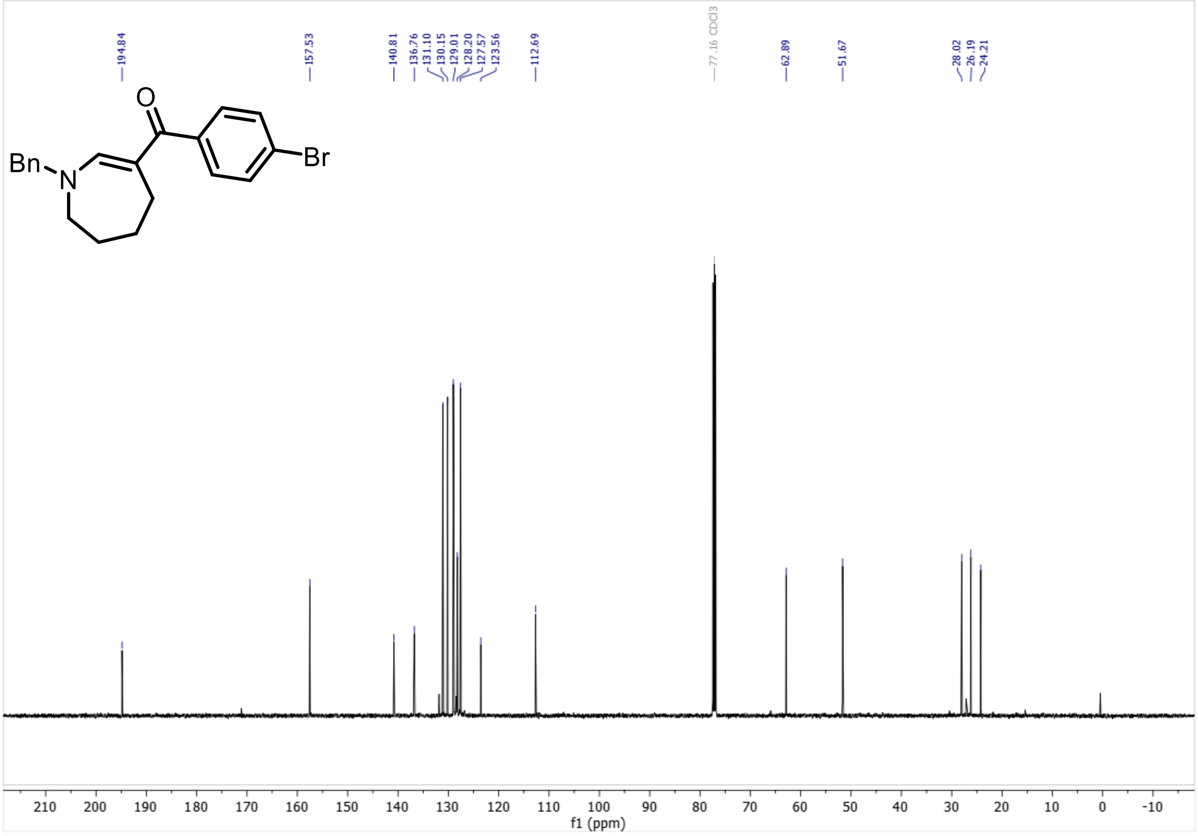

Compound 5d (^{1}H)

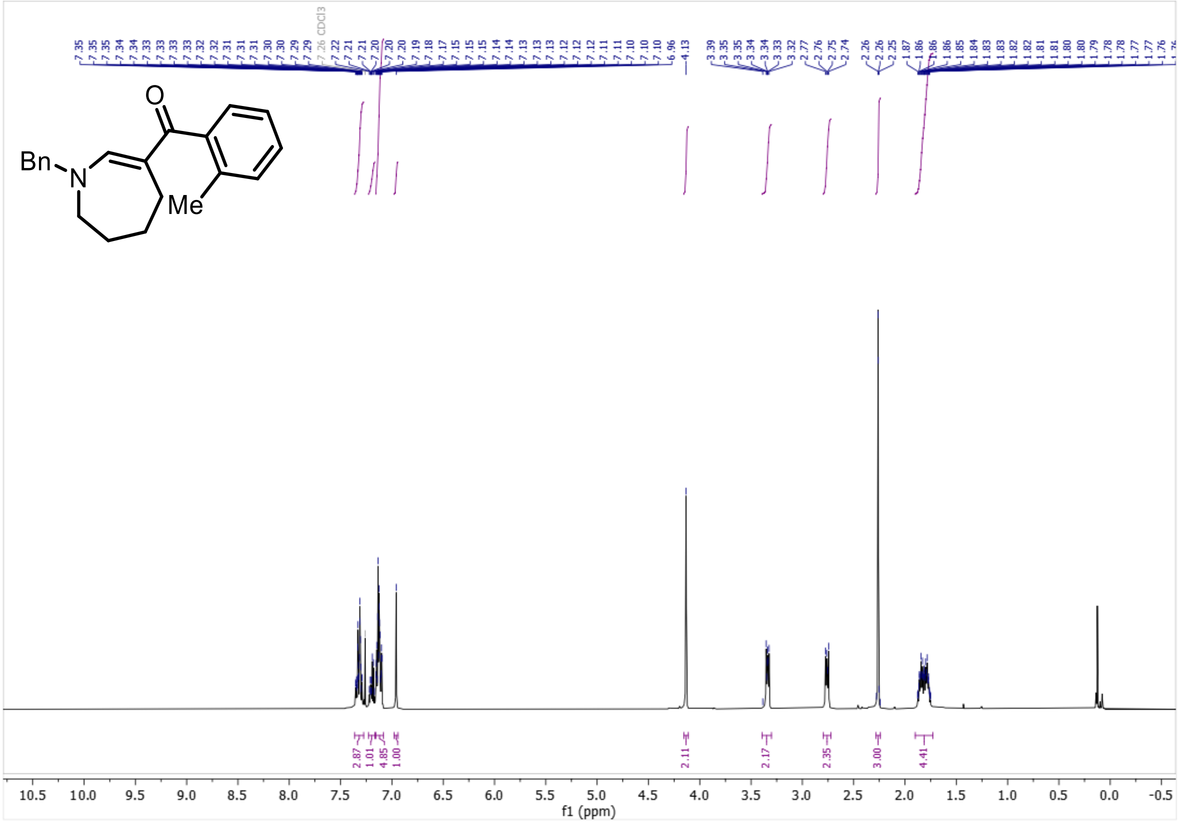

Compound 5d (^{13}C)

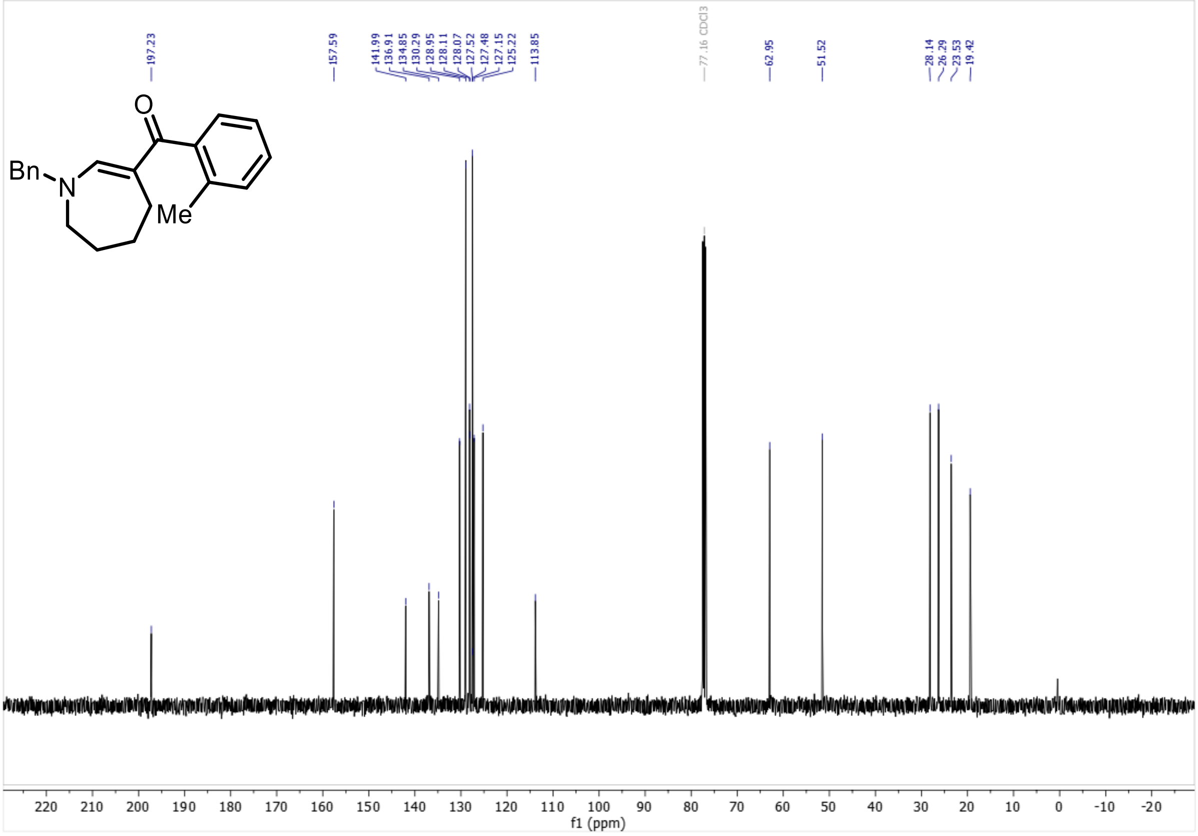

Compound 5e (^{1}H)

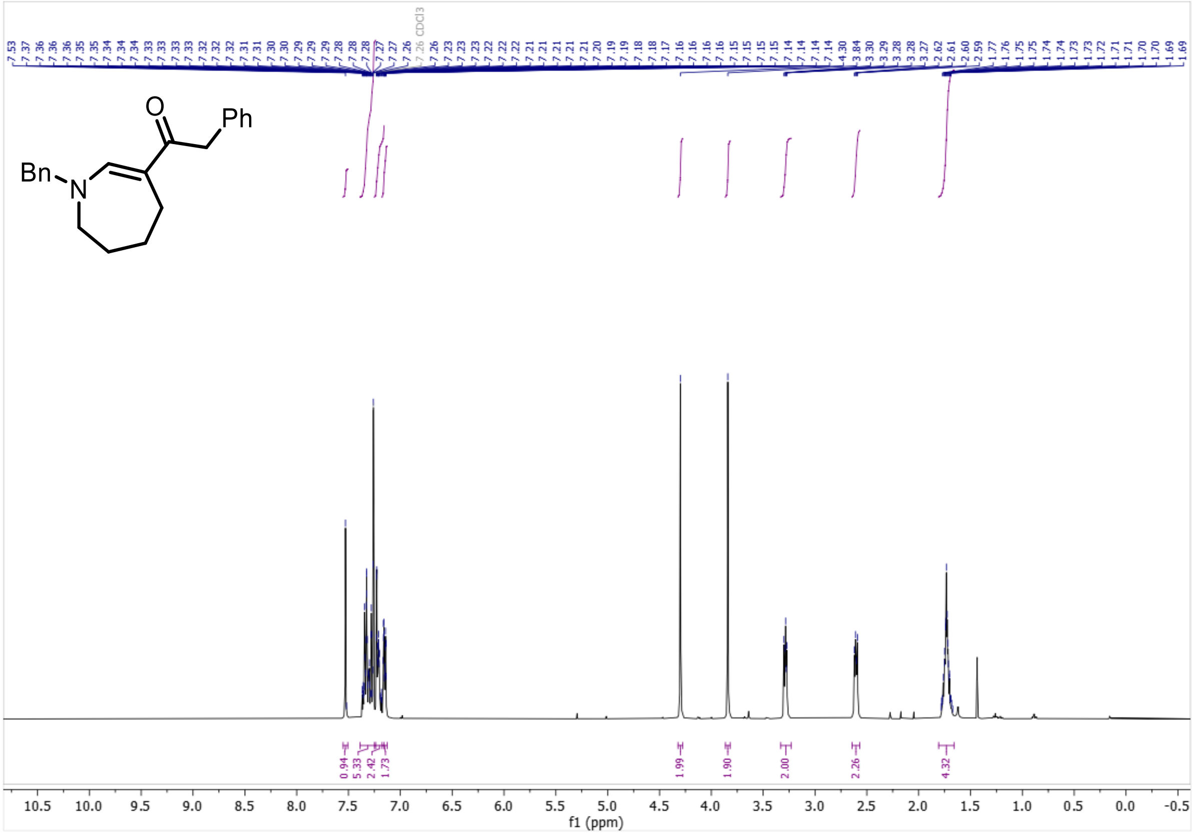

Compound 5e (^{13}C)

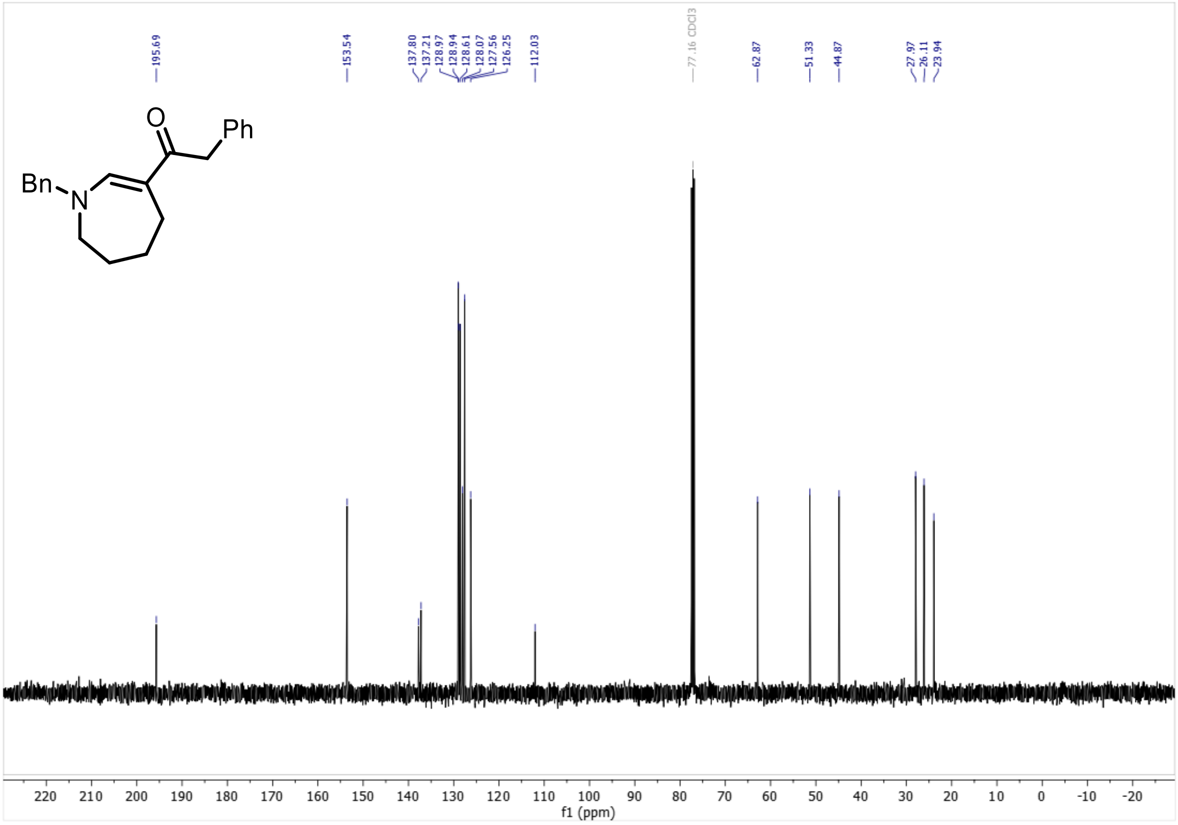

Compound 5f (^{1}H)

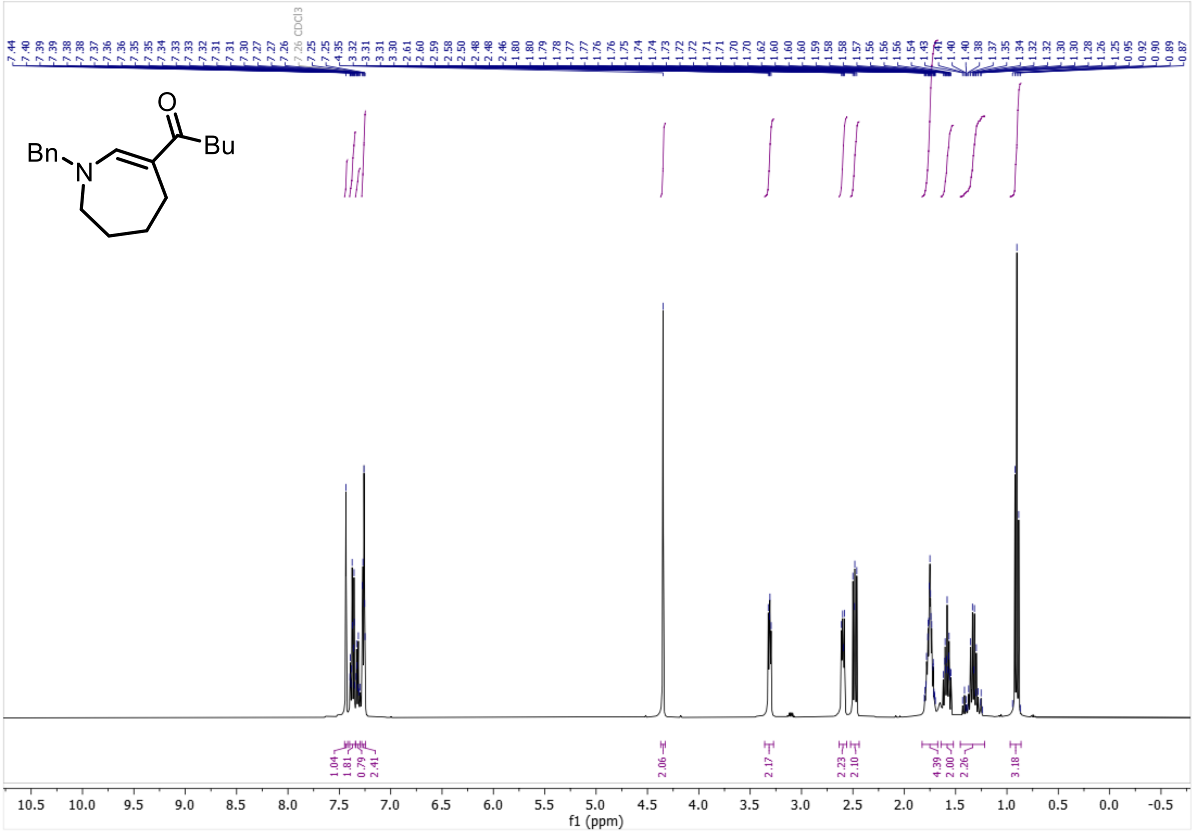

Compound 5f (^{13}C)

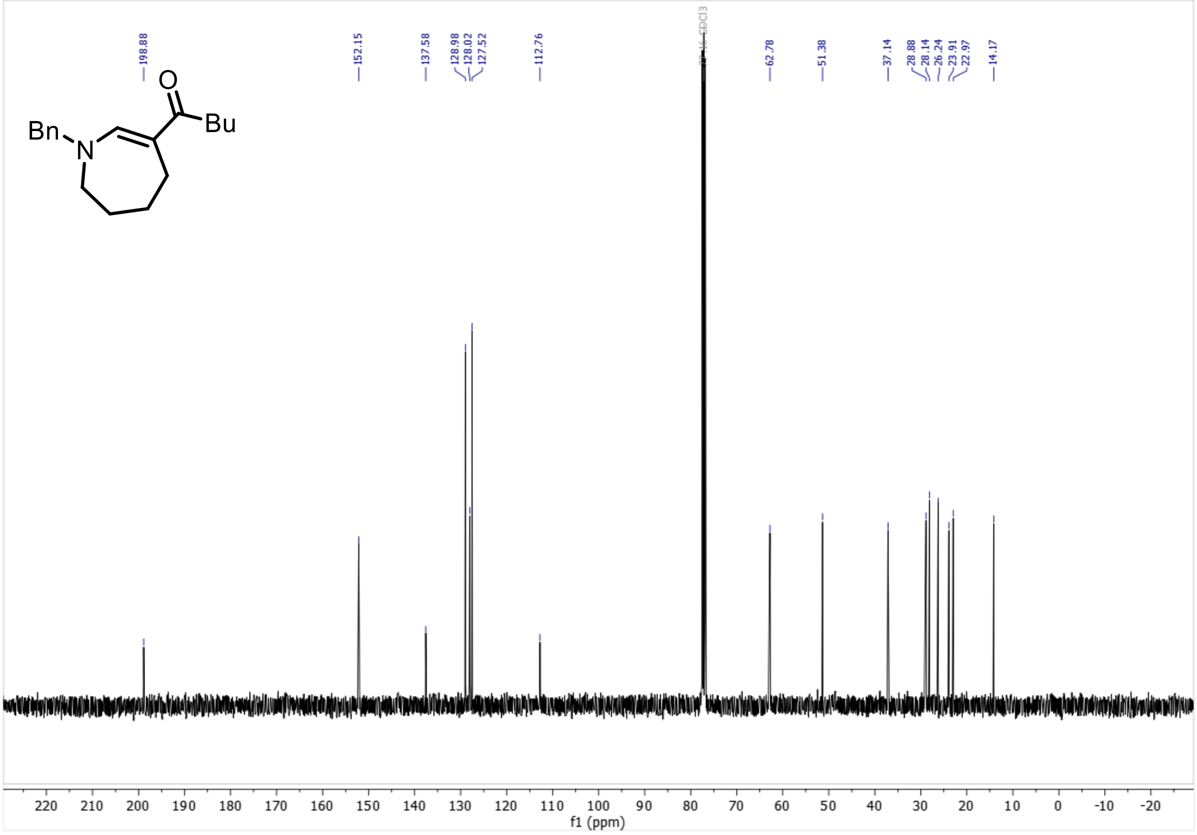

Compound 5g (^{1}H)

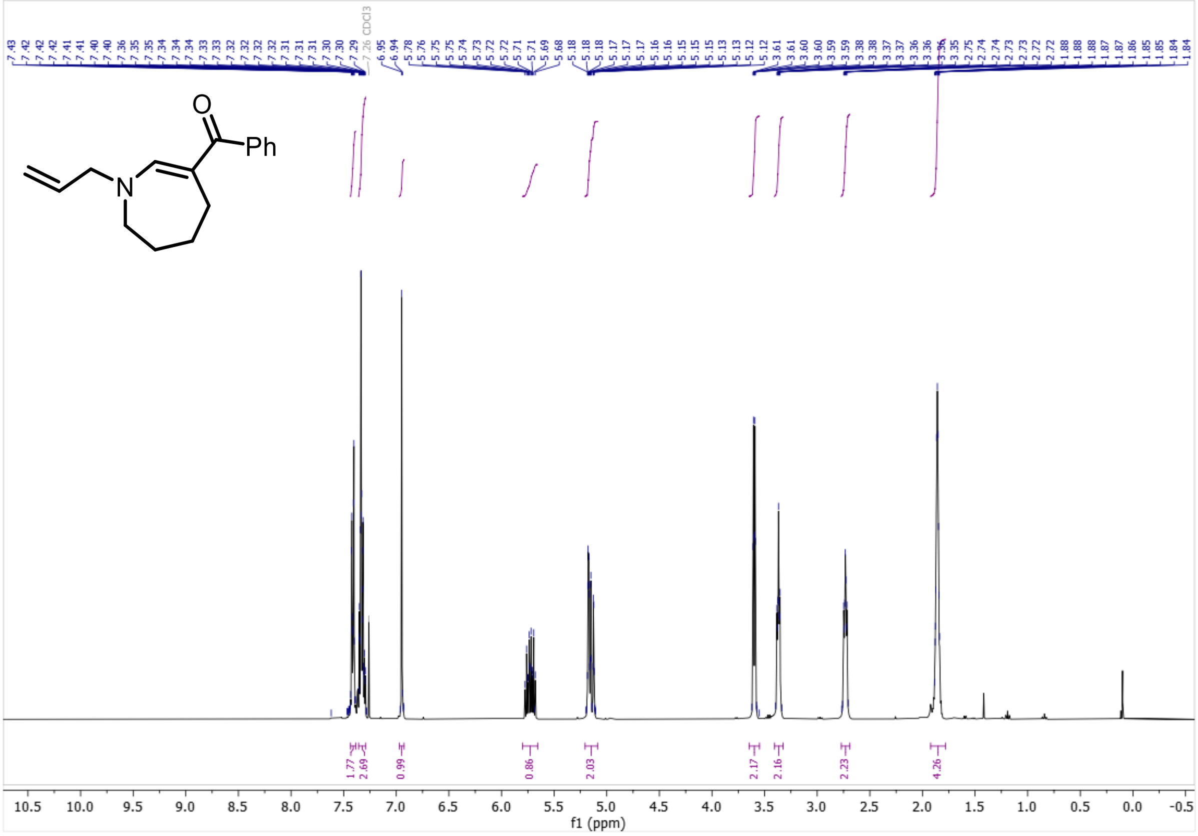

Compound 5g (^{13}C)

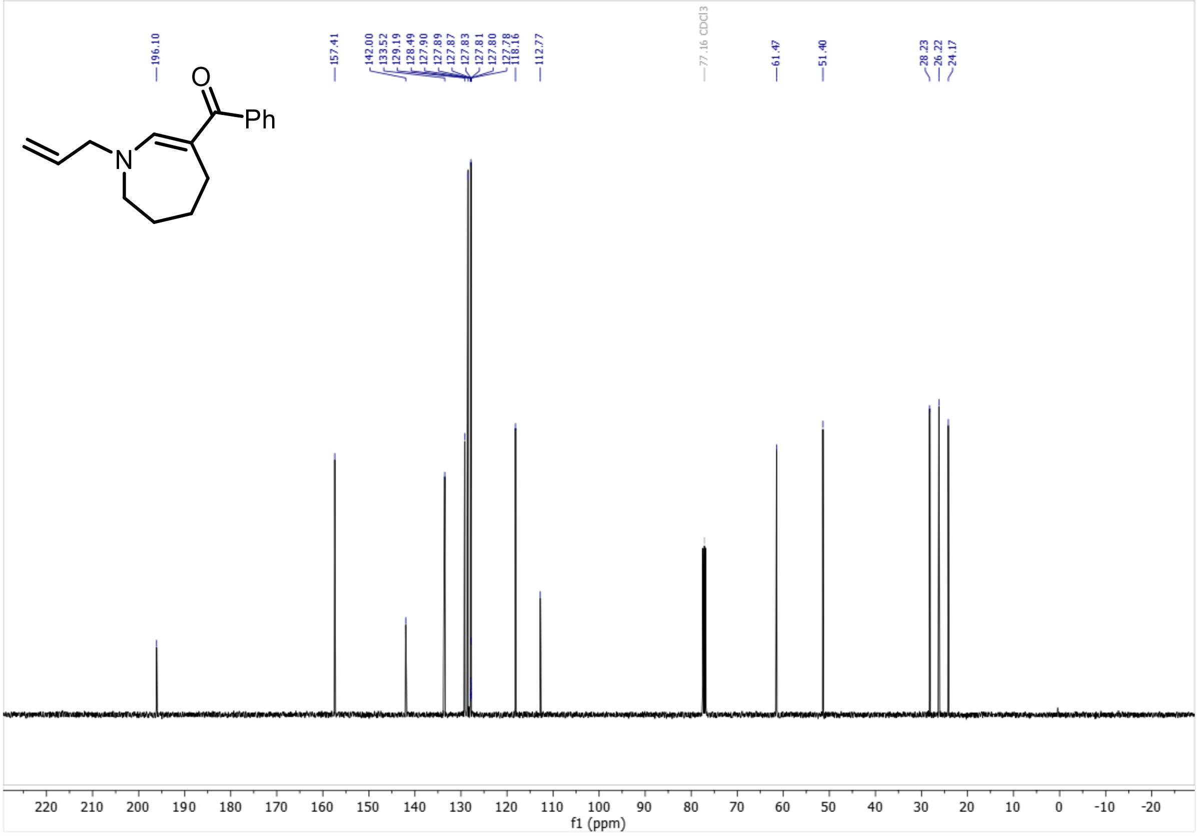

Compound 5h (^{1}H)

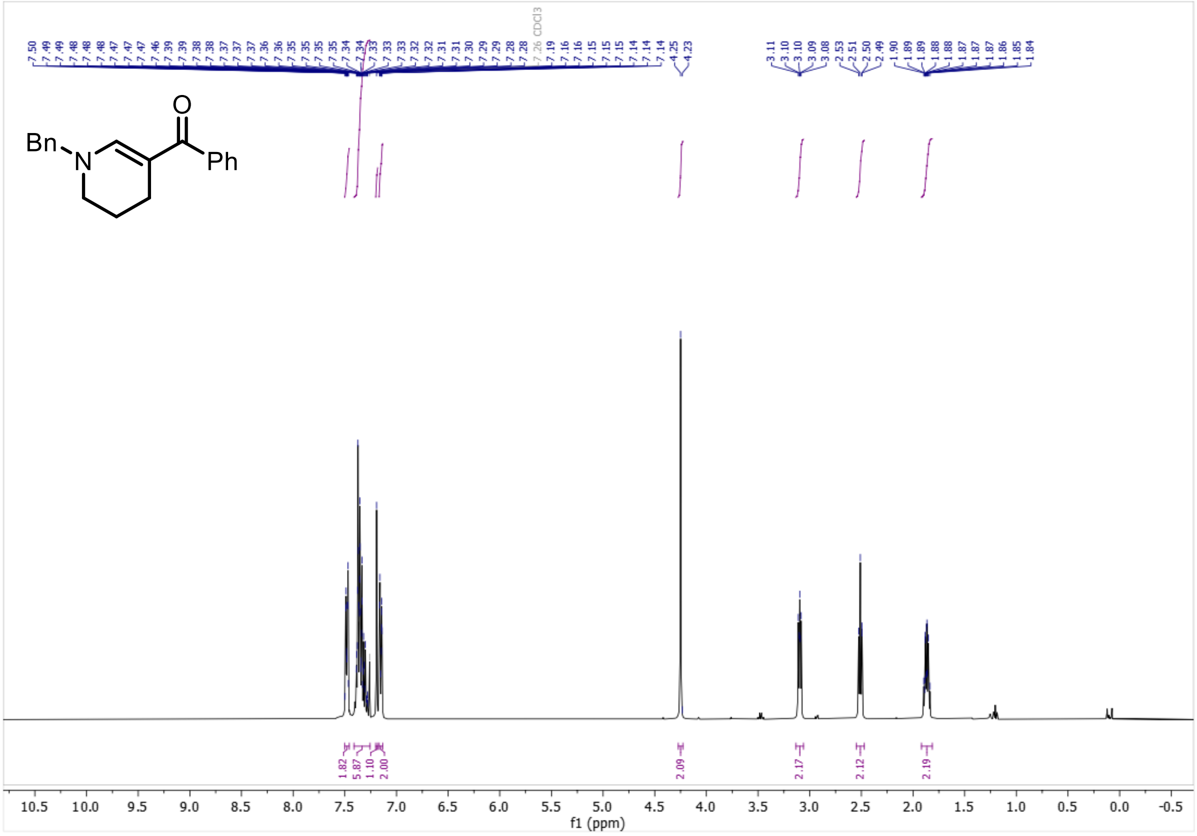

Compound 5h (^{13}C)

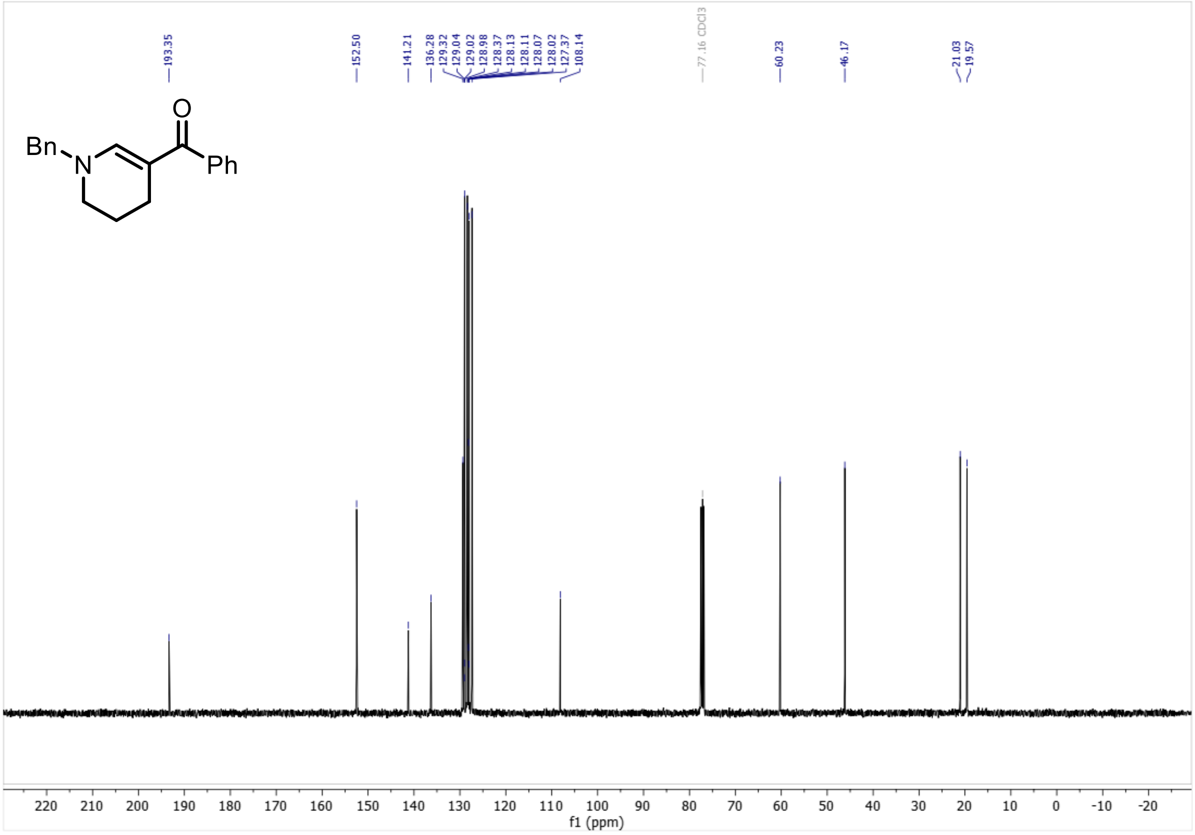

Compound 5i (^{1}H)

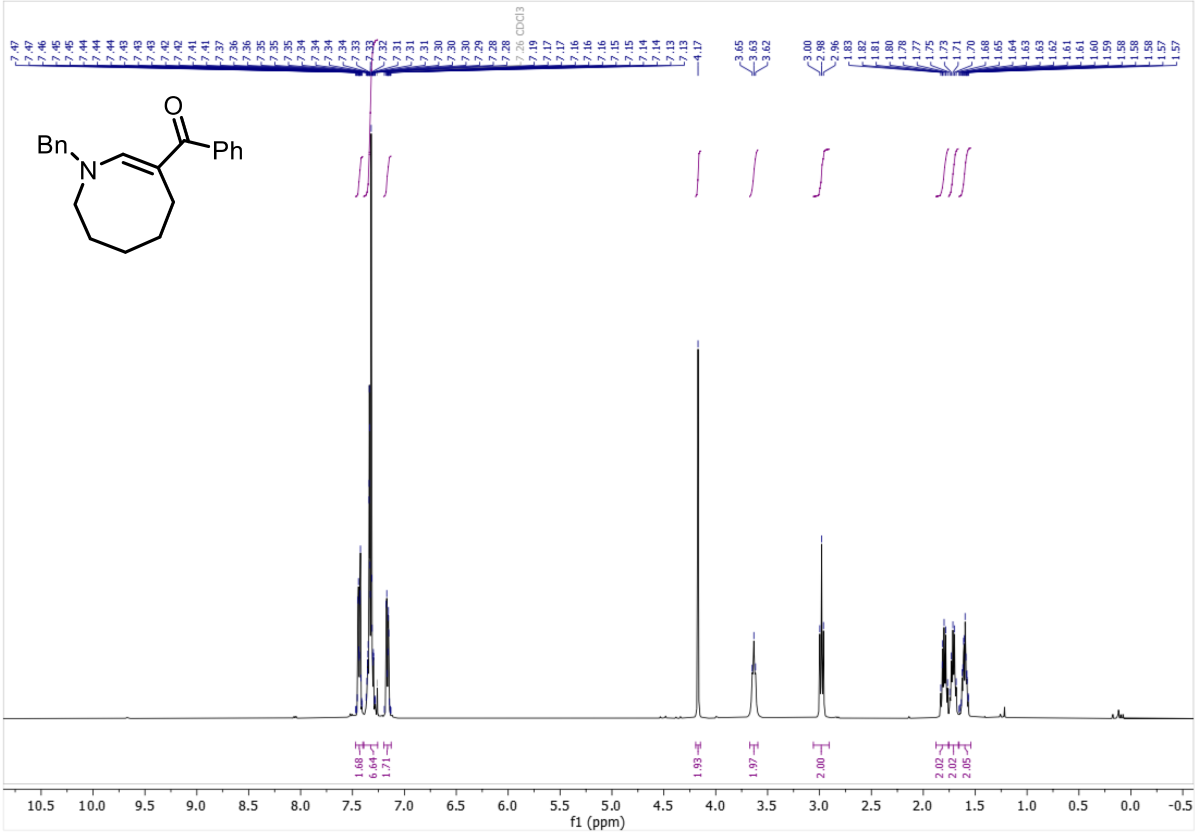

Compound 5i (^{13}C)

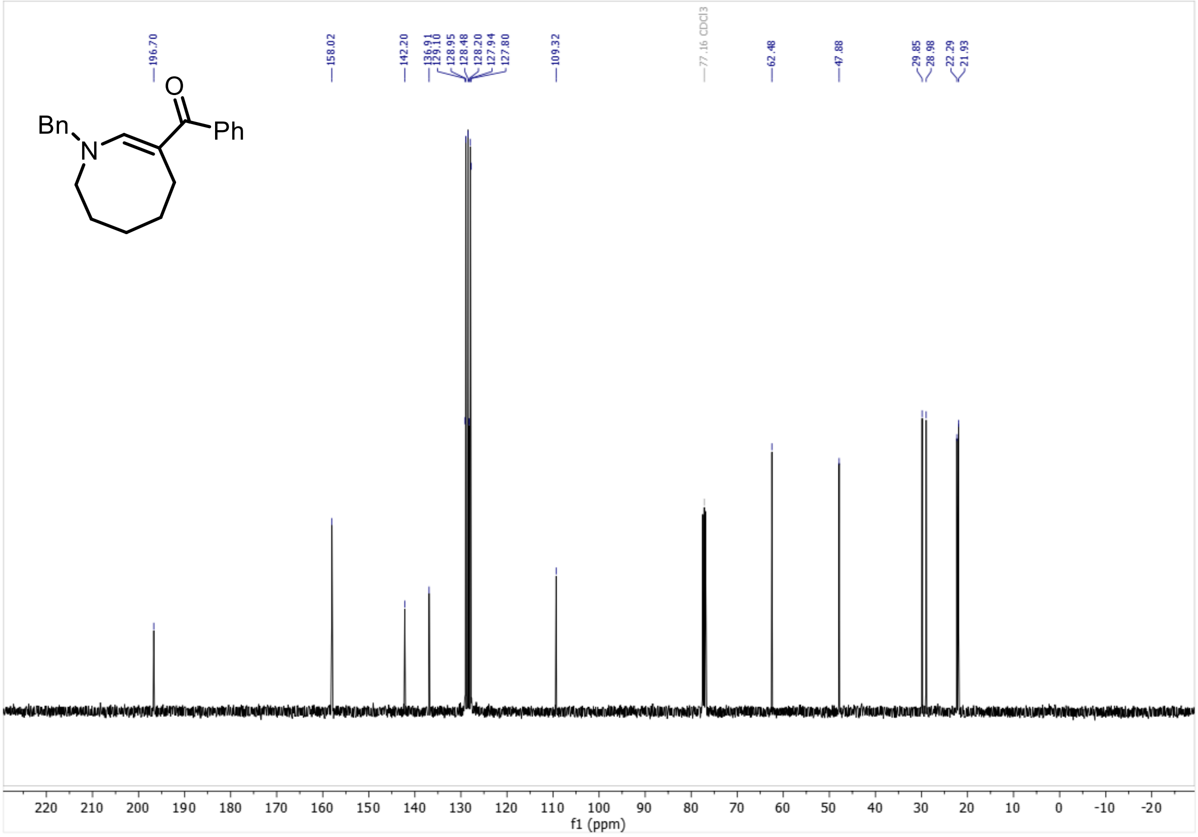

Compound 5j (^{1}H)

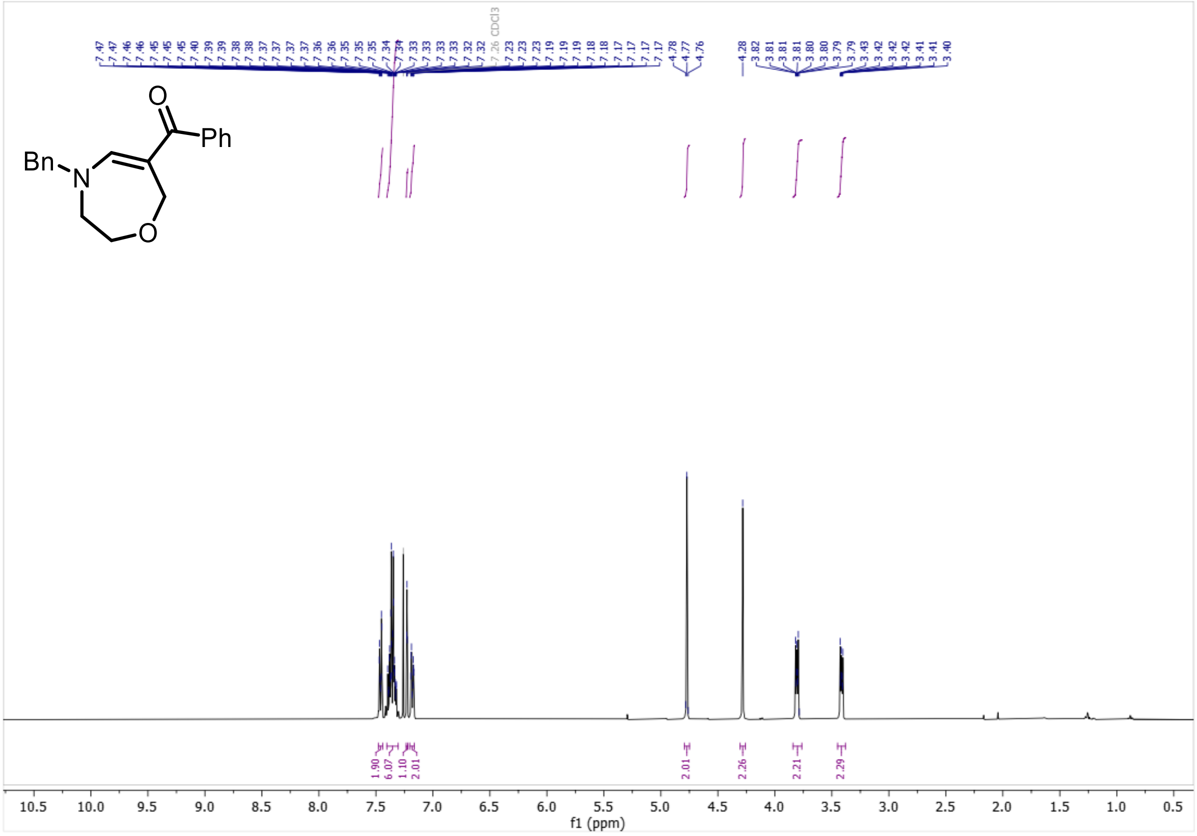

Compound 5j (^{13}C)

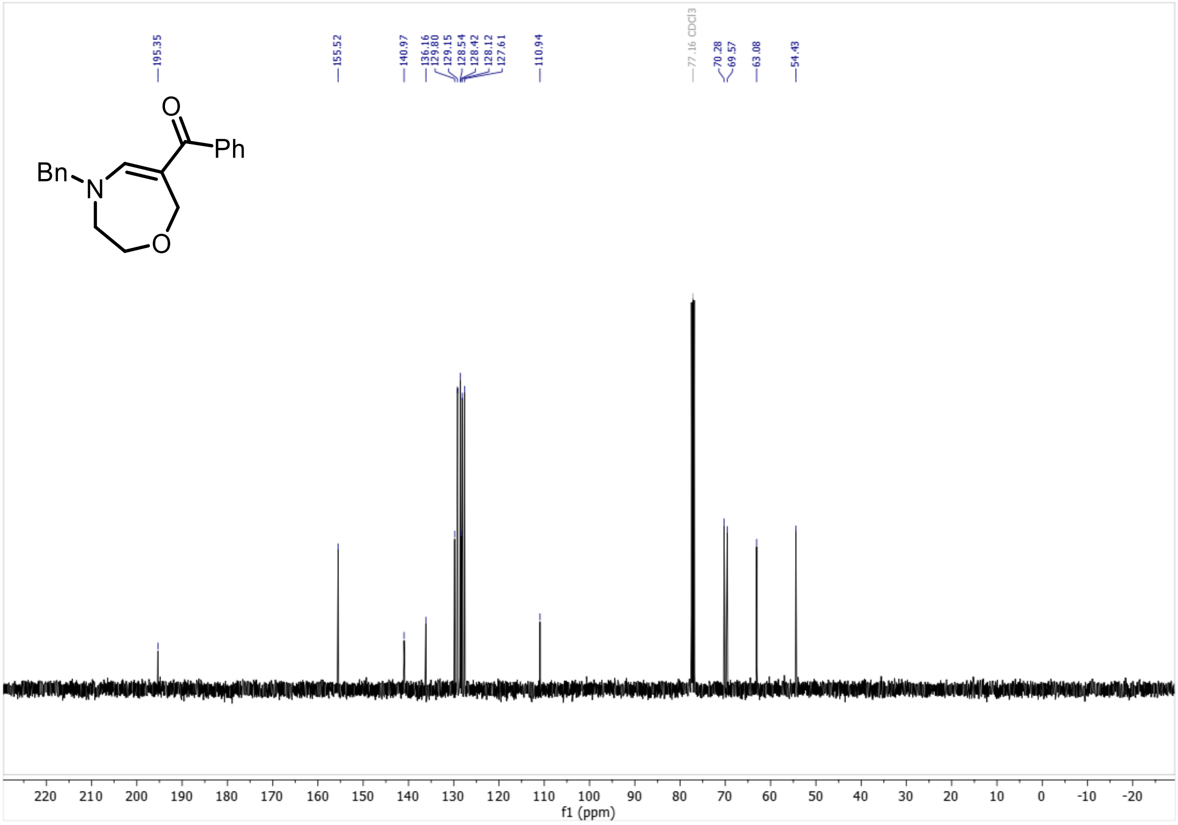

Compound 5k (^{1}H)

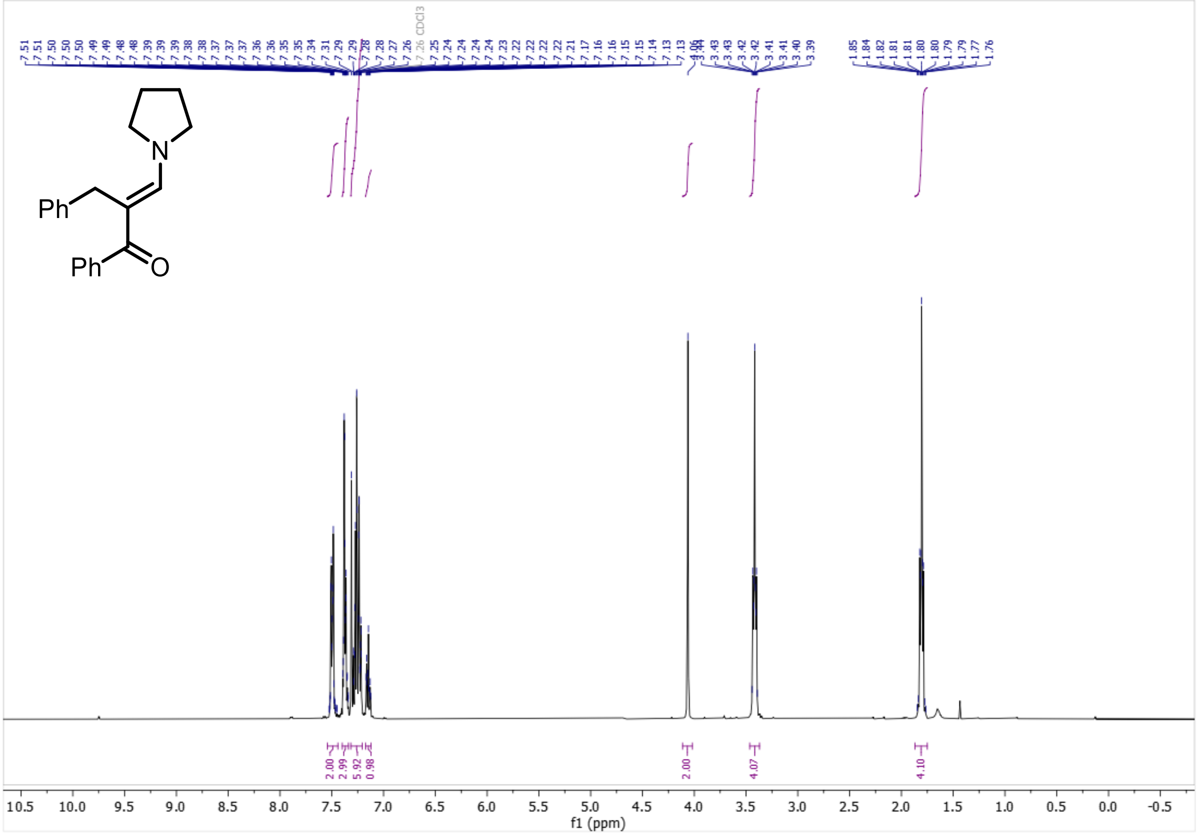

Compound 5k (^{13}C)

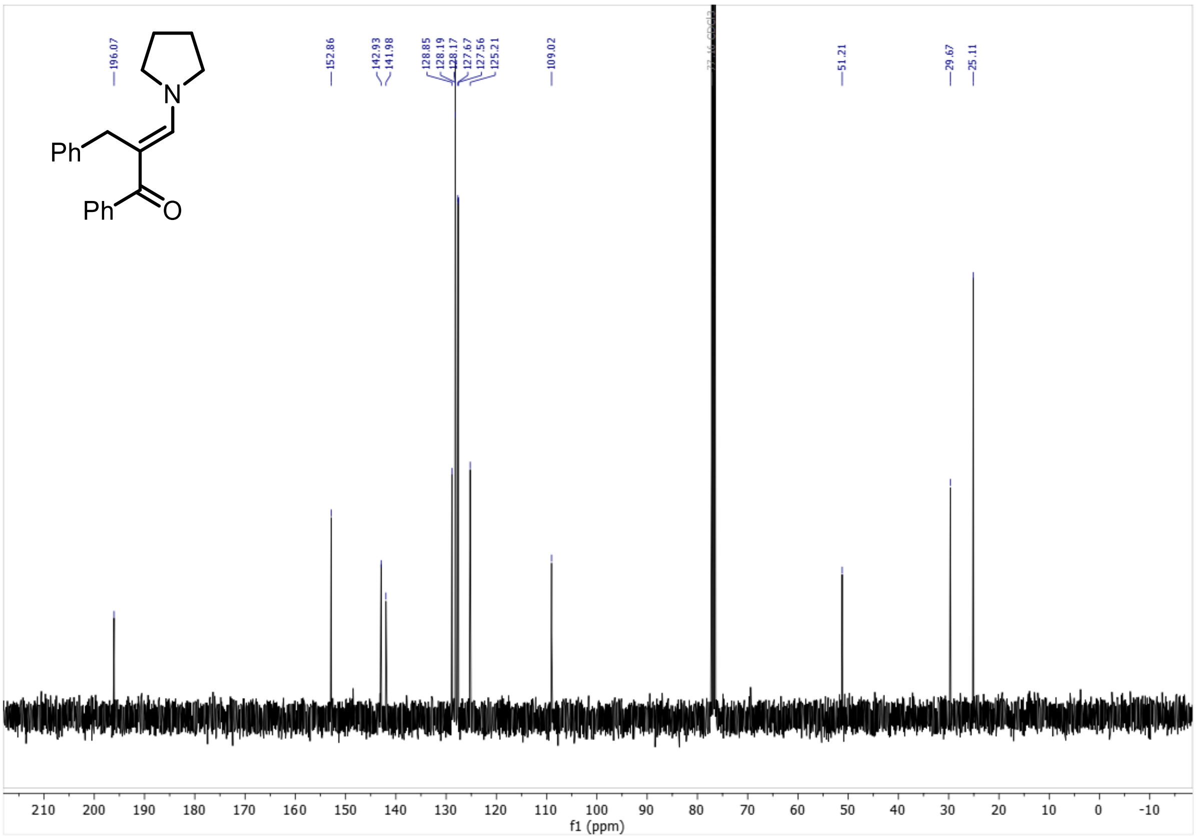

Compound 5l (^{1}H)

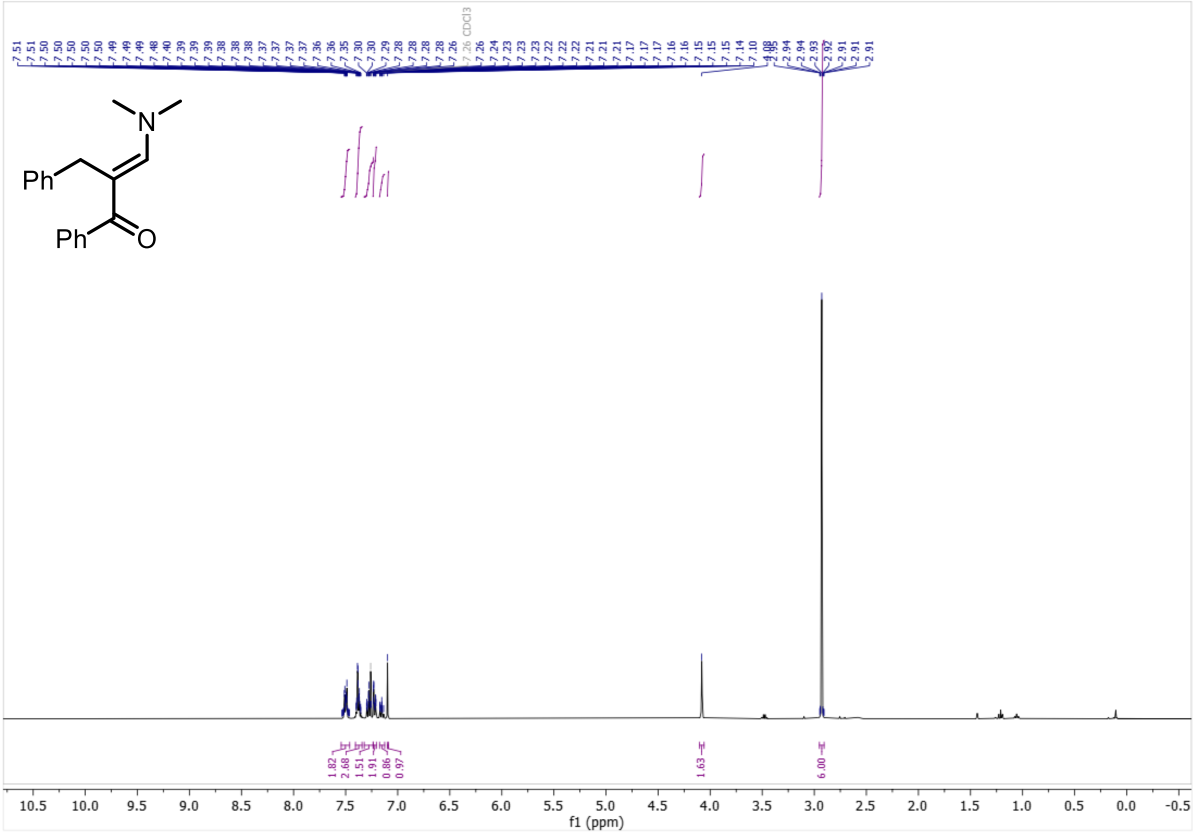

Compound 5l (^{13}C)

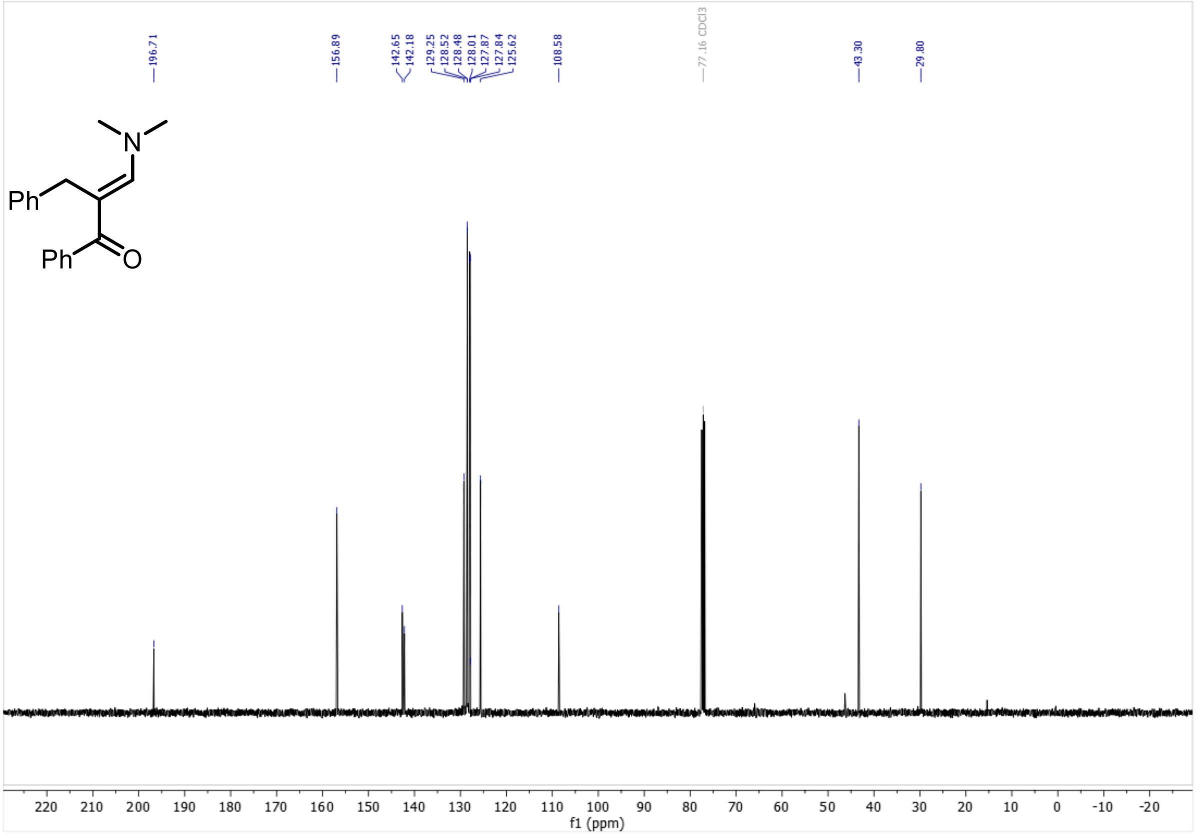

Compound 5m (^{1}H)

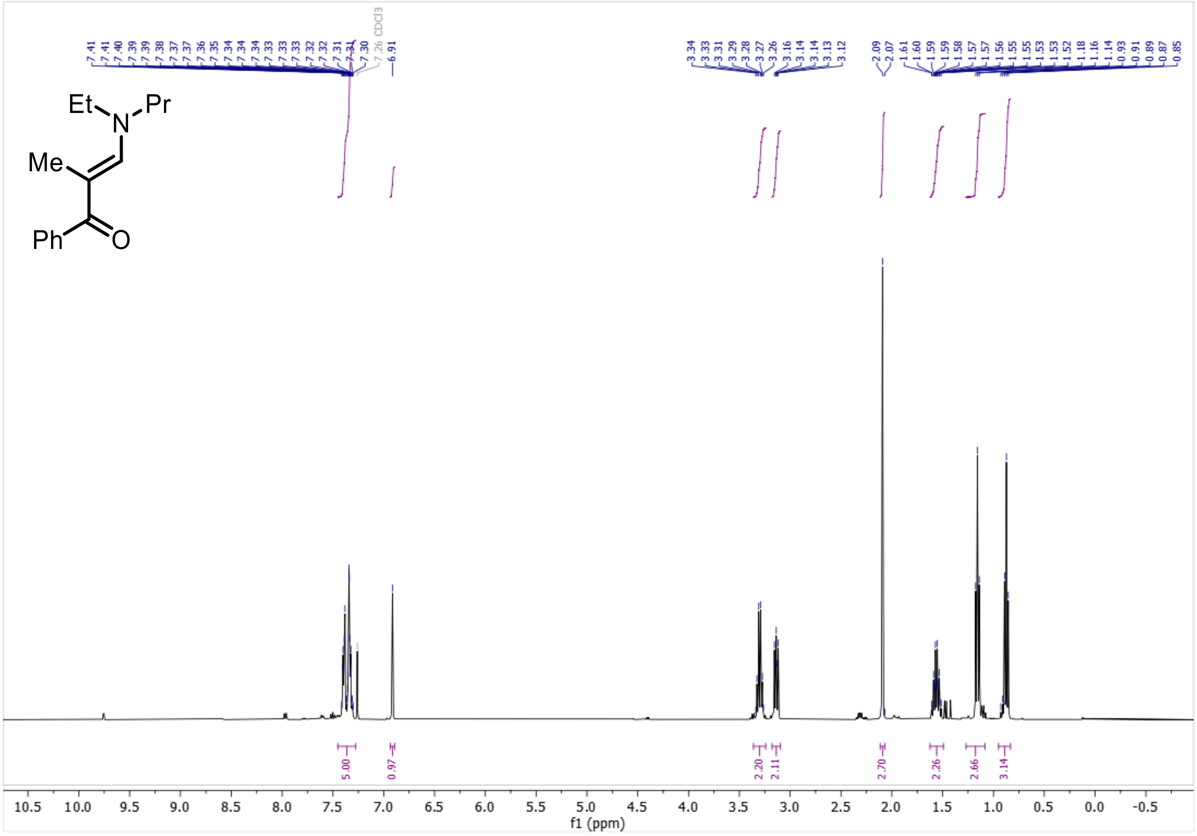

Compound 5m (^{13}C)

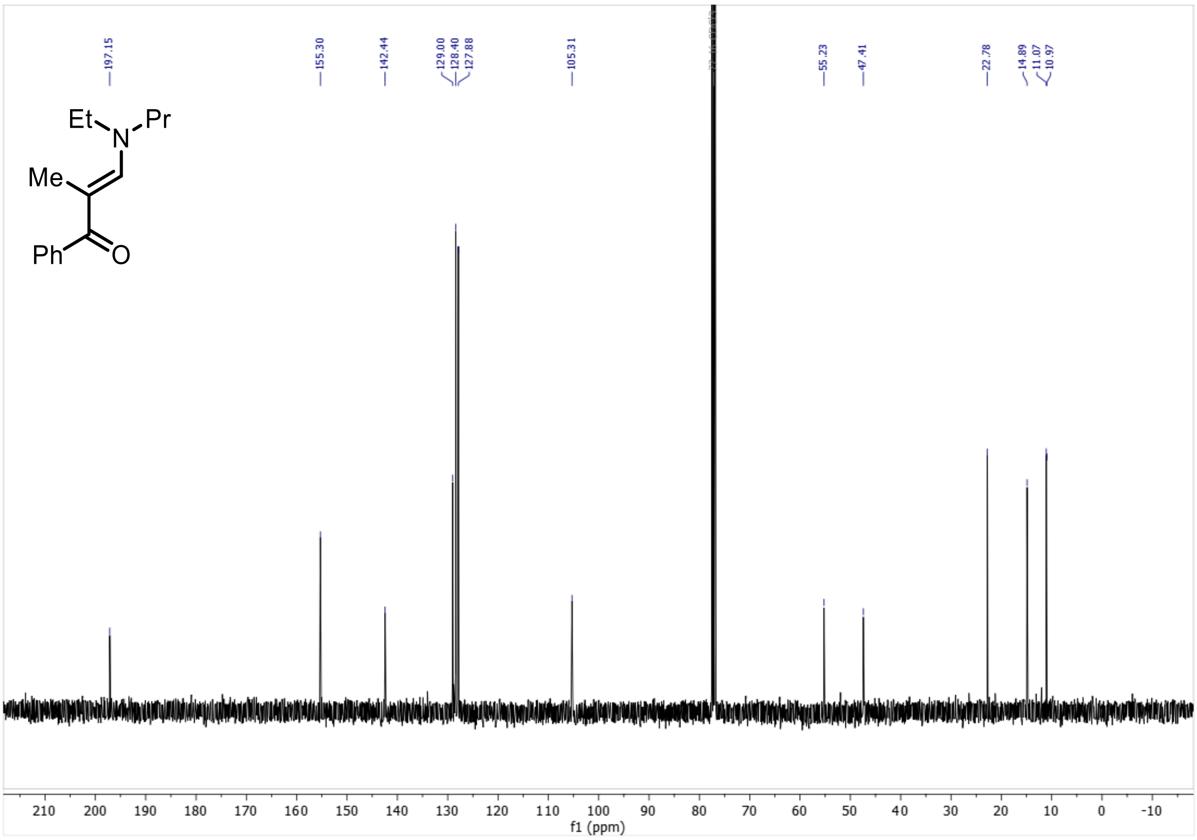

Compound 5n (^{1}H)

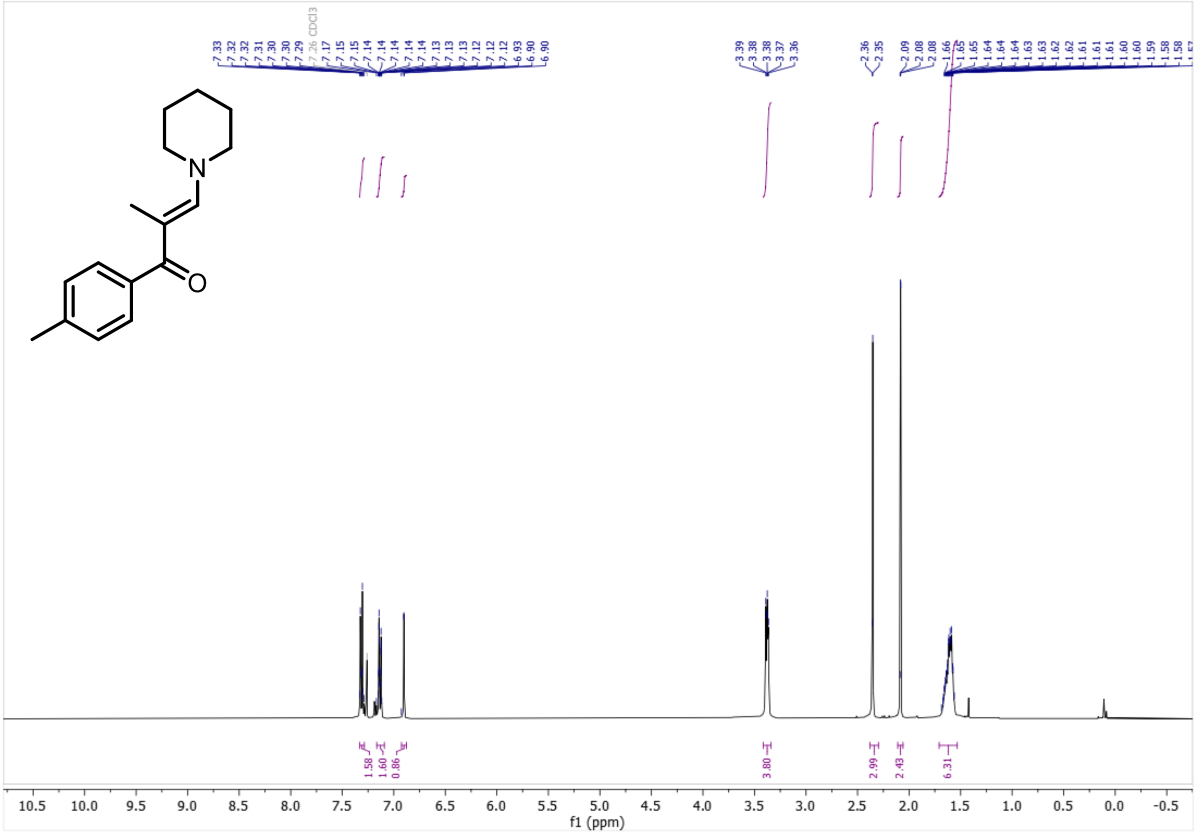

Compound 5n (^{13}C)

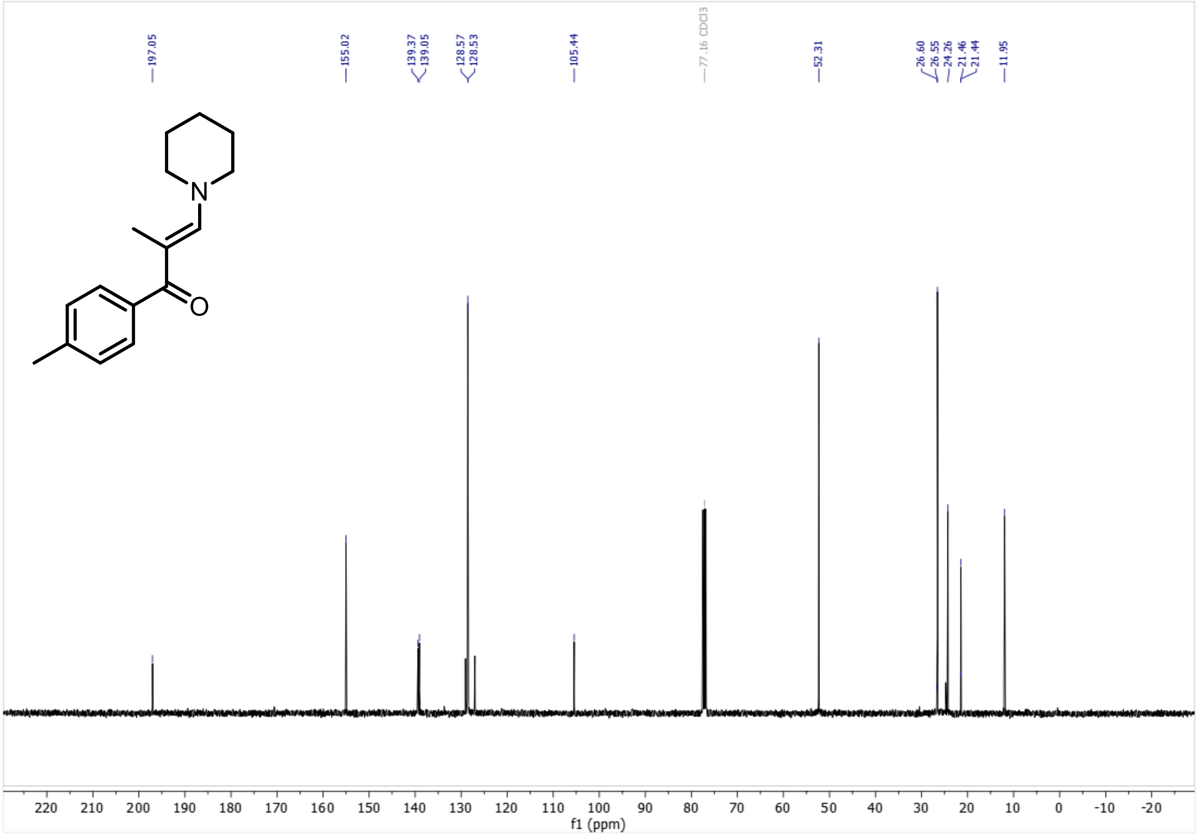

Tolperisone (^{1}H)

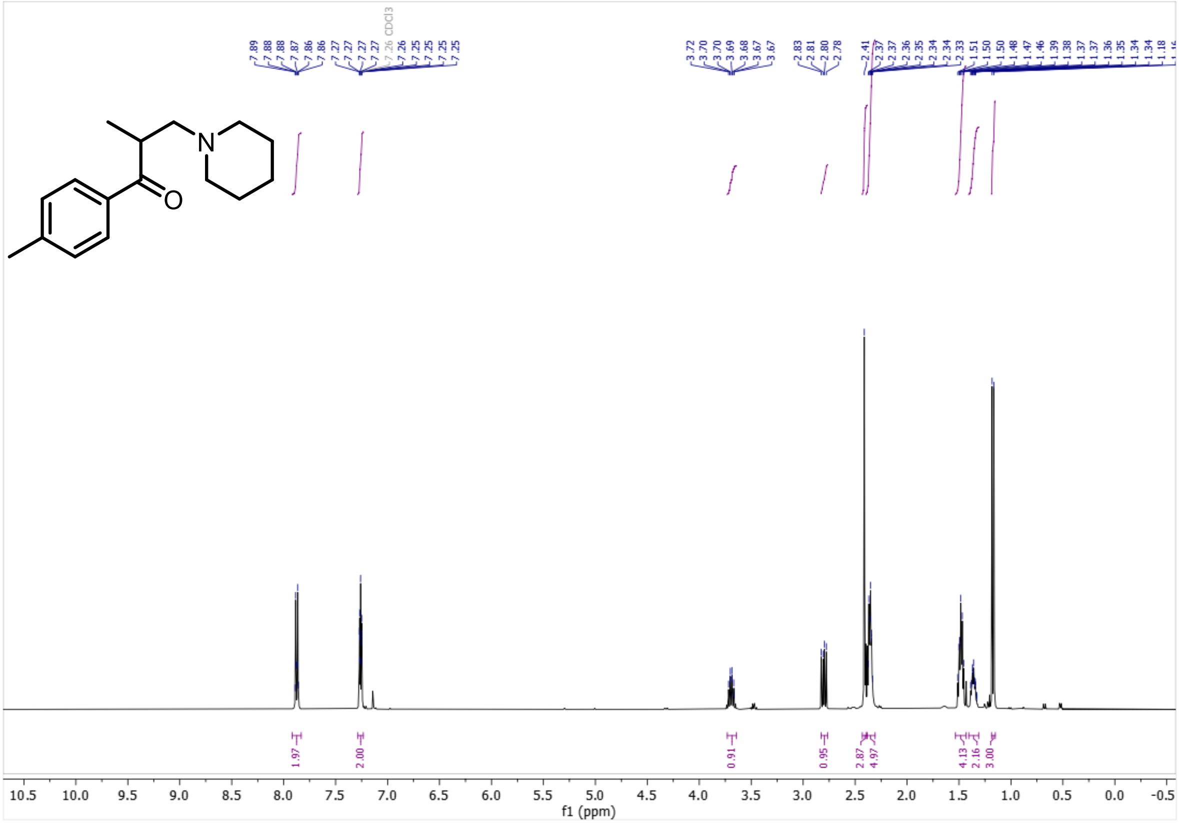

Tolperisone (^{13}C)

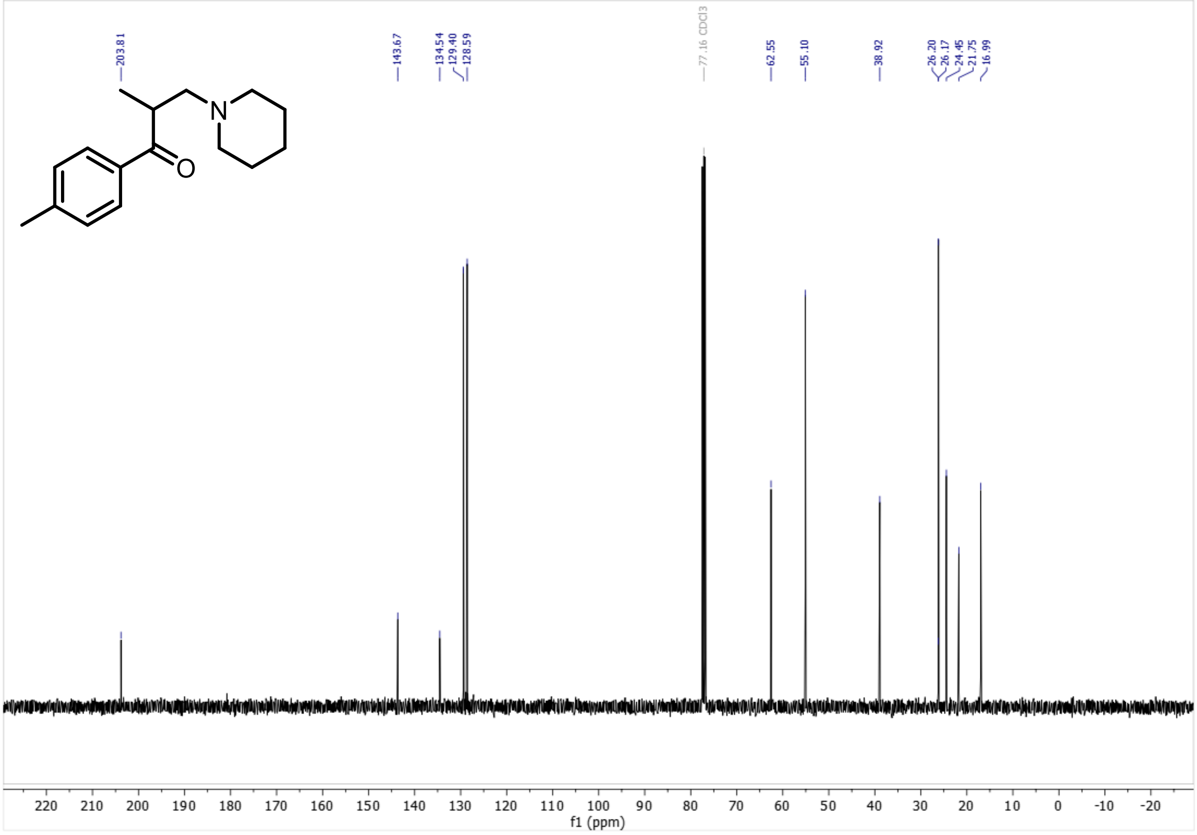

Compound 5o (^{1}H)

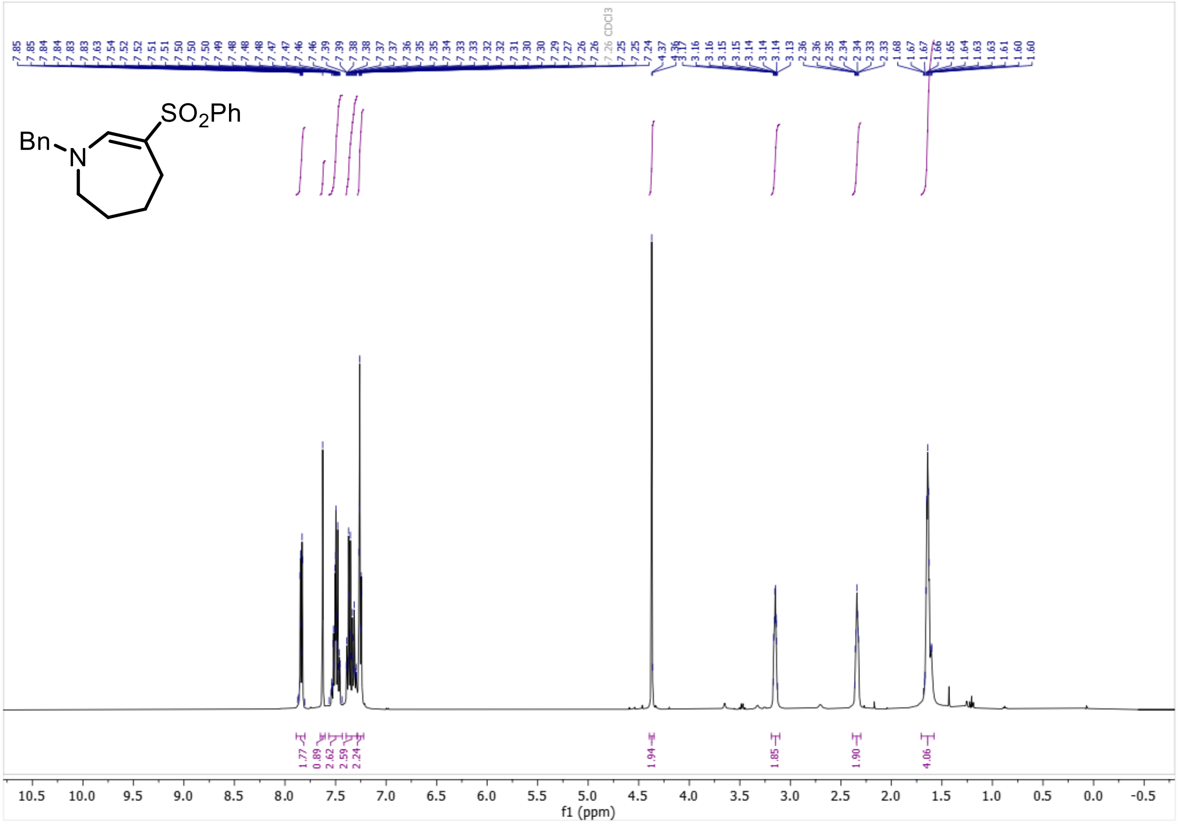

Compound 5o (^{13}C)

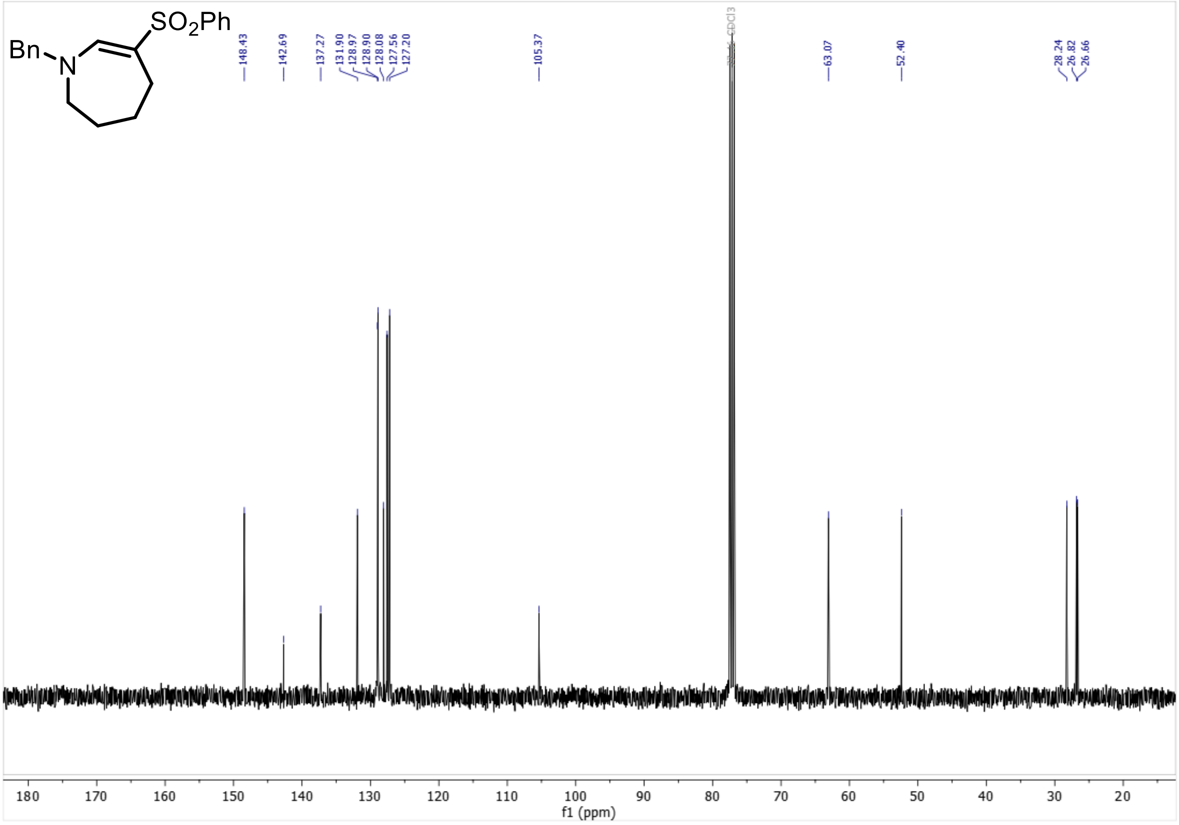

Compound 5p (^{1}H)

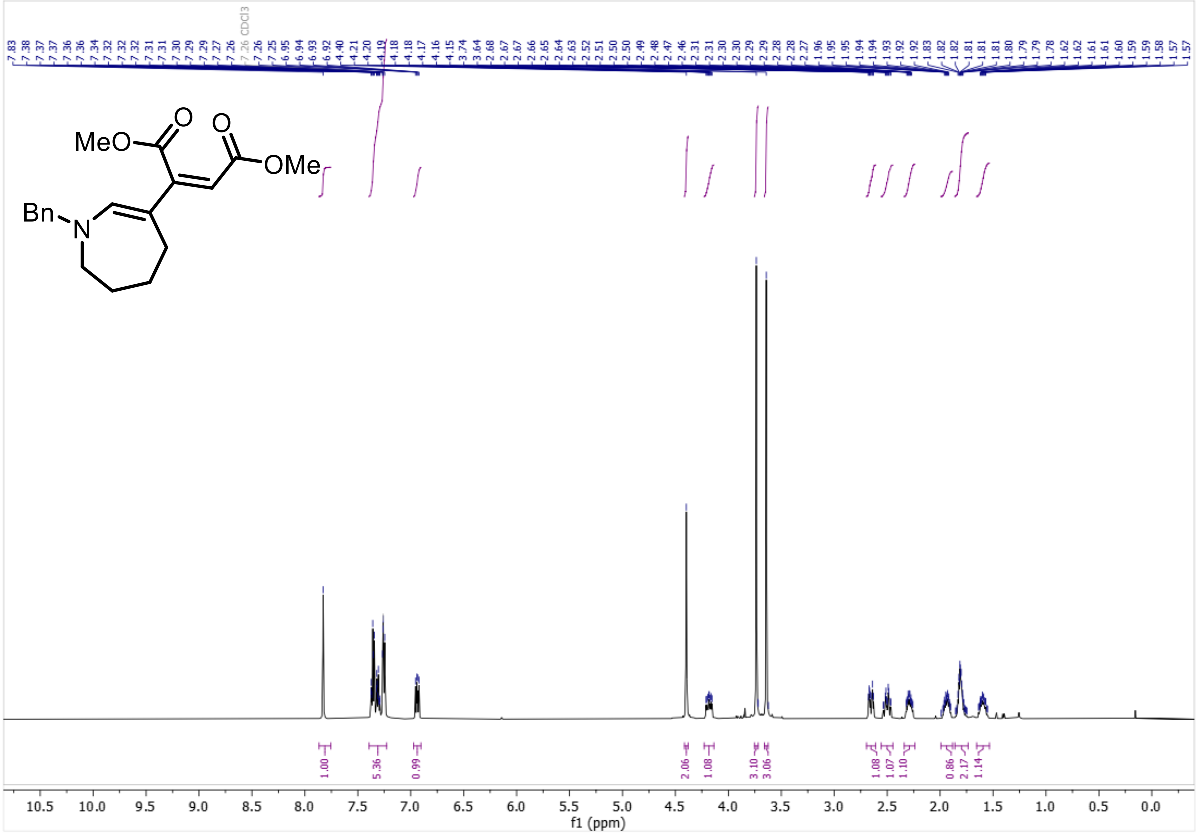

Compound 5p (^{13}C)

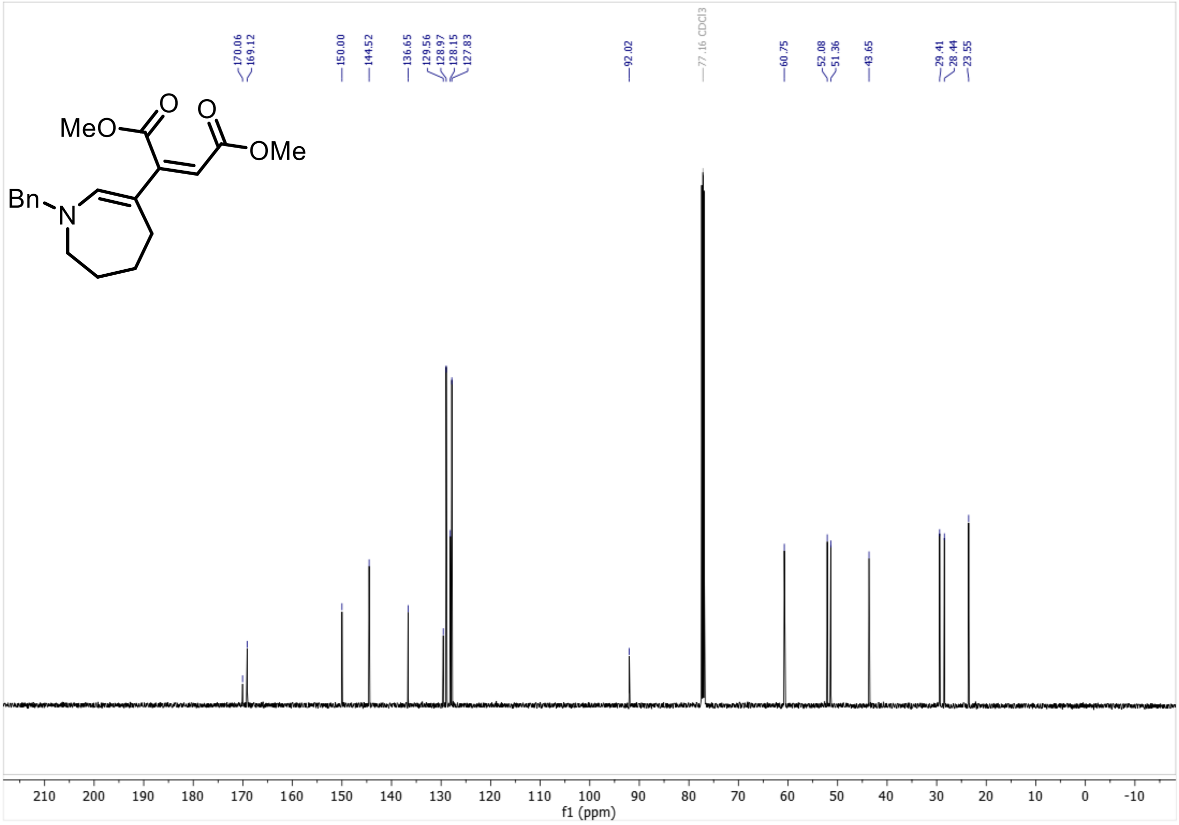

Compound 5q (^1H)

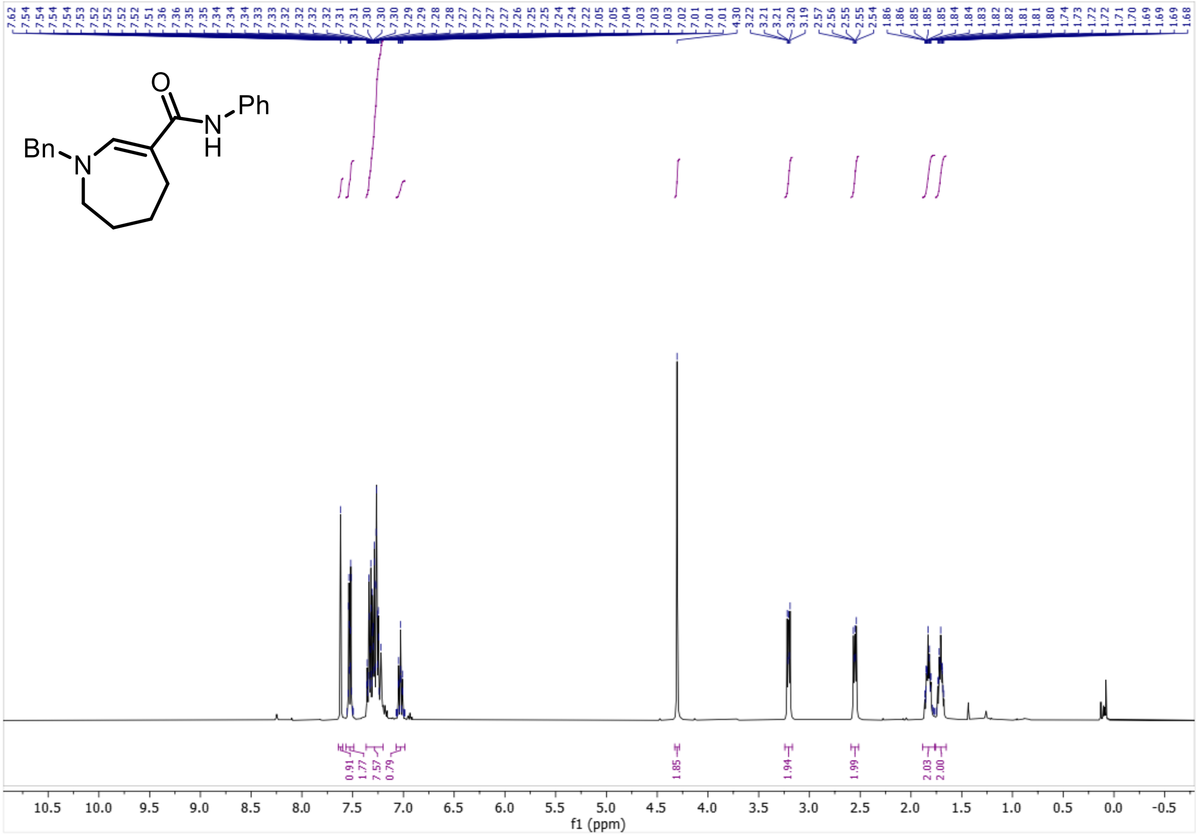

Compound 5q (^{13}C)

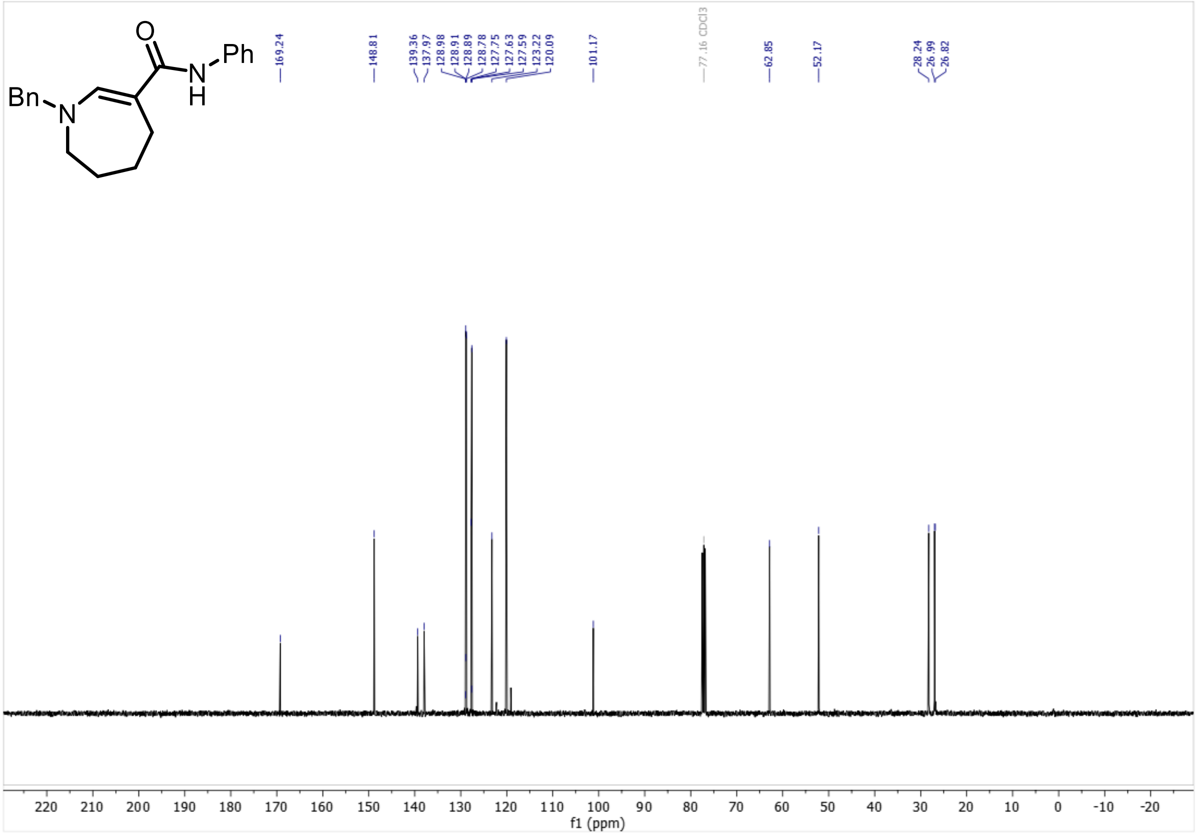

Compound 5r (^{1}H)

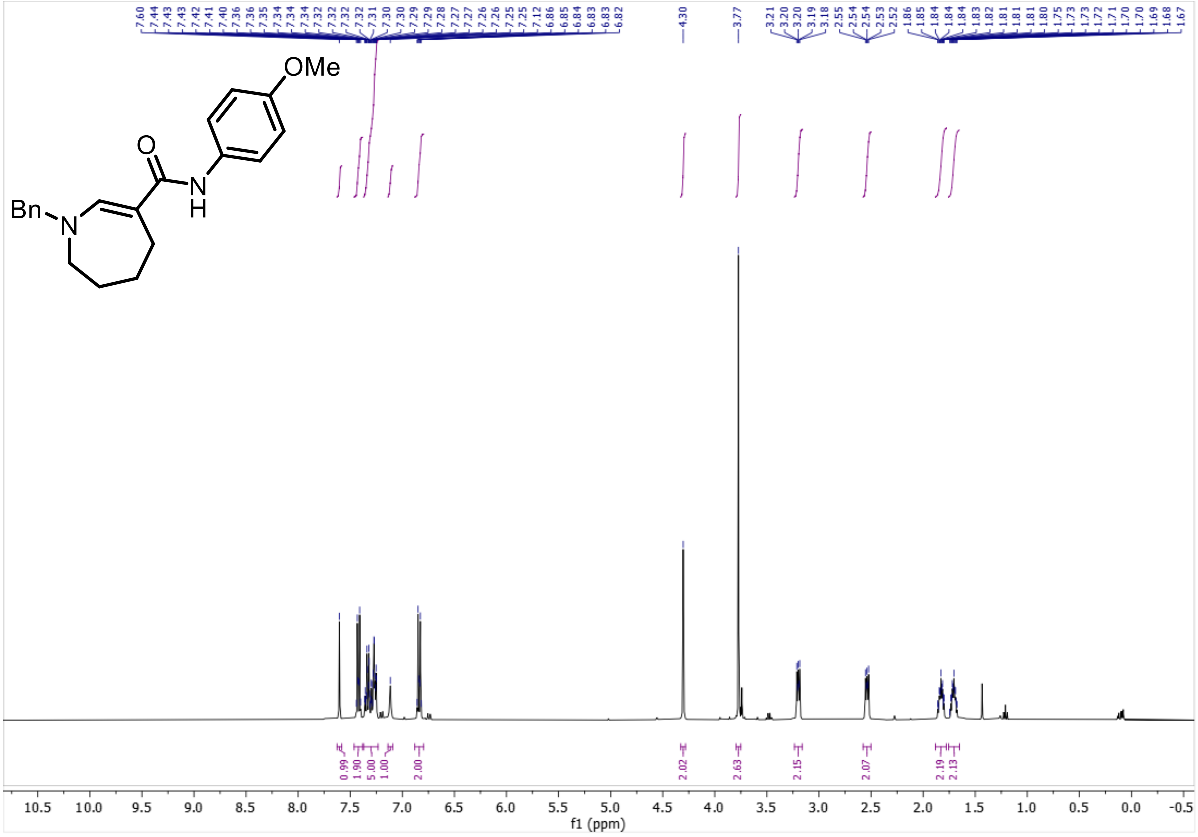

Compound 5r (^{13}C)

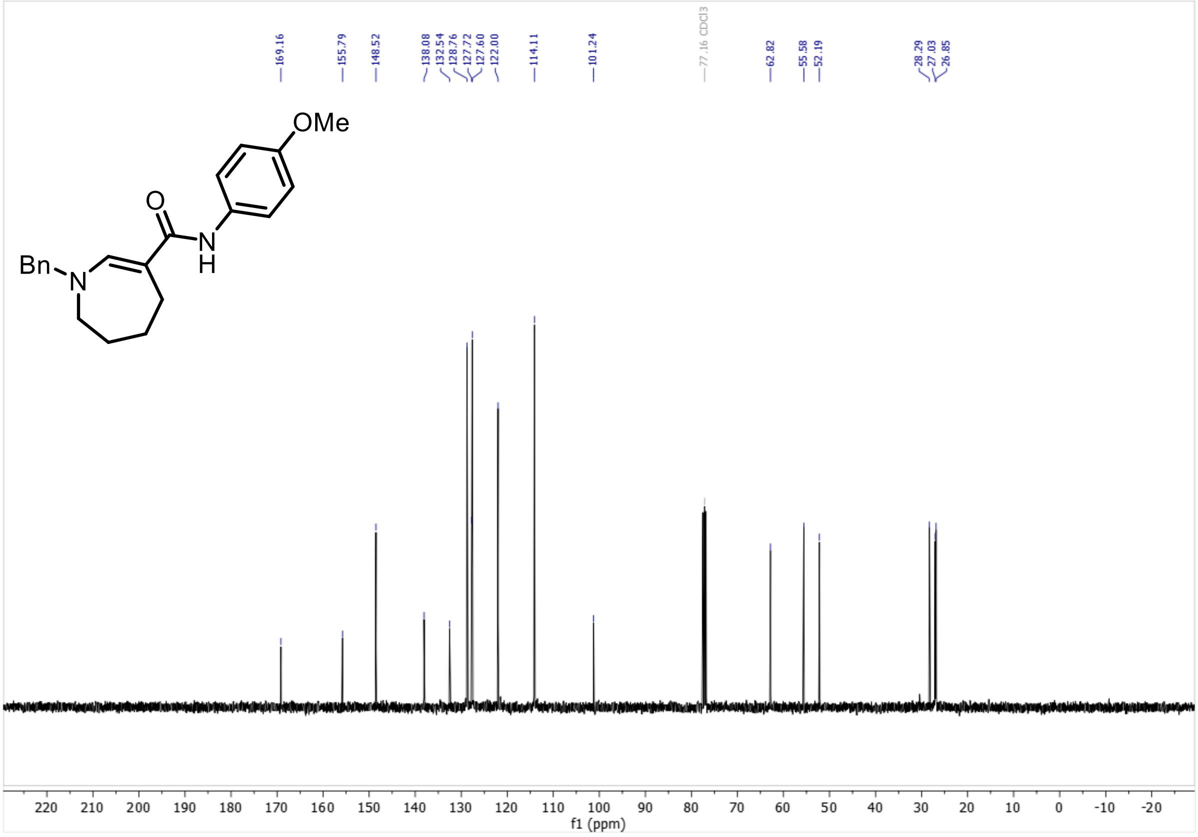

Compound 5s (^{1}H)

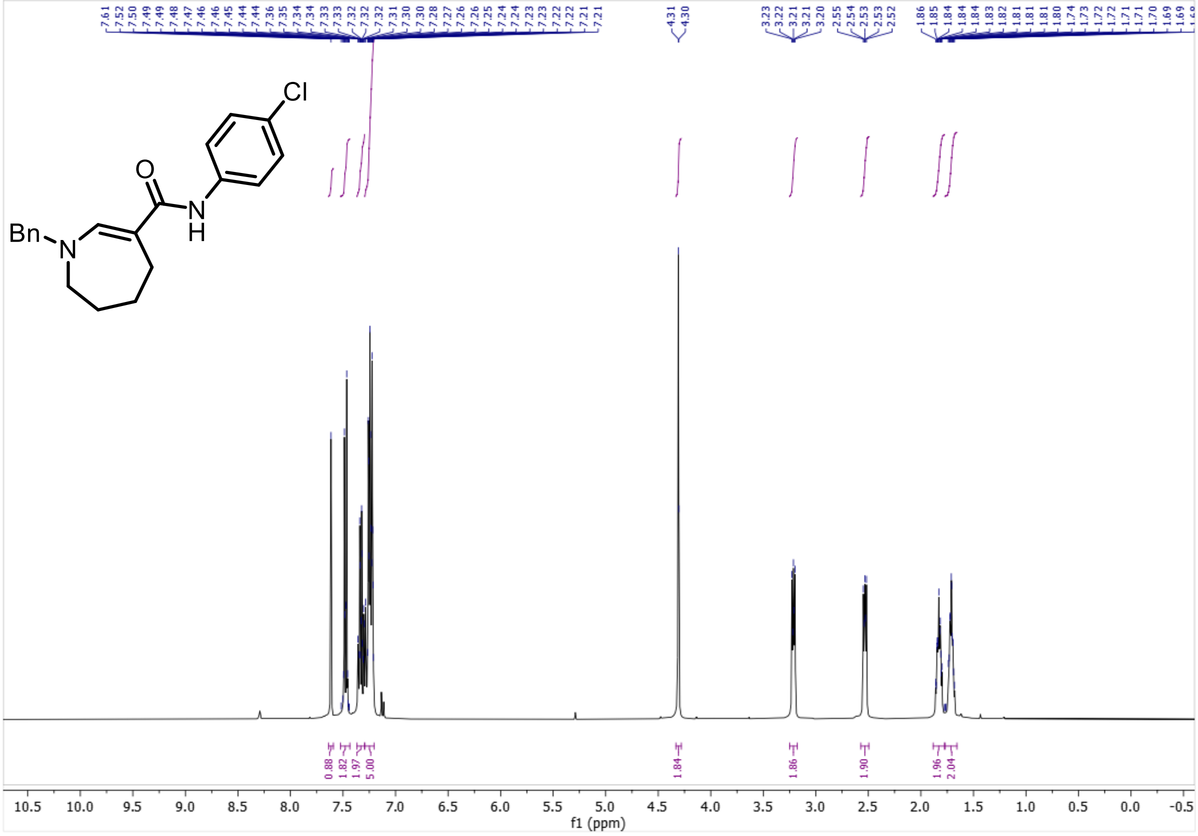

Compound 5s (^{13}C)

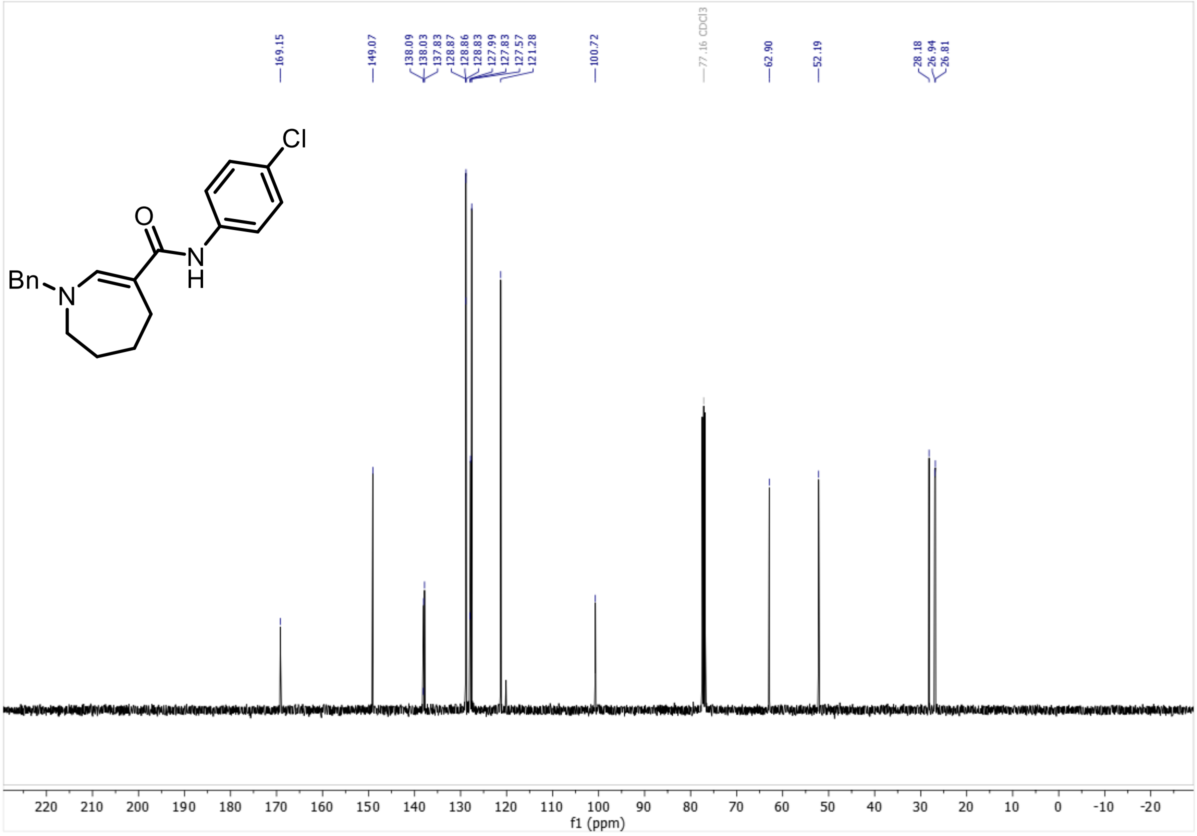

Compound 5t (^{1}H)

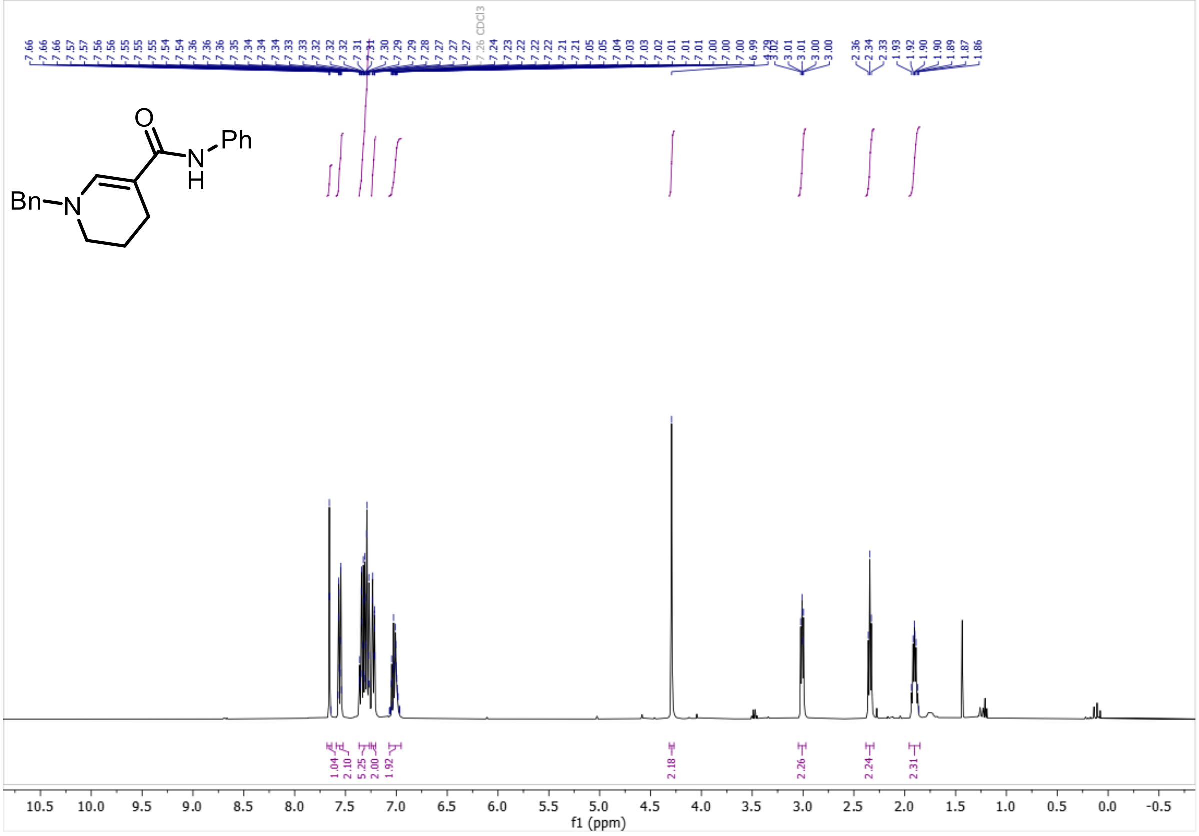

Compound 5t (^{13}C)

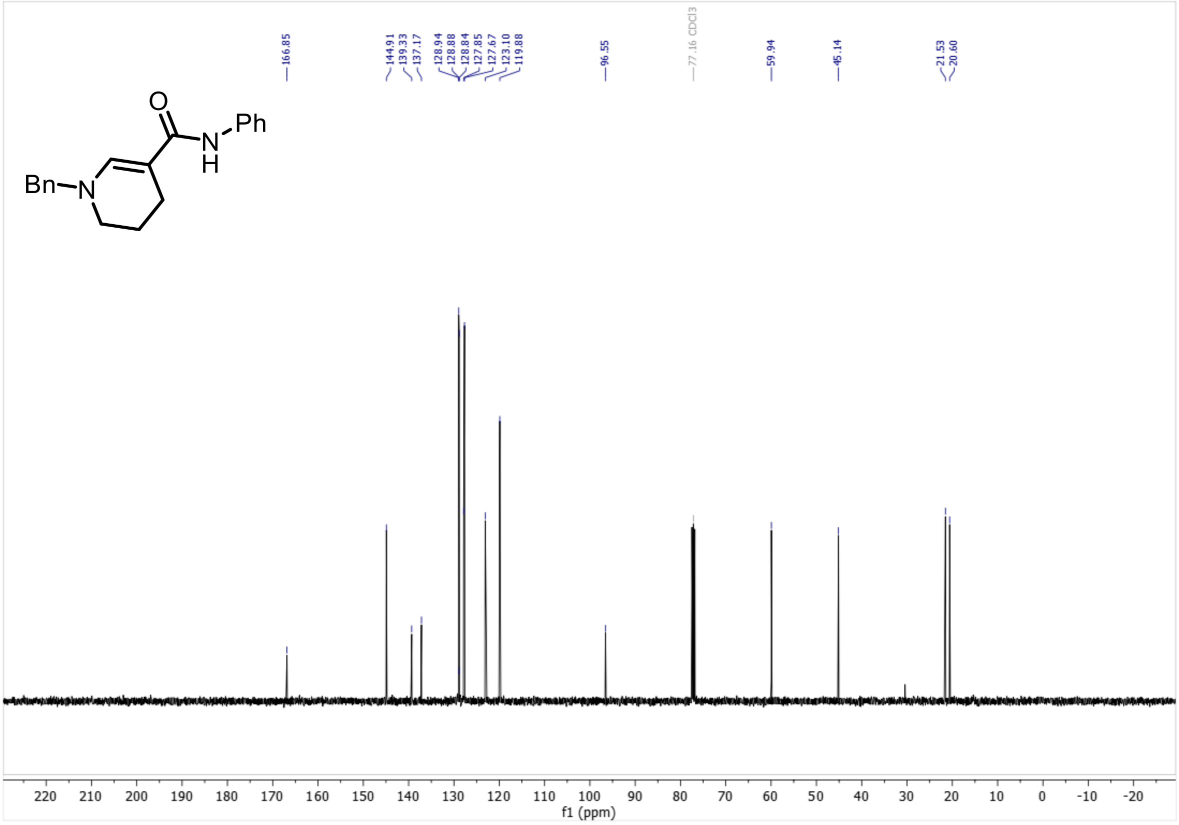

Compound 5u (^{1}H)

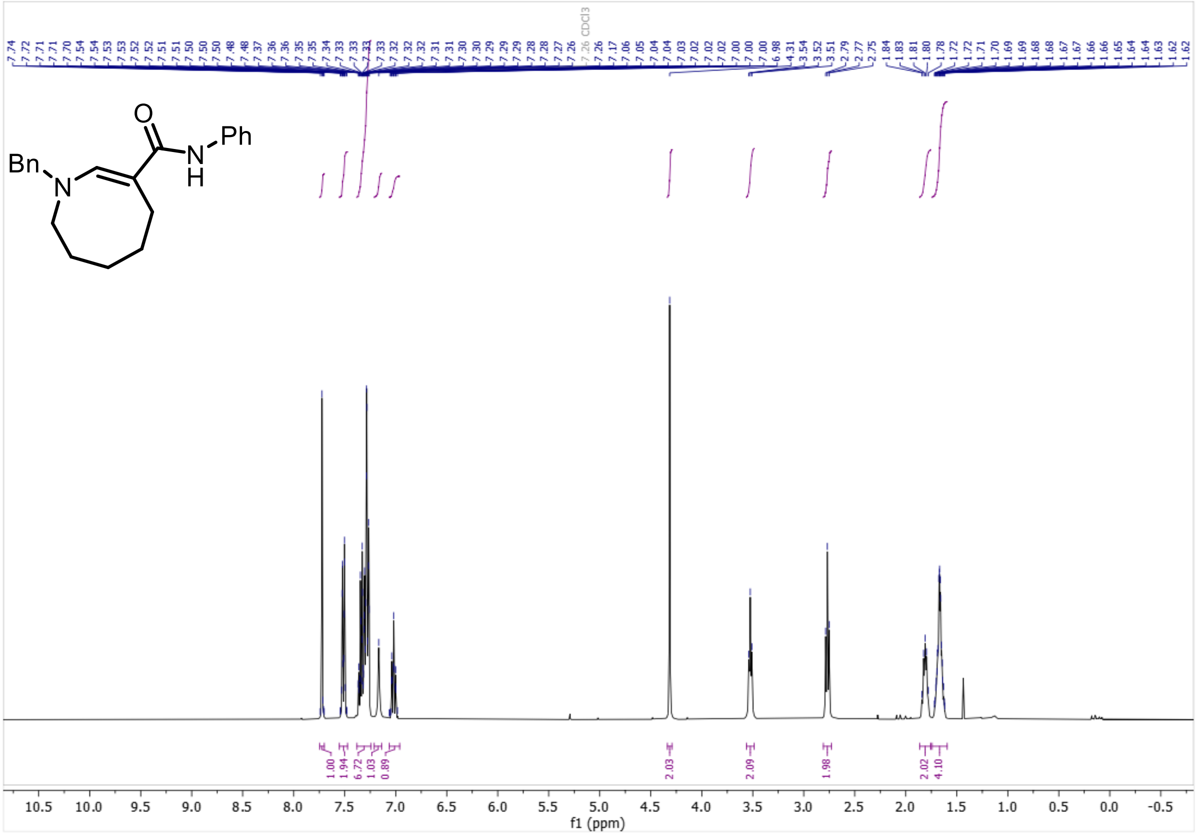

Compound 5u (^{13}C)

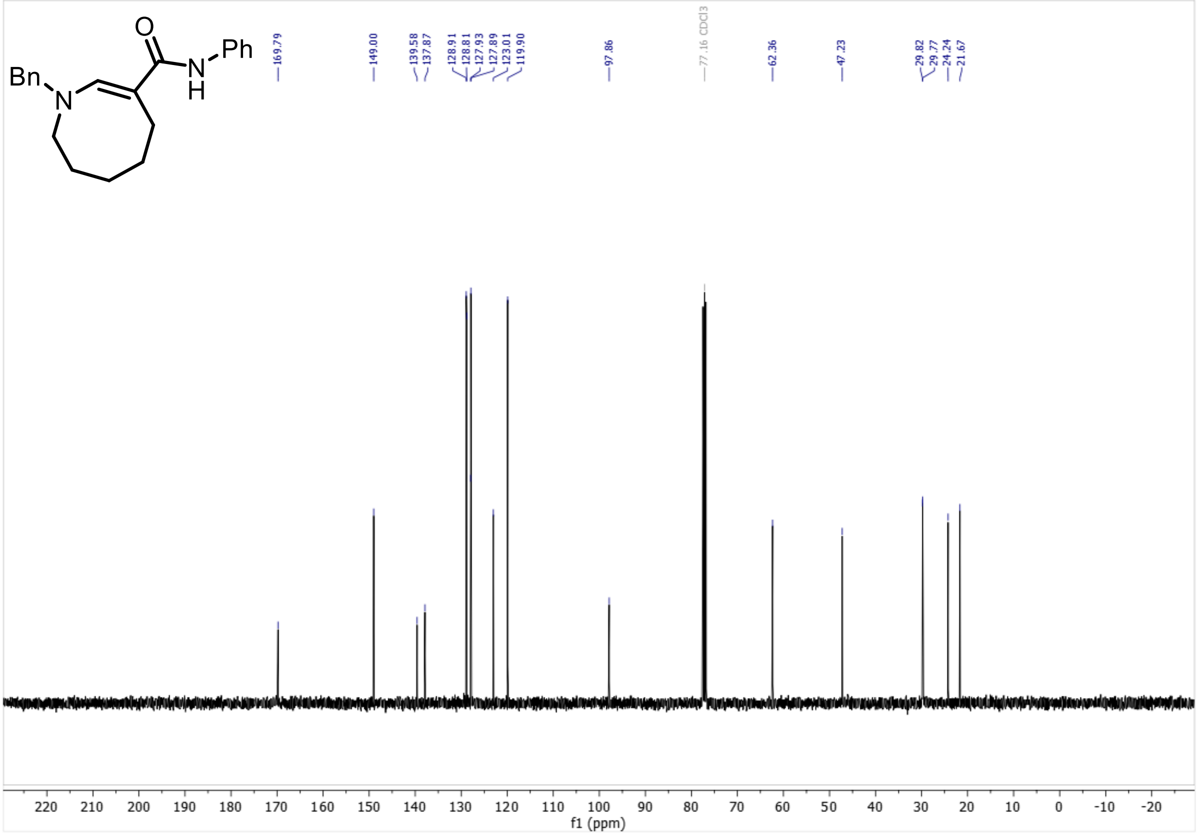

Compound 6a (¹H)

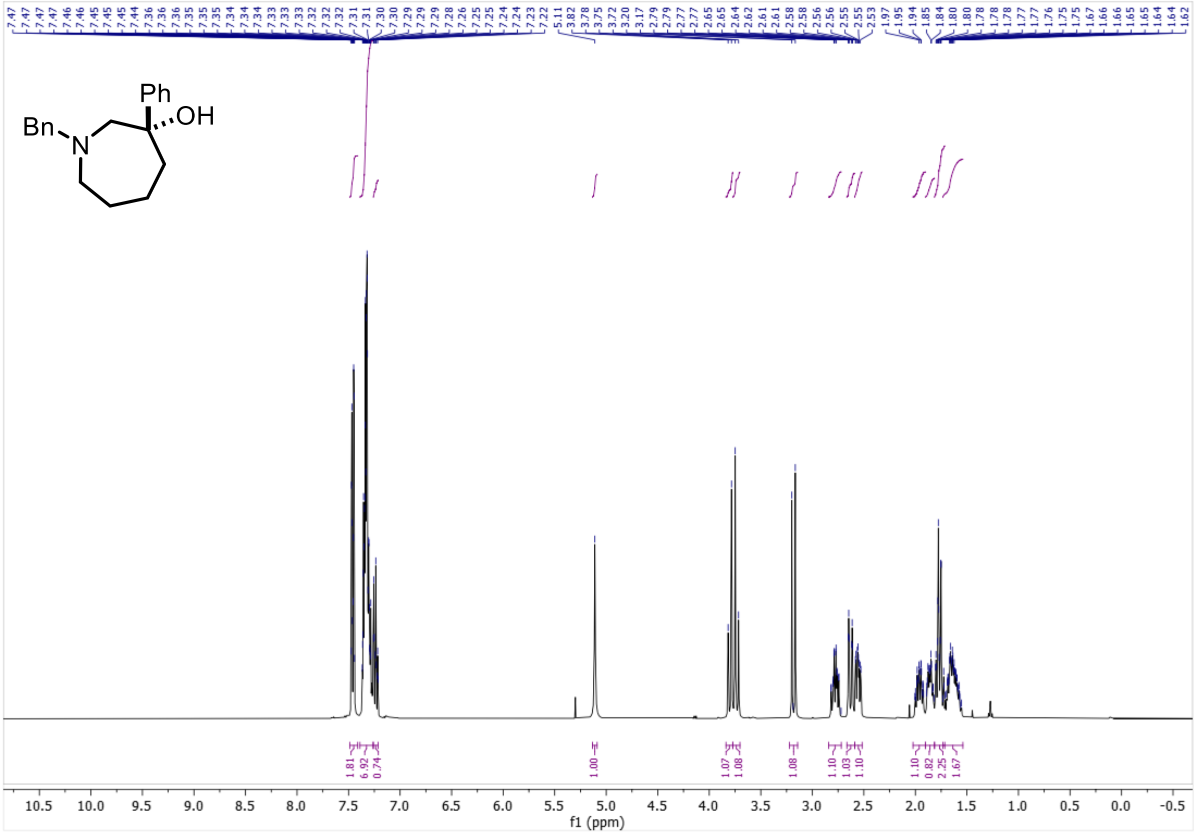

Compound 6a (¹³C)

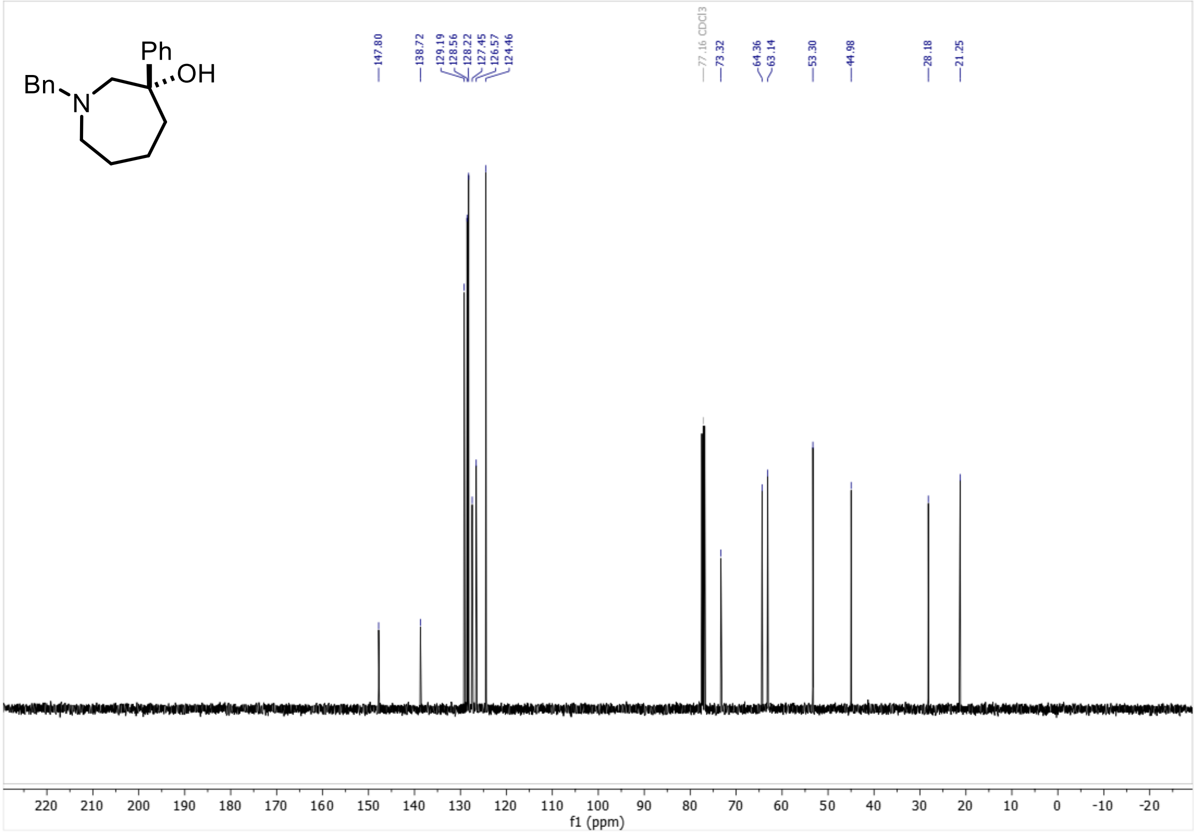

Compound 6b (^{1}H)

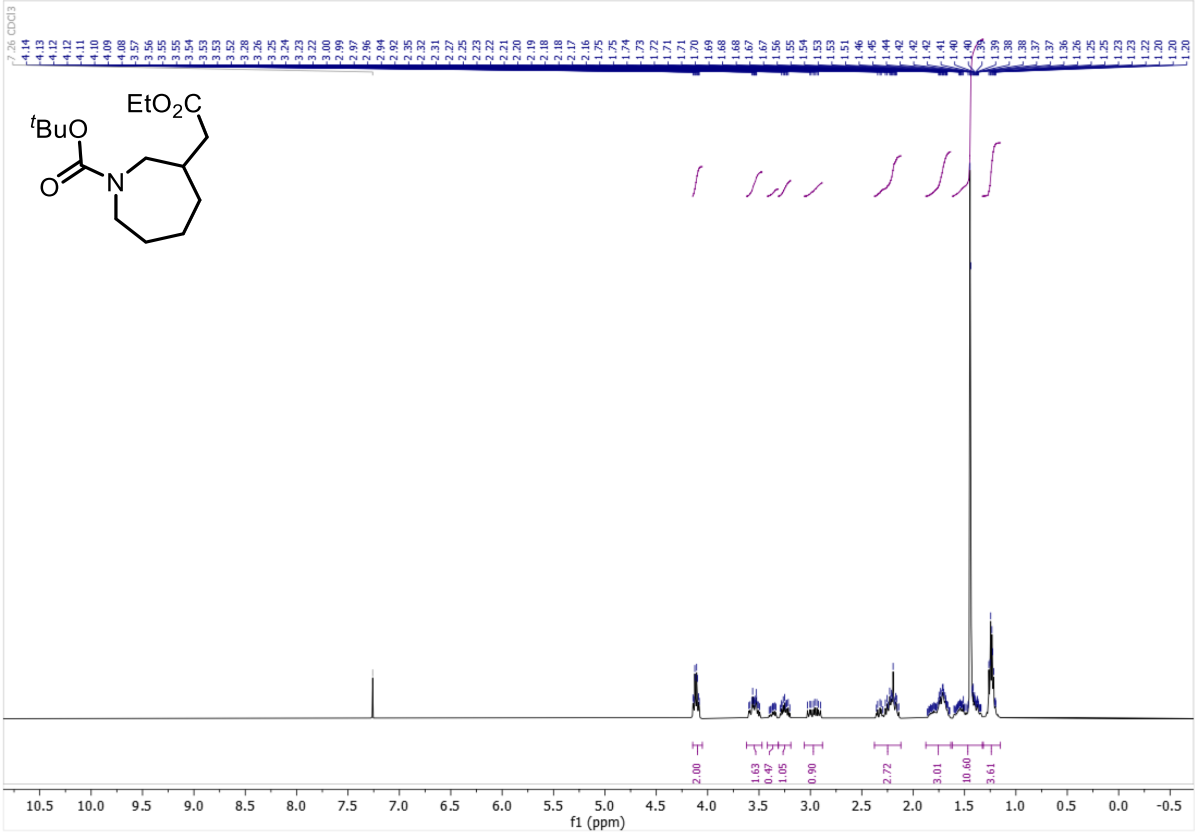

Compound 6b (^{13}C)

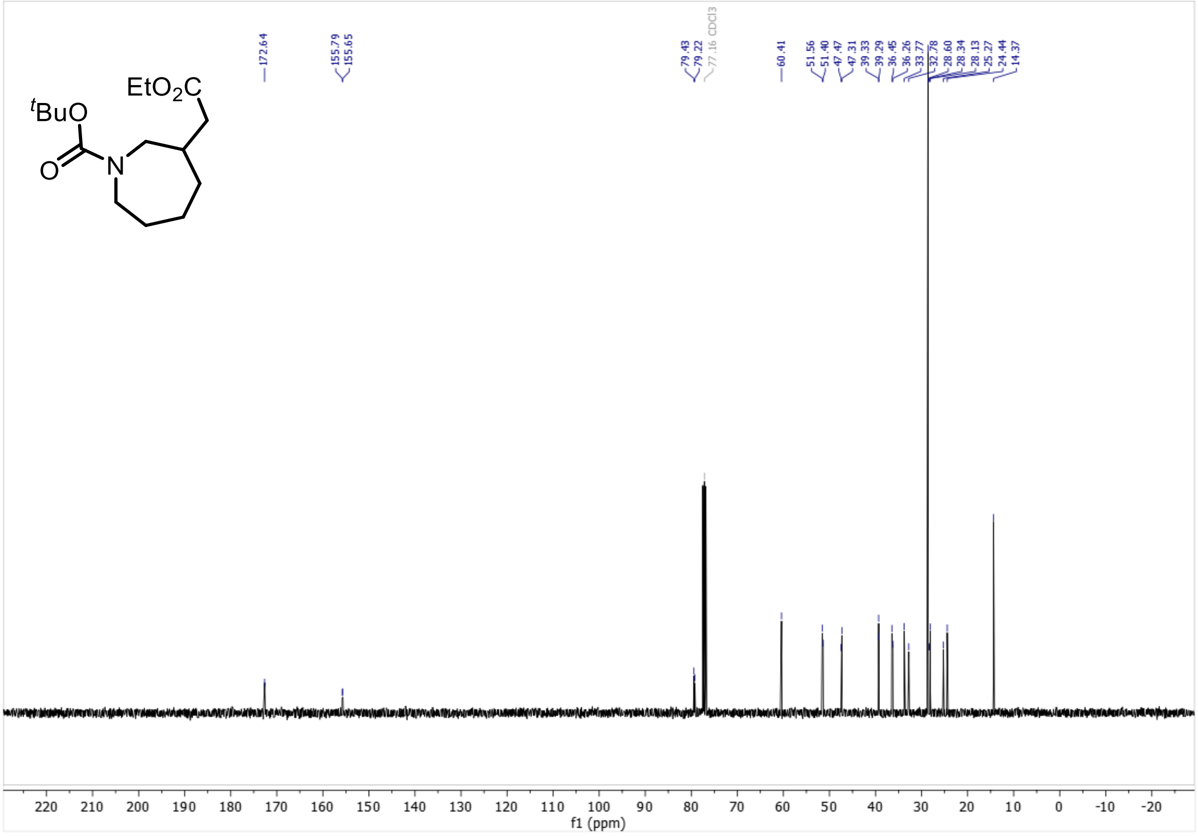

Compound 6c (1H)

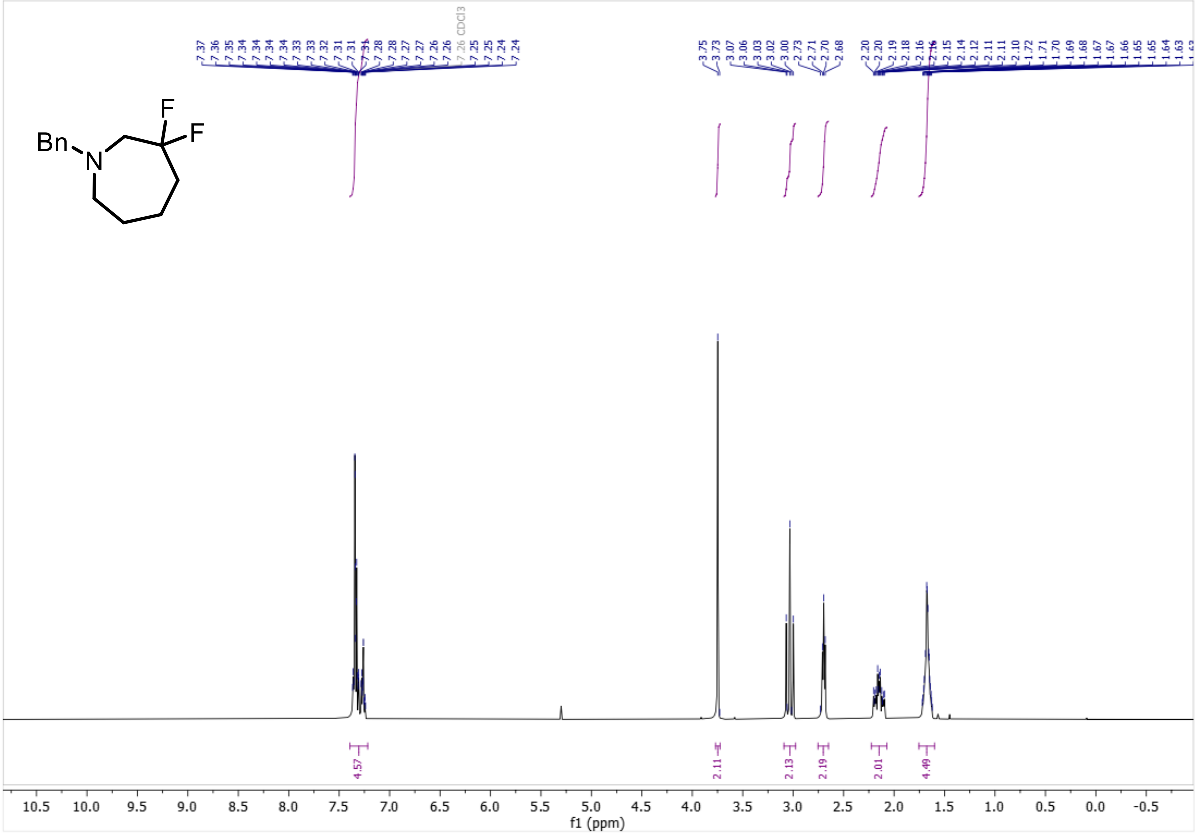

Compound 6c (13C)

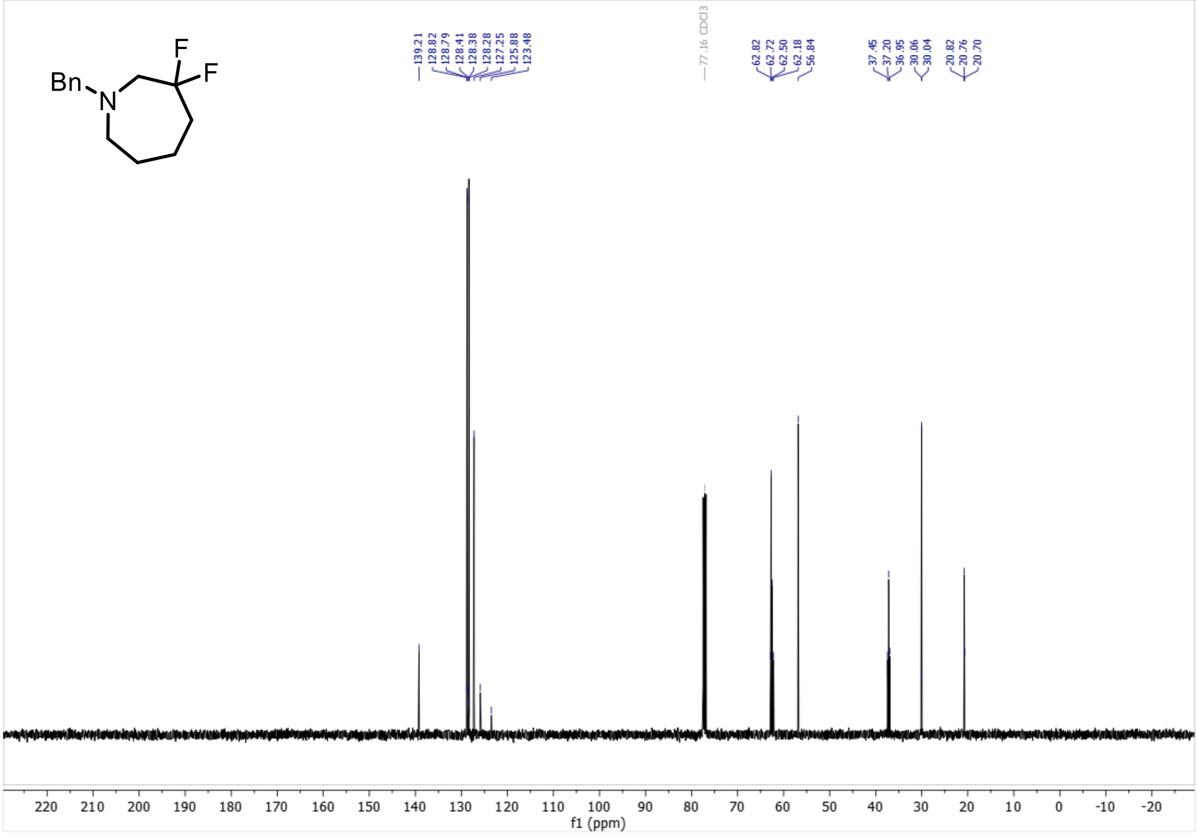

Compound 6d (¹H)

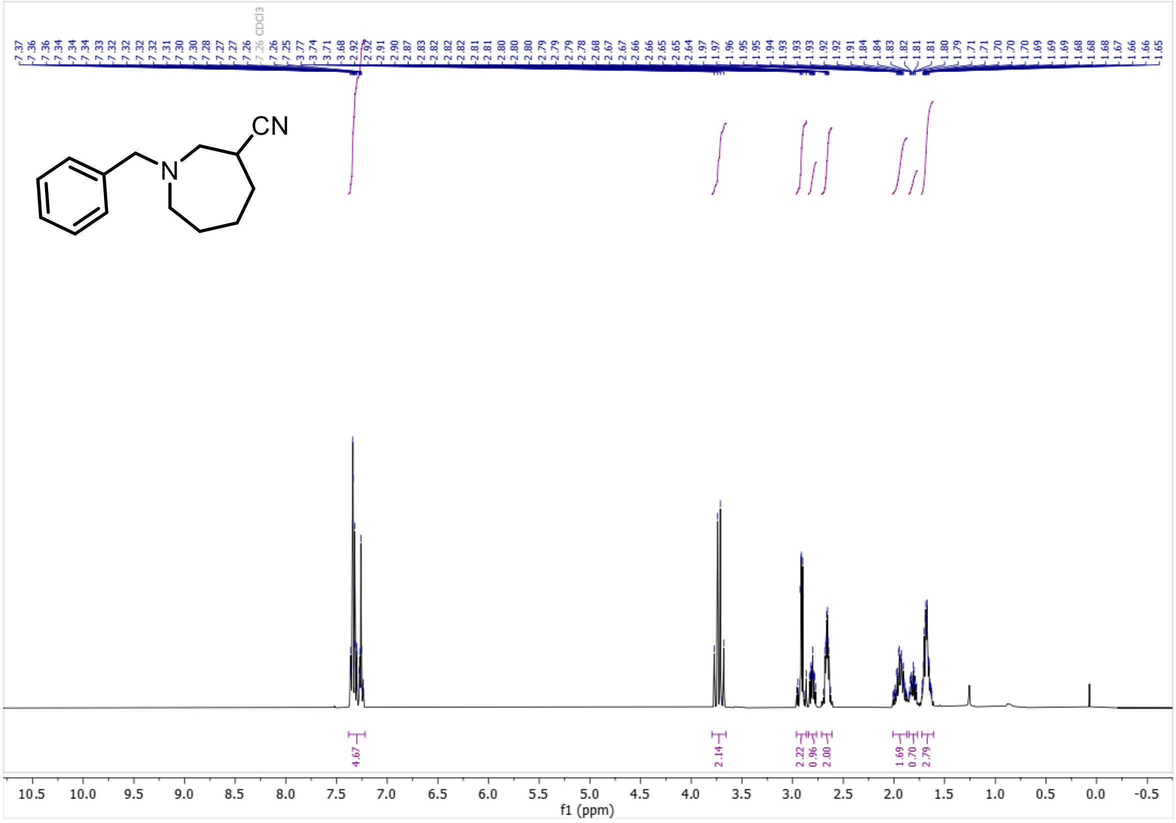

Compound 6d (¹³C)

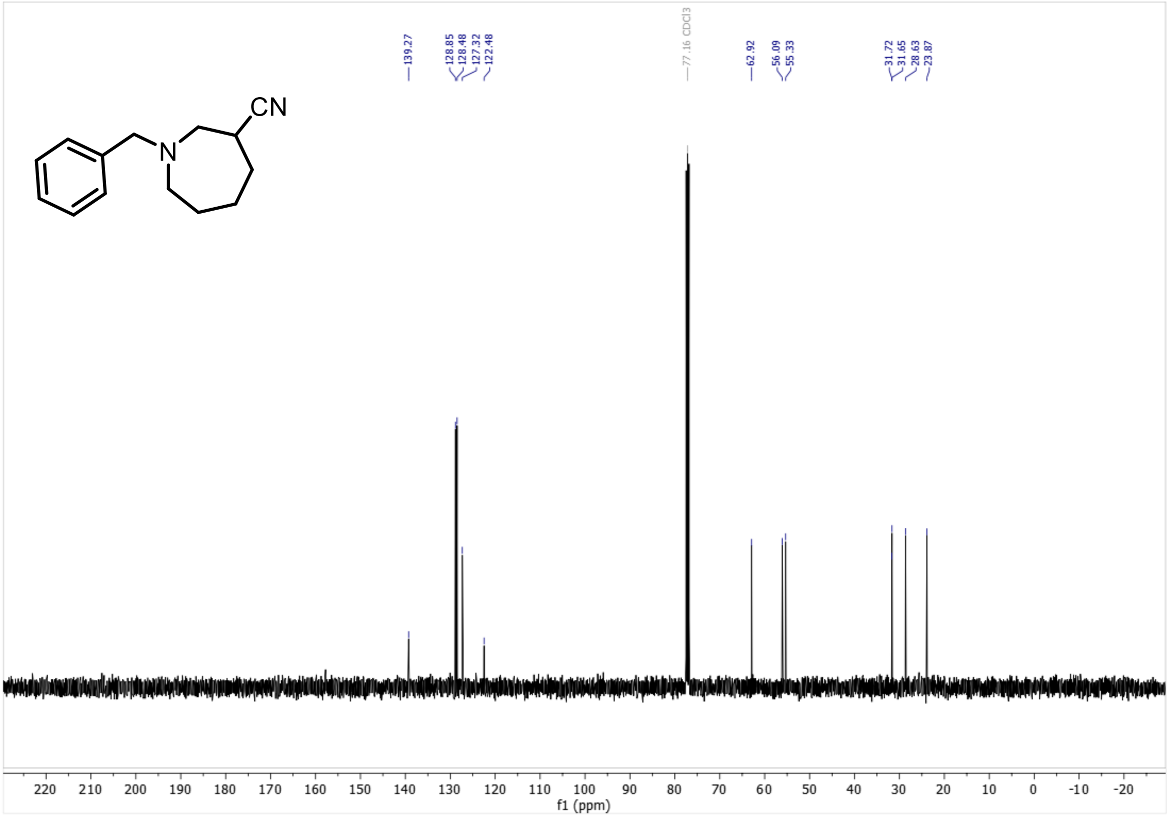

Compound 6e (^{1}H)

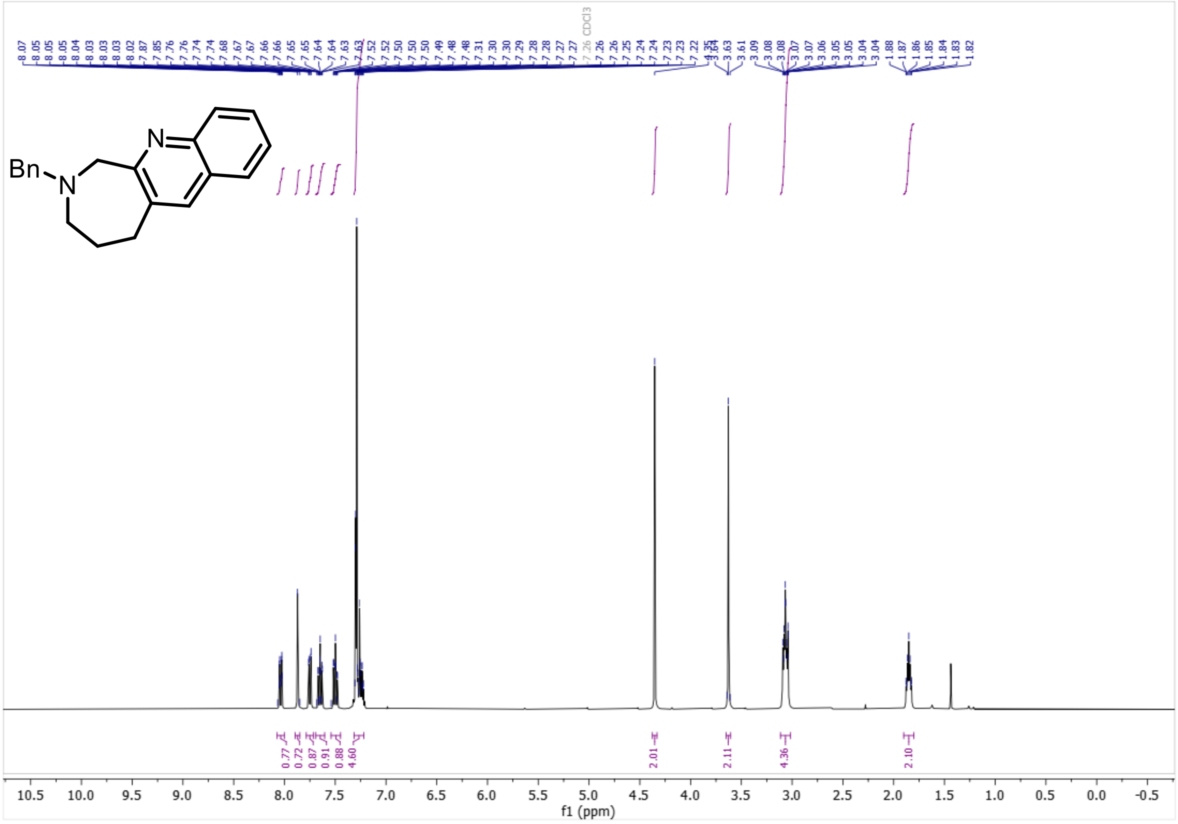

Compound 6e (^{13}C)

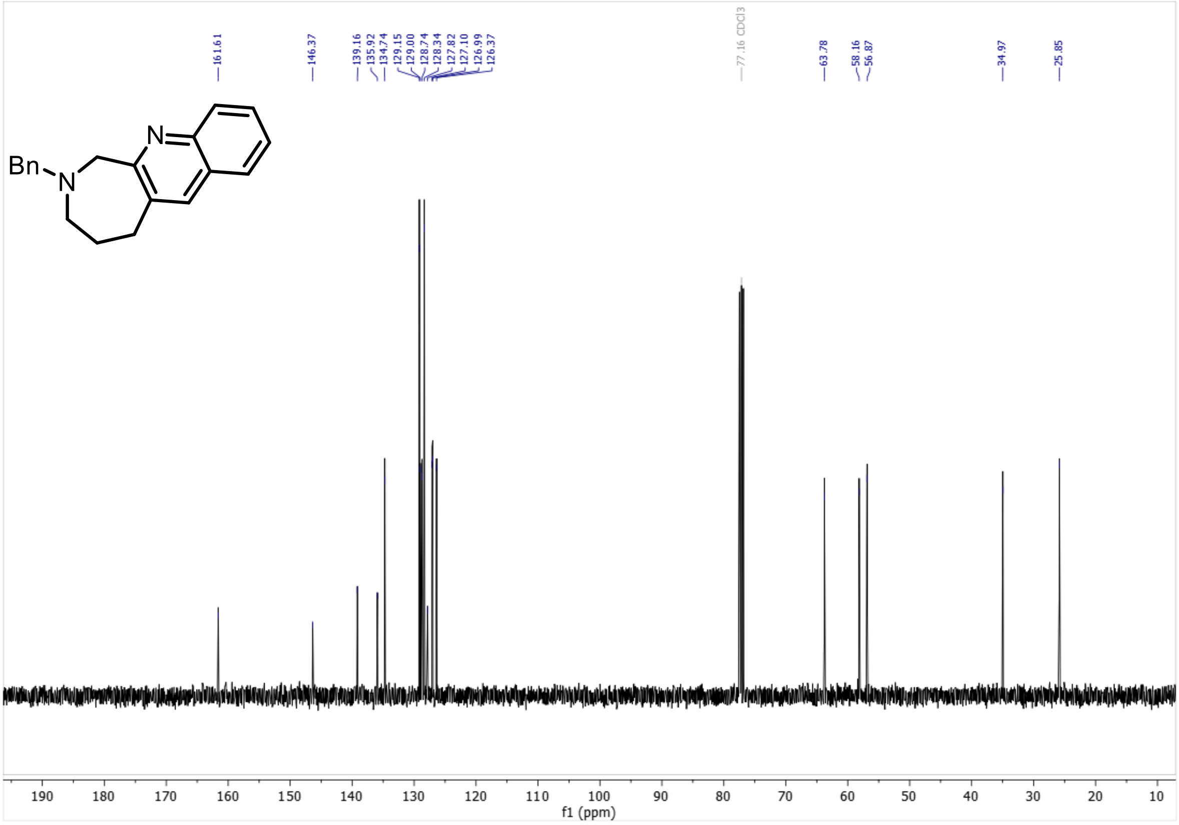

Compound 6f (^{1}H)

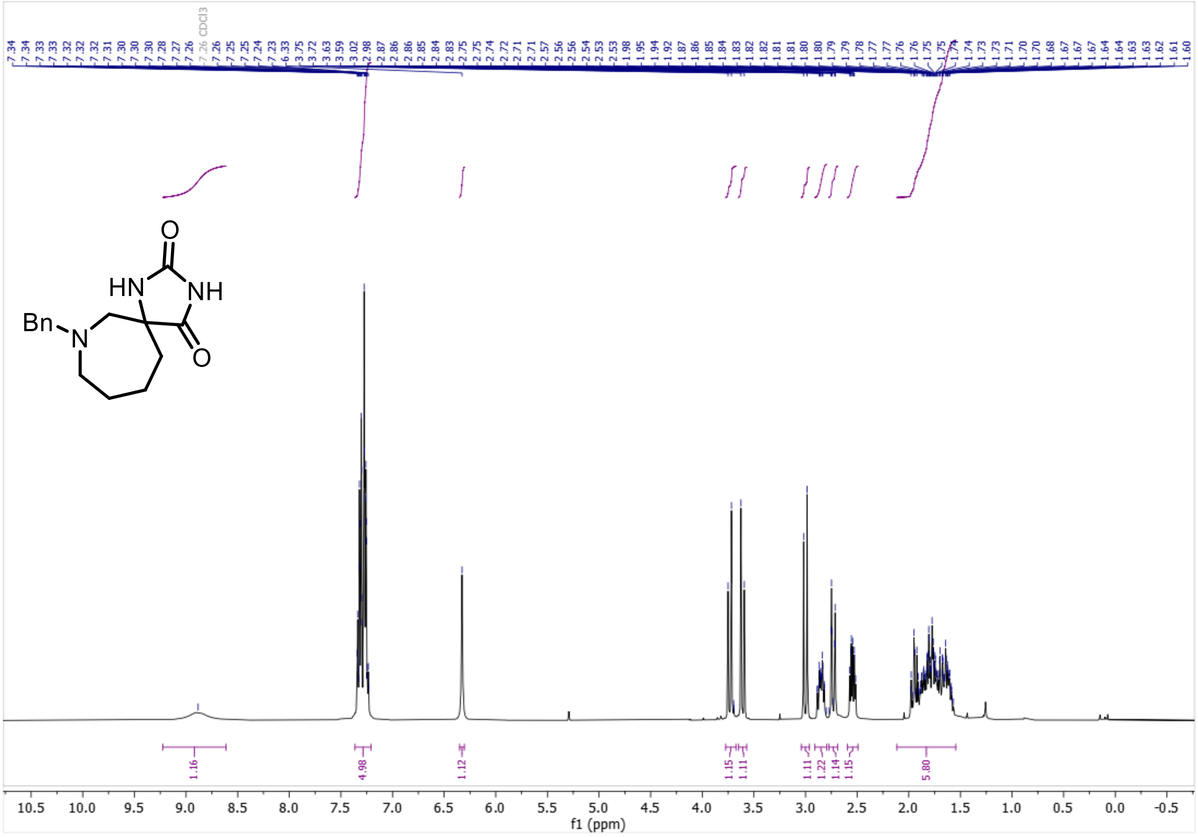

Compound 6f (^{13}C)

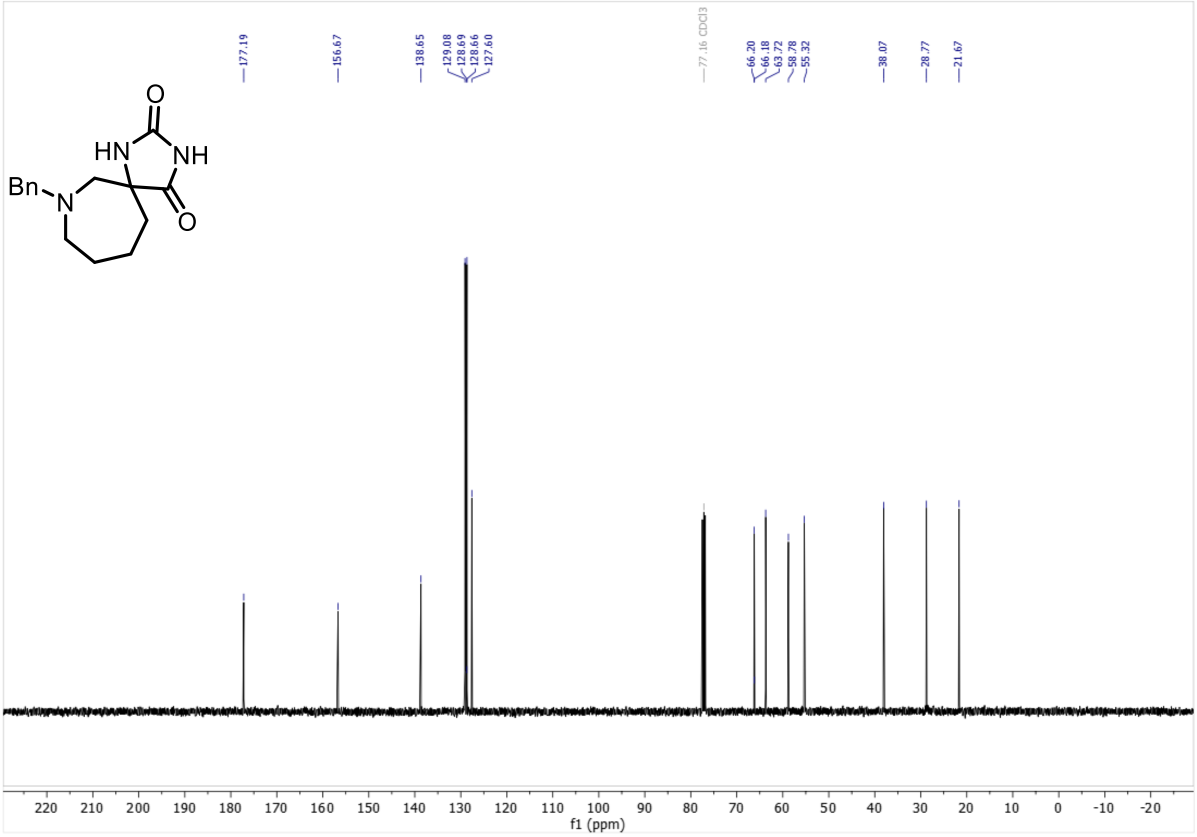

Compound S1-5 (^{1}H)

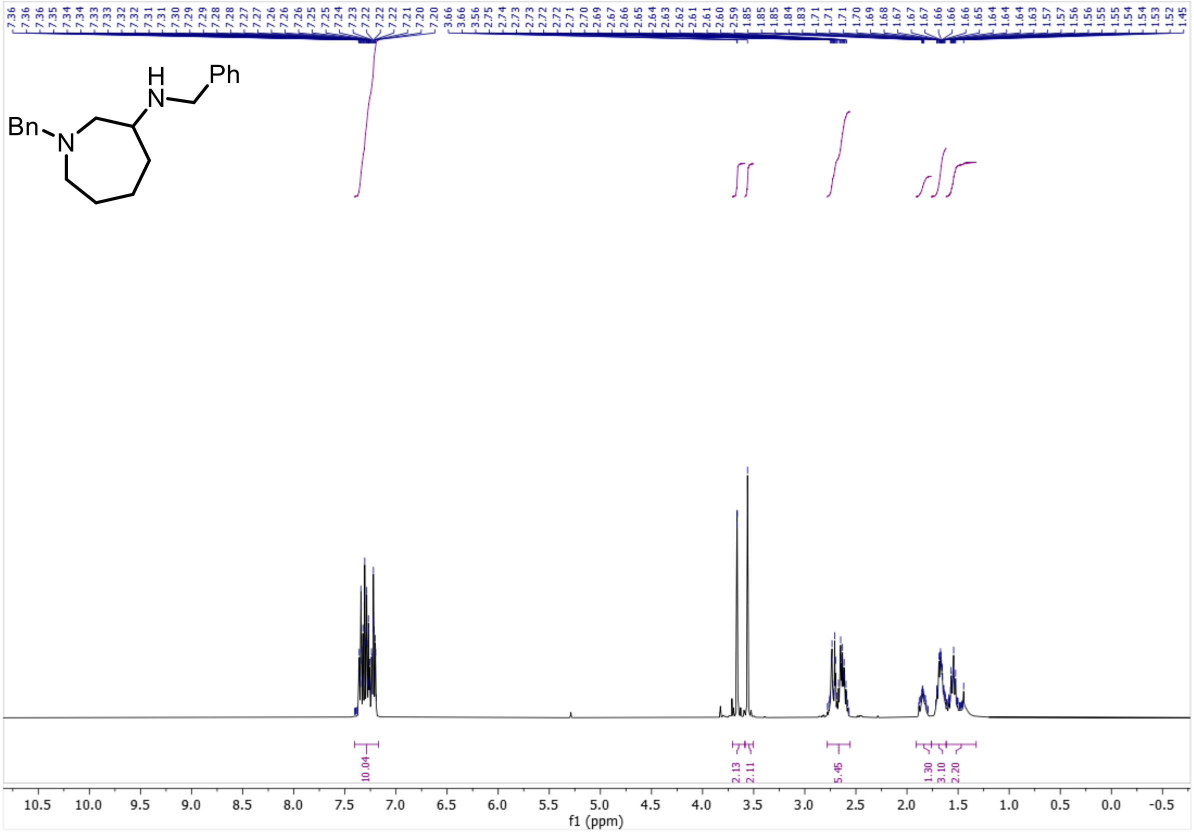

Compound S1-5 (^{13}C)

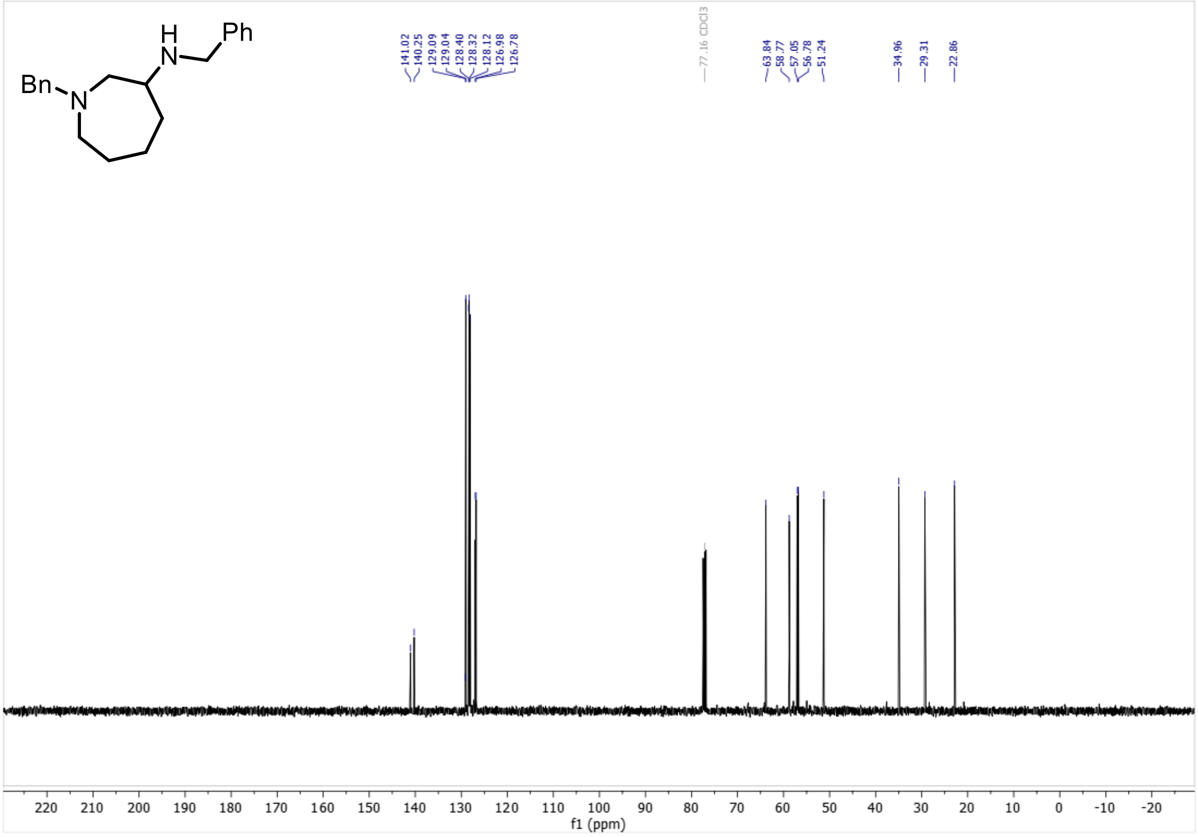

Compound 6h (^{1}H)

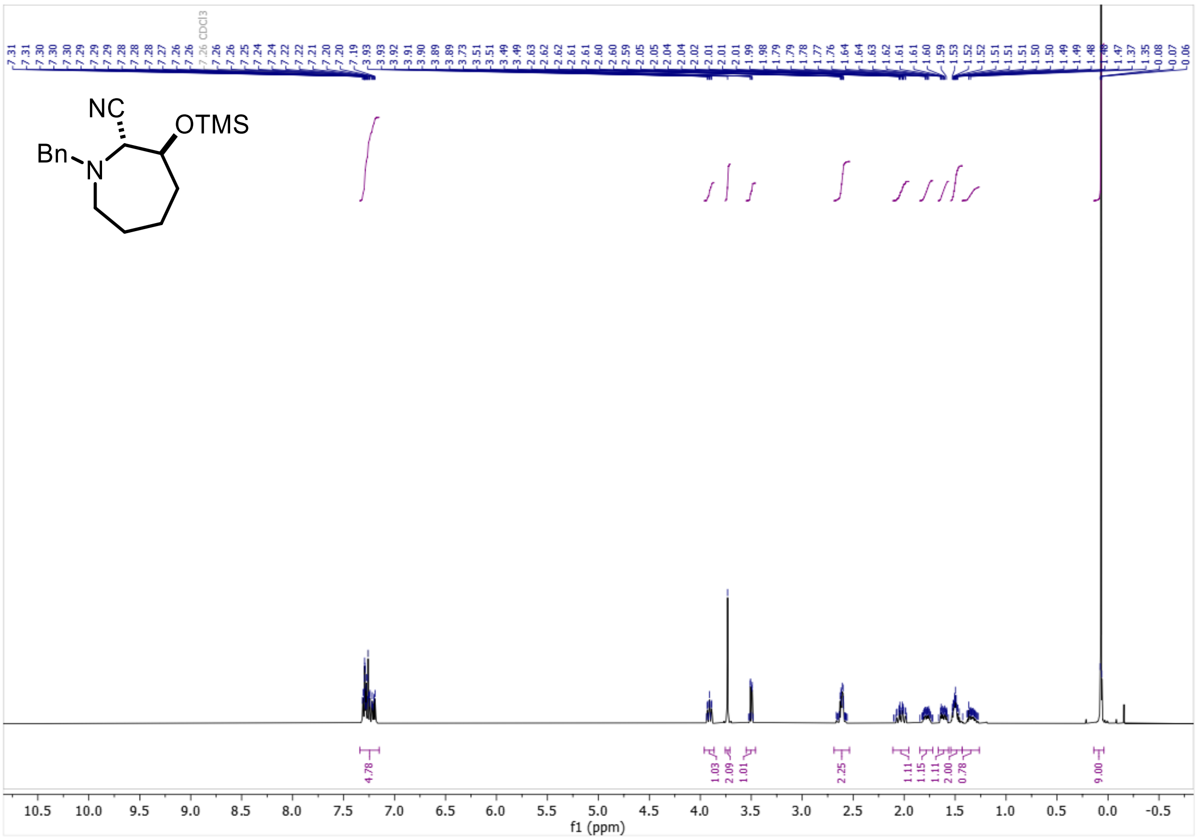

Compound 6h (^{13}C)

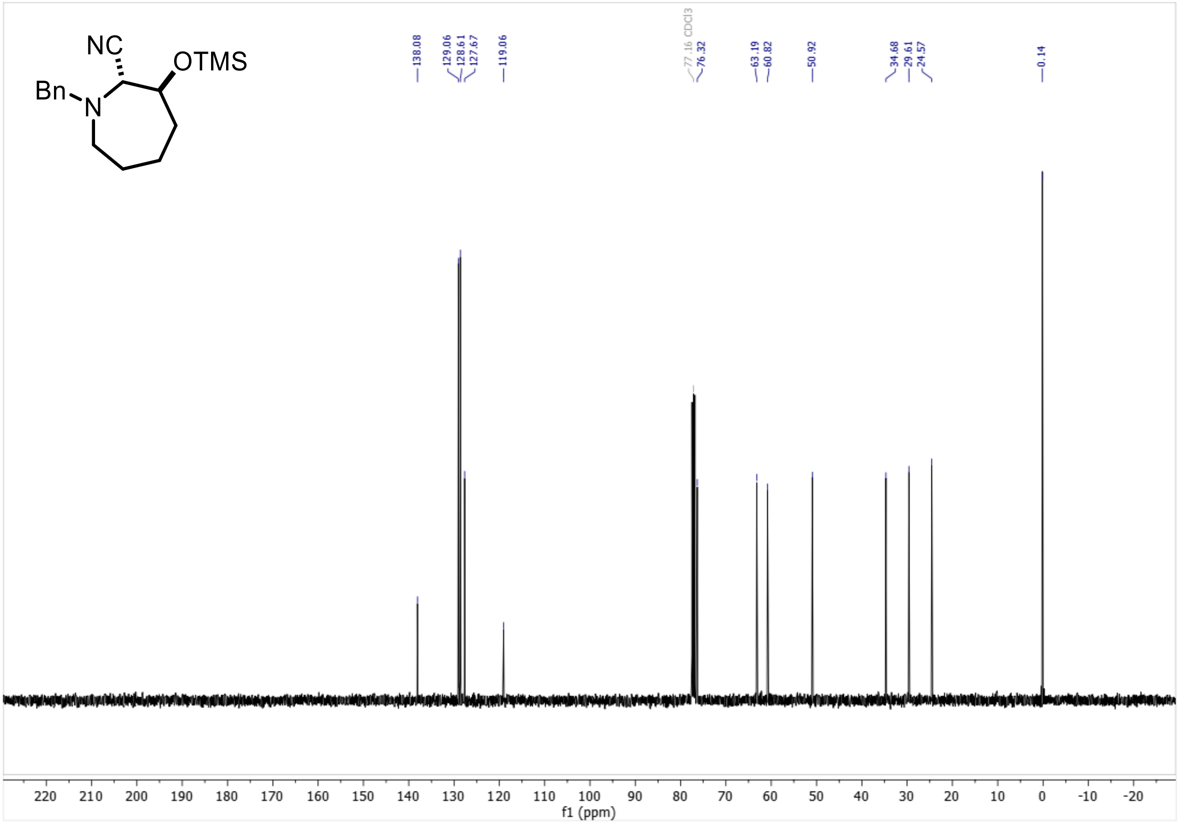

Compound 6i (^{1}H)

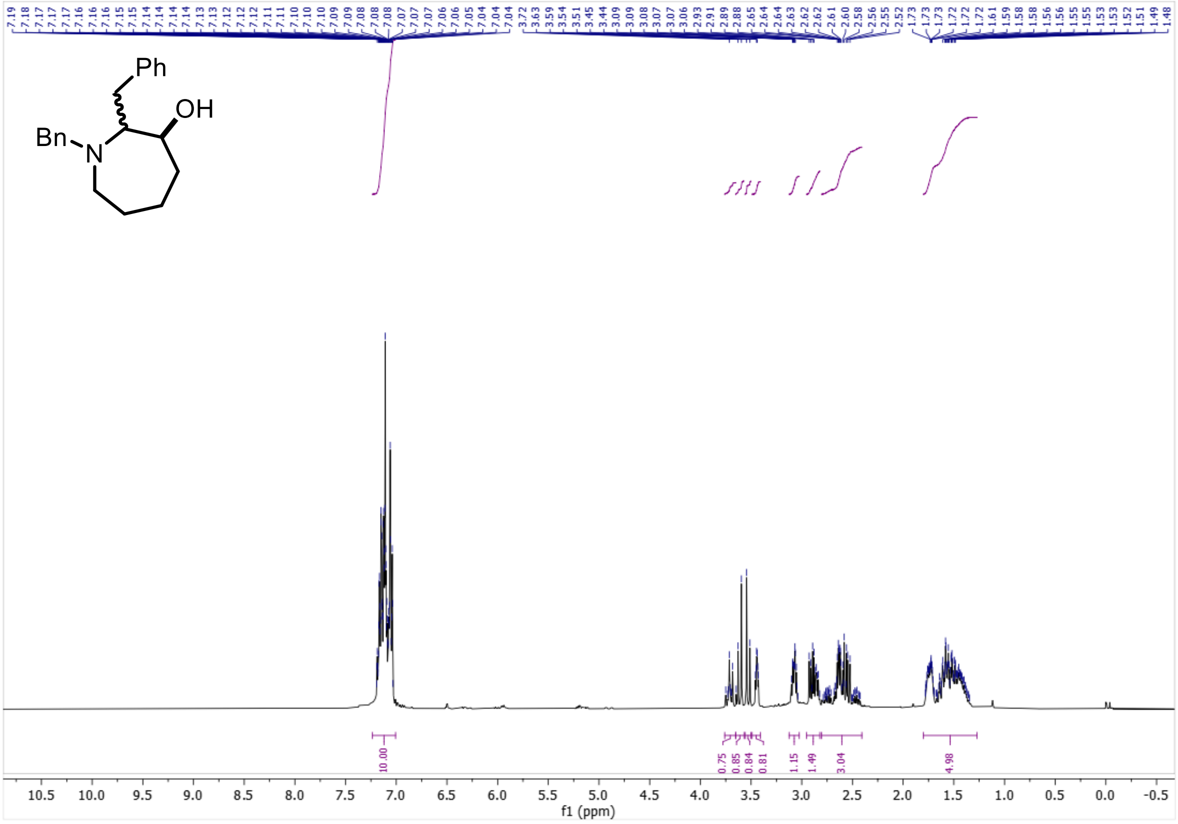

Compound 6i (^{13}C)

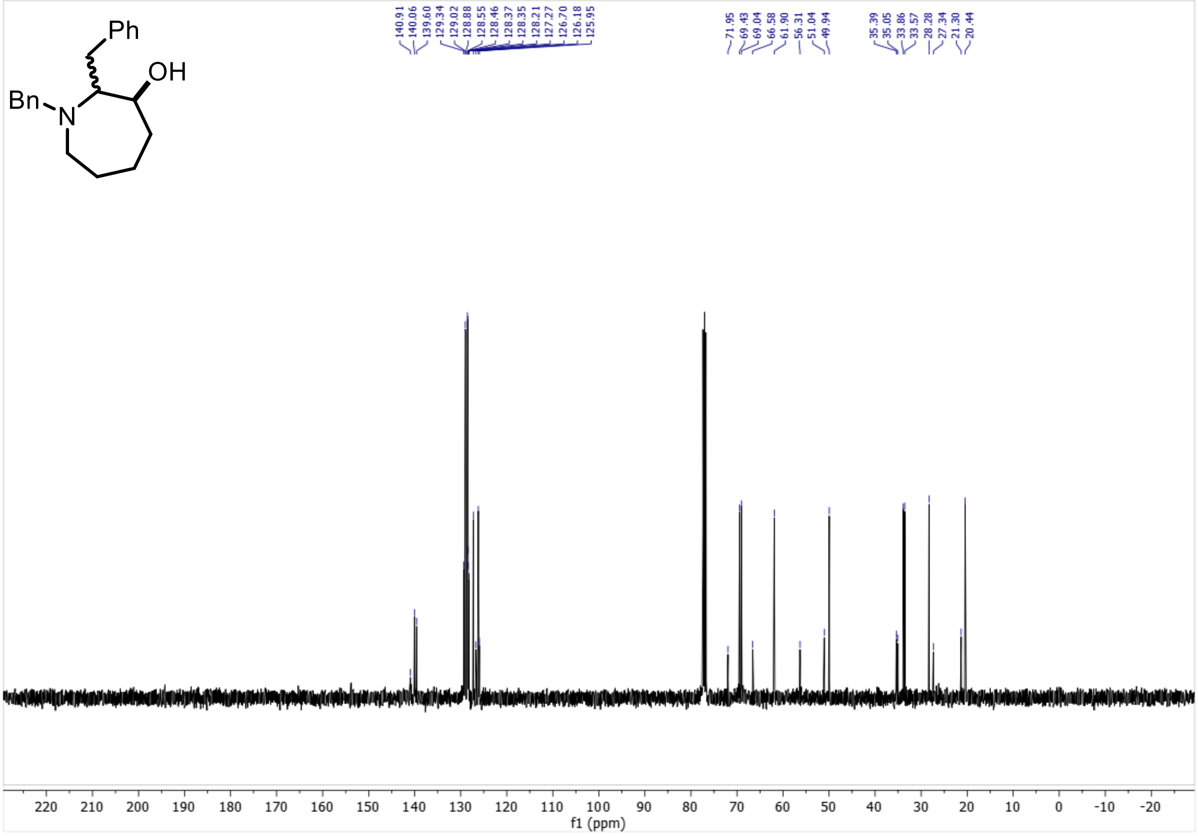

Compound 6j-A (^{1}H)

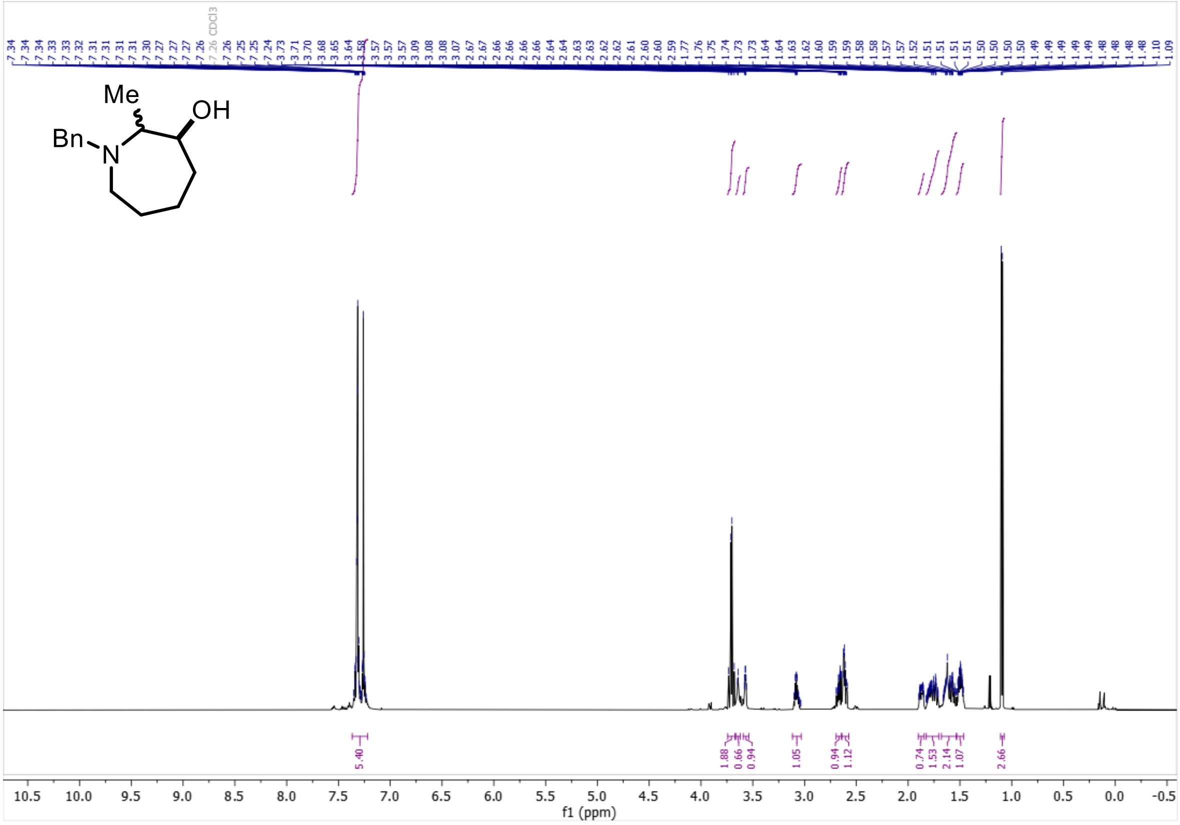

Compound 6j-A (^{13}C)

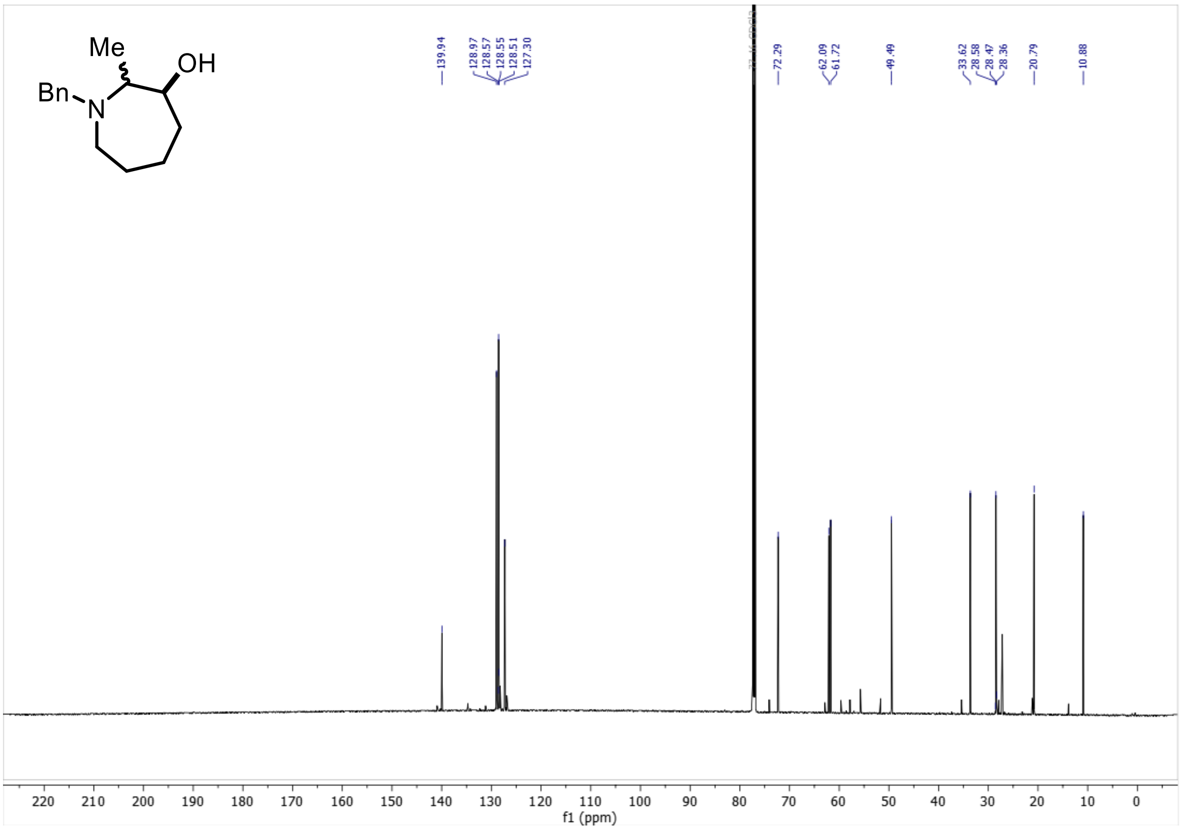

Compound 6j-B (^{1}H)

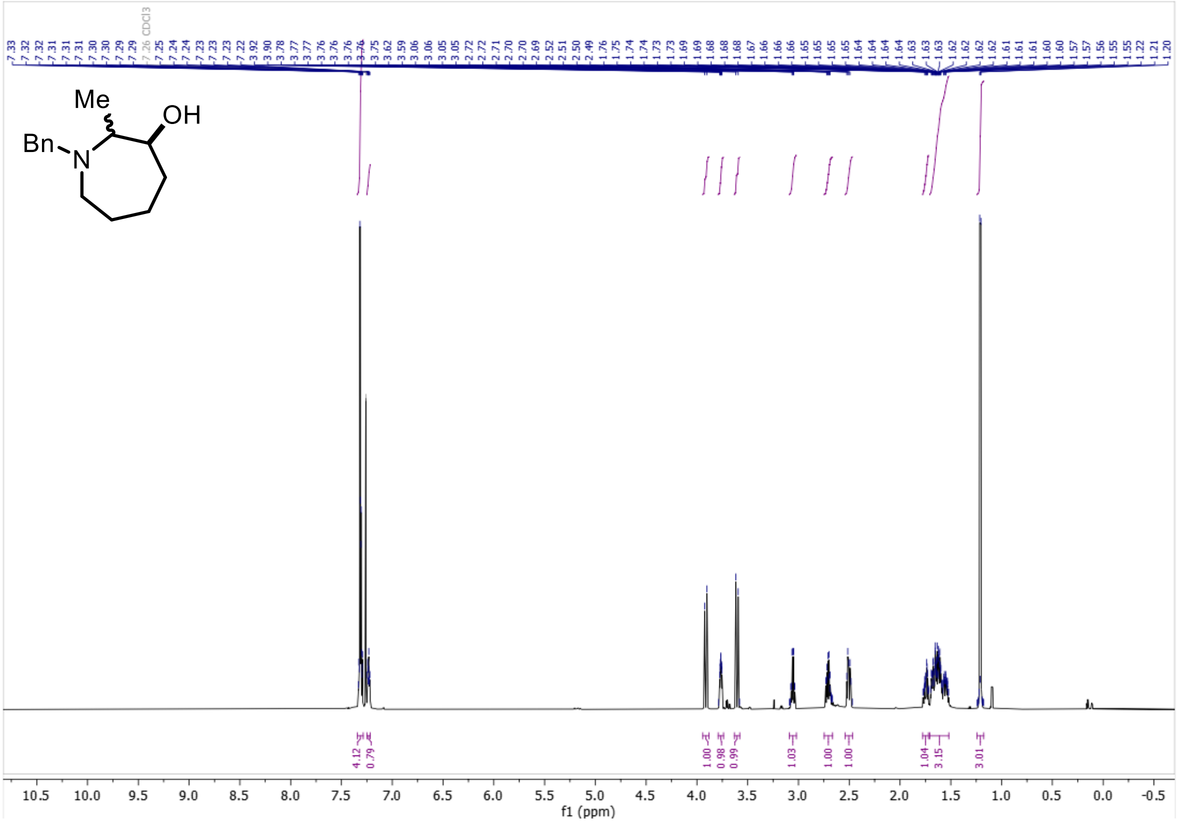

Compound 6j-B (^{13}C)

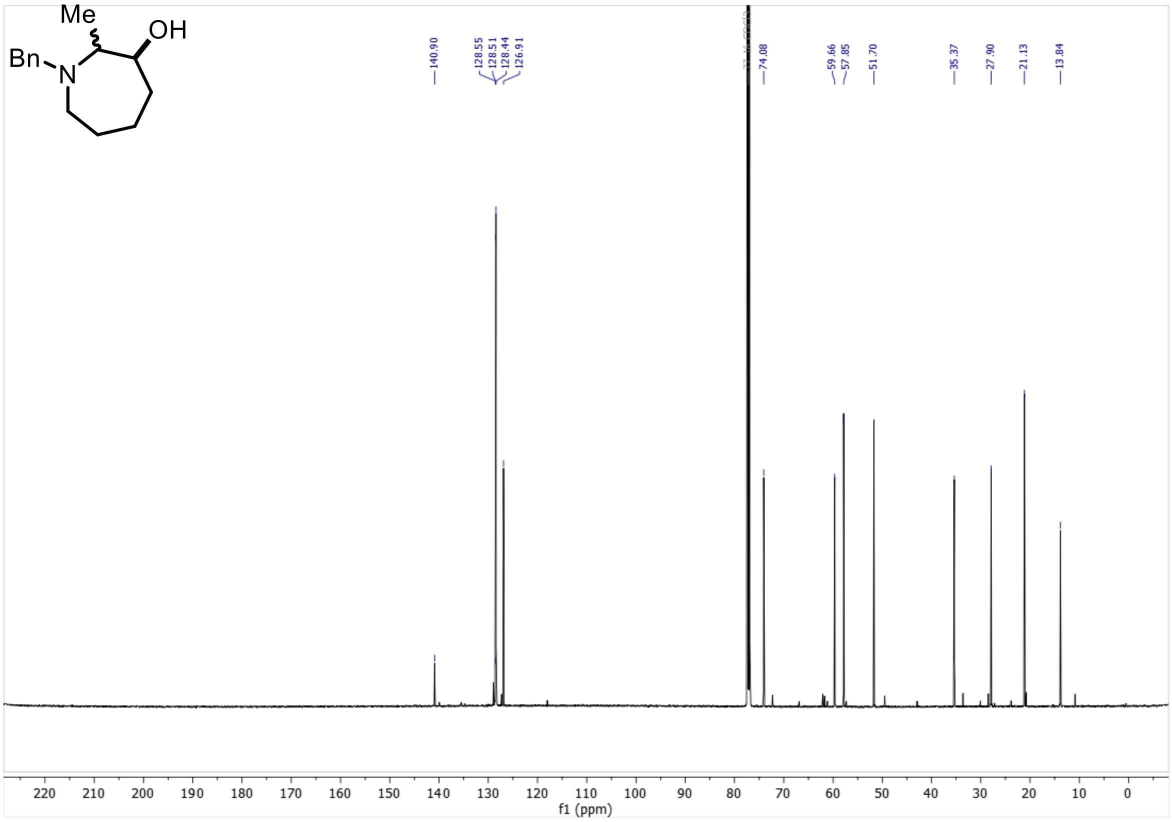

Supplement: Supplementary file 1 — ja3c08466_si_001.pdf [file ja3c08466_si_001.pdf]
